# Cooperative catalytic methoxycarbonylation of alkenes: uncovering the role of palladium complexes with hemilabile ligands†
†Electronic supplementary information (ESI) available. See DOI: 10.1039/c7sc02964k


**DOI:** 10.1039/c7sc02964k

**Published:** 2018-02-07

**Authors:** Kaiwu Dong, Rui Sang, Zhihong Wei, Jie Liu, Ricarda Dühren, Anke Spannenberg, Haijun Jiao, Helfried Neumann, Ralf Jackstell, Robert Franke, Matthias Beller

**Affiliations:** a Leibniz-Institut für Katalyse e.V. an der Universität Rostock , Albert-Einstein Straße 29a , Rostock , 18059 , Germany . Email: matthias.beller@catalysis.de; b Evonik Performance Materials GmbH , Paul-Baumann-Str. 1 , 45772 Marl , Germany; c Lehrstuhl für Theoretische Chemie , Ruhr-Universität Bochum , 44780 Bochum , Germany

## Abstract

Mechanistic studies of the catalyst [Pd_2_(dba)_3_/1,1′-bis(*tert*-butyl(pyridin-2-yl)phosphanyl)ferrocene, **L2**] for olefin alkoxycarbonylation reactions are described. X-ray crystallography reveals the coordination of the pyridyl nitrogen atom in **L2** to the palladium center of the catalytic intermediates. DFT calculations on the elementary steps of the industrially relevant carbonylation of ethylene (the Lucite α-process) indicate that the protonated pyridyl moiety is formed immediately, which facilitates the formation of the active palladium hydride complex. The insertion of ethylene and CO into this intermediate leads to the corresponding palladium acyl species, which is kinetically reversible. Notably, this key species is stabilized by the hemilabile coordination of the pyridyl nitrogen atom in **L2**. The rate-determining alcoholysis of the acyl palladium complex is substantially facilitated by metal–ligand cooperation. Specifically, the deprotonation of the alcohol by the built-in base of the ligand allows a facile intramolecular nucleophilic attack on the acyl palladium species concertedly. Kinetic measurements support this mechanistic proposal and show that the rate of the carbonylation step is zero-order dependent on ethylene and CO. Comparing CH_3_OD and CH_3_OH as nucleophiles suggests the involvement of (de)protonation in the rate-determining step.

## Abstract 2

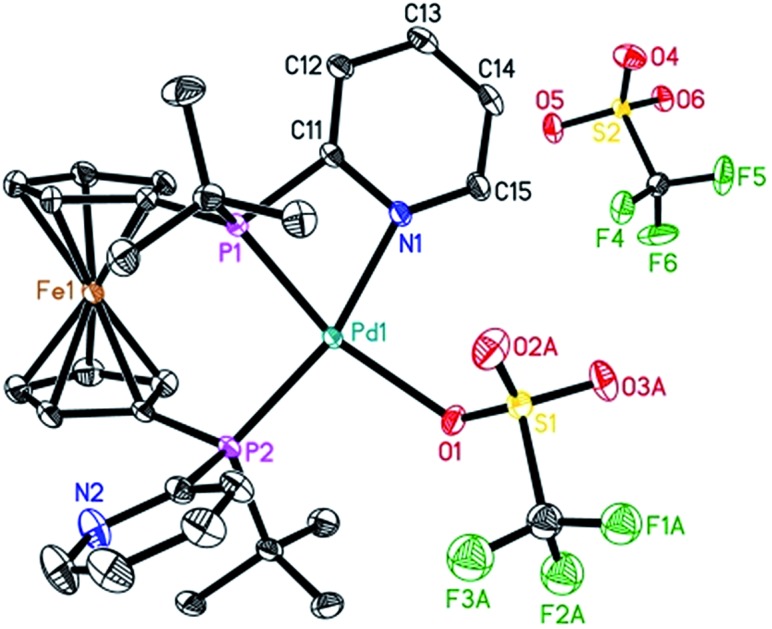
Mechanistic studies of the catalyst [Pd_2_(dba)_3_/1,1′-bis(*tert*-butyl(pyridin-2-yl)phosphanyl)ferrocene, **L2**] for olefin alkoxycarbonylation reactions are described.

## 1. Introduction

Transition metal complexes play a crucial role in homogeneous catalysis both for basic organic synthesis and on a large scale in the chemical industry.^1^ In general, elementary catalytic steps occur at a given metal center and the stabilizing ligands remain untouched during the reaction. By tuning the electronic and steric properties of the ligands, the activity and/or selectivity at the metal center can be adjusted. In contrast to this classic scenario, metal–ligand cooperative catalysis requires the ligand to take part in the activation and/or recognition of the substrates or intermediates.^2^ For a given catalytic cycle, this strategy is a powerful tool to accelerate the rate-limiting step by lowering the energy of the key transition state. This multifunctional catalysis, which mimicks natural enzymes, opens up new avenues to synergistically activate small molecules and develop new and potentially ‘greener’ catalytic processes.^3^

Hence, in the past two decades, several types of ligand have been developed for this purpose. Typically, substituents such as amino, carboxylate and hydroxyl groups are introduced at a specific position of the ligand.^4^ Most of these systems have been favorably applied for catalytic (de)hydrogenation reactions.4a–f,^5^

In order to expand the use of this compelling concept, we became interested in its application for carbonylation reactions. In fact, the hydroformylation and alkoxycarbonylation of alkenes constitute the most common methodologies in industry to synthesize aliphatic oxygenated compounds,^6^ which are found widely in our daily life. As a representative example, the palladium/**L1**-catalyzed methoxycarbonylation of ethylene^7^ followed by condensation with paraformaldehyde is used to produce methyl methacrylate, which is an important monomer in the polymer industry.

**Scheme 1:**
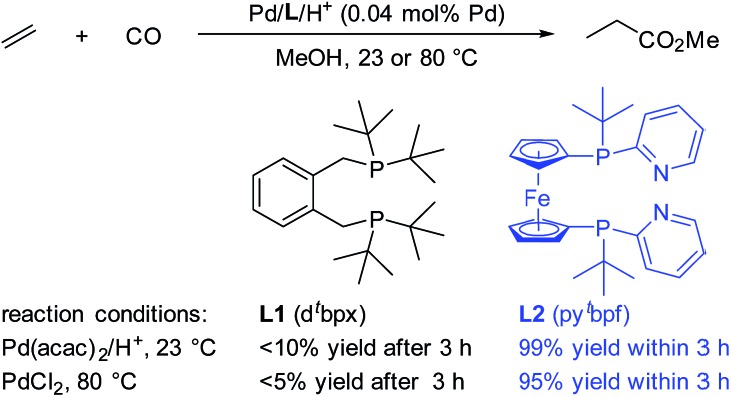
Comparison between **L1** and **L2** in the Pd-catalyzed ethylene methoxycarbonylation. **L2** was used as a mixture of *meso*- and *rac*-isomers.

Interestingly, Drent *et al.* showed the superiority of diphenylphosphinopyridine (Ph_2_P(2-Py)) in the carbonylation of propyne with CO.^8^ Here, the 2-pyridyl moiety in this ligand is suggested to promote the nucleophilic attack of an alcohol on the key palladium acyl species, which is often rate-limiting, *via* metal–ligand cooperativity.^9^ Inspired by this seminal work, very recently we developed highly efficient palladium catalysts for more significant olefin alkoxycarbonylations.^10^ Compared to ligand **L1**, which is currently applied in industry, ligand **L2** shows very high activity even at room temperature (Scheme 1). The yield of the reaction with **L2** is nearly quantitative within 3 hours, while that with **L1** is only less than 10% under the same reaction conditions.10*b* The key feature of **L2** is the combination of amphoteric and sterically hindered moieties on the P atoms. Although the built-in base is thought to be responsible for the enhancement of the activity, until now its exact role has been unclear. Obviously, a better understanding of this substantial effect could be of enormous importance for the rational design of advanced industrial catalysts.

Thus, a detailed investigation into ethylene methoxycarbonylation catalyzed by a palladium/**L2** complex is presented here for the first time. Stoichiometric model reactions, X-ray crystallographic and ESI-mass spectroscopic studies as well as density functional theory computations reveal the importance of metal–ligand cooperativity and provide the basis for understanding the outstanding efficiency of this catalyst, which paves the way for rational catalyst development in the future.

## 2. Results and discussion

### 2.1. Kinetic analysis

To gain insight into the mechanism of the Pd/**L2**–catalyzed methoxycarbonylation, we performed some kinetic experiments under the previously optimized reaction conditions using ethylene as a model substrate due to its industrial relevance. The initial rate of this process was estimated by an online gas drop of ethylene and CO (see ESI† for experimental details). However, it is noted that our reaction was carried out in a closed autoclave under neither isobaric nor isothermal conditions. During the reaction, the total pressure was reduced considerably over time (up to 20 bar) due to the consumption of ethylene and CO, and this can change the partial pressure and the solubility of the gas molecules in solvent. At the same time, the temperature increased up to 7 °C (using 0.0420 mmol of Pd source) over time due to its exothermic properties, and this again can influence the properties of the gas molecules. In addition, it is worth mentioning that ethylene gas does not follow the ideal gas rule,^11^ and this makes such a pressure-dependent reaction more complicated than expected on the basis of the ideal gas models. All of these factors can influence our kinetic model as well as the corresponding kinetic parameters. Thus, our results can only be qualitative rather than quantitative.

**Fig. 1:**
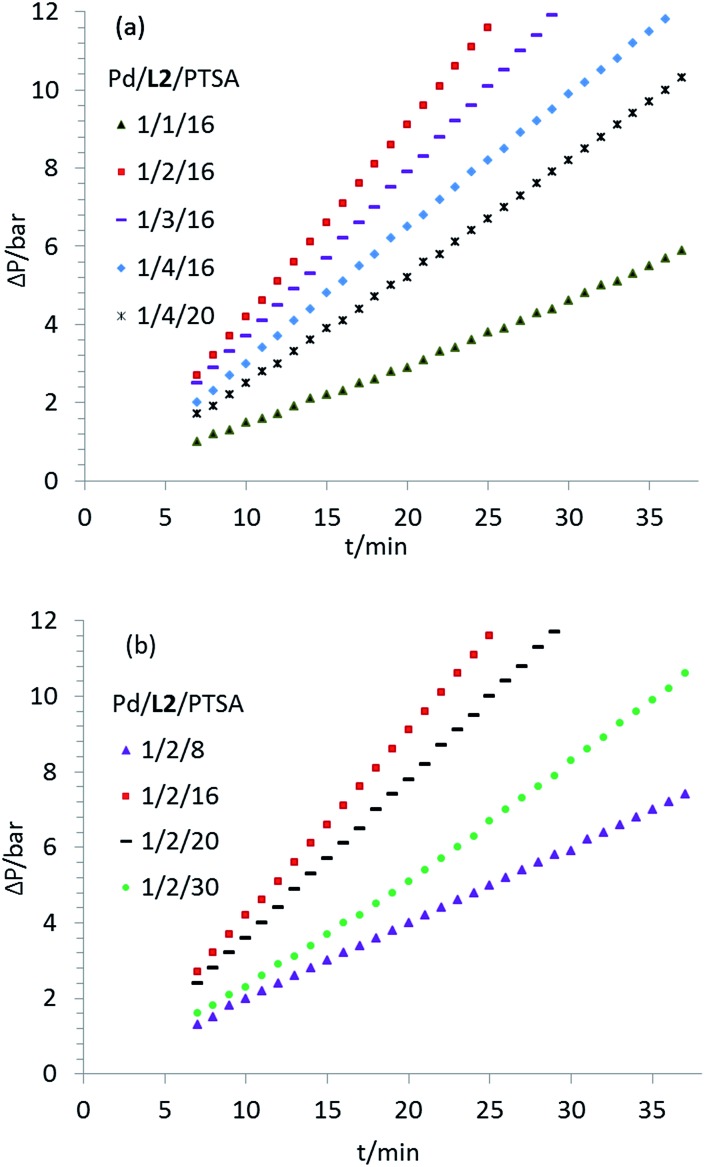
Effect of the amount of (a) ligand **L2** and (b) co-catalytic PTSA on the initial reaction rates. Reaction conditions: [Pd_2_(dba)_3_·CHCl_3_] (11.1 mg, 0.0107 mmol), **L2** (11.1–44.3 mg, 0.0215–0.0858 mmol), PTSA (32.6–121.4 mg, 0.1716–0.6390 mmol), MeOH (20 mL) and ethylene (1.5 g) under CO (30 bar) at 23 °C.

Variation of the Pd(0)- and (ii)-precursors revealed an induction time of almost 3 h using Pd(acac)_2_. Advantageously, the reaction proceeded immediately in the presence of [Pd_2_(dba)_3_·CHCl_3_] (ESI, Fig. S1†), which suggests that the catalytic cycle is triggered by a low valent Pd(0) species. When the ratio of Pd(0)/**L2** changes from 1/1 to 1/2, formation of the active complex is promoted and the initial reaction rate increases substantially, which also indicates the possibility of reversible coordination of the ligand to the metal center (Fig. 1a). However, a further increase in the amount of **L2** inhibited the reaction, probably due to the blocking of free coordination sites. To disprove the notion that the reduced activity is an effect of the acid consumption by the built-in base in **L2**, control experiments with additional amounts of PTSA (*p*-toluenesulfonic acid) were performed (Fig. 1a). The reaction rate increased significantly when the concentration of PTSA increased within a certain range (Pd/**L2**/PTSA from 1/2/8 to 1/2/16, Fig. 1b). Considerably negative effects were observed both at lower and higher concentrations of PTSA.

A nearly zero-order dependence on the concentration of ethylene and CO was observed (ESI, Fig. S4 and S5†), demonstrating their facile incorporation in the corresponding palladium hydride and palladium alkyl complexes. Even at room temperature these elementary steps are not rate-limiting. On the other hand, the reaction has a 1.05-order dependence on the concentration of the palladium catalyst (Fig. S6†), indicating that the active Pd/**L2**/PTSA complex is involved in the rate-limiting step.

Notably, ethylene carbonylation using Pd/**L2**/PTSA proceeded well, even at 0 °C, albeit 24 h was needed to reach full conversion. Gratifyingly, at 100 °C the catalyst loading can be decreased to as low as 0.9 ppm, and the desired product was afforded with very high activity and chemoselectivity (TON: >1 100 000; TOF: 46 000 h^–1^; selectivity: 99%, see ESI† for details). To estimate the kinetic parameters, we plotted the Arrhenius and Eyring equations in the temperature range of 296 to 333 K (Fig. S7†). The estimated activation energy (*E*_a_ = 44.5 kJ mol^–1^) and activation enthalpy (Δ*H*^‡^ = 41.9 kJ mol^–1^) are comparable.

Under otherwise identical conditions, we carried out ethylene methoxycarbonylation in CH_3_OD and found only the mono-deuterated β-isomer product, in contrast with the previously reported labelling results using **L1** at a higher temperature.^12^ To further check the H/D exchange products, we slightly modified our procedure and started the reaction at first using CD_3_OD and ethylene without CO for 3 h, and then further with CO. Finally we found the multi-deuterated α- and β-isomer products (Fig. S12†). This difference might be associated with the ethylene concentration in solution as well as the respective reaction rates.

### 2.2. Characterization of the catalytic intermediates

**Fig. 2:**
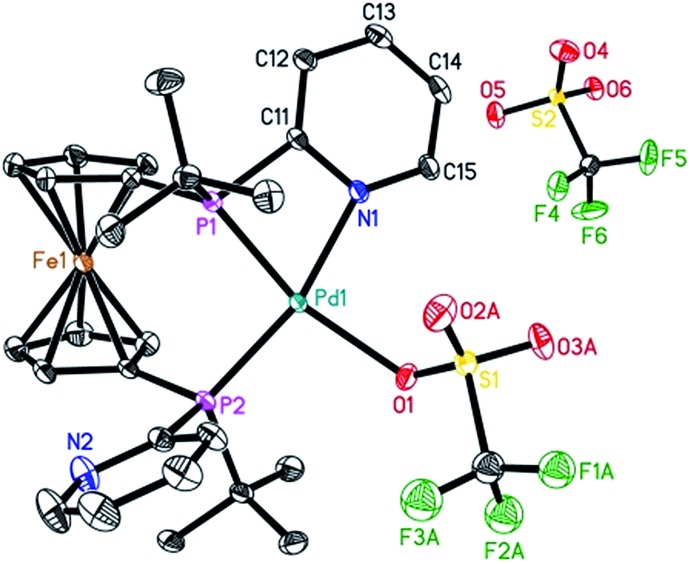
ORTEP view of the palladium complex [Pd(**L2**)(OTf)]OTf with thermal ellipsoids drawn at the 30% probability level. Hydrogen atoms and one part of the disordered coordinated triflate ligand are omitted for clarity. Selected bond lengths and bond angles: N1–Pd1, 2.116(3) Å; P1–Pd1, 2.2363(8) Å; P2–Pd1, 2.2695(8) Å; O1–Pd1, 2.188(2) Å; P1–Pd1–P2, 97.20(3)°; N1–Pd1–P1, 70.07(7)°; N1–Pd1–P2, 163.29(7)°.

To characterize the assumed active palladium hydride species,^13^ [Pd(**L2**)(dba)] was prepared according to the procedure reported for the [Pd(**L1**)(dba)] complexes.^7^ Next, the slow addition of trifluoromethanesulfonic acid (TfOH) into the solution of Pd(**L2**)(dba) in methanol at room temperature should provide the desired active species. Unfortunately, no suitable crystals for X-ray crystallography were obtained. However, in the presence of benzoquinone, the corresponding Pd(ii) complex with **L2** was formed in Et_2_O. Notably, this is the first detailed characterization of an **L2**/Pd(ii) complex. Single crystals of [Pd(**L2**)(OTf)]OTf were obtained in CH_2_Cl_2_/Et_2_O at 0 °C. As shown in Fig. 2, the complex has a distorted square-planar geometry. The palladium atom is coordinated to the two phosphorus centers and one nitrogen atom from one pyridyl ring of **L2**, as well as one oxygen atom from [OTf]^–1^. The bond length of N–Pd is exactly 2.116(3) Å, while those of P–Pd are 2.2363(8) and 2.2695(8) Å, respectively. Bite angles of 70.07(7)° for N1–Pd1–P1 and 97.20(3)° for P1–Pd1–P2 were observed. The crystal structure of [Pd(**L2**)(OTf)]OTf shows that **L2** could also act as a bidentate or a tridentate ligand under different conditions. Actually, **L2** as a bidentate ligand has been observed in the previously reported pre-catalyst Pd(**L2**)(NMM) (NMM, *N*-methylmaleimide).10*b*

Using this novel palladium complex and a catalytic amount of PTSA, the methoxycarbonylation of ethylene gave the desired ester under otherwise identical conditions. In the presence of an additional amount of **L2**, similar activity compared to the *in situ* generated catalyst was observed (Fig. S11†).

Next, we were interested in the structural assignment of intermediates in the catalytic cycle by means of *in situ* spectroscopy. While ^31^P and ^1^H NMR investigations proved unsuccessful, to our delight, electrospray ionization mass spectroscopy (ESI-MS) analysis allowed for the detection of palladium complexes in the crude solution from our standard reaction. NMR control experiments showed the preferential protonation of the pyridyl nitrogen atom compared to the phosphorous atom in **L2**. As shown in Fig. S12,† ESI-MS signals at *m*/*z* = 622.8 and 792.8, with their characteristic isotope distribution, matched with the calculated patterns for [Pd(**L2**)(H)]^+^ and [Pd(**L2**)(OTs)]^+^. However, these results cannot distinguish the location of the proton in the protonated form [Pd(**L2**)(H)]^+^, and DFT computations prefer the protonation of the pyridyl nitrogen atom over the Pd center by 11.3 kJ mol^–1^, indicating a possible equilibrium between the protonation of the pyridyl nitrogen atom and the Pd center. Besides, no double protonation has been found by ESI-MS. In addition, oxidation of the ligand was observed, and the corresponding palladium complexes were also detected (*m*/*z* = 517.0, 533.0 and 549.0, as well as 656.8, 666.8 and 870.8).^14^ In fact, a comparison with the complex of **L1** was carried out under similar conditions, and the protonation of **L1** as well as the cation complex [Pd(**L1**)(H)]^+^ were also detected by ESI-MS.

### 2.3. DFT analysis

To understand the significant difference in activity of the currently used industrial ligand, **L1**, and our ligand, **L2**, we carried out detailed density functional theory computations. In our calculations, we used the real-sized ligands, catalysts (**L1**Pd and **L2**Pd) and substrates (ethylene, CO and CH_3_OH) in the gas phase and with the incorporation of methanol solvation, as well as in methanol solution including van der Waals dispersion correction. All of these computational details and results are given in the ESI.† Since there are plentiful mechanistic investigations into the methoxycarbonylation reaction,7a,^13^,^15^,^16^ we followed the well-accepted catalytic cycle starting from the cationic [**L**PdH]^+^ complex. The first step is olefin coordination and insertion into Pd–H with the formation of the alkyl complex, the second step is CO coordination and insertion with the formation of the acyl complex, and the last step is methanolysis resulting in the formation of the ester and the regeneration of the active [**L**PdH]^+^ catalyst.

On the basis of the computed effective and apparent barriers, we found that **L1**Pd (Table S2†) is less active than **L2**Pd (Table S3†) in the gas phase and in methanol solution, as well as in methanol solution including van der Waals dispersion correction. Inspired by Mecking’s work,^13^,15b we computed the methanolysis step assisted by a three-methanol cluster [Section S4.2(a)†] using **L1** as the ligand. However, no expected energy lowering and stabilizing effects were found, and the single methanol route has a lower effective barrier than the three-methanol assisted route in the gas phase (155.1 *vs.* 160.6 kJ mol^–1^) and in methanol solution (177.2 *vs.* 191.7 kJ mol^–1^). The reason for this artificial effect in Mecking’s work comes from the constrained planar geometry of the three-methanol cluster, which represents a higher order saddle point (three imaginary frequencies), and is 25.1 kJ mol^–1^ higher in energy than the non-planar energy minimum structure calculated in our work. Since the potential energy surfaces in the gas phase and in methanol solution are very similar, and including van der Waals dispersion correction gives a negative apparent free energy barrier (Scheme S3†), only the results including solvation are used for discussion and comparison.

**Fig. 3:**
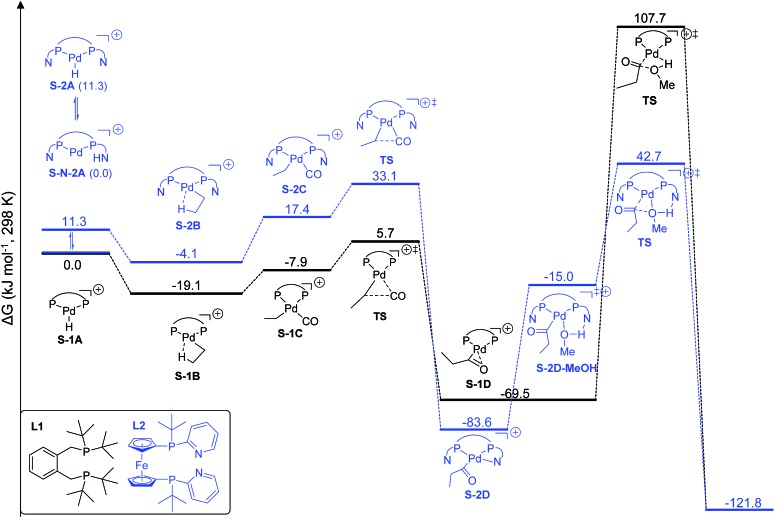
Potential free energy surface using [**L1**Pd–H]^+^ and [**L2**Pd–H]^+^ in ethylene methoxycarbonylation (S denotes solvation).

We found that **L2**Pd is more active than **L1**Pd in both the gas phase and in methanol solution on the basis of the computed effective (between the lowest and highest points) and apparent (between the reference and highest points) barriers. Therefore, we present here only the results in methanol solution to show their differences in stability and activity in the catalytic cycle (Fig. 3).

Starting from the active catalyst, [**L1**Pd–H]^+^, the first step of ethylene insertion into Pd–H is barrierless and the formation of the ethyl complex [**L1**Pd–CH_2_CH_3_]^+^ is exergonic by 19.1 kJ mol^–1^. In the second step, CO coordination to the ethyl intermediate is endergonic by 11.2 kJ mol^–1^ and the effective barrier of CO insertion is 24.8 kJ mol^–1^, and the formation of the acyl complex [**L1**Pd–COCH_2_CH_3_]^+^ is strongly exergonic by 50.4 kJ mol^–1^. The final step of methanolysis has an effective barrier of 177.2 kJ mol^–1^ and is exergonic by 52.3 kJ mol^–1^; the transition state represents the highest point on the potential energy surface and is therefore rate-determining.

For the **L2**Pd catalyst, it is found that the nitrogen atom of the pyridyl ring has a higher proton affinity than the palladium atom by 11.3 kJ mol^–1^, and both protonated forms can have a dynamic equilibrium (99 : 1). Starting from the [**L2**Pd–H]^+^ complex, the formation of the ethyl complex [**L2**Pd–CH_2_CH_3_]^+^ is barrierless and exergonic by 4.1 kJ mol^–1^, revealing the reversibility of this step. In the second step, CO coordination to the ethyl complex is endergonic by 21.5 kJ mol^–1^ and the effective barrier of CO carbonylation is 37.2 kJ mol^–1^, and the formation of the acyl complex is exergonic by 79.5 kJ mol^–1^. Finally, the effective barrier of methanolysis is 126.3 kJ mol^–1^, which is much lower than that using **L1** by 50.9 kJ mol^–1^. This is in qualitative agreement with our experimentally observed activity trend between **L1** and **L2** at room temperature (Scheme 1).

**Fig. 4:**
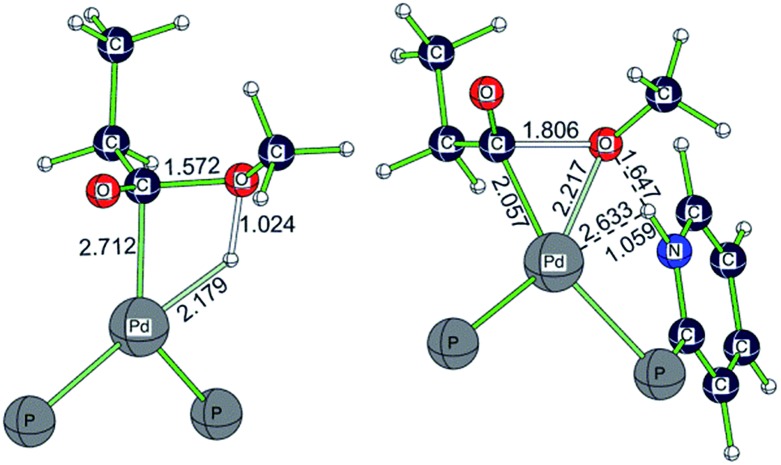
Optimized transition state structures for methanolysis using **L1** and **L2** (only the central part of the structure is shown; other parts are omitted for clarity).

Taking the active catalyst, CO and methanol as a reference, the apparent free energy barrier from using **L2** (42.7 kJ mol^–1^) is lower than that (107.7 kJ mol^–1^) from using **L1** by 65.0 kJ mol^–1^; such a low barrier for **L2** and the remarkable difference between **L1** and **L2** rationalize clearly the observed activity of the **L2** complex. Since the largest difference between **L1**Pd and **L2**Pd is found in the methanolysis step, we analyzed the transition state structures. Detailed structural analysis of the transition state shows that pyridyl N-assisted H–O bond dissociation and O–C bond formation occur when using **L2**; there is no such additional stabilization of the transition state when using **L1** (Fig. 4).

For the transition state with **L1**, methanolysis proceeds through a concerted pathway *via* the formation of the O–C bond (1.572 Å) and Pd–H bond (2.179 Å), as well as the breaking of the O–H bond (1.024 Å) and Pd–C bond (2.712 Å). For the transition state with **L2**, however, the proton from methanol is readily transferred to the N atom of the pyridyl ring (N–H: 1.059 Å), and the transferred proton also interacts with the oxygen atom of the CH_3_O group *via* hydrogen bonding (O–H: 1.647 Å). At the same time, both methoxy and acyl groups are coordinated to the Pd center (C–Pd: 2.057 Å; O–Pd: 2.217 Å), and the critical C–O distance from the nucleophile attack of the negatively charged CH_3_O group to the acyl group is 1.806 Å. This kind of pyridyl N-assisted methanolysis has been reported by Bühl *et al.*, in their work on the methoxycarbonylation of alkynes catalyzed by a Pd complex bearing a chelating 2-pyridyl-diphenylphosphine ligand,^8^,^9^ where the pyridyl N atom can stabilize the transition state *via* N–H interactions.

Comparing the energies of the intermediates and the transition states of the different complexes, it is evident that only the acyl complex and the transition state of the N-assisted methanolysis are lower in energy for ligand **L2**. In particular, the latter step is substantially different to that for **L1**Pd due to the base-assisted activation of the nucleophile (methanol).

In addition, it should be noted that ligand **L2** might prevent the formation of palladium black by the hemi-labile coordination of the pyridine nitrogen atom.

### 2.4. Catalytic cycle

**Fig. 5:**
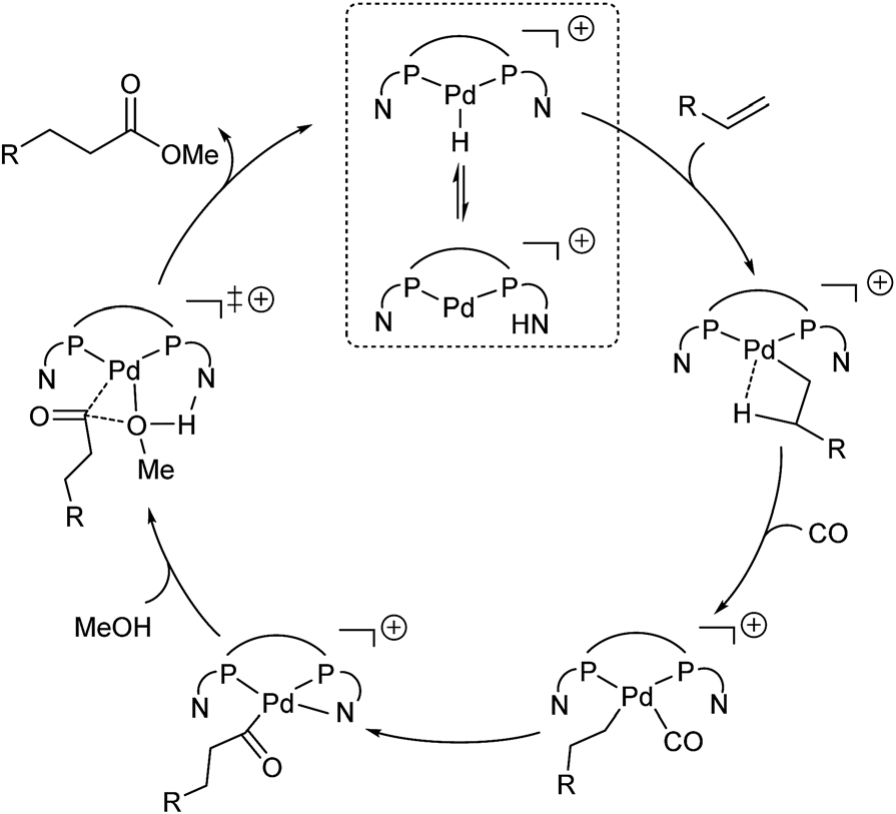
Proposed catalytic cycle for the Pd/**L2**-catalyzed methoxycarbonylation of alkenes.

On the basis of the experiments *vide supra* and the computed potential energy surface, we propose the following catalytic cycle for the **L2**Pd-catalyzed methoxycarbonylation of alkenes (Fig. 5). The first step is the protonation of the complex, and the proton is in equilibrium between the N atom of the pyridyl ring on the phosphorus ligand and the Pd center. Subsequently, the barrierless and exergonic insertion of the alkene into the Pd–H bond proceeds to give the corresponding alkyl complex. The third step is CO coordination and insertion with the formation of the acyl species. Finally, N-assisted methanolysis takes place, which is energetically favorable for **L2**Pd.

### 2.5. Extension of alkene methoxycarbonylation

So far, palladium-catalyzed alkoxycarbonylations using **L2** have only been reported for ethylene, propene and butenes. To demonstrate the superiority of this ligand with a built-in base, the methoxycarbonylation of various alkenes, including sterically hindered and functionalized substrates, was investigated. As shown in Table 1, aside from tetramethylethylene **1a**, the sterically hindered alkene **1b** (an industrial mixture of C8 alkenes, known as “diisobutene”, from the dimerization of isobutene) was converted into a single ester, **2b**, in quantitative yield. Similarly, cyclic substrates including cyclohexene, cyclooctene and indene proved to be suitable substrates, giving the corresponding esters **2c–e** in high yields. The excellent TON of 55 000 for the methoxycarbonylation of **1c** demonstrated the outstanding efficiency of the catalyst, albeit in lower yield. When terminal and internal olefins were employed using ligand **L2**, the corresponding products **2f–i** were afforded in high yields, although the regioselectivity was somewhat lower compared to previous data using **L1**.^7^ This is explained by the accelerated reaction of the nucleophile with the internal acyl complex. 1,1-disubstituted double bonds were carbonylated selectively into the desired esters **2j–m** in very high yields. Interestingly, diester **2m** is a promising monomer for polymerization. Gratifyingly, our catalyst also allowed the alkoxycarbonylation of functionalized olefins **1n** and **1o** with ester and cyano groups, which afforded the ester with high yields and selectivity. Even the carbonylation of the less reactive push–pull substituted unsaturated bond of **1p** proceeded well, and product **2p** was obtained in 70% yield as a single isomer.

**Table 1:** Pd-catalyzed carbonylation of various alkenes with ferrocenyl ligand **L2**^*a*^

Alkene	Ester	Yield/%, *n*/iso
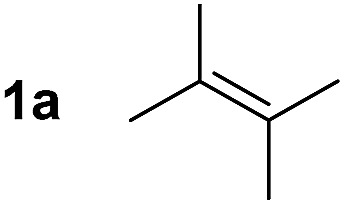	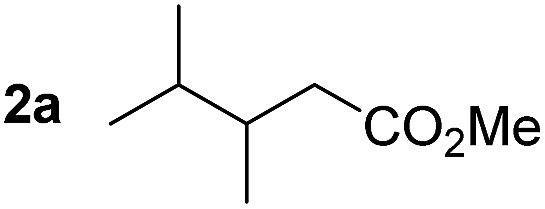	99^*b*^, 99/1
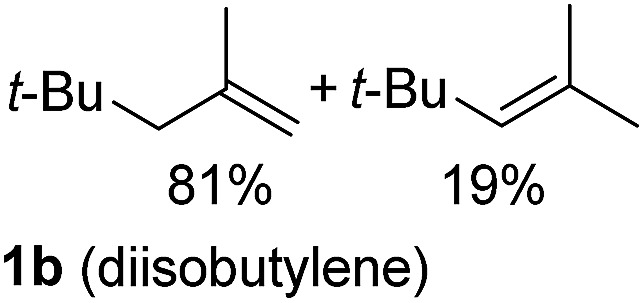	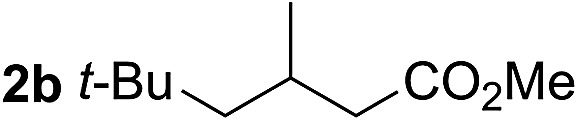	99, 99/1
		48^*c*^, 99/1
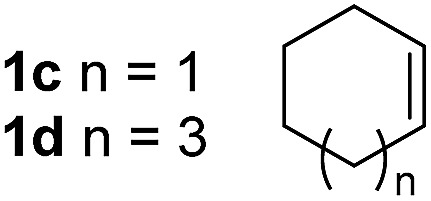	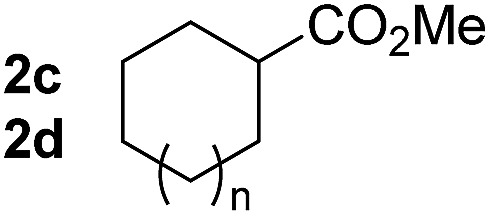	99^*b*^ (55^*b*^ ^,^^*d*^)
		98
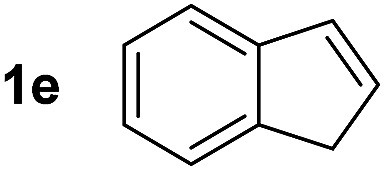	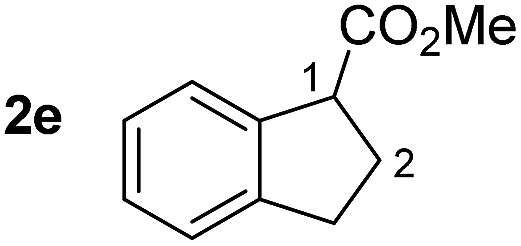	70
		1/2 = 74/26
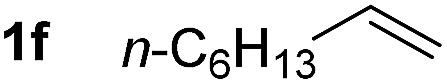	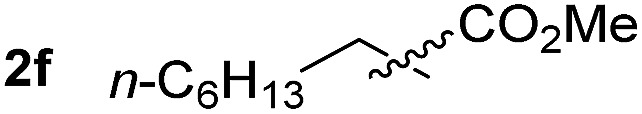	99^*b*^, 75/25
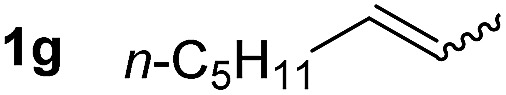		99^*b*^, 72/28
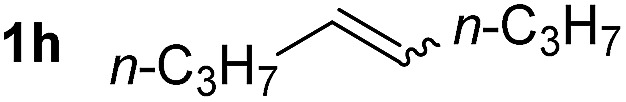		99^*b*^, 71/29
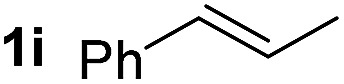	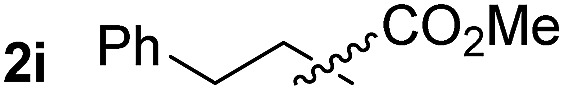	92, 79/21
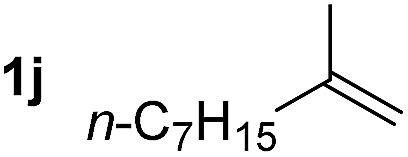	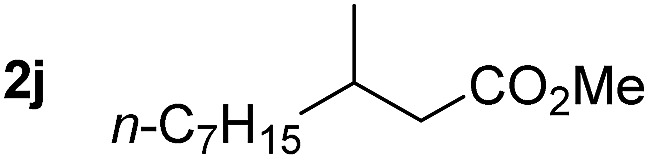	99, 99/1
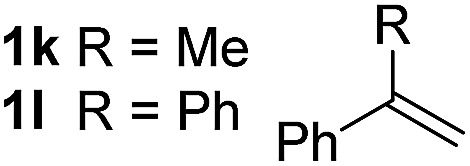	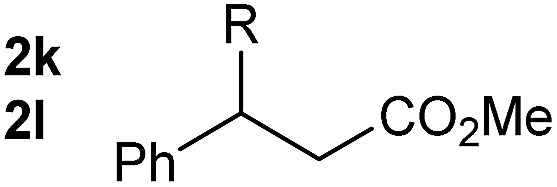	98, 99/1
		98, 99/1
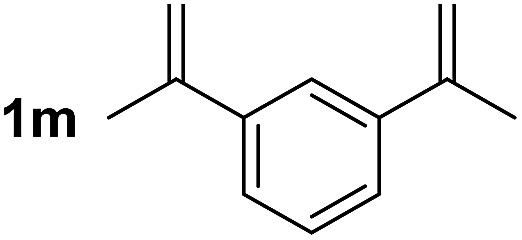	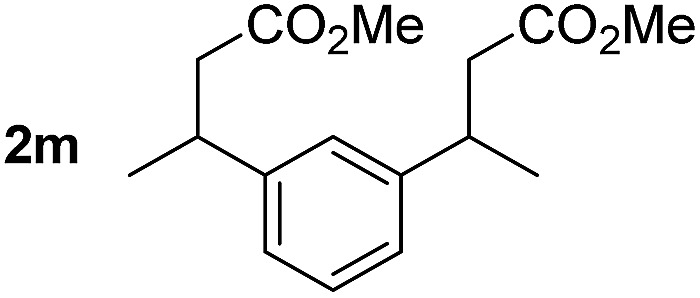	99, 99/1
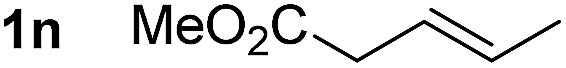	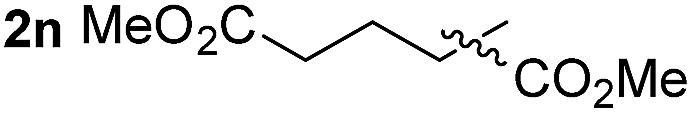	99^*b*^, 77/23
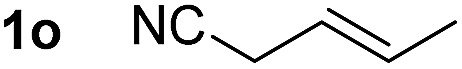	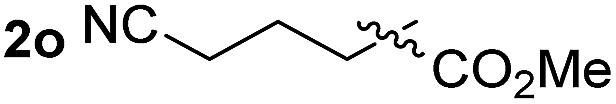	98, 80/20
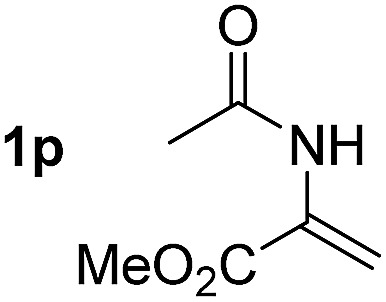	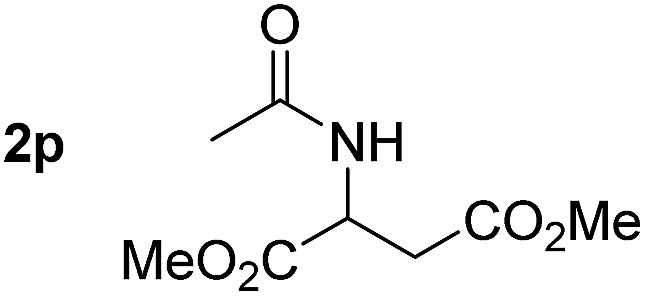	70, 99/1

^*a*^Reaction conditions: alkene **1** (2.0 mmol) and Pd(acac)_2_/**L2/**PTSA (0.2/0.8/3.2 mol%) in MeOH (2 mL) under CO (40 bar) at 120 °C for 20 h. Isolated yields are shown.

^*b*^Yields were detected by GC analysis using isooctane as the internal standard.

^*c*^ **1b** (40 mmol) and Pd(acac)_2_/**L2**/PTSA (0.005/0.1/0.4 mol%) in MeOH (20 mL). TON of 9600.

^*d*^ **1c** (80 mmol) and Pd(acac)_2_/**L2**/PTSA (0.001/0.5/0.2 mol%) in MeOH (20 mL) for 72 h. TON of 55 000.

## 3. Conclusions

The mechanism of the palladium-catalyzed alkoxycarbonylation of olefins using the state-of-the-art ligand **L2** has been elucidated for the first time. More specifically, the multifunctional roles of the 2-pyridyl moiety in **L2** are shown. On one hand, this integrated base acts as a proton shuttle for the formation of the palladium hydride and the rate-determining N-assisted methanolysis. On the other hand, the nitrogen atom is able to improve the durability of the catalyst *via* hemilabile coordination to the palladium center in the catalytic cycle. Experimental and DFT computational studies support the metal–ligand cooperativity in these alkene carbonylation reactions. In this respect, we believe this work will stimulate the more rational design of advanced catalysts for carbonylations and other reactions involving (de)protonation steps.

Crystal structures have been deposited at the Cambridge Crystallographic Data Centre and allocated the deposition number CCDC 1554504 ([Pd(**L2**)(OTf)]OTf).

## Conflicts of interest

There are no conflicts to declare.

## Supplementary Material

Supplementary information
