# Supplementary material for: Cooperative catalytic methoxycarbonylation of alkenes: uncovering the role of palladium complexes with hemilabile ligands
Source: Chem Sci. 2018 Feb 7;9(9):2510–6. doi: 10.1039/c7sc02964k (PMC5912013; doi:10.1039/c7sc02964k)
Supplement: Supplementary file 1 [file SC-009-C7SC02964K-s001.pdf]

Electronic Supplementary Material (ESI) for Chemical Science.
This journal is © The Royal Society of Chemistry 2018

Supporting Information

Cooperative Catalytic Methoxycarbonylation of Alkenes: Uncovering the Role of Palladium Complexes with Hemilabile Ligands

Table of Contents

1. General information 2

2. Kinetic analysis on Pd/L2-catalyzed methoxycarbonylation of ethylene 3

3. Characterization of catalytic intermediates 15

4. DFT caculation 18

5. Alkoxycarbonylation of various alkenes with ferrocenyl phosphine ligands 60

6. References 62

7. Spectra of the carbonylation products (Figure S12) 62

1. General information

Air- and moisture-sensitive reaction were performed under argon atmosphere. Chemicals were purchased from Aldrich, TCI, Alfa, Acros, or Strem. Unless otherwise noted, all commercial reagents were used without further purification.

The isolated esters were characterized by ^{1}H NMR and ^{13}C NMR spectroscopy, which were recorded on Bruker Avance 300 (300 MHz) NMR spectrometers. Chemical shifts δ (ppm) are given relative to solvent: references for $CDCl_3$ were 7.26 ppm (^{1}H-NMR) and 77.16 ppm (^{13}C-NMR). ^{13}C-NMR spectra were acquired on a broad band decoupled mode. Multiplets were assigned as s (singlet), d (doublet), t (triplet), dd (doublet of doublet), m (multiplet) and br (broad singlet). ESI (electrospray ionization) mass spectrum was recorded on an Agilent Technologies 6210 TOF LC/MS using methanol (100%) as eluent. GC analysis was performed on a Agilent 7890A chromatograph with a a 29 m HP5 column.

Data were collected on a Bruker Kappa APEX II Duo diffractometer. The structures were solved by direct methods (SHELXS-97: Sheldrick, G. M. *Acta Crystallogr.* **2008**, *A64*, 112.) and refined by full-matrix least-squares procedures on F^2 (SHELXL-2014: G. M. Sheldrick, *Acta Crystallogr.* **2015**, *C71*, 3.). XP (Bruker AXS) was used for graphical representations.

2. Kinetic analysis on Pd/L2-catalyzed methoxycarbonylation of ethylene

The comparision between ligands **L2** and **L1** in the palladium catalyzed methoxycarbonylation of ethylene at room temperature was studies previously (*Angew. Chem. Int. Ed.*, **2017**, 56, 5267-5271):

(a) reaction conditions: $Pd(acac)_2$ (6.5 mg, 0.04 mol%), **L1** or **L2** (0.16 mol%), PTSA (61 mg, 0.6 mol%), CO (30 bar), MeOH (20 mL), ethylene (1.5 g, 53.6 mmol), 23 ^{o}C.

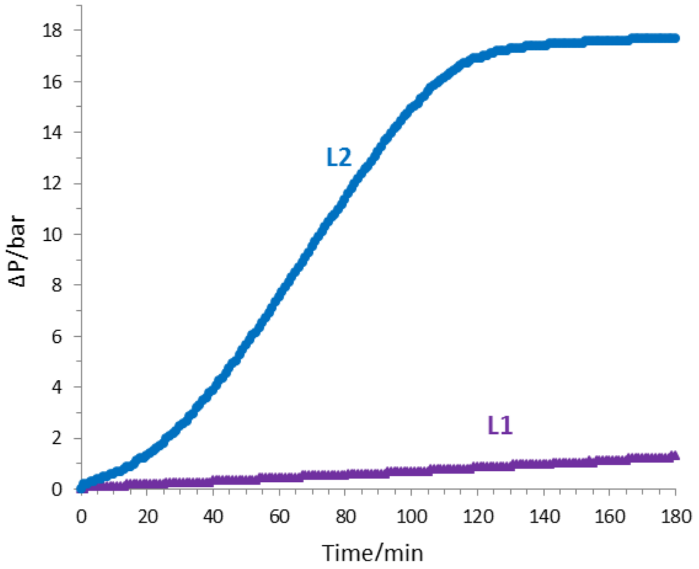

(b) Reaction conditions: $PdCl_2$ (2.53 mg, 0.04 mol%), **L1** or **L2** (0.16 mol%), MeOH (5 mL), ethylene (1.0 g, 35.7 mmol), CO (30 bar), 80 ^{o}C.

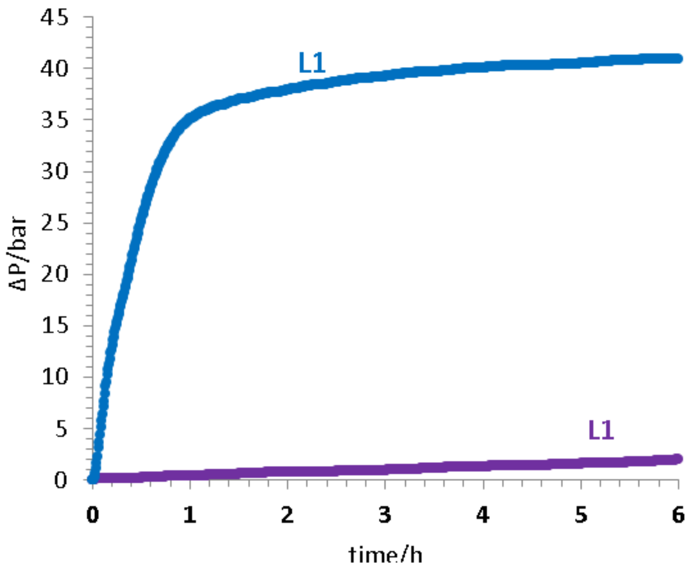

General procedure: In these studies, the concentration of ethylene was calculated by gas consumption of CO and ethylene, which was monitored on line automatically. Under argon atmosphere, a 100 mL steel autoclave was charged with $[Pd_2(dba)_3{\cdot}CHCl_3]$, ligand **L2**, and *p*-toluenesulfonic acid (PTSA). Methanol (20 mL) was injected into the autoclave by syringe. Then ethylene was introduced into the autoclave (mass control by balance). When the reaction mixture was reached to the desired temperature, CO was introduced into the autoclave and the reaction was carried out at this temperature. After the reaction was completed, the autoclave was cooled to room temperature and depressurized slowly. The content was transferred to a 50 mL Schlenk flask and isooctane (internal standard) was added into the solution. The yield and chemo-selectivity was measured by GC analysis.

2.1. Effect of palladium precursors. Reaction conditions: [$Pd_2(dba)_3 \cdot CHCl_3$] (11.1 mg, 0.0107 mmol, 0.0214 mmol Pd) or $Pd(acac)_2$ (6.5 mg, 0.0214 mmol), **L2** (44.3 mg, 0.0858 mmol), PTSA (65.2 mg, 0.3432 mmol), MeOH (20 mL), ethylene (1.5 g), CO (30 bar), 23 °C.

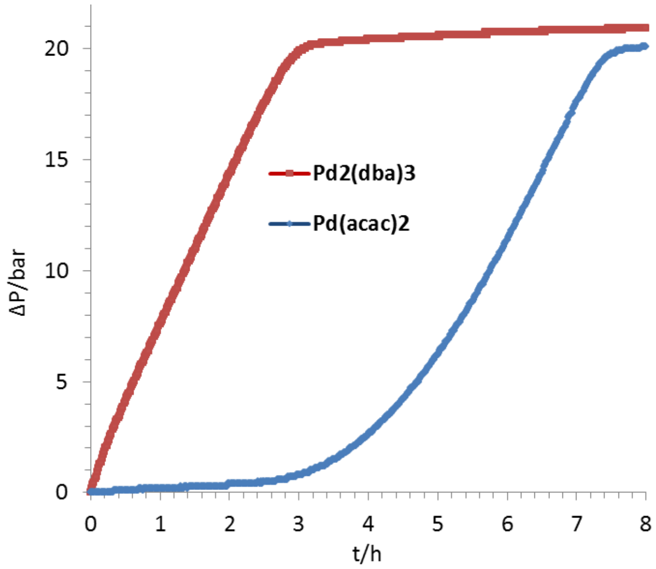

Figure S1. Effect of palladium precursors on ethylene methoxycarbonylation.

2.2. Effect of the amount of ligand L2. Reaction conditions: [$Pd_2(dba)_3 \cdot CHCl_3$] (11.1 mg, 0.0107 mmol, 0.0214 mmol Pd), **L2** (11.1-44.3 mg, 0.0215-0.0858 mmol), PTSA (65.2 mg, 0.3432 mmol), MeOH (20 mL), ethylene (1.5 g), CO (30 bar), 23 °C.

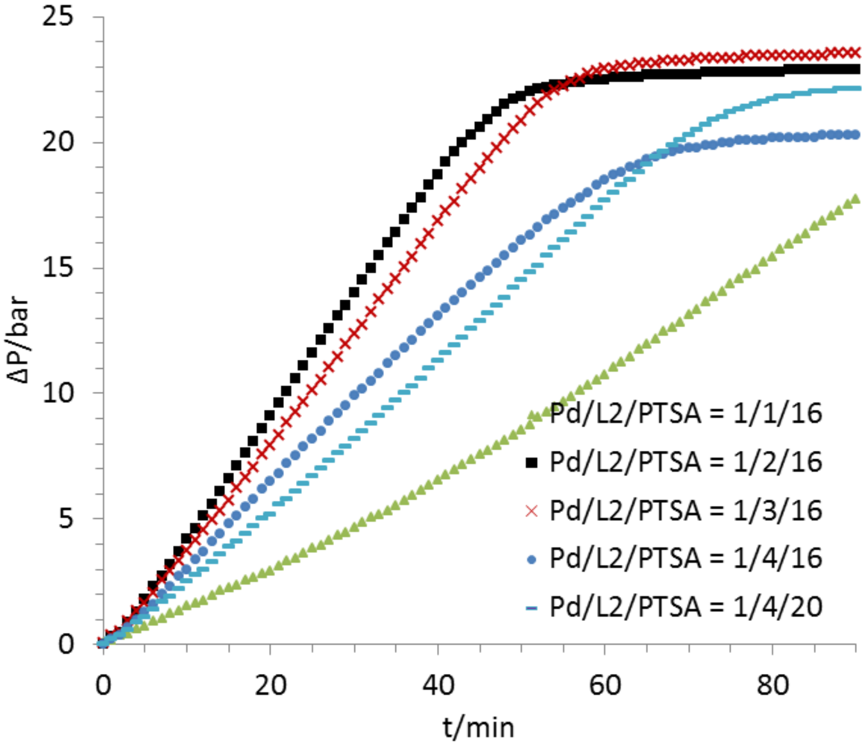

Figure S2. Gas consumption of CO and ethylene versus reaction time: effect of ligand **L2** concentration.

2.3. Effect of the acid concentration. Reaction conditions: $[Pd_2(dba)_3 \cdot CHCl_3]$ (11.1 mg, 0.0107 mmol, 0.0214 mmol Pd), **L2** (22.2 mg, 0.0430 mmol), PTSA (32.6-121.4 mg, 0.1716-0.6390 mmol), MeOH (20 mL), ethylene (1.5 g), CO (30 bar), 23 ^{o}C.

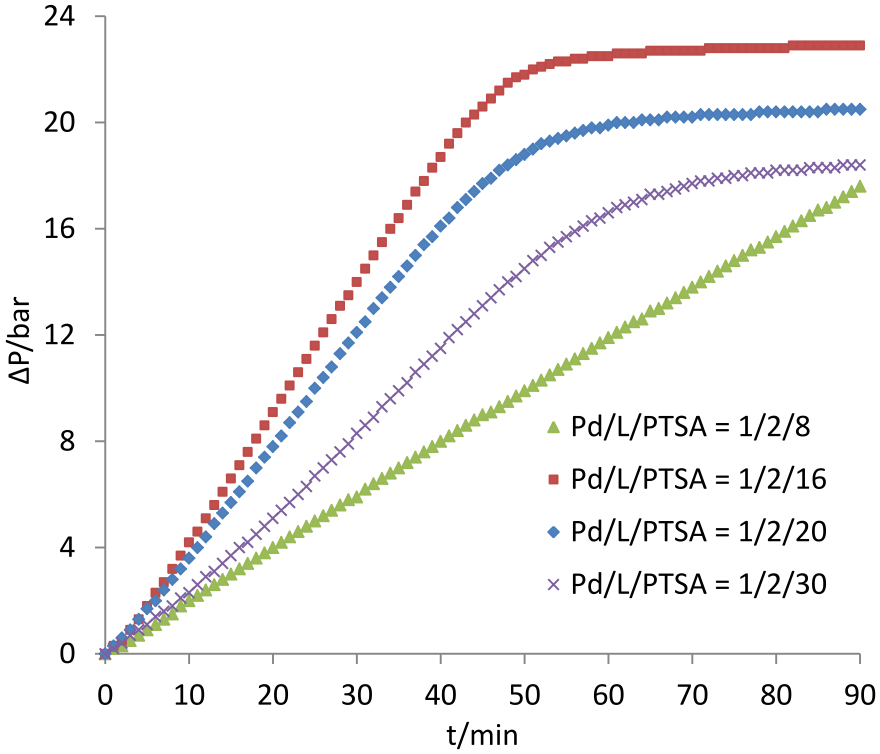

Figure S3. Gas consumption of CO and ethylene versus reaction time: effect of the amount of PTSA.

2.4. Effect of the amount of ethylene. Reaction conditions: $[Pd_2(dba)_3 \cdot CHCl_3]$ (11.1 mg, 0.0107 mmol, 0.0214 mmol Pd), **L2** (22.2 mg, 0.0430 mmol), PTSA (65.2 mg, 0.3432 mmol), MeOH (20 mL), ethylene (1-2.5 g, 35.7-89.3 mmol), CO (30 bar), 23 ^{o}C.

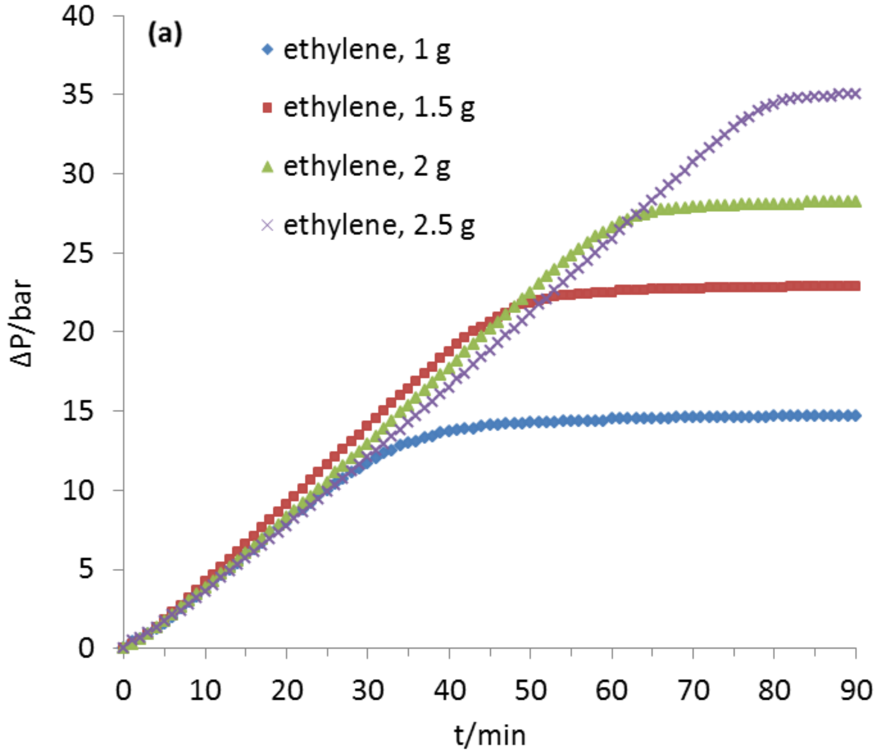

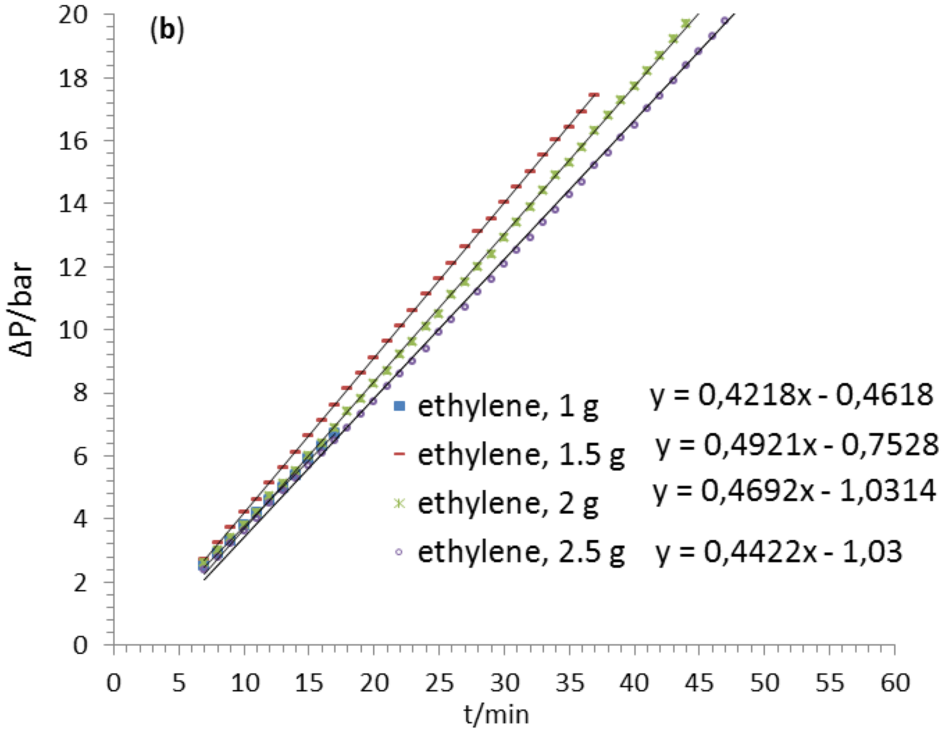

Figure S4. Gas consumption of CO and ethylene versus reaction time: effect of the amount of ethylene

2.5. Effect of the pressure of CO. Reaction conditions: [$Pd_2(dba)_3 \cdot CHCl_3$] (11.1 mg, 0.0107 mmol, 0.0214 mmol Pd), **L2** (22.2 mg, 0.0430 mmol), PTSA (65.2 mg, 0.3432 mmol), MeOH (20 mL), ethylene (1.5 g, 53.57 mmol), CO (10-42 bar, 1.3-5.1 g, 46.43-182.14 mmol), 23 ^{o}C.

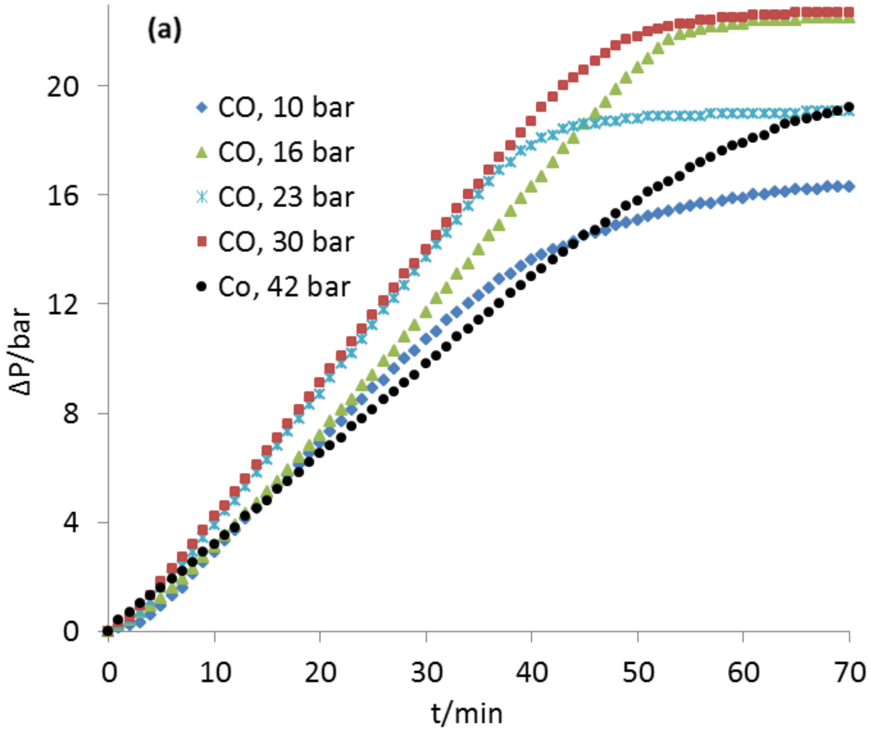

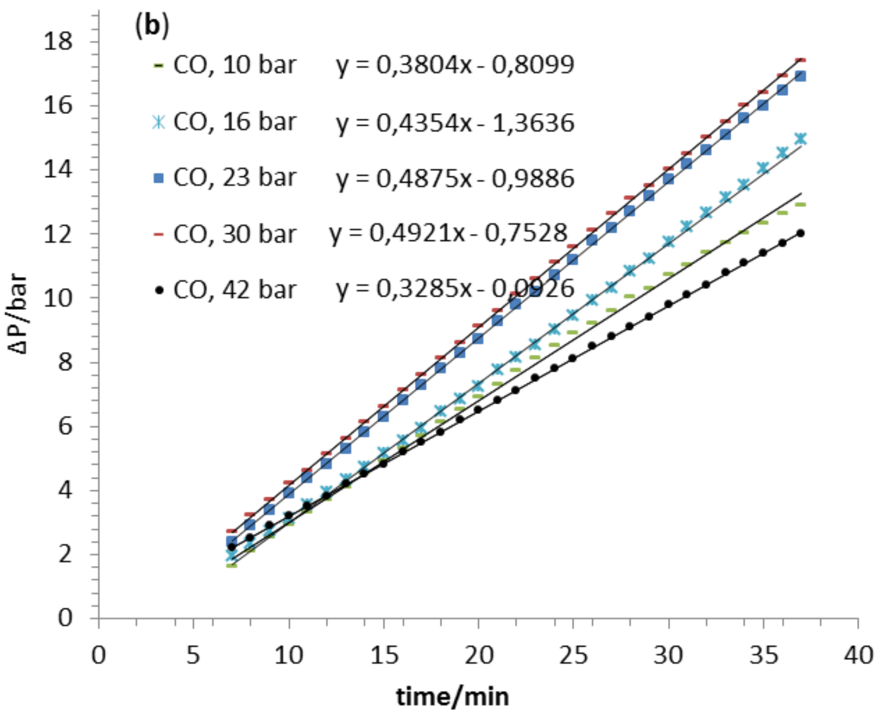

Figure S5. Gas consumption of CO and ethylene versus reaction time: effect of the amount of CO.

2.6. Effect of the Palladium catalyst concentration. The reactions were carried out under a constant large excess of ligand **L2** and of PTSA conditions (Pd/**L2**/PTSA: from 1/4/16 to 0.125/4/16): [$Pd_2(dba)_3$] (11.45-1.43 mg, 0.00313-0.025 mmol Pd), **L2** (51.6 mg, 0.1 mmol), PTSA (76 mg, 0.4 mmol), MeOH (20 mL), ethylene (2.0 g, 71.43 mmol), CO (30 bar), 23 °C.

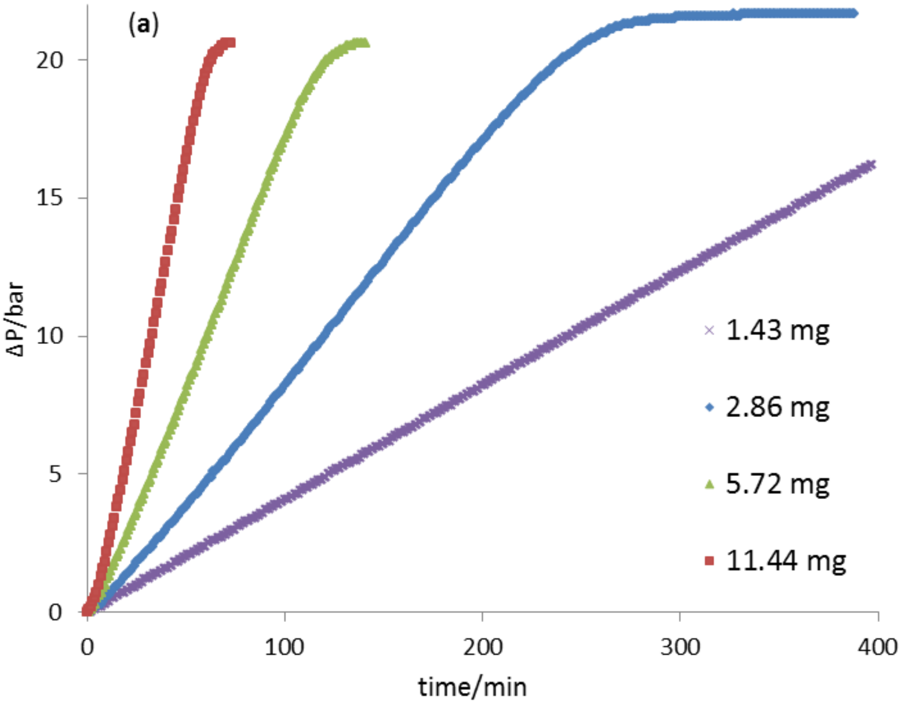

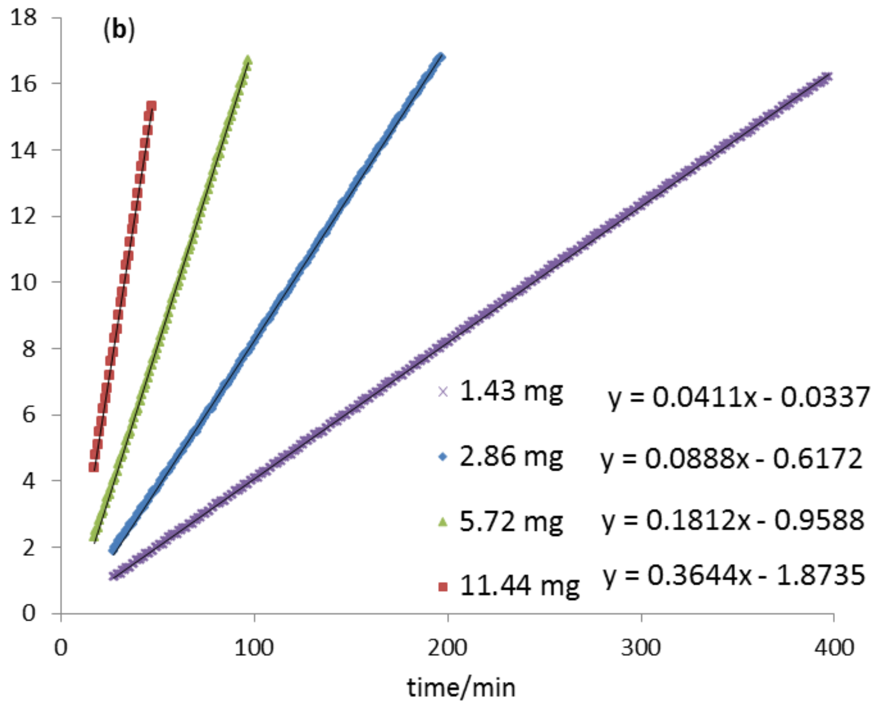

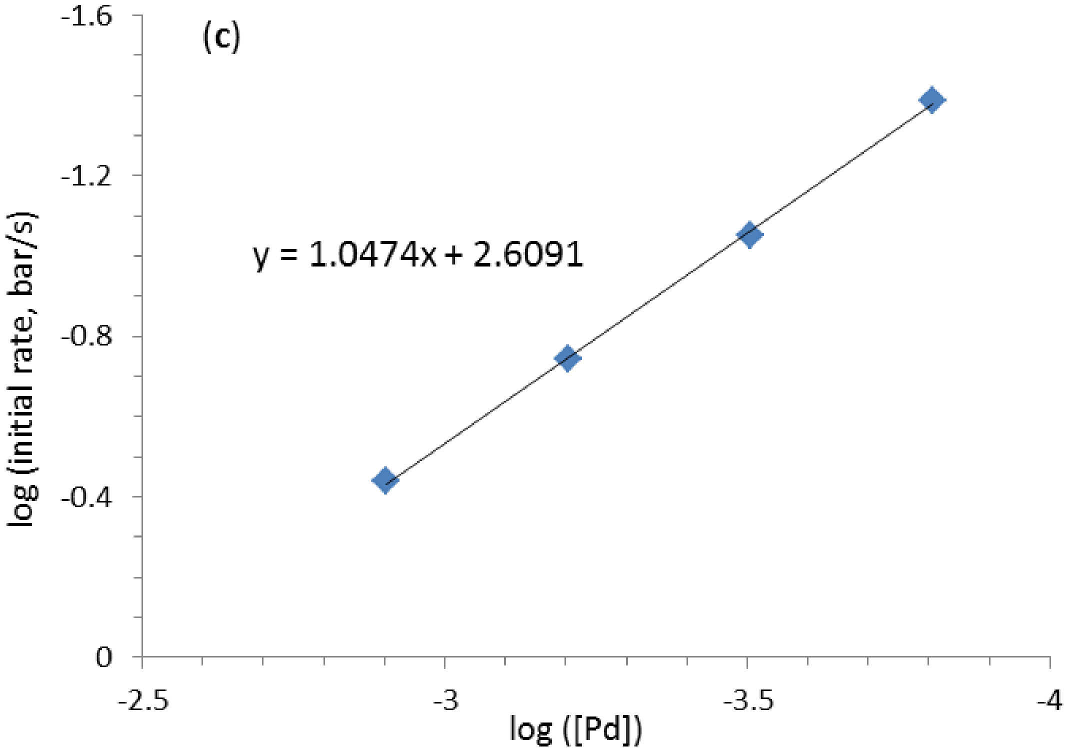

Figure S6. Effect of the palladium catalyst concentration: (a) gas consumption of CO and ethylene, (b) calculation of initial reaction rate and (c) plot of log (initial rate) versus log (the palladium catalyst concentration).

2.7. Ethylene methoxycarbonylation at various reaction temperature: [$Pd_2(dba)_3 \cdot CHCl_3$] (11.1 mg, 0.0107 mmol, 0.0214 mmol Pd), **L2** (22.2 mg, 0.0430 mmol), PTSA (65.2 mg, 0.3432 mmol), MeOH (20 mL), ethylene (2.0 g, 71.43 mmol), CO (30 bar), 0-80 °C.

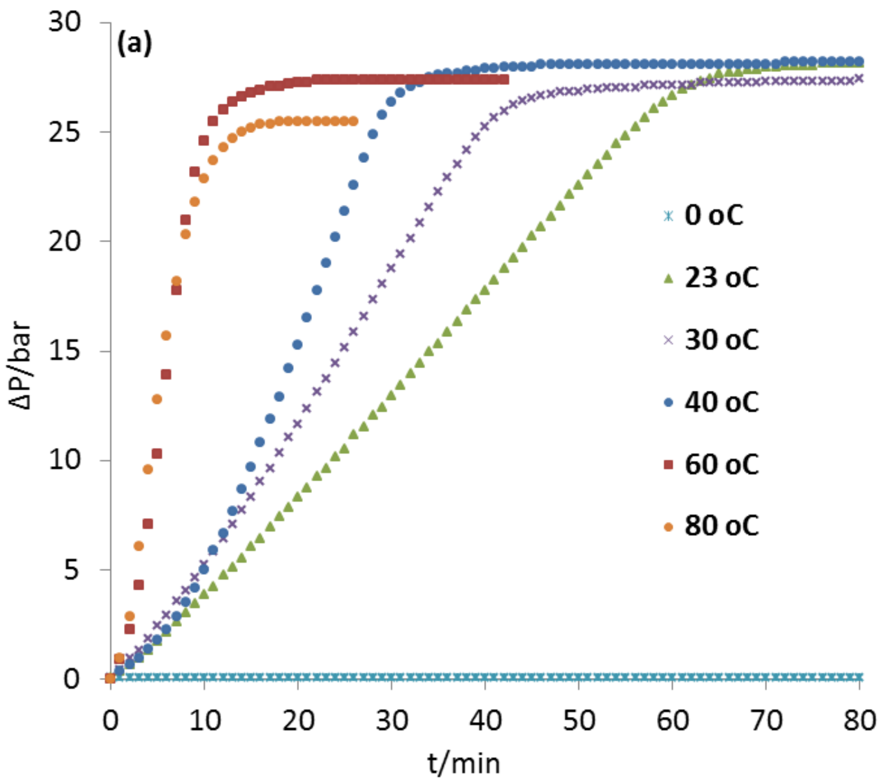

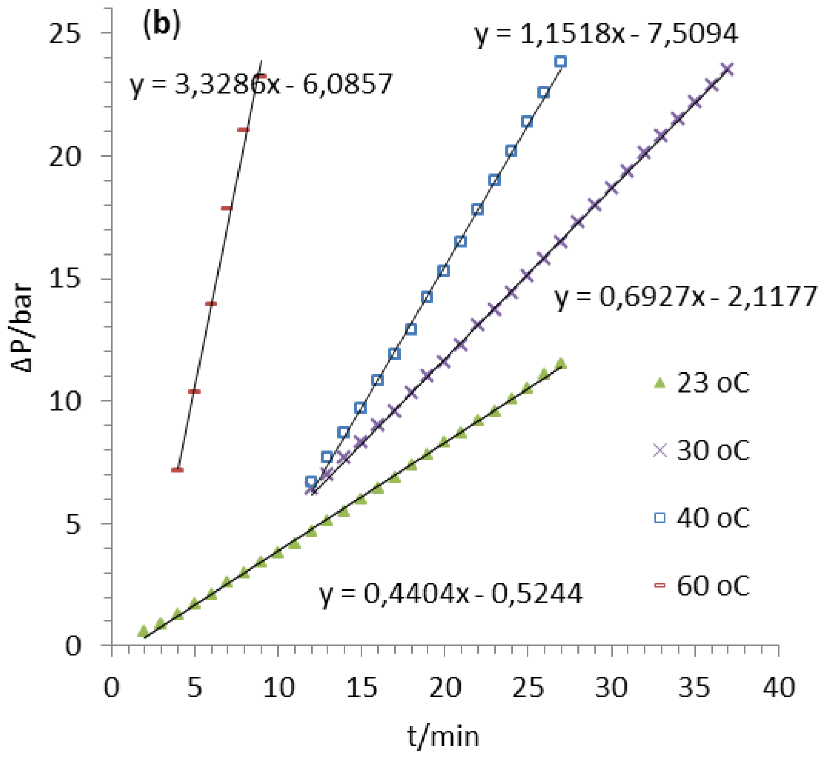

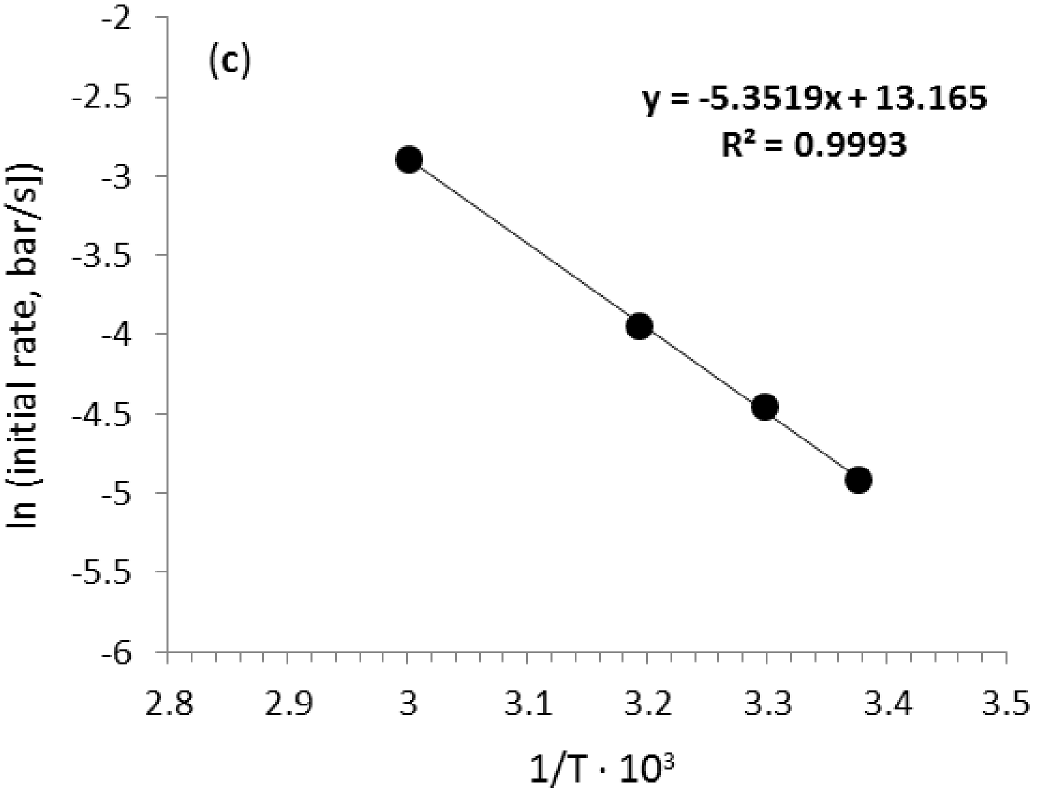

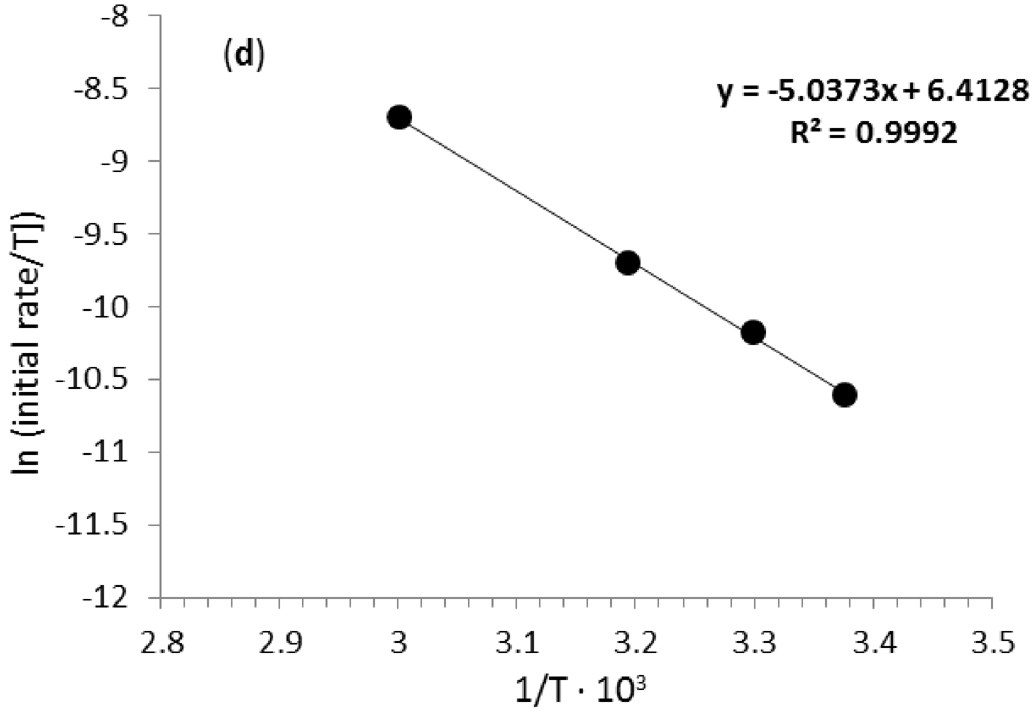

Figure S7. Pd/**L2**-catalyzed ethylene methoxycarbonylation at various reaction temperature: (a) Gas consumption of CO and ethylene, (b) calculation of the initial rate, (c) Arrhenius plot and (d) Eyring plot.

a). Calculation of apparent activation energy and derivation of Arrhenius equation

According the reaction rate,

$$r = k[ethylene]^a[CO]^b[MeOH]^c[cat.]^d$$

and a = 0, b = 0, d = 1,
So initial reaction rates,

$$r = k[cat.]^1[MeOH]^c$$

According Arrhenius equation,

$$k = Ae^{-\frac{E_a}{RT}}$$

Initial reaction rates,

$$r = Ae^{-\frac{E_a}{RT}}[cat.]^1[MeOH]^c$$

So,

$$\ln r = -\frac{E_a}{RT} + \ln A + \ln([cat]\,[MeOH]^c)$$

So, E_a = 44.5 kJ/mol.

b). Calculation of Gibbs free energy, enthalpy, entropy, and derivation of Eyring equation

According Eyring equation ,

$$k = \frac{k_B T}{h} e^{-\frac{\Delta G^{\neq}}{RT}}$$

And

$$r = k[cat.]^1[MeOH]^c$$

So,

$$r = \frac{k_B T}{h} e^{-\frac{\Delta G^{\neq}}{RT}}[cat.]^1[MeOH]^c$$

Gibbs free energy equation,

$$\Delta G^{\neq} = \Delta H^{\neq} - T\Delta S^{\neq}$$

Then,

$$\ln\frac{r}{T} = -\frac{\Delta H^{\neq}}{RT} + \frac{\Delta S}{R} + \ln\frac{k_B}{h} + \ln([cat][MeOH]^c)$$

So,

$$\Delta H^{\neq} = 41.9\ kJ/mol$$

Table S1. Effect of reaction temperature (T) on initial rates (*r*).

T/K	1/T, 1/K	r, bar/min	ln (r, bar/s)	r/K	Ln (r/K)
296.15	3.376667229	0.4404	-4.914416436	2.47847E-05	-10.60528252
303.15	3.298697015	0.6972	-4.461502836	3.80835E-05	-10.17573057
313.15	3.193357816	1.1518	-3.953018626	6.13018E-05	-9.699700935
333.15	3.001650908	3.3286	-2.891792767	0.000166522	-8.700385606

2.8. Pd/L2-catalyzed ethylene methoxycarbonylation at 100 °C: Under argon atmosphere, a 300 mL steel autoclave was charged with [$Pd_2(dba)_3 \cdot CHCl_3$] (0.6 mg, 0.00058 mmol, 0.00116 mmol Pd), **L2** (258 mg, 0.5 mmol), and PTSA (760 mg, 4 mmol). Methanol (100 mL) was injected into the autoclave via syringe. The weight of the autoclave was determined. Then ethylene (12 g, circa, 357 mmol) was introduced into the autoclave (mass control by balance). After CO (30 bar) was introduced into the autoclave, the reaction mixture was carried out at 100 °C for 6 h. Significant gas consumption was observed. The autoclave was cooled to room temperature and depressurized slowly. Then the same amount of ethylene and CO was introduced into the autoclave again and the reaction was carried out at 100 °C for 6 h. After 4 times, we weighted the autoclave and total 74 g mass addition of the autoclave was detected by balance, which corresponded to 116 g of the desired product. The product methyl propionate was confirmed by GC analysis (>99% selectivity). Total TON (turnover number): 1,139,000, TOF for 24 h: 47,000 h^{-1}.

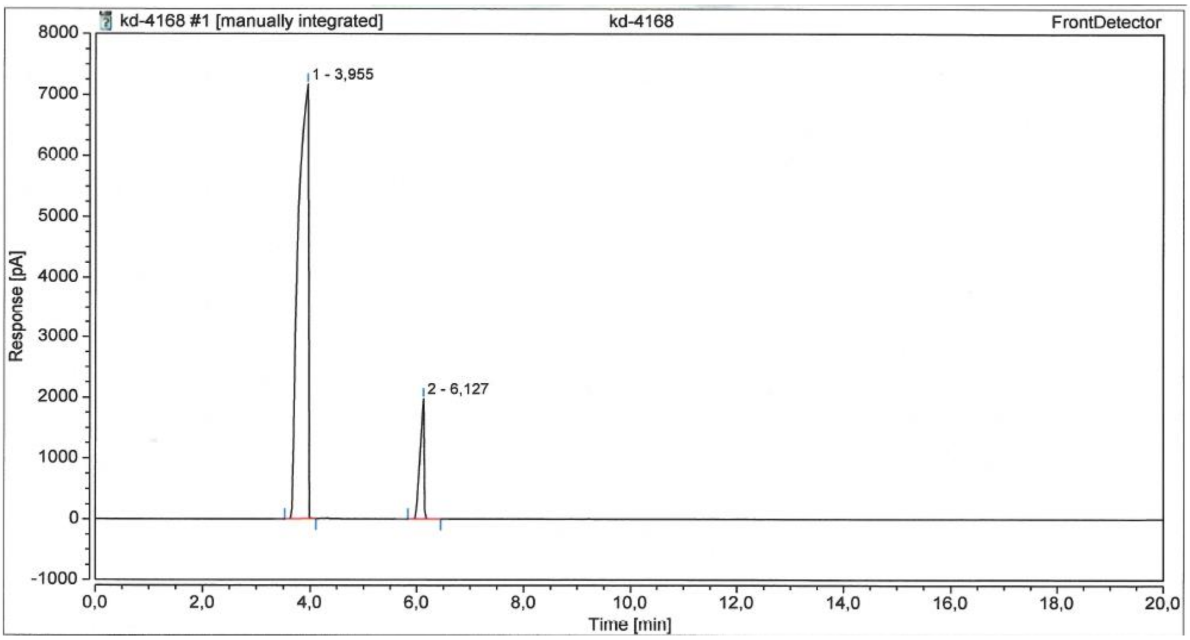

Figure S8. GC spectrum for Pd-catalyzed methoxycarbonylation of ethylene

2.9. Kinetic isotope effect on ethylene methoxycarbonylation with Pd/L2: Reaction conditions: [$Pd_2(dba)_3 \cdot CHCl_3$] (11.1 mg, 0.0107 mmol, 0.0214 mmol Pd), **L2** (22.2 mg, 0.0430 mmol), PTSA (65.2 mg, 0.3432 mmol), MeOH or CH_3OD (20 mL), ethylene (2 g, 71.43 mmol), CO (30 bar), 23 °C.

To further check the H/D exchange products, we slightly modified our procedure and started the reaction at first using CD_3OD and ethylene without CO for 3 h; and then further with CO. Finally we found multi-deuterated α- and β-isomer products (Figure S12). Reaction conditions: [$Pd_2(dba)_3$] (9.82 mg, 0.0107 mmol, 0.0214 mmol Pd), **L2** (22.2 mg, 0.0430 mmol), PTSA (65.2 mg, 0.3432 mmol), CD_3OD (20 mL), ethylene (2 g, 71.43 mmol), CO (30 bar), 23 °C.

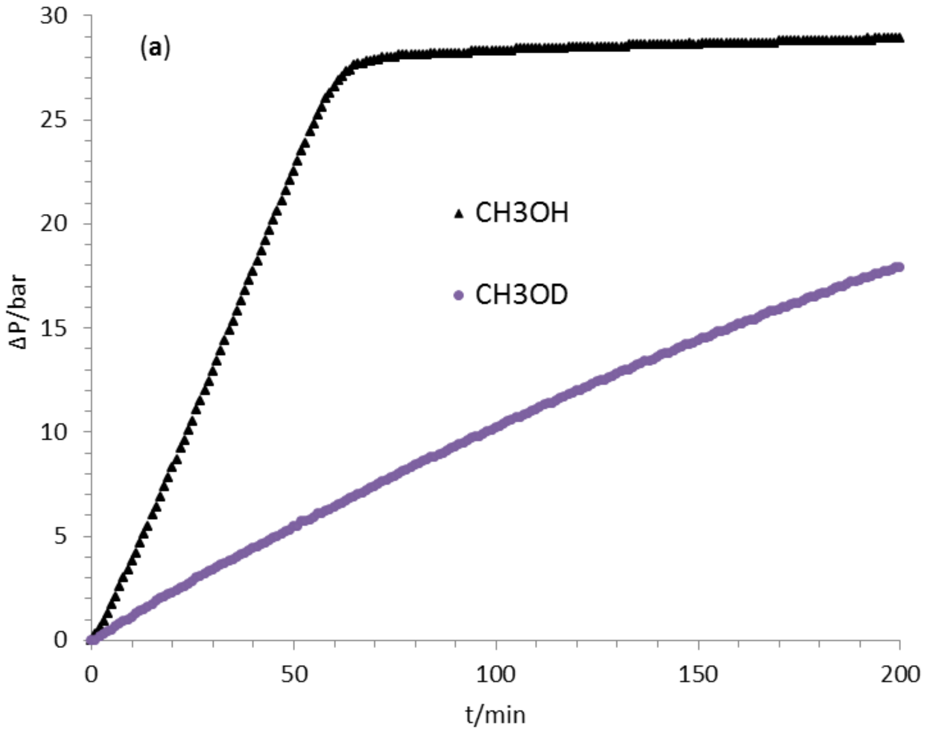

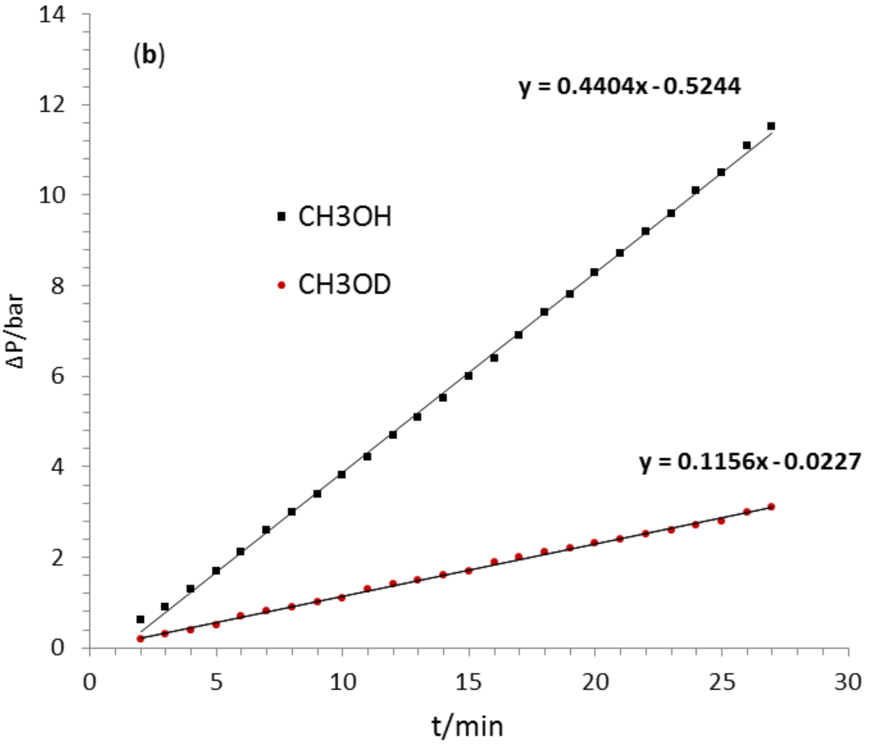

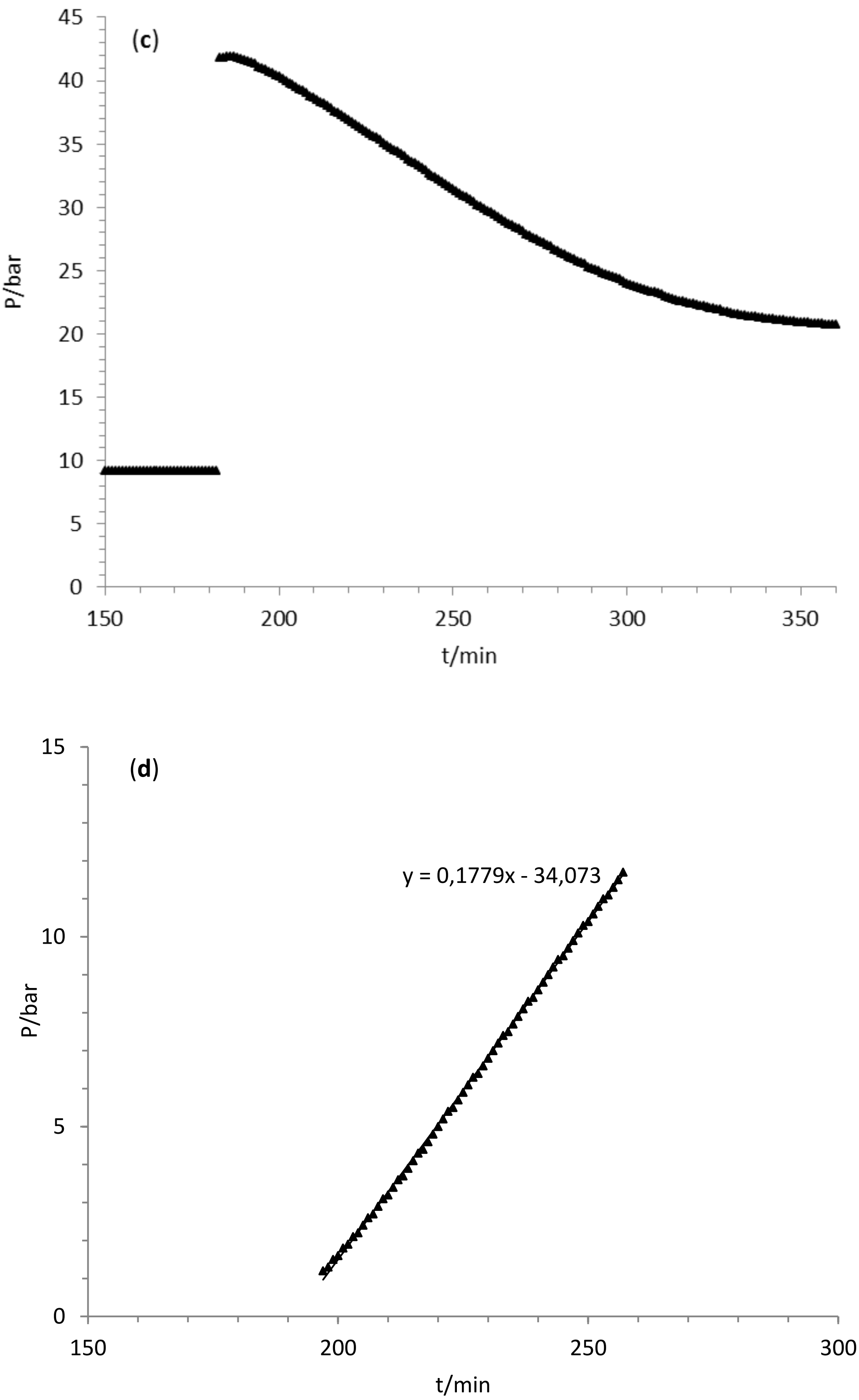

Figure S9. Kinetic isotope effect: (a) Gas consumption of CO and ethylene in CH_3OH or CH_3OD, (b) calculation of the initial rate in CH_3OH or CH_3O, (c) gas pressure of the reaction system versus time under modified conditions, (d) calculation of the initial rate under modified conditions.

3. Characterization of catalytic intermediates

3.1. Methoxycarbonylation of ethylene catalyzed by [Pd(L2)(OTf)]OTf: Reaction conditions: [Pd(**L2**)(OTf)]OTf (19.75 mg, 0.0214 mmol), **L2** (11.1 mg, 0.0215 mmol), PTSA (65.2 mg, 0.3432 mmol), MeOH (20 mL), ethylene (1.5 g, 53.57 mmol), CO (30 bar), 23 ^{o}C.

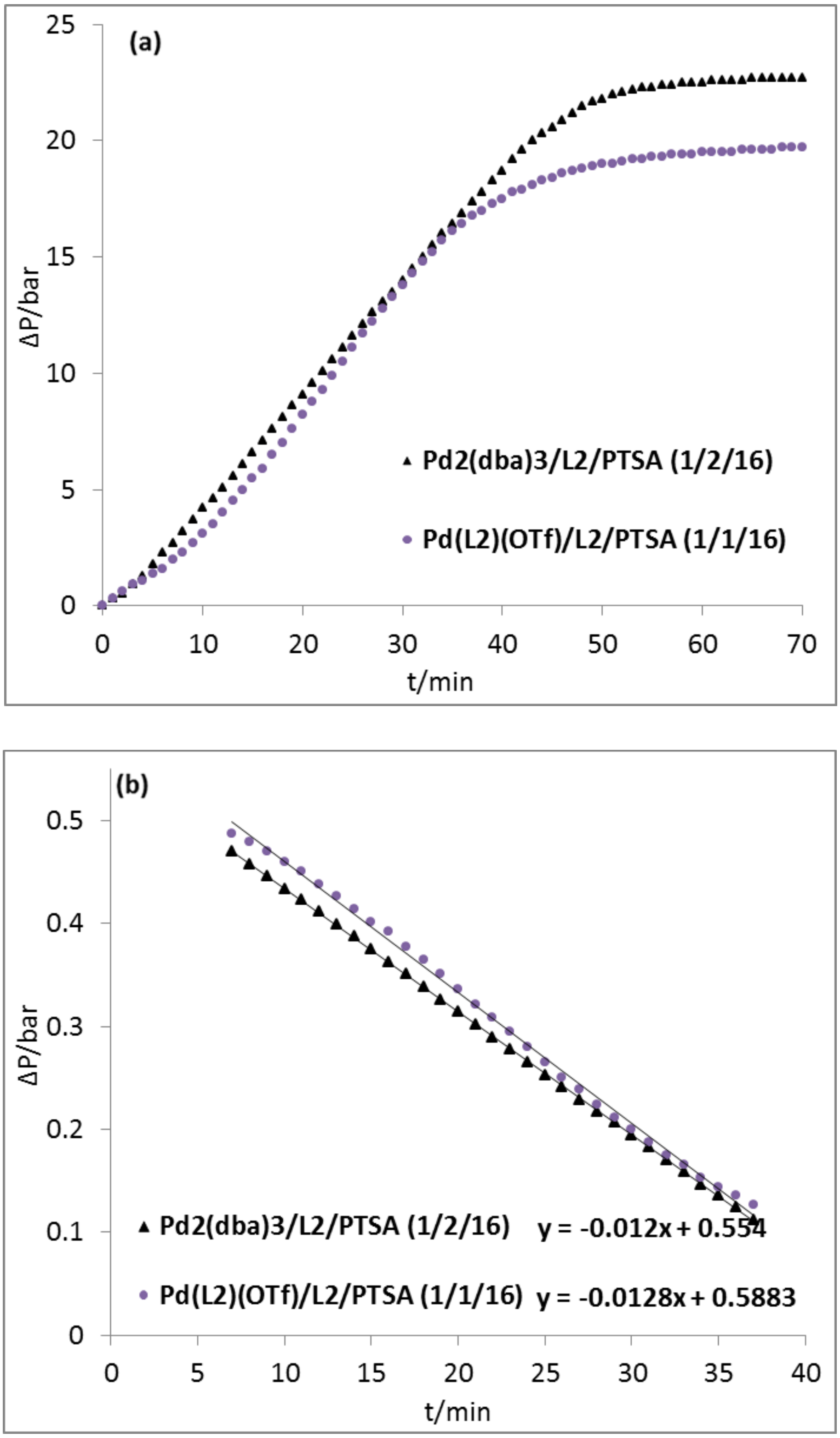

Figure S10. Methoxycarbonylation of ethylene catalyzed by [Pd(**L2**)(OTf)]OTf: (a) gas consumption of CO and ethylene, (b) ethylene concentration versus reaction time.

3.2. ESI-mass spectroscopy analysis

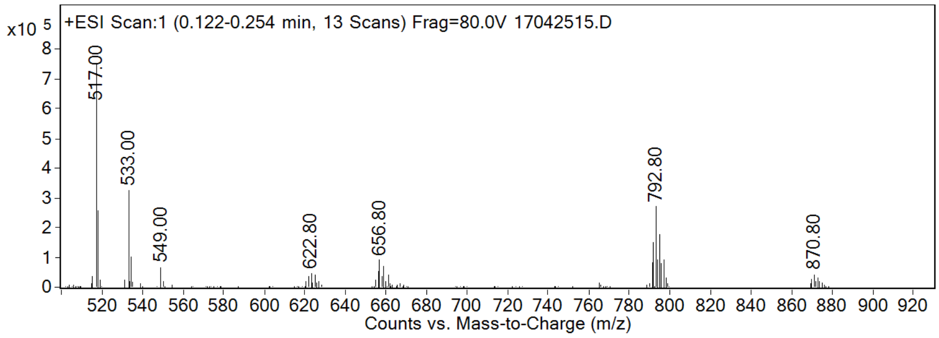

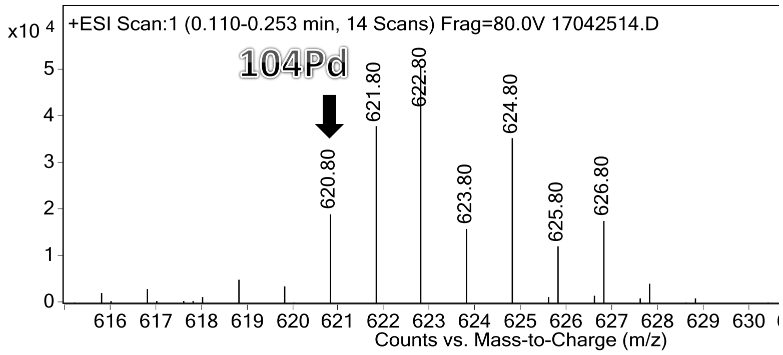

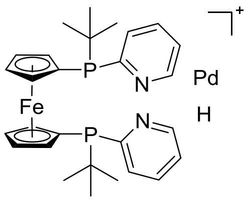

Exact Mass: 623,06542
Molecular Weight: 623,81398
m/z: 623.06542 (100.0%), 625.06583 (96.8%), 622.06702 (81.7%), 627.06709 (42.9%),

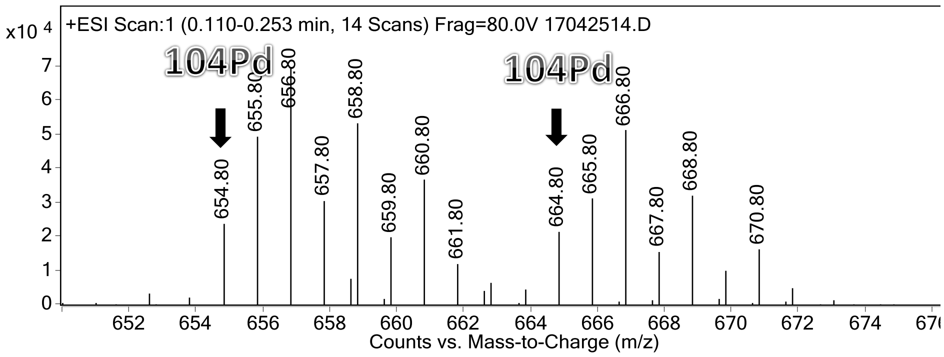

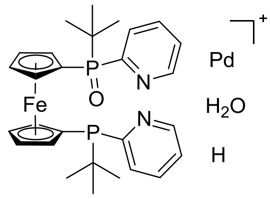

Exact Mass: 657,07090
Molecular Weight: 657,82798
m/z: 657.07090 (100.0%), 659.07131 (96.8%), 656.07250 (81.7%), 661.07257

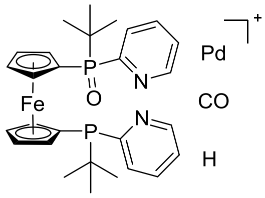

Exact Mass: 667,05525
Molecular Weight: 667,82298
m/z: 667.05525 (100.0%), 669.05566 (96.8%), 666.05685 (81.7%), 671.05692 (42.9%)

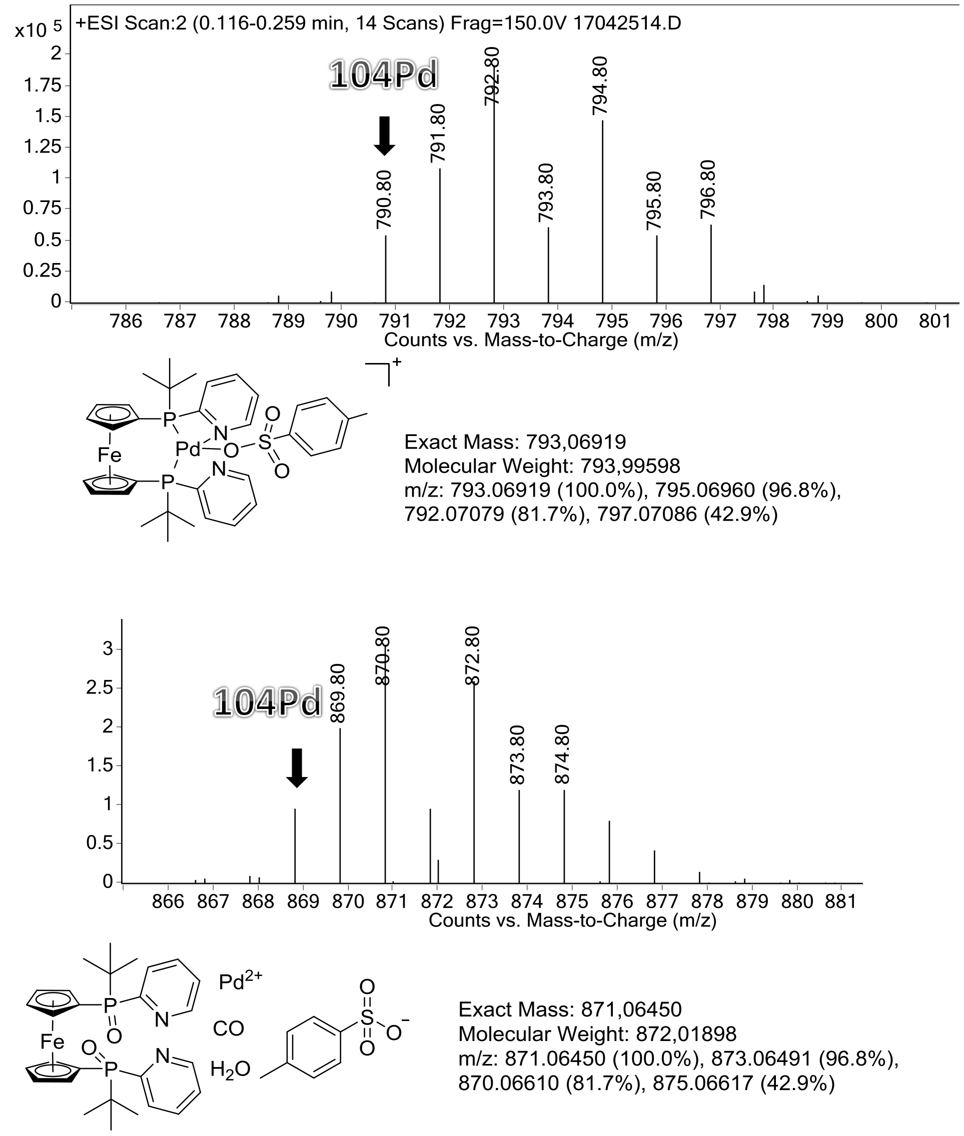

Figure S11. ESI-MS analysis of the crude reaction solution for detection of Pd complexes.

4. DFT caculation

4.1: Computational methods and models

All calculations were carried out by using the Gaussian 09 program.[1] All structures were optimized at the B3PW91[2] level of density functional theory (DFT) with the TZVP[3] basis set (LANL2DZ effective core potential for Pd and Fe[4]). All optimized structures were characterized either as energy minimums without imaginary frequencies or transition states with only one imaginary frequency by frequency calculations; and the imaginary model connects the initial and the final states. The thermal correction to Gibbs free energy at 298 K from frequency analysis was added to the total electronic energy, and the corrected Gibbs free energy (ΔG) at 298 K was used for discussion and comparison. We also carried out self-consistent reaction field (SCRF) structure optimization and frequency calculation at the B3PW91 level using the solvation model based on solute electron density (SMD)[5] and methanol as solvent to estimate the solvation effect (B3PW91-SCRF). To testing the effect of van der Waals dispersion correction, we computed the B3PW91-GD3BJ-SCRF/LANL2DZ(Pd)/TZVP/SMD(MeOH) single-point energies of the full potential energy surface using the GD3BJ paremeter with the B3PW91-SCRF/LANL2DZ(Pd)/TZVP/SMD(MeOH) geometries. In all our calculations, we used the real-size model systems without constrains and simplifications for the ligands, *o*-$C_6H_4(CH_2P_tBu_2)_2$ (**L1**) and ferrocenyl($P_tBuPy)_2$ (**L2**), as well as their corresponding Pd complexes, **L1**Pd and **L2**Pd.

There are reports about the mechanism of alkoxycarbonylation of olefin (R-CH=CH_2) catalyzed by Pd complexes (Scheme S1).[6] Starting from the catioinc $[LPd\text{-}H]^+$ complex, the first step is olefin coordination and Pd-H insertion with the formation of the alkyl complex $[LPd(\text{-}CH_2CH_2R)]^+$; and the second step is CO coordination and insertion with the formation of the acyl complex $[LPd(\text{-}CO\text{-}R')]^+$; and the last step is methanolysis resulting the formation of ester and the regeneration of the active $[LPd\text{-}H]^+$ catalyst. In our study, we followed this mechanism for **L1**Pd and **L2**Pd. At first, we presented the results from gas phase calculations and then those under the consideration of solvation with methanol as solvent as well as those in methanol solution including van der Waals dispersion correction. Finally comparison between **L1**Pd and **L2**Pd has been made.

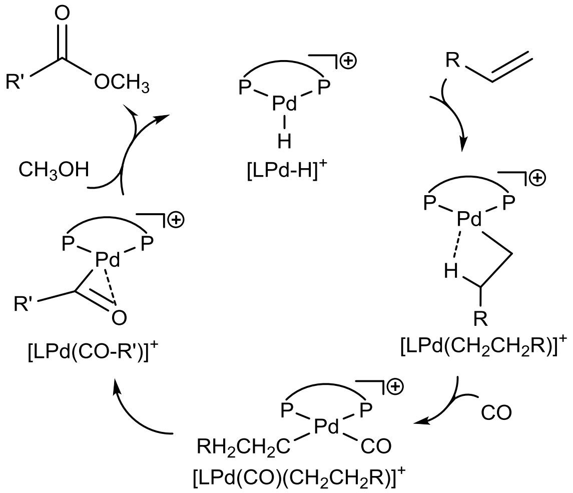

Scheme S1. Well-accepted mechanism of Pd-catalyzed methoxylcarbonylation of olefins

4.2: Computational results

(a) Reaction with L1Pd complex in gas phase

For **L1**Pd, the classic mechanism starting with $[\mathbf{L1}Pd\text{-}H]^+$ species (**1A**) for ethylene methoxycarbonylation in gas phase was computed at first. The potential energy surfaces is shown in Scheme S2. Surprisingly, no stable intermediate of side-on coordinated ethene could be located and all attempts to optimize such structures resulted in the

spontaneous formation of ethyl complex via β-H agostic interaction, [**L1**Pd(CH_2CH_3)]$^+$ (**1B**); and this is probably due to the rather small size of ethylene, which can free rotate in the side-on coordination. The formation of **1B** is therefore barrier-less and exergonic by 27.1 kJ/mol.

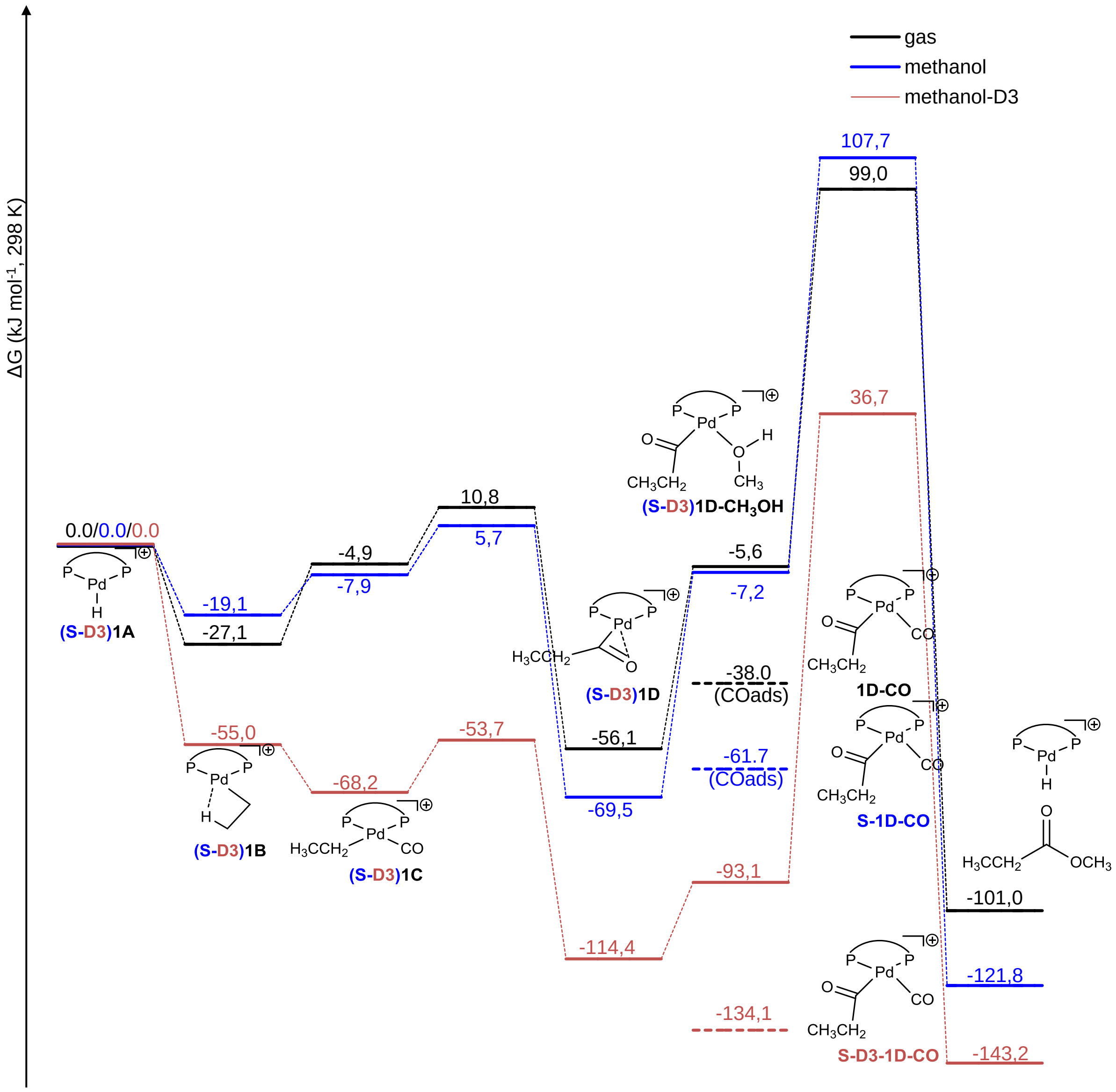

Scheme S2. Potential energy surface by using [**L1**Pd-H]$^+$ in ethene methoxycarbonylation in gas phase (black line), in methanol solution (blue line with **S**) and in methanol solution including van der Waals dispersion correction (bark-red line with **D3**)

On the basis of the ethyl complex **1B**, the next step is CO coordination to form complex [**L1**Pd(CO)(-CH_2CH_3)]$^+$ (**1C**), followed by CO insertion resulting in the formation of acyl complex, [**L1**Pd(-CO-CH_2CH_3)]$^+$ (**1D**). Starting from complex **1B**+CO, CO coordination is endergonic by 22.2 kJ/mol; and CO insertion has barrier of 37.9 kJ/mol and is exergonic by 29.0 kJ/mol. In acyl complex **1D**, not only the carbon atom but also the oxygen atom of the C=O group interact with the Pd center.

Starting from acyl complex **1D**, CO coordination resulting in the formation of [**L1**Pd(CO)(-CO-CH_2CH_3)]$^+$ intermediate (**1D-CO**) which is endergonic by 18.1 kJ/mol and therefore CO does not affect the acyl complex. Alternatively, acyl complex **1D** undergoes methanolysis with CH_3OH to form ester [CH_3CH_2-CO-OCH_3] and then regenerates the

active catalyst [**L1**Pd-H]$^+$. Starting from complex **1D**+CH_3OH, methanolysis has barrier of 155.1 kJ/mol and is exergonic by 44.9 kJ/mol. On the basis of the potential energy surface, one can see that the acyl complex **1D** as the most stable species is the resting state; and methanolysis as the highest energy point is the rate-determining step; and the effective barrier is 155.1 kJ/mol. Due to the structure of the ligand backbone, we searched different orientations of methanol, acyl group and ligand; the reported transition state has the lowest energy.

To reduce the barrier, Mecking *et al.*,[7] investiagted a three-methanol cluster assisted methanolysis step for methyl oleate alkoxycarbonylation. It is found that the effective barrier is 124.3 kJ/mol, which is 20.0 kJ/mol (or 4.8 kcal/mol) lower than that of the single methanol route (144.3 kJ/mol or 29.7 kcal/mol). On the basis of this idea, we also calculated the methanolysis step by using a three-methanol cluster. However, we found that the effective barrier is 160.6 kJ/mol, and is 5.5 kJ/mol higher than that of the single methanol route (155.1 kJ/mol).

In order to find the reason for the opposite result as reported by Mecking *et al.*, we checked the reported structure of the three-methanol cluster at the same level of theory [B3LYP/6-31g(d) and LANL2DZ effective core potential for Pd]. It is found that the reported planar geometry in C_s symmetry of the three-methanol cluster by Mecking is indeed not an energy minimum structure. Instead, it has three imaginary frequencies (*i*175.3 cm^{-1}, *i*102.2 cm^{-1} and *i*95.9 cm^{-1}) and represents a higher order saddle point, which is 25.1 kJ/mol (or 6.0 kcal/mol) higher than the non-planar energy minimum structure used in our work (see Table S6). Taking this energy difference into account, the effective barrier of the three-methanol route by Mecking should be 149.4 kJ/mol, which is 5.1 kJ/mol higher in energy than the single methanol route (144.3 kJ/mol). This corrected result is in fully agreement with our result obtained here. Therefore, the three-methanol cluster assisted route does not bring the expected energetic advantage.

(b) Reaction with L1Pd complex in methanol solution as well as including van der Waals dispersion

Under the consideration of methanol as solvent; we computed the same elementary steps as in gas phase and the potential energy surface is shown in Scheme S2 for comparison. Once again, no stable intermediate of side-on coordinated ethene to active catalyst [**S-L1**PdH]$^+$ species (**S-1A**) was located and all attempts to optimize such structures also resulted in the spontaneous formation of ethyl complex via β-H agostic interaction, [**S-L1**Pd(-CH_2CH_3)]$^+$ (**S-1B**); and this step is exergonic by 19.1 kJ/mol, which is less by 8.0 kJ/mol than that in gas phase.

On the basis of ethyl complex **S-1B**, the next step is CO coordination to form complex [**S-L1**Pd(CO)(-CH_2CH_3)]$^+$ (**S-1C**), followed by CO insertion leading to in the formation of acyl complex, [**S-L1**Pd(-CO-CH_2CH_3)]$^+$ (**S-1D**). Starting from complex **S-1B**+CO, CO coordination is endergonic by 11.2 kJ/mol; and CO insertion has barrier of 24.8 kJ/mol and is exergonic by 50.4 kJ/mol.

Starting from acyl complex **S-1D**, further CO coordination resulting in the formation of [**L1**Pd(CO)(-CO-CH_2CH_3)]$^+$ (**S-1D-CO**) is endergonic by 7.8 kJ/mol and does not affect acyl complex. Alternatively, acyl complex **S-1D** undergoes methanolysis with CH_3OH to form the ester [CH_3CH_2-CO-OCH_3] and then regenerates the active catalyst [**S-L1**PdH]$^+$. Starting from complex **S-1D**+CH_3OH, the methanolysis has barrier of 177.2 kJ/mol and is exergonic by 52.3 kJ/mol. The potential surfaces show that acyl complex **S-1D** is the resting state; and methanolysis is the rate-determining step; and the effective barrier is 177.2 kJ/mol. Again, the reported transition state has the lowest energy among several conformations on the basis of different orientations among methanol, acyl group and ligand.

On the basis of a three-methanol cluster, we calculated the Gibbs free energies which are obtained from the solvent corrected electronic energies and thermal correction from the gas phase. It is found that the effective barrier is raised from 177.2 to 191.7 kJ/mol, and apparent barrier is raised from 107.7 to 122.2 kJ/mol, indicating that there is no expected energy lowering and stabilizing effect by methanol clustering, instead it raises the barrier by 14.5 kJ/mol.

Furthermore, van der Waals dispersion energies have been included by single point calculation at the B3PW91-GD3BJ-SCRF/LANL2DZ(Pd)/TZVP/SMD(MeOH) level of theory. Taking dispersion into consideration, the formation of [**L1**Pd(-CH_2CH_3)]$^+$ (**S-D3-1B**) is highly exergonic by 55.0 kJ/mol, which is more exergonic by 27.9 and 35.9 kJ/mol than in gas phase and methanol without dispersion, respectively. Starting from complex **S-D3-1B**, CO coordination is exergonic by 13.2 kJ/mol which is opposite to the results calculated without dispersion and CO insertion has barrier of 14.5 kJ/mol and is exergonic by 46.2 kJ/mol.

Starting from acyl complex **S-D3-1D**, further CO coordination resulting in the formation of [**L1**Pd(CO)(-CO-CH_2CH_3)]$^+$ (**S-D3-1D-CO**) is exergonic by 19.7 kJ/mol and this is opposite to the results calculated without dispersion. The coordination of CH_3OH is endergonic by 21.2 kJ/mol, and methanolysis through [**L1**Pd(CH_3OH)(-CO-CH_2CH_3)]$^+$ (**S-D3-1D-**CH_3OH) to form the ester [CH_3CH_2-CO-OCH_3] and to regenerate the active catalyst [**S-L1**PdH]$^+$ has barrier of 170.8 kJ/mol and is exergonic by 9.1 kJ/mol. The potential surfaces show that acyl complex with CO coordination **S-D3-1D-CO** is also the resting state; and methanolysis as the highest energy point is the rate-determining step; and the effective barrier is 170.8 kJ/mol.

Compared with the potential energy surface without dispersion, all species were further stabilized. It is noted that the CO coordinated ethyl (**S-D3-1C)** and acyl complex **(S-D3-1D-CO)** become more stable than those without CO coordination, and this is just opposite to the results in gas and methanol without dispersion. Furthermore, it is found that the effective barrier is 155.1 kJ/mol in gas phase, 177.2 kJ/mol in methanol and 170.8 kJ/mol in methanol solution by including dispersion.

(c) Reaction with L2Pd complex in gas phase

The potential energy surfaces for **L2**Pd are shown in Scheme S3. In contrast to **L1**Pd, the protonation of **L2**Pd can have to isomers, one with proton at the Pd center [**L2**Pd-H]$^+$ (**2A**) and one with proton at the N center of the pyridyl substitution [NH-**L2**Pd]$^+$ (**N-2A**). It is found that **2A** is more stable than **N-2A** in gas phase; and the energy difference of 7.0 kJ/mol indicates dynamic equilibrium in favor of **2A** (94 %). Starting from [**L2**Pd-H]$^+$, no stable complex of ethene side-on coordination was located, and spontaneous formation of ethyl complex via β-H agostic interaction, [**L2**Pd(CH_2CH_3)]$^+$ (**2B**), was found. The formation of ethyl complex (**2B**) is barrier-less and exergonic by 28.7 kJ/mol.

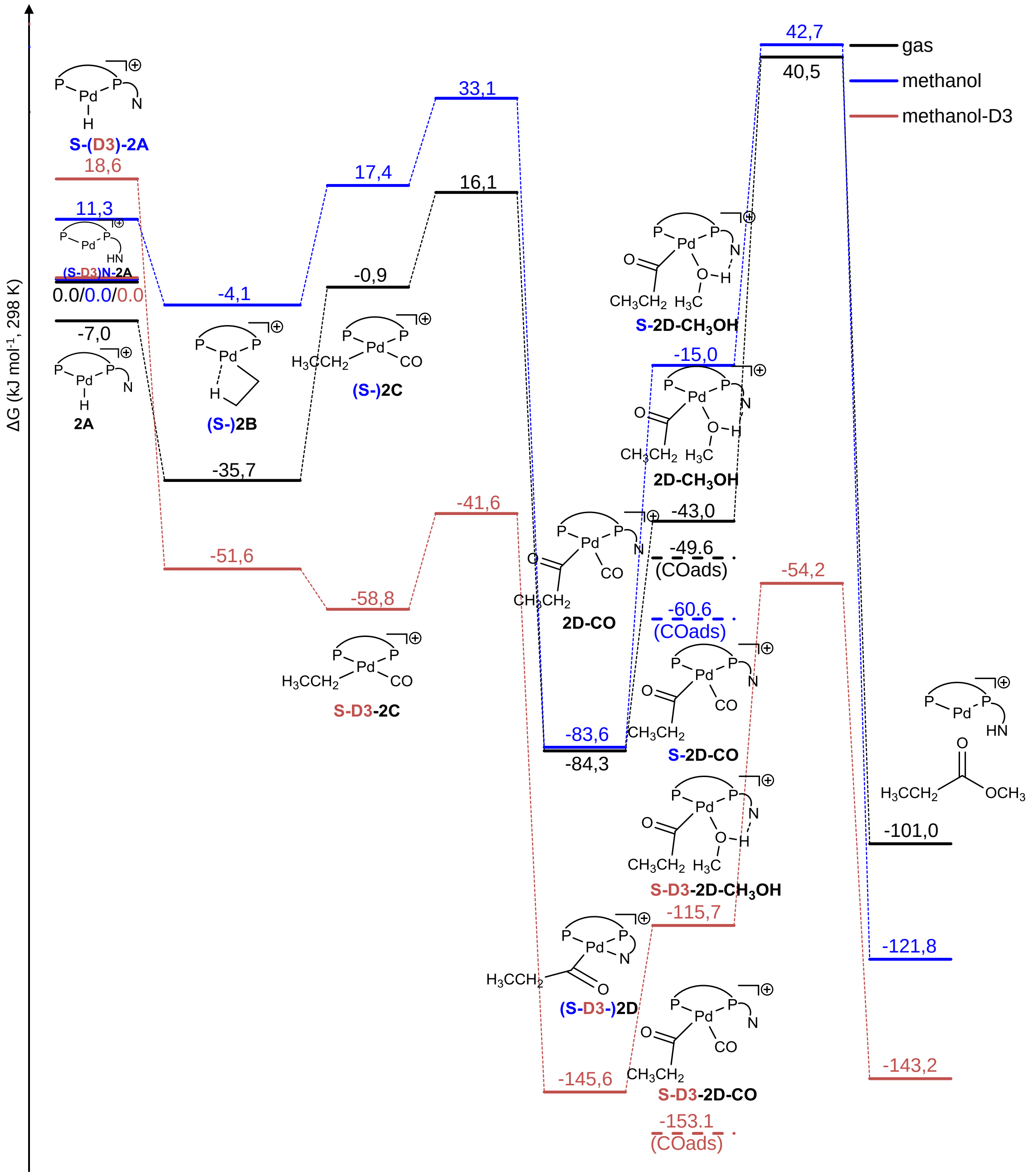

Scheme S3. Potential energy surface by using $[NH\text{-}\mathbf{L2}Pd]^+$ in ethene methoxycarbonylation in gas phase (black line), in methanol solution (blue line with **S**) and n methanol solution including van der Waals dispersion correction (bark-red line with **D3**)

On the basis of ethyl complex **2B**, the next step is CO coordination to form complex $[\mathbf{L2}Pd(CO)(\text{-}CH_2CH_3)]^+$ (**2C**), followed by CO insertion resulting in the formation of acyl complex, $[\mathbf{L2}Pd(\text{-}CO\text{-}CH_2CH_3)]^+$ (**2D**). Starting from complex **2B**+CO, CO coordination is endergonic by 34.8 kJ/mol; and the formation of **2D** has barrier of 51.8 kJ/mol and is exergonic by 48.6 kJ/mol.

Starting from acyl complex **2D**, the formation of $[\mathbf{L2}Pd(CO)(\text{-}CO\text{-}CH_2CH_3)]^+$ intermediate (**2D-CO**) from CO coordination is endergonic by 34.7 kJ/mol. Alternatively, the formation of the methanol adduct $[\mathbf{L1}Pd(CH_3OH)(\text{-}CO\text{-}CH_2CH_3)]^+$ (**2D-CH$_3$OH**) is endergonic by 41.3 kJ/mol. Starting from complex **1D**+CH_3OH, methanolysis has barrier of 124.8 kJ/mol and is exergonic by 16.7 kJ/mol. The potential energy surface shows that acyl complex **2D** is the resting state and methanolysis is the rate-determining step and the effective barrier is 124.8 kJ/mol, which is lower than that for **L1Pd** (155.1 kJ/mol) in gas phase by 30.3 kJ/mol; indicating that **L2**Pd is more active than **L1**Pd in gas phase.

It is the noted that reported transition state has the lowest energy among several conformations on the basis of different orientations among methanol, the acyl group and the ligand.

(d) Reaction with L2Pd complex in methanol solution as well as including van der Waals dispersion

Under the consideration of methanol as solvent; we computed the same elementary steps as in gas phase; and the potential energy surface is shown in Scheme S3. It is worth noting that **S-N-2A** is more stable than **S-2A** by 11.3 kJ/mol indicates dynamic equilibrium in favor of **S-2A** (99.0%), and this is just the opposite to the results in gas phase. Once again, no stable intermediate of side-on coordinated ethene to active catalyst $[$**L2**$PdH]^+$ species (**S-2A**) in methanol was located and all attempts to optimize such structures resulted in the spontaneous formation of ethyl complex via H-agostic interaction, $[$**L2**$Pd(CH_2CH_3)]^+$ (**S-2B**); and this step is exergonic by 15.4 kJ/mol from **S-2A**.

On the basis of ethyl complex **S-2B**, the next step is CO coordination to form complex $[$**S-L2**$Pd(CO)(CH_2CH_3)]^+$ (**S-2C**), followed by CO insertion (carbonylation) leading to in the formation of acyl complex, $[$**L2**$Pd(\text{-}CO\text{-}CH_2CH_3)]^+$ (**S-2D**). Starting from complex **S-2B**+CO, CO coordination is endergonic by 21.5 kJ/mol; and CO insertion has barrier of 37.2 kJ/mol and is exergonic by 79.5 kJ/mol.

Starting from acyl complex **S-2D**, further CO coordination resulting in the formation of $[$**L2**$Pd(CO)(\text{-}CO\text{-}CH_2CH_3)]^+$ (**S-2D-CO**) is endergonic by 23.0 kJ/mol and does not affect acyl complex. Alternatively, acyl complex **S-2D** can also coordinate one CH_3OH molecule to form the methanol adduct $[$**L2**$Pd(CH_3OH)(\text{-}CO\text{-}CH_2CH_3)]^+$, (**S-2D-CH$_3$OH**), which can undergo methanolysis to form the ester $[CH_3CH_2\text{-}CO\text{-}OCH_3]$ and then regenerate the active catalyst $[$**S-L2**$PdH]^+$. Starting from complex **S-2D**+CH_3OH, CH_3OH coordination is endergonic by 68.6 kJ/mol; and methanolysis has barrier of 126.3 kJ/mol and is exergonic by 38.2 kJ/mol. On the basis of the potential surfaces, one can see that acyl complex **S-2D** is the resting state; and methanolysis represents the rate-determining step; and the effective barrier is 126.3 kJ/mol, which is much lower than that (177.2 kJ/mol) for **L1**Pd in methanol solution by 50.9 kcal/mol. This indicates that **L2**Pd is also more active than **L1**Pd in methanol solution. It is noted that the reported transition state has the lowest energy among several conformations on the basis of different orientations among methanol, acyl group and ligand.

Taking dispersion into consideration, $[NH\text{-}$**L2**$Pd]^+$ (**S-D3-N-2A**) is more strongly stabilized than $[$**L2**$PdH]^+$ (**S-D3-2A)** by 18.6 kJ/mol. The formation of the ethyl complex $[$**L2**$Pd(CH_2CH_3)]^+$ (**S-D3-2B**) is exergonic by 70.4 kJ/mol from **S-D3-2A**. The CO coordination to form complex $[$**L2**$Pd(CO)(CH_2CH_3)]^+$ (**S-D3-2C**) is exergonic by 7.2 kJ/mol, and the following CO insertion has energy barrier of 17.2 kJ/mol and the formation of $[$**L2**$Pd(\text{-}CO\text{-}CH_2CH_3)]^+$ (**S-D3-2D**) is exergonic by 86.8 kJ/mol. Starting from the acyl complex **S-D3-2D**, further CO coordination $[$**L2**$Pd(CO)(\text{-}CO\text{-}CH_2CH_3)]^+$ (**S-2D-CO**) is exergonic by 7.5 kJ/mol, while CH_3OH coordination $[$**L2**$Pd(CH_3OH)(\text{-}CO\text{-}CH_2CH_3)]^+$ (**S-2D-CH$_3$OH**) is endergonic by 29.9 kJ/mol. Starting from the resting state of **S-2D-CO**, the methanolysis has barrier of 98.9 kJ/mol and is endergonic by 2.4 kJ/mol.

Compared with the potential energy surface without dispersion, all species were further stabilized, except for $[$**L2**$PdH]^+$ (**S-D3-2A)**. It is also found that the CO coordinated ethyl $[$**L2**$Pd(CO)(CH_2CH_3)]^+$ (**S-D3-2C**) and acyl **L2**$Pd(CO)(\text{-}CO\text{-}CH_2CH_3)]^+$ (**S-2D-CO**) complexes become more stable than that without CO coordination, and this is also the opposite to the results in gas and methanol without dispersion. And the effective barrier is 124.8 kJ/mol in gas phase, 126.3 kJ/mol in methanol and 98.9 kJ/mol in methanol solution including dispersion.

In addition, we also compared the theoretical and experimental results in gas phase and in methanol solution as well as in methanol solution including GD3BJ dispersion by single point calculation, as shown in Table S2.

Table S2. Effective barriers calculated in gas phase and in methanol solution as well as including GD3BJ dispersion by using L2 as ligand.

E_{eff}	Gas	Methanol	Methanol-D3(SP)	exp.
ΔG/(kJ mol^{-1})	124.8	126.3	98.9	--
ΔH/(kJ mol^{-1})	66.9	67.0	86.1	41.9
ΔS/(J mol^{-1} K^{-1})	-194.2	-198.9	-42.9	--

Qualitatively, all results are in agreement with the experiment. The negative value of the entropy barriers in gas phase (-194.2 kJ/mol) and in methanol solution (-198.9 kJ/mol) as well as including GD3BJ dispersion (-42.9 kJ/mol) are reasonable as the transition state is more ordered. The highly corrected CCSD(T) method might give reasonable results, but it is not applicable for such larger systems. On the other hand, we are more interested in the difference between L1Pd and L2Pd systems rather than their absolute values.

(e) Comparison between L1Pd and L2Pd.

To show the significant difference in activity of the currently used industrial ligand L1 and our ligand L2, we compared the effective and apparent barriers in gas phase and in methanol solution as well as in methanol solution includeing van der Waals dispersion correction (Table S3). It shows that the computed effective barrier of the L1Pd system in gas phase and in methanol solution as well as in methanol solution including van der Waals dispersion correction (155.1, 177.2 and 170.8 kJ/mol, respectively) are higher than those (124.8, 126.3 and 98.9 kJ/mol, respectively) of the L2Pd system by 30.3, 50.9 and 71.9 kJ/mol, respectively.

Table S3. Comparison between L1Pd and L2Pd system in effective (ΔG_{eff}) and apparent (ΔG_{app}) free energy barriers (kJ/mol)

ΔG	L1Pd ΔG_{eff}	L2Pd ΔG_{eff}	L1Pd-L2Pd $\Delta G_{eff} - \Delta G_{eff}$	L1Pd ΔG_{app}	L2Pd ΔG_{app}	L1Pd-L2Pd $\Delta G_{app} - \Delta G_{app}$
Gas	155.1	124.8	30.3	99.0	40.5	58.5
Methanol	177.2	126.3	50.9	107.7	42.7	65.0
Methanol-D3(SP)	170.8	98.9	71.9	36.7	-54.2	90.9

It also shows that the computed apparent barrier of the L1Pd system in gas phase and in methanol solution as well as in methanol solution including van der Waals dispersion correction (99.0, 107.7 and 36.7 kJ/mol, respectively) are higher than those (40.5, 42.7 and -54.2 kJ/mol, respectively) of the L2Pd system by 58.5, 65.0 and 90.9 kJ/mol, respectively. It is noted that including dispersion lowers both effective and apparent energy barriers, however, the negative apparent free energy barrier for L2Pd system is unreasonable.

Consequently, all these calculation results show that the L2Pd system has both lower effective and apparent barriers than the L1Pd system; and therefore the L2Pd system is more active than the L1Pd system. This is qualitatively in full agreement with the experimental observation.

Table S4. The Cartesian Coordinates (xyz) for All Optimized Structures

**B3PW91-gas phase**	**B3PW91-SCRF-methanol**
**CO**	**S-CO**
C 0.00000000 0.00000000 -0.64366200<br>O 0.00000000 0.00000000 0.48274700	C 0.00000000 0.00000000 -0.64334700<br>O 0.00000000 0.00000000 0.48251000
**CH_3OH**	**S-CH_3OH**
C 0.04654900 0.66052000 0.00000000<br>H 1.08923700 0.97933700 0.00000000<br>H -0.43789900 1.07873600 0.89167400<br>H -0.43789900 1.07873600 -0.89167400<br>O 0.04654900 -0.75523400 0.00000000<br>H -0.86512200 -1.05805600 0.00000000	C 0.04772500 0.66650000 0.00000000<br>H 1.08945100 0.99087300 0.00000000<br>H -0.44418500 1.07128400 0.89145300<br>H -0.44418500 1.07128400 -0.89145300<br>O 0.04772500 -0.75957400 0.00000000<br>H -0.86923700 -1.05584500 0.00000000
**$(CH_3OH)_3$**	**S-$(CH_3OH)_3$**
O 0.70322300 -1.34359000 -0.49595500<br>C 1.33494200 -2.37717800 0.23405700<br>H 2.34622000 -2.48977900 -0.15892400<br>H 1.40201400 -2.15033300 1.30500800<br>H 0.81400300 -3.33395400 0.11289500<br>H -0.19540400 -1.19001400 -0.14289300<br>O -1.52488500 -0.05340300 0.46919500<br>C -2.79860300 0.15368200 -0.11335200<br>H -3.30222400 1.02605000 0.31845000<br>H -2.74084500 0.28456500 -1.20020300<br>H -3.40536300 -0.72780600 0.09723800<br>H -0.94640900 0.70923100 0.27195800<br>O 0.63295100 1.41282300 -0.39578100<br>H 0.97440100 0.50772200 -0.53095900<br>C 1.61949400 2.20914400 0.23135000<br>H 2.51887500 2.30008000 -0.38837500<br>H 1.20053100 3.20649000 0.36967900<br>H 1.90889300 1.81722000 1.21412600	O -2.29117100 -0.51716200 -0.70063000<br>C -3.04871600 -0.98591300 0.40520700<br>H -3.94062600 -1.48224300 0.01668400<br>H -2.48871000 -1.71111700 1.00845000<br>H -3.37078100 -0.16648300 1.05951800<br>H -1.48878200 -0.06931900 -0.35170100<br>O -0.07817500 0.74233800 0.32549300<br>C -0.04093400 2.13936900 0.05272700<br>H 0.81037500 2.61864600 0.54773200<br>H 0.01653400 2.34106700 -1.02261800<br>H -0.96029400 2.58056400 0.44109200<br>H 0.75045500 0.33505100 -0.01404800<br>O 2.25634900 -0.35894700 -0.59380700<br>H 2.07959500 -1.00612400 -1.28681400<br>C 3.04583300 -0.98343900 0.42100200<br>H 4.00488500 -1.32271900 0.01856600<br>H 3.23173300 -0.23426200 1.19078500<br>H 2.52249700 -1.83299900 0.87028900
**C_2H_4**	**S-C_2H_4**
C 0.00000000 0.66233600 0.00000000<br>H 0.92286000 1.23317800 0.00000000<br>H -0.92281500 1.23323000 0.00000000<br>C 0.00000000 -0.66233600 0.00000000<br>H -0.92286000 -1.23317800 0.00000000<br>H 0.92281500 -1.23323000 0.00000000	C 0.00000000 0.66303200 0.00000000<br>H 0.92428900 1.23438200 0.00000000<br>H -0.92423700 1.23444400 0.00000000<br>C 0.00000000 -0.66303200 0.00000000<br>H -0.92428900 -1.23438200 0.00000000<br>H 0.92423700 -1.23444400 0.00000000
**$CH_3OCOCH_2CH_3$**	**S-$CH_3OCOCH_2CH_3$**
H 2.56694300 -0.56886200 0.87895300<br>C 0.70130200 -1.23112300 0.00000000<br>C 2.21486300 -1.11089800 0.00000000<br>H 2.56694300 -0.56886200 -0.87895300<br>C 0.00000000 0.10428200 0.00000000<br>O 0.53592300 1.18278700 0.00000000<br>O -1.33822600 -0.05582000 0.00000000<br>C -2.10739500 1.15002400 0.00000000<br>H -1.89058400 1.74645900 0.88746100<br>H -1.89058400 1.74645900 -0.88746100	H 2.57056700 -0.61088300 0.88377400<br>C 0.69063200 -1.23535600 0.00000000<br>C 2.20438000 -1.13828500 0.00000000<br>H 2.57056700 -0.61088300 -0.88377400<br>C 0.00000000 0.09821400 0.00000000<br>O 0.55046800 1.18063100 0.00000000<br>O -1.33113900 -0.03651000 0.00000000<br>C -2.10524100 1.17505200 0.00000000<br>H -1.89294400 1.76406200 0.89336100<br>H -1.89294400 1.76406200 -0.89336100

H -3.14859500 0.83537200 0.00000000	H -3.14541100 0.85645000 0.00000000
H 0.34345100 -1.78947600 0.87070000	H 0.32767300 -1.78719500 0.87312900
H 0.34345100 -1.78947600 -0.87070000	H 0.32767300 -1.78719500 -0.87312900
H 2.67477800 -2.10106400 0.00000000	H 2.64156200 -2.13912800 0.00000000
**1A**	**S-1A**
Pd 0.06946100 -1.26799100 0.81346900	Pd 0.06181000 -1.31779700 0.83429000
P -1.98351600 -0.30527800 -0.06088900	P -1.94185000 -0.28338700 -0.07515600
P 1.93987700 -0.36589500 -0.02309700	P 1.88705600 -0.36495500 -0.03000600
C -1.70562000 1.01835400 -1.35600000	C -1.71736000 1.08294000 -1.32777500
C 1.40272200 0.83361400 -1.37390200	C 1.38295000 0.85634500 -1.36637600
C 0.62533100 2.09842000 -1.11245200	C 0.62712000 2.13087100 -1.09380300
C 1.36068700 3.28633400 -1.02441900	C 1.37934300 3.30918000 -1.00367400
C 0.75913700 4.52462600 -0.88749200	C 0.79410500 4.55471500 -0.84808800
C -0.62542100 4.60376900 -0.86638900	C -0.59034200 4.65124200 -0.80518600
C -1.36937100 3.44340400 -0.98820000	C -1.35155000 3.50042400 -0.92814300
C -0.77940400 2.18007800 -1.10365600	C -0.77704700 2.23100000 -1.06972400
H 2.44203200 3.23861000 -1.08732100	H 2.45931400 3.24104300 -1.07115500
H 1.36623100 5.41994600 -0.82489000	H 1.41487200 5.44173500 -0.78079900
H -1.12345600 5.56259500 -0.78369600	H -1.07626800 5.61531000 -0.69888600
H -2.45148600 3.51698600 -1.02028800	H -2.43360600 3.58254400 -0.93494100
H 2.33097200 1.11144100 -1.87690700	H 2.31953200 1.12921200 -1.85540100
H 0.86226900 0.18201600 -2.06496300	H 0.83037700 0.22591100 -2.06779500
H -1.35296600 0.45096800 -2.22254300	H -1.39388300 0.55180400 -2.22830300
H -2.68463600 1.41205200 -1.63382600	H -2.70590500 1.49005800 -1.54641100
C 2.95003600 -1.63762700 -1.03621600	C 2.90315500 -1.62365300 -1.05730200
C 2.94500500 0.55969500 1.30422200	C 2.91027200 0.54150900 1.29975800
C -2.98020500 -1.58717900 -1.06950500	C -2.92487900 -1.55228900 -1.11136600
C -2.99129400 0.42978500 1.37754300	C -2.97005800 0.39922600 1.37352800
H 1.40679800 -1.88504900 1.35928100	H 1.35634400 -1.98145300 1.41636100
C -2.96295800 -0.59178600 2.52360000	C -2.92997100 -0.64335400 2.49776200
H -3.47147100 -0.16407400 3.39283600	H -3.43232100 -0.23103400 3.37967400
H -3.46185500 -1.52897900 2.28235700	H -3.43316400 -1.57417900 2.23831900
H -1.93564100 -0.81569100 2.83219200	H -1.89913100 -0.87705500 2.78625200
C -4.43389300 0.79638800 1.03166900	C -4.41481700 0.74800900 1.02553200
H -4.88489300 1.30787500 1.88750200	H -4.87794700 1.24175300 1.88736600
H -4.49875400 1.47752700 0.18019600	H -4.48139400 1.43648100 0.17979200
H -5.04878600 -0.07932600 0.82093500	H -5.01044000 -0.13750900 0.79894700
C -2.24889500 1.68102500 1.85904100	C -2.25632700 1.65390500 1.88615200
H -2.67192500 1.99168100 2.81891700	H -2.70109000 1.94046900 2.84514300
H -1.18411200 1.49077900 2.01133400	H -1.19022300 1.47969000 2.05357300
H -2.34559200 2.51615900 1.16874900	H -2.36312600 2.49766100 1.20627000
C 1.99154600 1.56170600 1.96872400	C 1.97131500 1.54701500 1.97597600
H 2.54736300 2.10165300 2.74073900	H 2.53279800 2.06394600 2.76101700
H 1.58163500 2.29596000 1.27874500	H 1.58307200 2.30099200 1.29412400
H 1.16424100 1.04491800 2.46041600	H 1.12736100 1.03858200 2.44928600
C 4.14174400 1.28029600 0.67611400	C 4.11237500 1.25291000 0.67542300
H 4.61855700 1.89753200 1.44244800	H 4.59374800 1.86311200 1.44651500
H 4.89543700 0.58160000 0.31057000	H 4.85474300 0.54258100 0.30756500
H 3.85864300 1.94458800 -0.14043400	H 3.83368200 1.91823400 -0.14205100
C 3.43901500 -0.38582500 2.40338400	C 3.40116300 -0.40965600 2.39394500
H 4.18773100 -1.09627000 2.06257300	H 4.14046500 -1.12604600 2.04342200
H 3.90019500 0.22575400 3.18416200	H 3.87481600 0.19952800 3.17086600
H 2.61801800 -0.93783400 2.86419900	H 2.57760000 -0.95583100 2.85806900
C -3.54922600 -2.65124100 -0.12659400	C -3.49617300 -2.63571400 -0.19429000

H	-3.95416200	-3.47407900	-0.72264200	H	-3.87672300	-3.45536500	-0.81313300
H	-2.78244800	-3.07185400	0.53034400	H	-2.73340900	-3.05144500	0.47065500
H	-4.36276300	-2.26954500	0.49018700	H	-4.32661200	-2.27234600	0.41233000
C	-1.97974800	-2.27521700	-2.00811700	C	-1.90861000	-2.21814300	-2.04729300
H	-1.17388100	-2.76145800	-1.45499700	H	-1.12339000	-2.72955900	-1.48685900
H	-2.50414700	-3.04400500	-2.58317800	H	-2.42911500	-2.96341500	-2.65840800
H	-1.53516800	-1.58728800	-2.73108800	H	-1.43739400	-1.50908700	-2.73206400
C	-4.10913400	-0.98511700	-1.91117100	C	-4.04670400	-0.94647500	-1.95741600
H	-4.62629600	-1.79601900	-2.43315700	H	-4.54081400	-1.75519300	-2.50738700
H	-4.85171300	-0.46012200	-1.31239200	H	-4.80716400	-0.44690400	-1.35786800
H	-3.73970500	-0.30238700	-2.67806600	H	-3.67103800	-0.23781500	-2.69807900
C	3.83281200	-2.48923300	-0.12274500	C	3.79268300	-2.47976800	-0.15684300
H	4.21944500	-3.32927700	-0.70633500	H	4.18537700	-3.30656900	-0.75748700
H	4.69285000	-1.93649300	0.25447100	H	4.64636700	-1.92119300	0.22744400
H	3.27907900	-2.90612800	0.72099700	H	3.24241200	-2.91356000	0.68137900
C	3.82491700	-0.97096700	-2.10742300	C	3.77452300	-0.95010600	-2.12468300
H	4.44199700	-1.74601100	-2.57013200	H	4.38944100	-1.72560300	-2.59251800
H	3.23671800	-0.51702500	-2.90574700	H	3.18135600	-0.48994800	-2.91624500
H	4.50264900	-0.21939800	-1.70223800	H	4.45066500	-0.20017800	-1.71329100
C	1.93871200	-2.56048300	-1.72742100	C	1.89491200	-2.54073700	-1.75714100
H	1.36140100	-3.13760900	-1.00202600	H	1.30688000	-3.11543200	-1.03746200
H	1.24498700	-2.02036500	-2.37437400	H	1.21240300	-1.98961800	-2.40683300
H	2.48703800	-3.26610000	-2.35826500	H	2.44869800	-3.24689900	-2.38474600
**1B**				**S-1B**			
Pd	0.08432300	-1.32788400	-0.58761000	Pd	0.07349000	-1.32243900	-0.58869700
P	1.88062500	-0.06630900	0.21978700	P	1.86852500	-0.07505000	0.21613400
P	-1.91293600	-0.20611600	0.21768800	P	-1.90446400	-0.18751400	0.22183300
C	1.36510100	1.43021000	1.23683500	C	1.35842400	1.41688600	1.24050500
C	-1.68334100	1.31636200	1.28093900	C	-1.69634400	1.34621900	1.26798300
C	-0.90763000	2.49742000	0.76753200	C	-0.89960000	2.51410200	0.75829000
C	-1.63433200	3.64517300	0.43751000	C	-1.60924700	3.67029500	0.41380900
C	-1.01944300	4.81895100	0.03709900	C	-0.97499000	4.83830600	0.02311100
C	0.36519700	4.87360900	-0.00507400	C	0.41242500	4.87763100	0.00159000
C	1.09960300	3.75431000	0.35027400	C	1.12933400	3.74796400	0.36407000
C	0.49630100	2.54725700	0.71728500	C	0.50689500	2.54643900	0.72264700
H	-2.71625500	3.61901300	0.51453700	H	-2.69290400	3.64831700	0.46749600
H	-1.61576400	5.68941500	-0.20982600	H	-1.55927500	5.71435700	-0.23777900
H	0.87399900	5.78985600	-0.28051700	H	0.93720900	5.78774700	-0.26898000
H	2.17849900	3.83916400	0.38625800	H	2.20949800	3.81279500	0.40790900
H	-2.67739400	1.65782300	1.57007100	H	-2.69583500	1.69822700	1.52328500
H	-1.22095800	0.92496400	2.19232100	H	-1.25269700	0.96727500	2.19386200
H	0.87870700	0.98116100	2.10318700	H	0.84954400	0.96189600	2.09078400
H	2.29954700	1.85990300	1.60506200	H	2.29079900	1.83562400	1.62560700
C	-2.90764400	-1.31004800	1.43604300	C	-2.90325000	-1.28459100	1.44050400
C	-2.95694500	0.32509300	-1.29456300	C	-2.95046900	0.31731900	-1.29829700
C	2.86920300	-1.03538500	1.56098900	C	2.85014600	-1.04958400	1.55919900
C	3.02504800	0.58184400	-1.16908500	C	3.02539900	0.56813800	-1.16653000
C	1.16172800	-2.70509000	-1.72524200	C	1.14088800	-2.70042200	-1.72225600
C	-0.16341500	-3.35140100	-1.80562100	C	-0.16999000	-3.38193800	-1.75955700
H	1.51258900	-2.23811900	-2.64078300	H	1.45218900	-2.23255600	-2.65290000
H	-0.57453700	-3.42575300	-2.81268600	H	-0.59373000	-3.49615800	-2.75859000
H	1.93689500	-3.24961400	-1.20184400	H	1.94538500	-3.22257500	-1.21943800
C	3.91768000	-0.53971100	-1.70718600	C	3.89819700	-0.56318700	-1.71425800
H	4.42376600	-0.17460400	-2.60557300	H	4.41697900	-0.19126000	-2.60422000

H 4.69272200 -0.82569600 -0.99566800<br>H 3.36023800 -1.43040600 -1.98695900<br>C 3.92761300 1.73881600 -0.73117800<br>H 4.55656800 2.02005600 -1.58103200<br>H 3.36014400 2.62217400 -0.45246300<br>H 4.59307500 1.47654800 0.08916600<br>C 2.09514000 1.07443800 -2.28553500<br>H 2.70268200 1.46359100 -3.10797900<br>H 1.47352500 0.26753100 -2.67832700<br>H 1.43906300 1.87955600 -1.94935100<br>C -2.11376300 1.32968500 -2.09315200<br>H -2.62364300 1.53412800 -3.03947600<br>H -1.97554900 2.27717000 -1.57882200<br>H -1.12729600 0.92343100 -2.32986600<br>C -4.29772200 0.95303500 -0.91354200<br>H -4.77412800 1.35028400 -1.81501100<br>H -4.98577100 0.22594000 -0.48041800<br>H -4.18979800 1.78506600 -0.21546000<br>C -3.19464000 -0.87331700 -2.22135100<br>H -3.77068600 -1.67609800 -1.76709900<br>H -3.75362400 -0.52486600 -3.09481700<br>H -2.25215600 -1.28356500 -2.59000500<br>C 3.16833300 -2.45519000 1.07410500<br>H 3.76630600 -2.96486000 1.83501800<br>H 2.24823600 -3.02481200 0.93932900<br>H 3.73373300 -2.48048100 0.14380200<br>C 1.99034000 -1.16048600 2.81323600<br>H 0.98469000 -1.51059900 2.57337400<br>H 2.44314700 -1.89979000 3.47985600<br>H 1.91833100 -0.22922200 3.37640500<br>C 4.18094200 -0.34654800 1.94791900<br>H 4.61693900 -0.88757300 2.79325200<br>H 4.91604600 -0.36499100 1.14356200<br>H 4.04162800 0.68674000 2.27028000<br>C -3.62254000 -2.44464400 0.69880200<br>H -4.00180600 -3.15635600 1.43785700<br>H -4.47972300 -2.09157500 0.12513200<br>H -2.95794200 -2.99730200 0.03105100<br>C -3.93581800 -0.54077500 2.27381600<br>H -4.48908100 -1.25791400 2.88755200<br>H -3.46958300 0.16775600 2.95942700<br>H -4.66564600 -0.00562600 1.66645600<br>C -1.87687600 -1.93413900 2.38552100<br>H -1.15496800 -2.55716100 1.85165200<br>H -1.32873200 -1.18233800 2.95780600<br>H -2.39661800 -2.56930000 3.10913900<br>H -0.24018600 -4.29799200 -1.27296500<br>H -0.99215000 -2.70246000 -1.28493600	H 4.66033600 -0.87407100 -0.99877300<br>H 3.32457500 -1.43857100 -2.01135800<br>C 3.94842600 1.70590100 -0.72542100<br>H 4.57743000 1.98032300 -1.57926900<br>H 3.39992600 2.59785700 -0.43373900<br>H 4.61209200 1.42397400 0.09043500<br>C 2.10613000 1.08025900 -2.28147700<br>H 2.72440300 1.46008100 -3.10189600<br>H 1.46875000 0.28662200 -2.67754100<br>H 1.46662600 1.89823200 -1.94297300<br>C -2.13152300 1.34631200 -2.08786700<br>H -2.62378700 1.51352500 -3.05193800<br>H -2.05869200 2.30642100 -1.58181200<br>H -1.11889300 0.98609600 -2.28944000<br>C -4.31183700 0.90788800 -0.93677800<br>H -4.79187700 1.27577800 -1.85071800<br>H -4.97652100 0.16235100 -0.49733400<br>H -4.23286300 1.75229700 -0.24831600<br>C -3.13713700 -0.88893300 -2.22504700<br>H -3.71743800 -1.69533400 -1.78115900<br>H -3.67203300 -0.55180300 -3.11962200<br>H -2.17526700 -1.29179100 -2.55038300<br>C 3.13351000 -2.47265200 1.07530200<br>H 3.72376700 -2.98445400 1.84265900<br>H 2.20819700 -3.03389100 0.93645800<br>H 3.70146200 -2.50543000 0.14623100<br>C 1.97092900 -1.16261800 2.81069100<br>H 0.96143200 -1.50346100 2.57246500<br>H 2.42117700 -1.90474000 3.47786700<br>H 1.90428500 -0.22718300 3.36828900<br>C 4.16916900 -0.37701400 1.94455300<br>H 4.59363400 -0.92332100 2.79404100<br>H 4.90306500 -0.40963100 1.13873800<br>H 4.04097100 0.66025300 2.25946600<br>C -3.61817600 -2.41474200 0.69856600<br>H -3.99601000 -3.12914000 1.43776200<br>H -4.47465400 -2.05410500 0.12702500<br>H -2.95248300 -2.96055100 0.02563800<br>C -3.92939200 -0.51376300 2.27662300<br>H -4.47870900 -1.23262900 2.89441300<br>H -3.45950700 0.19947900 2.95582400<br>H -4.66069800 0.01825800 1.66716600<br>C -1.87675200 -1.90990300 2.39244300<br>H -1.15932800 -2.54148400 1.86196800<br>H -1.32300800 -1.15727500 2.95861700<br>H -2.40516100 -2.53856200 3.11752300<br>H -0.20739700 -4.31563900 -1.19903900<br>H -1.00342900 -2.74737800 -1.24814400
**1C**<br><br>Pd -0.11772700 -1.32149500 -0.48524200<br>P -1.91016500 0.17811700 0.19051800<br>P 1.90375800 -0.07029100 0.33033700<br>C -1.35288900 1.73735500 1.08057800<br>C 1.65478400 1.50941100 1.31156700	**S-1C**<br><br>Pd -0.10877900 -1.31745100 -0.46814400<br>P -1.91227500 0.18051100 0.19512500<br>P 1.89498000 -0.06108700 0.34174300<br>C -1.37194600 1.74700400 1.07626600<br>C 1.63986100 1.51422700 1.32438800

C	0.98138600	2.69214300	0.67120500	C	0.97066600	2.69524300	0.67966900
C	1.75111000	3.81110900	0.34107100	C	1.74443500	3.81309400	0.34913400
C	1.19093100	4.96380300	-0.18383000	C	1.18894400	4.96433600	-0.18663200
C	-0.18292400	5.03165400	-0.35750500	C	-0.18475400	5.03040600	-0.37463700
C	-0.96475600	3.95284000	0.01923100	C	-0.97083600	3.95262100	0.00053100
C	-0.41235800	2.77095300	0.52258400	C	-0.42335600	2.77375500	0.51994600
H	2.81618200	3.79611900	0.53820600	H	2.80853100	3.79280200	0.55183600
H	1.82124600	5.81116300	-0.42661200	H	1.82339800	5.80959700	-0.43132400
H	-0.64768800	5.93045300	-0.74525400	H	-0.64692700	5.92548100	-0.77715800
H	-2.04269100	4.04155700	-0.05424900	H	-2.04799200	4.03359500	-0.08927800
H	2.63637200	1.81116500	1.68187400	H	2.61642400	1.81667100	1.70689400
H	1.08224200	1.19192400	2.18442400	H	1.05083300	1.19477100	2.18543600
H	-0.95438800	1.35244300	2.02309800	H	-0.98407900	1.37009200	2.02649600
H	-2.27658100	2.25132300	1.34119100	H	-2.29826000	2.26727100	1.31452800
C	2.72496400	-1.19063700	1.65692900	C	2.68111500	-1.19454200	1.67468600
C	3.13721500	0.38518200	-1.06486000	C	3.16090700	0.37915700	-1.02815000
C	-3.08547300	-0.49469900	1.57912400	C	-3.12590600	-0.48138000	1.55724000
C	-2.85603900	0.78683100	-1.35791400	C	-2.83241500	0.75483900	-1.37980900
C	-1.58243400	-2.81512400	-1.00152200	C	-1.53761100	-2.85569300	-0.92582200
C	-1.38816100	-4.07067800	-0.17474600	C	-1.20530900	-4.11818900	-0.15472900
H	-2.55266900	-2.37193300	-0.83230400	H	-2.50975200	-2.47086200	-0.65221800
H	-0.46021500	-4.59759300	-0.40456000	H	-0.29599100	-4.60640200	-0.51350800
H	-1.39435300	-3.87639200	0.89955300	H	-1.08078800	-3.94083000	0.91631000
C	0.91009300	-2.57912700	-1.54355700	C	0.89701800	-2.48975700	-1.62094300
O	1.37111700	-3.32200400	-2.26280300	O	1.32981900	-3.16419100	-2.42454600
H	-2.20731400	-4.77263300	-0.37336700	H	-2.02385800	-4.84307500	-0.26206000
H	-1.50721900	-3.01658700	-2.07042500	H	-1.54505700	-3.03072400	-2.00239700
C	2.28652800	0.96551300	-2.20246300	C	2.33945400	0.93010500	-2.19994900
H	2.94517800	1.27271200	-3.02046800	H	3.02317000	1.24741900	-2.99491100
H	1.58621000	0.22745800	-2.60008900	H	1.67371800	0.16980500	-2.61587200
H	1.71923200	1.84281300	-1.88822500	H	1.73856900	1.79627300	-1.91538200
C	4.18033500	1.42177500	-0.63849500	C	4.18510300	1.42766900	-0.58956000
H	4.76805300	1.10786900	0.22308700	H	4.73825300	1.13192200	0.30167500
H	4.87648500	1.56959900	-1.46954300	H	4.91096600	1.55592500	-1.39991900
H	3.73447200	2.38880200	-0.42602200	H	3.73201400	2.39884700	-0.41123300
C	3.88515300	-0.84432500	-1.58664600	C	3.93499500	-0.84919300	-1.51181100
H	4.59554600	-1.24198700	-0.86141200	H	4.64424700	-1.21224800	-0.76714000
H	3.22676700	-1.64991700	-1.90424500	H	3.29480200	-1.67617200	-1.81175500
H	4.46035100	-0.54251600	-2.46688400	H	4.51195700	-0.55393100	-2.39481800
C	-1.81270700	1.44833300	-2.26911400	C	-1.78804200	1.42079100	-2.28383900
H	-2.29963400	1.72912900	-3.20774700	H	-2.26547500	1.67191700	-3.23703800
H	-1.37126100	2.34592400	-1.84251500	H	-1.38009700	2.33904700	-1.86612000
H	-1.00701300	0.75091200	-2.51143000	H	-0.96016500	0.73882400	-2.49614300
C	-3.96439400	1.77890700	-0.99670400	C	-3.96633000	1.73085600	-1.06389100
H	-4.37109900	2.20009800	-1.92093900	H	-4.37633000	2.10277300	-2.00917000
H	-4.79112700	1.29642500	-0.47333900	H	-4.78088700	1.24853400	-0.52123400
H	-3.61500300	2.61379900	-0.39020400	H	-3.63658600	2.59622800	-0.48939200
C	-3.47254100	-0.36361600	-2.16098500	C	-3.39946500	-0.42901900	-2.16802700
H	-2.71058000	-1.03315800	-2.55638600	H	-2.60851500	-1.09434100	-2.51345200
H	-4.20160900	-0.94978100	-1.60375100	H	-4.13148300	-1.01376700	-1.61313700
H	-3.99647300	0.07291900	-3.01639900	H	-3.90281300	-0.02739200	-3.05394500
C	-3.80816200	0.61545500	2.35822100	C	-3.78907600	0.63960500	2.36940300
H	-4.40448100	1.26923400	1.72125900	H	-4.34647800	1.34242300	1.74785400
H	-4.49794700	0.13407400	3.05717000	H	-4.50463500	0.17010600	3.05213400
H	-3.13557100	1.22836500	2.95697100	H	-3.08275200	1.19779100	2.98388400

C	-4.16545900	-1.44807200	1.05531900	C	-4.25774800	-1.35345500	1.00355800
H	-3.76792900	-2.32333600	0.54580100	H	-3.91203600	-2.22216200	0.44612300
H	-4.73286500	-1.81538300	1.91549700	H	-4.82984000	-1.73124300	1.85778600
H	-4.87629900	-0.94821300	0.39747500	H	-4.94802600	-0.78671800	0.37824900
C	-2.18080800	-1.25437700	2.55607900	C	-2.26493700	-1.31874200	2.50673500
H	-1.44132500	-0.60134800	3.02464100	H	-1.46635500	-0.72740400	2.96046200
H	-2.79207600	-1.67432700	3.36038300	H	-2.89550700	-1.69677000	3.31870600
H	-1.65379800	-2.07683900	2.07014200	H	-1.81675400	-2.17560600	2.00181900
C	2.82579600	-2.63042600	1.14012600	C	2.79942100	-2.62654500	1.14253700
H	1.83776100	-3.06170600	0.96671700	H	1.81860200	-3.04690000	0.91007100
H	3.41230600	-2.72183800	0.22717800	H	3.42888700	-2.70838800	0.25758500
H	3.31599300	-3.24223900	1.90296700	H	3.24765700	-3.24855400	1.92456200
C	4.11202900	-0.70855900	2.09034900	C	4.05159700	-0.71398800	2.15498300
H	4.85209800	-0.79355400	1.29453000	H	4.81466100	-0.79377200	1.37988800
H	4.10822500	0.32120000	2.45243200	H	4.03289500	0.31496500	2.51929100
H	4.45660800	-1.33572300	2.91823900	H	4.36689500	-1.34858100	2.99066800
C	1.80090100	-1.21234800	2.88242800	C	1.71940900	-1.23504900	2.86856600
H	1.81610700	-0.27642600	3.44256300	H	1.70452500	-0.30257000	3.43511600
H	0.76927500	-1.44556800	2.60923900	H	0.69976800	-1.47465900	2.55854100
H	2.14241000	-1.99675700	3.56366200	H	2.05022700	-2.02402700	3.55160900
**TS-1C_1D**				**S-TS-1C_1D**			
Pd	0.09328900	-1.34104400	-0.24516700	Pd	0.11602600	-1.34249000	-0.25955100
P	-1.90657800	0.02133800	0.31210000	P	-1.90463400	-0.01581100	0.31344500
P	1.89023200	0.17522100	0.26885000	P	1.87533300	0.20613800	0.27042100
C	-1.57798700	1.68029100	1.12617200	C	-1.59924700	1.64194300	1.13561300
C	1.46089000	1.81115900	1.07401700	C	1.43569800	1.83199000	1.08137000
C	0.58470000	2.84143200	0.41993700	C	0.54008600	2.84652900	0.43146400
C	1.19912700	3.98167100	-0.10754100	C	1.13396000	3.99779200	-0.09956600
C	0.46888800	5.03559900	-0.62961900	C	0.38214000	5.04113300	-0.61554900
C	-0.91722700	4.97844600	-0.60333600	C	-1.00439600	4.95877600	-0.58496600
C	-1.53921600	3.86877600	-0.05672000	C	-1.60551800	3.83449500	-0.04184000
C	-0.81578800	2.78229600	0.44516200	C	-0.86024000	2.76014500	0.45773900
H	2.28142600	4.05220500	-0.08922200	H	2.21626300	4.07684100	-0.09369900
H	0.97833200	5.90462700	-1.02893400	H	0.87557700	5.91875600	-1.01943000
H	-1.51075900	5.80237300	-0.98163800	H	-1.61523200	5.77171000	-0.96302000
H	-2.62211700	3.85086000	0.00114500	H	-2.68763300	3.78701200	0.01261600
H	2.41760600	2.27973400	1.30292700	H	2.38727400	2.31585100	1.29879500
H	1.03553900	1.51321400	2.03702000	H	1.01630500	1.52700200	2.04449900
H	-1.08045400	1.40709800	2.06130000	H	-1.08485300	1.36859900	2.06121400
H	-2.55355300	2.07191700	1.41152100	H	-2.57905400	2.01607800	1.42995500
C	3.01092300	-0.55221700	1.65689500	C	3.02167700	-0.51263900	1.64174700
C	2.86312900	0.57794600	-1.32517000	C	2.83090700	0.62592500	-1.32884900
C	-2.85727500	-0.82672800	1.76916800	C	-2.80096300	-0.89789200	1.78041900
C	-3.04542200	0.38285800	-1.17982900	C	-3.08930300	0.33314100	-1.14547900
C	-2.21407300	1.18261400	-2.19200600	C	-2.30225000	1.15474500	-2.17355600
H	-2.82305600	1.35116600	-3.08532700	H	-2.93531300	1.30394600	-3.05485900
H	-1.89521800	2.15327500	-1.81972200	H	-2.01058000	2.13673200	-1.80635000
H	-1.32748100	0.62574800	-2.50283600	H	-1.40254400	0.62699500	-2.49999200
C	-4.30241700	1.16482200	-0.79403700	C	-4.34909300	1.08631400	-0.71884500
H	-4.97459700	0.58127900	-0.16366800	H	-4.98236500	0.48294100	-0.06635800
H	-4.08185300	2.10397300	-0.28509500	H	-4.13353000	2.02963900	-0.21499400
H	-4.85317400	1.41721500	-1.70513700	H	-4.93344300	1.32327300	-1.61491200
C	-3.45460900	-0.91667600	-1.88135900	C	-3.49612700	-0.96537400	-1.84754300
H	-4.04844500	-0.65548500	-2.76234100	H	-4.11905600	-0.70057500	-2.70881900

H -2.58595600 -1.47092900 -2.23755000<br>H -4.06486800 -1.57474800 -1.26670400<br>C -3.78023600 -1.95156200 1.29094800<br>H -4.64450800 -1.57411900 0.74467500<br>H -3.27355900 -2.69446500 0.67526000<br>H -4.16218200 -2.47709900 2.17131000<br>C -3.70072800 0.14960800 2.59880500<br>H -4.43516700 0.68971100 2.00057600<br>H -4.25503900 -0.42699100 3.34524800<br>H -3.09700700 0.87405200 3.14518600<br>C -1.77866500 -1.43368500 2.67353500<br>H -2.25616900 -1.90420700 3.53835200<br>H -1.19648500 -2.19888900 2.15457400<br>H -1.08614100 -0.68136400 3.05716600<br>C 3.95936100 -1.62558500 1.11753500<br>H 3.45354700 -2.40021000 0.54395400<br>H 4.75472500 -1.19979100 0.50499400<br>H 4.43966300 -2.11658500 1.96882500<br>C 2.07180300 -1.19734600 2.68337800<br>H 1.49958900 -2.02236800 2.25564300<br>H 2.66996200 -1.59747200 3.50730600<br>H 1.37017300 -0.48115100 3.11751000<br>C 3.85430500 0.50937500 2.37777500<br>H 4.52008200 -0.00670900 3.07545700<br>H 4.48464600 1.08804900 1.70223400<br>H 3.25151500 1.19874100 2.96928400<br>C 3.30450600 -0.70342900 -2.04017500<br>H 3.84379600 -0.41559500 -2.94737000<br>H 3.96911300 -1.33060100 -1.45137000<br>H 2.44867600 -1.30200400 -2.35518600<br>C 1.89471300 1.29959700 -2.27112700<br>H 2.40602000 1.47264500 -3.22269100<br>H 1.01665200 0.68371500 -2.47834800<br>H 1.55742100 2.26190800 -1.89333100<br>C 4.08767300 1.45353100 -1.04897800<br>H 4.85473900 0.92171100 -0.48459500<br>H 4.53473700 1.74483900 -2.00396800<br>H 3.84310600 2.37425500 -0.51718900<br>C -0.93770200 -3.32533500 -0.93557900<br>C -0.91492800 -3.72341400 -2.39511500<br>H -0.79030300 -4.15713300 -0.24944500<br>H -0.97303500 -2.86149500 -3.06229100<br>H -0.02392800 -4.29384000 -2.66029000<br>C 1.08153400 -2.89034400 -0.66765800<br>O 1.81882400 -3.74692700 -0.85376300<br>H -1.78150000 -4.35766600 -2.60984000<br>H -1.86288900 -2.83057200 -0.66434500	H -2.62984700 -1.50363400 -2.23226900<br>H -4.07823100 -1.63771500 -1.22053800<br>C -3.72298100 -2.02541100 1.31309500<br>H -4.61272500 -1.64809700 0.80801400<br>H -3.22624900 -2.74283200 0.66014500<br>H -4.05921200 -2.57418700 2.19952900<br>C -3.62674100 0.05106400 2.65502900<br>H -4.39138900 0.58842800 2.09192400<br>H -4.14079800 -0.55105300 3.41196100<br>H -3.01141200 0.77682400 3.18743600<br>C -1.68382300 -1.50425200 2.63548800<br>H -2.12686800 -1.98330200 3.51557700<br>H -1.12163900 -2.26252000 2.08388500<br>H -0.98113000 -0.74712700 2.99001900<br>C 4.01923100 -1.52757300 1.08358800<br>H 3.54666000 -2.31653900 0.50046900<br>H 4.78914500 -1.05266100 0.47421100<br>H 4.52214900 -2.00566100 1.93062600<br>C 2.10525700 -1.22677100 2.64140900<br>H 1.60205200 -2.08451400 2.18964900<br>H 2.71440700 -1.59506900 3.47400700<br>H 1.34704400 -0.56162400 3.06118000<br>C 3.81492400 0.56336400 2.39357000<br>H 4.49669700 0.05389700 3.08258100<br>H 4.42259300 1.18356000 1.73306900<br>H 3.17809600 1.21258200 2.99523400<br>C 3.27475000 -0.64724600 -2.05521200<br>H 3.79909200 -0.34670900 -2.96854000<br>H 3.95416500 -1.26902200 -1.47653000<br>H 2.41843900 -1.25309700 -2.35870400<br>C 1.85095200 1.34192200 -2.26565500<br>H 2.34559900 1.49206000 -3.23111400<br>H 0.95650200 0.73896800 -2.44209400<br>H 1.54107400 2.31707800 -1.89582300<br>C 4.04565700 1.51369500 -1.05625000<br>H 4.81849500 0.98612200 -0.49456500<br>H 4.48311500 1.81441700 -2.01444500<br>H 3.78664300 2.42599400 -0.51553700<br>C -0.88587300 -3.29055600 -1.01493400<br>C -0.80426100 -3.57353000 -2.49779900<br>H -0.72002500 -4.16619500 -0.38937100<br>H -0.86328200 -2.66058300 -3.09451500<br>H 0.11507500 -4.09401900 -2.77566400<br>C 1.14804700 -2.86189000 -0.64086100<br>O 1.88302300 -3.73051200 -0.79444100<br>H -1.64354700 -4.21325100 -2.79666900<br>H -1.83918700 -2.86063700 -0.73266600
**1D**<br><br>Pd 0.3063838394 -1.1544992646 0.2573880986<br>P -1.8952297739 -0.228348852 0.2708883732<br>P 1.7899384363 0.5330813166 0.2967264542<br>C -2.0617733356 1.4336791426 1.1089474332<br>C 0.9654216233 1.9581230094 1.2098485243	**S-1D**<br><br>Pd 0.2725926267 -1.168096816 -0.2855131138<br>P -1.9061162883 -0.2089437782 0.2791815634<br>P 1.7734041766 0.4994832527 0.2736468633<br>C -2.0839695747 1.4559924277 1.0994465823<br>C 0.9535332695 1.9307683784 1.179502679

C -0.0267540927 2.9023588075 0.5827980606	C -0.02746758 2.8898802117 0.5586327763
C 0.4463395926 4.1507039625 0.1640479592	C 0.4620196291 4.1327794195 0.1370732551
C -0.3942069551 5.1324490515 -0.3315980603	C -0.3678236103 5.1293094575 -0.3503819733
C -1.7585000284 4.8886309703 -0.3910426575	C -1.7377399533 4.9069086165 -0.4019070973
C -2.2472673048 3.6716041123 0.0524580883	C -2.2427343831 3.695616618 0.0428388823
C -1.409184299 2.6620500225 0.5356750808	C -1.4157690605 2.673382713 0.5233892623
H 1.5059645998 4.3652381213 0.2473199313	H 1.5267953728 4.3250285508 0.2071138417
H 0.0110793262 6.0873512507 -0.6448517324	H 0.0518038657 6.0776300686 -0.6688987278
H -2.4403431216 5.6482166355 -0.7549018474	H -2.410860171 5.6760576343 -0.7654962041
H -3.3192348947 3.5030368077 0.0479738218	H -3.3162433187 3.5362972702 0.0389771907
H 1.7892296891 2.559553259 1.5939958074	H 1.7858751229 2.5249691567 1.5552408213
H 0.5210848078 1.462090447 2.0772593129	H 0.502268273 1.4385599633 2.0454074685
H -1.6804733875 1.2538772335 2.1189640823	H -1.712428366 1.2874807723 2.1147755636
H -3.1291225093 1.6293411422 1.2239776974	H -3.1525869381 1.6561580756 1.1911215154
C 3.0420593434 -0.0141163005 1.6546815474	C 3.0125872985 -0.0485653394 1.6442650531
C 2.6466846861 1.2382638655 -1.2558832421	C 2.6510284456 1.2041609014 -1.2663156681
C -2.6566249215 -1.3626947897 1.6184270308	C -2.6514288108 -1.3382244177 1.6378441259
C -2.9720875257 -0.1822168298 -1.3047843948	C -2.9997999009 -0.1803391589 -1.2846930782
C 2.5937058754 -3.4815172011 -1.1222467409	C 2.6173688121 -3.4137309211 -1.1061121011
C 2.4689640383 -4.7929296815 -1.8878875262	C 2.571445942 -4.7173059168 -1.8899680831
H 3.2700038772 -2.7769810465 -1.6106933968	H 3.2988170272 -2.6881721998 -1.5521354778
H 3.4542583026 -5.2476007083 -1.9993118891	H 3.5784685739 -5.1336407662 -1.963090371
H 1.8228341771 -5.4979929196 -1.3646113636	H 1.9326930535 -5.455592312 -1.4016768358
C 1.298284692 -2.7810807228 -0.8772972259	C 1.292789565 -2.7590551551 -0.9185199591
O 0.2010489056 -3.2690281415 -1.0202917748	O 0.2255808726 -3.3015367547 -1.1267317965
H 2.0554075092 -4.6308543132 -2.8841321698	H 2.1971802931 -4.5567549316 -2.9034140681
H 3.0184450024 -3.6432429938 -0.123014127	H 2.990693587 -3.5783661975 -0.087387511
C 1.5434265758 1.7776289594 -2.1751950922	C 1.5693299913 1.7722788664 -2.1916679695
H 2.0102239957 2.1515579976 -3.0914562222	H 2.0545056012 2.1227911004 -3.108896111
H 0.8522106163 0.9817138944 -2.4611584835	H 0.8476219102 1.0016609414 -2.4739921712
H 0.9730911597 2.5925279936 -1.7357492954	H 1.0305785205 2.6130517811 -1.7602008806
C 3.6486553475 2.3440133305 -0.9161292959	C 3.664944833 2.2887952115 -0.8997171855
H 4.5128869079 1.9643459861 -0.369854453	H 4.5220024511 1.8803322279 -0.3624216674
H 4.0215124924 2.7792349367 -1.8480405706	H 4.0412220533 2.7420653569 -1.8231539461
H 3.2038750441 3.1547157493 -0.3389601063	H 3.2301699129 3.0867219427 -0.297106119
C 3.3566096081 0.1262591745 -2.0332987774	C 3.3500012476 0.0910693816 -2.0492538159
H 4.1958888138 -0.3109532354 -1.4970667219	H 4.1500237641 -0.3935860107 -1.4938576003
H 2.657338601 -0.664896714 -2.3078591218	H 2.6370317077 -0.6669984131 -2.3780541713
H 3.7475008043 0.5545018406 -2.9607240657	H 3.7932422571 0.5369838711 -2.9459583941
C -2.4735675709 0.9819809861 -2.1689834335	C -2.5295382182 0.9903075658 -2.1546183208
H -2.9521375578 0.9149258527 -3.1505402741	H -3.0079542594 0.906132053 -3.1363075681
H -2.7175065915 1.95510241 -1.7481546478	H -2.804448925 1.9572946279 -1.7362469321
H -1.393987465 0.9416568973 -2.3251715317	H -1.4485447102 0.9786127725 -2.312983137
C -4.4641408196 -0.0010197618 -1.0258630735	C -4.4910187726 -0.0216147483 -0.9952931719
H -4.9026715552 -0.8695199209 -0.534155786	H -4.9095586373 -0.8962585263 -0.4957888086
H -4.674920301 0.883433458 -0.4207010329	H -4.7046127125 0.8610283643 -0.3879823486
H -4.9876099326 0.1295375319 -1.9779578702	H -5.0247889938 0.0987977248 -1.944836258
C -2.7403640093 -1.4736529443 -2.0985361741	C -2.7506453486 -1.4676798361 -2.0777670088
H -1.6880836664 -1.5989261725 -2.3639410473	H -1.6933875359 -1.5802585934 -2.3330441172
H -3.0639934904 -2.3698280544 -1.5734341275	H -3.0718130885 -2.3651927109 -1.5511260977
H -3.3089287169 -1.4151157257 -3.0314615556	H -3.3146708373 -1.4147626877 -3.0152766045
C -3.0070423053 -2.7318977283 1.030008899	C -3.0065708162 -2.7089795539 1.0600324336
H -3.2578064552 -3.4117731466 1.8496904624	H -3.2429497341 -3.3864862244 1.8877311855
H -3.8750378704 -2.6899688732 0.3718274408	H -3.8813885302 -2.6717993627 0.4097184497
H -2.1736672825 -3.1748543894 0.4801663436	H -2.1755349255 -3.1493923698 0.5034793946

C -1.5624395128 -1.5656310279 2.674933266<br>H -0.6826847373 -2.0570507233 2.2539592033<br>H -1.2441212803 -0.6290492082 3.139237795<br>H -1.9524387967 -2.202207963 3.4747318535<br>C -3.8913823552 -0.7693672368 2.3051728435<br>H -4.7032504251 -0.5532245322 1.6121655581<br>H -4.2685621827 -1.4979874539 3.0292448987<br>H -3.6624492131 0.1401649246 2.8622585731<br>C 4.1872620132 -0.8278084241 1.0507990476<br>H 4.8882809226 -0.2055038094 0.4939331619<br>H 4.7485455775 -1.2959356714 1.8646188595<br>H 3.8313236875 -1.6224448347 0.3970341793<br>C 2.266438008 -0.908864083 2.6283296404<br>H 1.8805926758 -1.8039963974 2.1368380843<br>H 2.9384571786 -1.2265131127 3.4313112248<br>H 1.4280052009 -0.3900057659 3.096971806<br>C 3.6453336114 1.1555446063 2.4444106019<br>H 4.1133977367 1.9072967916 1.8083947381<br>H 2.9171946439 1.6484151916 3.0892678635<br>H 4.4257239364 0.756340315 3.0987950481	C -1.5411064147 -1.5352798736 2.6773495346<br>H -0.6784812286 -2.047521307 2.2448690267<br>H -1.1995424681 -0.593151419 3.1128737649<br>H -1.9272460845 -2.1521614317 3.4960504792<br>C -3.8770304624 -0.744942361 2.3372469012<br>H -4.7008945524 -0.5408241465 1.6536283133<br>H -4.2363387395 -1.4692300524 3.076690489<br>H -3.6411202947 0.1733077542 2.8778143691<br>C 4.1951772284 -0.8162787034 1.0554836976<br>H 4.8753331612 -0.1655554537 0.5046180744<br>H 4.760840203 -1.2559624371 1.8835157896<br>H 3.8860065734 -1.6309760577 0.403424484<br>C 2.2353928753 -0.9808401103 2.5782944846<br>H 1.8819082805 -1.8725756433 2.0557186487<br>H 2.8993841386 -1.3031017349 3.3877546046<br>H 1.3768975231 -0.484619878 3.0359566654<br>C 3.5666716804 1.1184954703 2.4707989306<br>H 4.0338388079 1.8913531925 1.8591520789<br>H 2.8086854886 1.5828629114 3.1029016294<br>H 4.3377154692 0.719395016 3.137993188
**1D-CO**<br><br>Pd -0.29623000 -1.15572800 -0.21610100<br>P 2.09141700 -0.32032100 0.26352100<br>P -1.62407200 0.74565600 0.61205500<br>C 2.34247500 1.46513200 0.77967000<br>C -0.56212000 2.20003900 1.15545700<br>C 0.33677900 2.93554200 0.19361800<br>C -0.13167800 4.09681000 -0.42680100<br>C 0.67434500 4.88450300 -1.23171800<br>C 2.00561300 4.53968000 -1.40269700<br>C 2.49836000 3.41507100 -0.76271600<br>C 1.68902400 2.58857900 0.02173100<br>H -1.15194500 4.41503400 -0.25699100<br>H 0.27031600 5.77692400 -1.69494800<br>H 2.66499100 5.15729300 -2.00103400<br>H 3.55613600 3.19419500 -0.84319100<br>H -1.24390000 2.91320900 1.62348300<br>H 0.03956800 1.78194300 1.96070100<br>H 2.00930900 1.48885900 1.81822200<br>H 3.42114200 1.63570900 0.80531500<br>C -2.32971600 0.16980300 2.30954600<br>C -2.98659900 1.51614600 -0.49681900<br>C 3.11827400 -1.21381500 1.62921400<br>C 2.92620900 -0.48298200 -1.45605300<br>C -1.85191500 -1.85745600 -1.45840400<br>C -2.87985700 -2.88309100 -1.04826500<br>H -3.41314300 -2.48909800 -0.18159000<br>H -2.31938100 -3.74146600 -0.66264700<br>O -1.70204800 -1.44406300 -2.56355600<br>C -3.82363500 -3.29282200 -2.16962400<br>H -4.40738700 -2.44512100 -2.53087300<br>H -3.27572700 -3.70354100 -3.01808900<br>H -4.51692700 -4.05355000 -1.80721900<br>C 4.22468200 0.31463000 -1.59566300	**S-1D-CO**<br><br>Pd -0.31551000 -1.14762900 -0.21642600<br>P 2.06756400 -0.35555400 0.28695500<br>P -1.63910900 0.77773800 0.55013000<br>C 2.35275100 1.41952200 0.80976400<br>C -0.55381800 2.22153800 1.07070000<br>C 0.40108800 2.92568000 0.14612100<br>C -0.02619200 4.09788700 -0.48796200<br>C 0.82565500 4.87657700 -1.25526700<br>C 2.16017400 4.51211300 -1.36877800<br>C 2.60982200 3.37672000 -0.71344700<br>C 1.75409000 2.55848300 0.03244700<br>H -1.05248200 4.42022900 -0.35736200<br>H 0.45381800 5.77546700 -1.73538100<br>H 2.85444500 5.12087400 -1.93822800<br>H 3.66601200 3.13593100 -0.75014200<br>H -1.23876200 2.96619900 1.47570200<br>H -0.00611000 1.81340500 1.92196400<br>H 1.97812700 1.45190600 1.83400200<br>H 3.43388300 1.56125700 0.87004500<br>C -2.38486000 0.35404900 2.28226900<br>C -2.93878700 1.53094100 -0.64732700<br>C 3.03864600 -1.27448600 1.67229100<br>C 2.94183600 -0.54145500 -1.41177900<br>C -1.88206300 -1.89952700 -1.36297500<br>C -2.92458100 -2.83609600 -0.81686600<br>H -3.56345700 -2.23600200 -0.16298500<br>H -2.41833700 -3.54000700 -0.14999200<br>O -1.77611900 -1.61907200 -2.52671300<br>C -3.74396800 -3.55062800 -1.87978400<br>H -4.29656000 -2.84507100 -2.50352900<br>H -3.10891300 -4.15503300 -2.53150000<br>H -4.46636900 -4.21469300 -1.39963000<br>C 4.25267000 0.23870300 -1.52065600

H 4.62542700 0.14404900 -2.59949800<br>H 4.05998400 1.38505400 -1.50124200<br>H 4.99249600 0.01497300 -0.88585500<br>C 1.90789600 0.05154600 -2.47364200<br>H 2.34863100 0.00163500 -3.47413200<br>H 0.99315800 -0.54711000 -2.49157000<br>H 1.63783800 1.09123200 -2.28395200<br>C 3.21353300 -1.95129400 -1.78103200<br>H 3.98881000 -2.38332900 -1.14772600<br>H 2.32277500 -2.57680400 -1.71754300<br>H 3.56947100 -2.01273400 -2.81358300<br>C 4.63155400 -1.05084700 1.46174700<br>H 5.12487300 -1.50154000 2.32853300<br>H 5.01397400 -1.55808200 0.57671900<br>H 4.94291200 -0.00511800 1.42777800<br>C 2.72996200 -0.63865200 2.99856400<br>H 1.65044900 -0.60908000 3.15311400<br>H 3.14824000 -1.28605900 3.77449600<br>H 3.13599000 0.35952600 3.16662400<br>C 2.76655700 -2.70253100 1.63558200<br>H 3.37167100 -3.20670500 2.39469300<br>H 1.71961400 -2.86187700 1.90005700<br>H 2.96874300 -3.19394800 0.68439600<br>C -3.06491000 -1.16239700 2.14210500<br>H -3.42700900 -1.48997200 3.12108300<br>H -3.93149100 -1.08906700 1.48639400<br>H -2.39599100 -1.93566700 1.75927700<br>C -3.26523500 1.18557100 2.96741400<br>H -2.82317800 2.17947000 3.05521600<br>H -4.21617700 1.27204000 2.44234300<br>H -3.49021900 0.84367500 3.98234700<br>C -1.13942300 -0.09014100 3.24075900<br>H -0.39051900 -0.72297800 2.75824500<br>H -0.66191200 0.82630100 3.58949500<br>H -1.49794800 -0.62007700 4.12742100<br>C -4.16256100 0.55463500 -0.67823000<br>H -4.84405600 0.98318600 -1.41883300<br>H -3.86083200 -0.41982500 -1.05483400<br>H -4.73353900 0.41673700 0.24048000<br>C -2.33453700 1.77252400 -1.86400000<br>H -3.08742700 2.20370900 -2.53081500<br>H -1.50856900 2.47971700 -1.80231400<br>H -1.96970700 0.85713300 -2.32770900<br>C -3.55396000 2.83105900 0.05050900<br>H -4.22498200 3.24810000 -0.70627300<br>H -4.13860600 2.69377500 0.95793300<br>H -2.79617300 3.58451900 0.24935200<br>C 0.24387800 -3.03057500 -0.33944200<br>O 0.45562900 -4.14341100 -0.34008000	H 4.68070600 0.04800300 -2.51094900<br>H 4.10271300 1.31281800 -1.44018700<br>H 4.99149400 -0.06483000 -0.78118200<br>C 1.95852000 -0.00441100 -2.46005100<br>H 2.43327000 -0.05301000 -3.44617500<br>H 1.04555800 -0.60531700 -2.50062700<br>H 1.67998700 1.03502900 -2.27671000<br>C 3.22477800 -2.01119600 -1.72974800<br>H 3.97949800 -2.44711300 -1.07450000<br>H 2.32865400 -2.63110100 -1.69108000<br>H 3.60832300 -2.06893800 -2.75407600<br>C 4.55717300 -1.12084600 1.56402300<br>H 5.00901300 -1.57479100 2.45308000<br>H 4.96993900 -1.63291800 0.69471700<br>H 4.87366700 -0.07611200 1.53920900<br>C 2.59549100 -0.70848700 3.02646400<br>H 1.50954400 -0.67960800 3.12513300<br>H 2.97970500 -1.36511600 3.81373800<br>H 2.99228800 0.28986700 3.21618000<br>C 2.67561200 -2.75967000 1.65311200<br>H 3.25731900 -3.27028500 2.42778900<br>H 1.61991300 -2.91346500 1.88618100<br>H 2.90203100 -3.24287700 0.70285500<br>C -3.67030000 -0.46380000 2.17168000<br>H -3.93330900 -0.81602100 3.17458300<br>H -4.50976300 0.12752500 1.80735300<br>H -3.55244400 -1.34406900 1.54052200<br>C -2.67613300 1.59520300 3.13312500<br>H -1.77180800 2.13755500 3.41076500<br>H -3.36466400 2.28950900 2.65109900<br>H -3.14783600 1.25888500 4.06256200<br>C -1.33858900 -0.49443000 3.00976500<br>H -1.08570000 -1.40004900 2.45117100<br>H -0.42296700 0.06570100 3.20318600<br>H -1.74517200 -0.79550600 3.98113900<br>C -4.07379400 0.56600500 -1.00252200<br>H -4.74559300 1.09616300 -1.68611500<br>H -3.72929700 -0.31781800 -1.53306200<br>H -4.66524800 0.25666100 -0.14423400<br>C -2.18487200 1.84101300 -1.94729700<br>H -2.90500100 2.21137300 -2.68457400<br>H -1.41773300 2.60270900 -1.82342700<br>H -1.71565700 0.94619300 -2.36105600<br>C -3.58069100 2.80033400 -0.08284300<br>H -4.17231400 3.26616600 -0.87821700<br>H -4.26315600 2.57558400 0.73869200<br>H -2.86098000 3.54152600 0.26064800<br>C 0.24595400 -3.01158000 -0.39285400<br>O 0.42645100 -4.12937300 -0.43299900
**1D-CH_3OH**<br><br>Pd 0.29655600 -1.05160900 -0.16765400<br>P 1.69768500 0.71599400 0.49747600<br>P -2.09235800 -0.21617300 0.21227800<br>C 2.89174200 1.47386600 -0.79121500	**S-1D-CH_3OH**<br><br>Pd 0.26713000 -1.05014900 -0.19432600<br>P 1.73635000 0.64354000 0.49901400<br>P -2.06886400 -0.11631000 0.24282300<br>C 3.02407300 1.32453100 -0.74925300

C	2.61021000	0.23103500	2.13319200	C	2.56522500	0.10002100	2.15846500
C	-3.10999600	-1.10309000	1.58928700	C	-3.12869300	-0.94676200	1.62316900
C	-2.97063800	-0.30593200	-1.48441800	C	-2.95851000	-0.18570600	-1.44721000
C	3.91192600	0.45482200	-1.30661200	C	4.01603100	0.26368800	-1.23241600
C	2.03101600	1.90360400	-1.98634000	C	2.23394200	1.79115400	-1.97692500
C	3.66082700	2.66821500	-0.21883500	C	3.82980500	2.47983300	-0.14941600
C	1.68551500	-0.74423500	2.87023900	C	1.55877600	-0.80851100	2.86873300
C	2.88307900	1.42788400	3.05506800	C	2.89368200	1.27241200	3.09117100
C	3.94371300	-0.47310700	1.86404300	C	3.85380600	-0.68848300	1.91822700
C	-3.56223100	-2.49669300	1.14974500	C	-3.68819800	-2.29860200	1.17954400
C	-4.34322600	-0.32563200	2.06465600	C	-4.29654600	-0.08737000	2.11859300
C	-2.14562900	-1.26439500	2.77074600	C	-2.16793500	-1.18242500	2.79244600
C	-2.80597700	-1.71503800	-2.06720100	C	-2.85477700	-1.60699300	-2.00918500
C	-4.45174300	0.06550300	-1.44672400	C	-4.42084100	0.25082500	-1.40333300
C	-2.21896100	0.65006400	-2.42063600	C	-2.18015700	0.72907900	-2.40096300
C	0.65436500	2.14480500	1.15847600	C	0.78269600	2.13886600	1.12981700
C	-2.32081300	1.54566900	0.80879500	C	-2.21660400	1.65838200	0.82186300
C	-1.62867400	2.70292800	0.14678000	C	-1.48475800	2.77425200	0.13375800
C	-2.42692600	3.61796700	-0.54784700	C	-2.25314700	3.71045300	-0.56931000
C	-1.91507700	4.78872300	-1.07944900	C	-1.69658000	4.84469600	-1.13707600
C	-0.57323900	5.08594800	-0.88985000	C	-0.33622600	5.08007800	-0.98354300
C	0.22437100	4.19807400	-0.18897100	C	0.43359400	4.17113100	-0.27585600
C	-0.26478400	2.98727300	0.31409000	C	-0.10489500	2.99990200	0.27190300
H	0.70903000	-0.30732200	3.08775400	H	0.61915300	-0.29447100	3.07692300
H	2.14349800	-1.00803700	3.82858800	H	1.98446400	-1.12004600	3.82896200
H	1.53819400	-1.66506500	2.30307200	H	1.34037500	-1.70659800	2.28626800
H	1.97405900	1.86219800	3.47186100	H	2.00241500	1.76996800	3.47528000
H	3.47681600	1.06693200	3.89986600	H	3.43172200	0.86643600	3.95444300
H	3.46024800	2.21876400	2.57472600	H	3.53997600	2.02032700	2.62965600
H	4.30495600	-0.88117300	2.81298300	H	4.15712900	-1.13386700	2.87198400
H	3.85197000	-1.30415200	1.16788800	H	3.72722400	-1.49851500	1.20116200
H	4.70771400	0.21867700	1.50782400	H	4.66935700	-0.04295500	1.58984500
H	3.01791300	3.42684300	0.22527400	H	3.21182500	3.27589900	0.26423100
H	4.39095000	2.35885800	0.53036000	H	4.50758500	2.13153300	0.63201100
H	4.21555100	3.14973700	-1.02975800	H	4.44412600	2.92120100	-0.94172300
H	4.59153200	0.09318700	-0.54038600	H	4.66327200	-0.11708800	-0.44682200
H	4.51419900	0.94628500	-2.07654200	H	4.65566300	0.73629600	-1.98542800
H	3.43611700	-0.40227500	-1.77648000	H	3.52643300	-0.57681000	-1.71815000
H	2.68996400	2.26779700	-2.78008100	H	2.94353100	2.13985100	-2.73460900
H	1.46120600	1.06207800	-2.38722200	H	1.65805100	0.97177700	-2.41402200
H	1.32918300	2.69818700	-1.74487000	H	1.55170200	2.61004900	-1.75928200
H	-1.14280100	0.45624000	-2.41286100	H	-1.10612500	0.52220800	-2.37544900
H	-2.37138600	1.69618600	-2.16592500	H	-2.32689700	1.78473000	-2.18033400
H	-2.57937500	0.49855600	-3.44279800	H	-2.53121100	0.54981500	-3.42303200
H	-3.29941600	-2.48721900	-1.47631200	H	-3.36286800	-2.35258900	-1.39871900
H	-3.25384200	-1.74094700	-3.06491500	H	-3.31457200	-1.62524900	-3.00313200
H	-1.74806400	-1.96512000	-2.18924700	H	-1.80990700	-1.90482500	-2.12997300
H	-4.62101500	1.05155200	-1.01136400	H	-4.54181900	1.24958600	-0.97914300
H	-5.04861400	-0.66086800	-0.89375900	H	-5.04159500	-0.44375800	-0.83548900
H	-4.84228100	0.09489600	-2.46884400	H	-4.81363500	0.28045200	-2.42619400
H	-2.72817200	-3.10362700	0.79648800	H	-2.91726100	-2.96453000	0.79311100
H	-4.33187600	-2.46523600	0.37793400	H	-4.47340100	-2.19886000	0.42886200
H	-3.99294100	-3.01544800	2.01130900	H	-4.13285100	-2.78912700	2.05227100
H	-5.04366300	-0.10328100	1.25976500	H	-4.98842900	0.18923900	1.32231600
H	-4.87700400	-0.93805700	2.79796100	H	-4.86085600	-0.67184300	2.85373900

H -4.08590500 0.60855500 2.56430000	H -3.96590100 0.82242100 2.62126600
H -2.66839600 -1.74749300 3.60189100	H -2.71907900 -1.63733600 3.62290600
H -1.77679100 -0.30483200 3.14132700	H -1.73120900 -0.25165600 3.16211200
H -1.28339500 -1.87961900 2.50764500	H -1.35581100 -1.85744500 2.51566500
H -3.39224600 1.74832000 0.81199700	H -3.27824100 1.90473600 0.82869800
H -2.02547500 1.49864100 1.86168300	H -1.90319800 1.61167600 1.86957000
H 0.09515800 1.67132300 1.96954400	H 0.20599300 1.71801600 1.95692100
H 1.37089400 2.81309700 1.63573200	H 1.53850400 2.78443100 1.57736400
H -3.48772800 3.41372900 -0.65067000	H -3.32247100 3.54541200 -0.65288500
H -2.56452100 5.47636700 -1.60832200	H -2.32372000 5.54889000 -1.67354600
H -0.15468000 6.01351900 -1.26236900	H 0.12105300 5.97433700 -1.39349700
H 1.25923500 4.46363300 -0.00870600	H 1.48592400 4.38224700 -0.12697100
C 1.87222400 -2.08844000 -0.87219200	C 1.74513200 -2.11946200 -1.00167800
C 1.77473900 -2.25132200 -2.38458500	C 1.51718100 -2.22644300 -2.49988600
C 2.89937200 -3.09158300 -2.97732000	C 2.16609400 -3.44968800 -3.13041900
H 0.80283200 -2.71917300 -2.57091000	H 0.43849900 -2.20638000 -2.68134100
H 1.71919000 -1.26024000 -2.84027100	H 1.90272500 -1.30155200 -2.94059800
O 2.60915200 -2.71319400 -0.17325200	O 2.53997500 -2.79009000 -0.39810400
O -0.46104800 -3.35001000 -0.35168200	O -0.47487400 -3.35779300 -0.29250300
C -0.11191800 -4.42646700 0.53204500	C -0.24185300 -4.27490500 0.77984900
H -0.15227800 -5.38042600 0.00224200	H -0.38635000 -5.30256100 0.43537600
H 0.90837600 -4.24650900 0.86077900	H 0.79261700 -4.15152400 1.09514100
H -0.77541200 -4.45594900 1.39978100	H -0.90442300 -4.07941100 1.62685800
H -1.33812000 -3.49850800 -0.71752000	H -1.35688400 -3.50614800 -0.65154700
H 3.87436700 -2.63471600 -2.80063700	H 3.24772700 -3.44694100 -2.97949500
H 2.91732000 -4.08952900 -2.53918700	H 1.76638700 -4.37342700 -2.70603900
H 2.75900700 -3.18968500 -4.05516200	H 1.97232400 -3.45826200 -4.20565900
**TS-1D_P**	**S-TS-1D_P**
Pd -0.04451600 -0.62703000 0.00948800	Pd -0.04827100 -0.63286100 -0.02239100
P 2.16996400 0.10776000 0.38581100	P 2.17867300 -0.01974300 0.37784500
P -2.10464000 0.36581900 0.34110600	P -2.03046700 0.49640200 0.36756400
C 3.36260000 0.30930500 -1.09017000	C 3.38759900 0.07311700 -1.10146400
C 3.04177600 -0.58167900 1.94988800	C 3.00626100 -0.76051300 1.94590400
C -3.14129000 -0.11144400 1.89086600	C -3.06312700 0.05336100 1.93004100
C -3.19841400 0.69241700 -1.18834600	C -3.14476600 0.90108100 -1.13280900
C 1.72406400 1.84244100 1.01474100	C 1.88709500 1.74892400 0.99317100
C -1.43427500 2.03986400 0.94659900	C -1.26860900 2.12794900 0.97538300
C -0.48386900 2.89865900 0.14721000	C -0.27825400 2.92855900 0.16520400
C -1.06350000 3.95838700 -0.56410100	C -0.80525500 4.02184300 -0.54028500
C -0.31887200 4.90331700 -1.24535600	C -0.01688300 4.91798500 -1.24095700
C 1.06548300 4.82860900 -1.20006400	C 1.36219900 4.75425000 -1.22997300
C 1.65892700 3.80903300 -0.47894500	C 1.90666500 3.69952300 -0.51836700
C 0.92454500 2.81969900 0.18889400	C 1.12594500 2.76308300 0.17680300
H -2.30961700 2.64994900 1.17700700	H -2.10542600 2.78799400 1.21167900
H -0.98604700 1.77670400 1.90757100	H -0.82088500 1.83343000 1.92729200
H 1.20404200 1.62569600 1.95098700	H 1.37939900 1.58602800 1.94652100
H 2.66088900 2.32851200 1.29252700	H 2.86445600 2.17638700 1.22567200
H -2.14378500 4.05613700 -0.55819300	H -1.87848000 4.17847100 -0.51668300
H -0.81443500 5.70652500 -1.77799700	H -0.47484000 5.74769500 -1.76910700
H 1.67799300 5.57212000 -1.69652400	H 2.00992500 5.45243600 -1.74967500
H 2.74090100 3.78537200 -0.40752900	H 2.98597600 3.59709400 -0.47720900
C 0.28521200 -3.11139700 -1.10463600	C -0.07966300 -3.12032700 -1.10289300
C -0.00275900 -2.67166400 -2.49636200	C -0.26249500 -2.59984900 -2.48439600
C -0.43922000 -3.87023700 -3.35251100	C -0.82343200 -3.70634800 -3.38860500

H	-0.76521000	-1.89427600	-2.48664400	H	-0.92728000	-1.73596700	-2.47129800
H	0.92390800	-2.23875400	-2.87567900	H	0.72181800	-2.28265100	-2.82938500
O	1.15629300	-3.69838100	-0.59103200	O	0.74853100	-3.80760200	-0.61662300
O	-1.18406800	-3.24478600	-0.34339900	O	-1.47648500	-3.12744000	-0.38100300
C	-1.17723700	-4.13348700	0.80385600	C	-1.58887200	-4.03582400	0.75701300
H	-2.16340800	-4.07100600	1.25760700	H	-2.59567100	-3.90671900	1.14406800
H	-0.99614400	-5.13781700	0.43067300	H	-1.45368800	-5.04027400	0.36575500
H	-0.39945700	-3.84131800	1.50738800	H	-0.83693000	-3.79394400	1.50396600
H	-1.28731000	-2.24735300	-0.05768300	H	-1.61139800	-2.14875900	-0.11161200
H	0.32765800	-4.64528100	-3.36847000	H	-0.15572100	-4.56941000	-3.41482400
H	-1.37178400	-4.30154000	-2.98744300	H	-1.81001800	-4.03329800	-3.05610700
C	3.97720600	0.41525100	2.64119400	C	4.08719600	0.12089900	2.57394800
C	1.92148200	-0.96444100	2.92462600	C	1.87905100	-0.95896100	2.96709500
C	3.82539900	-1.85316200	1.60995400	C	3.59670400	-2.13685600	1.62973100
C	4.60148000	1.15811600	-0.80237200	C	4.69402200	0.81638600	-0.83048600
C	3.79733700	-1.07729500	-1.58234000	C	3.68957200	-1.34991900	-1.58363000
C	2.53284700	0.94651200	-2.21333800	C	2.61837100	0.77499500	-2.22757000
C	-4.31892300	1.70620600	-0.95441000	C	-4.19181300	1.98091400	-0.86764300
C	-2.25222200	1.19307900	-2.28782000	C	-2.20119000	1.34846600	-2.25595700
C	-3.80432200	-0.62759100	-1.68170300	C	-3.83411300	-0.37759300	-1.62117000
C	-3.92113900	1.05496800	2.50850000	C	-3.76704100	1.24635200	2.58203100
C	-4.12411300	-1.23815200	1.56222800	C	-4.10261100	-1.01733700	1.59296300
C	-2.13961100	-0.63069400	2.92997900	C	-2.07573600	-0.54290800	2.94045500
H	2.36494900	-1.39512300	3.82769700	H	2.29765300	-1.41757100	3.86980000
H	1.32264400	-0.10673300	3.23745800	H	1.41090600	-0.01924800	3.26761300
H	1.24773900	-1.70294200	2.48538600	H	1.10104900	-1.62120000	2.58039800
H	4.10287100	-2.35297400	2.54307800	H	3.82919300	-2.64503000	2.57207700
H	3.24095200	-2.55996400	1.01754300	H	2.89858300	-2.77115700	1.07723300
H	4.75189900	-1.63414400	1.07905600	H	4.52568800	-2.06553900	1.06377900
H	4.46443200	-0.08671800	3.48299900	H	4.49804300	-0.39735200	3.44806900
H	4.76562900	0.78155400	1.98382900	H	4.91611900	0.31835100	1.89411100
H	3.44386400	1.27469000	3.04983100	H	3.69497100	1.07722800	2.92463100
H	3.12881700	0.95559300	-3.13114300	H	3.20995600	0.71847800	-3.14791200
H	1.62187700	0.37310300	-2.40509000	H	1.65436000	0.29426700	-2.41707600
H	2.23804500	1.97089700	-1.99671500	H	2.43238600	1.82674600	-2.01714600
H	5.15634200	1.31263700	-1.73320600	H	5.24270900	0.93365600	-1.77242600
H	4.35673600	2.14510700	-0.40612800	H	4.52932600	1.81650600	-0.42361400
H	5.28043300	0.66785700	-0.10293400	H	5.34421000	0.26852300	-0.14624400
H	4.26619200	-0.96814500	-2.56489300	H	4.17938900	-1.29292900	-2.56195000
H	4.52236400	-1.55648800	-0.92840200	H	4.35736700	-1.89166700	-0.91590400
H	2.94736500	-1.75372200	-1.69196200	H	2.77599800	-1.93777900	-1.70453000
H	-4.26008800	-0.45610900	-2.66151000	H	-4.27462500	-0.17905000	-2.60433100
H	-3.04484300	-1.40317300	-1.80727300	H	-3.12546000	-1.20172700	-1.74218000
H	-4.58172800	-1.01530800	-1.02683900	H	-4.63818900	-0.70756700	-0.96601200
H	-2.81583500	1.28986200	-3.22082700	H	-2.78744800	1.49155700	-3.17029800
H	-1.80960500	2.16069200	-2.06193900	H	-1.69153000	2.28511700	-2.03696000
H	-1.43526300	0.48691400	-2.45732200	H	-1.44098500	0.58941100	-2.46137300
H	-4.80776200	1.92824800	-1.90791700	H	-4.70358300	2.22493400	-1.80564600
H	-5.08654900	1.32073200	-0.28218900	H	-4.95272800	1.64546500	-0.16111400
H	-3.95460400	2.65220100	-0.55118000	H	-3.75079000	2.90415600	-0.48714500
H	-2.67835500	-0.89711500	3.84467200	H	-2.61460700	-0.77793500	3.86516800
H	-1.60761400	-1.51623900	2.57699700	H	-1.62807100	-1.46787800	2.57032000
H	-1.39088000	0.11629800	3.19931000	H	-1.26902400	0.14717200	3.19704100
H	-4.49869900	-1.66520400	2.49732500	H	-4.48252000	-1.44473800	2.52733200
H	-4.98983800	-0.87960000	1.00529700	H	-4.95683700	-0.60580000	1.05454900

H -3.66498900 -2.04850200 0.99049200	H -3.68260800 -1.83674700 1.00358400
H -4.53574700 0.67011700 3.32818600	H -4.38323900 0.87748200 3.40973800
H -3.26665000 1.81544200 2.93561900	H -3.06212200 1.96481400 3.00364100
H -4.59280200 1.53785100 1.79923200	H -4.42797400 1.77414200 1.89408900
H -0.60094500 -3.52794400 -4.37521700	H -0.91670900 -3.31106000 -4.40171800
**2A**	**S-2A**
Pd 0.00485200 -0.01305500 -1.01964000	Pd 0.00051200 0.11212000 -0.99372800
P -2.14667900 0.68393700 -0.62227100	P -2.15111000 0.76272200 -0.53585100
P 2.15549500 -0.71251700 -0.59395600	P 2.15370000 -0.62989000 -0.64972800
C 2.98021200 -2.23249100 -1.35157100	C 2.96308500 -2.07840600 -1.55169000
C -2.99022200 2.19396000 -1.37233700	C -2.96829400 2.34892200 -1.14313400
C 3.47971300 -1.79119300 -2.73098800	C 3.47525200 -1.51890600 -2.88117600
H 3.91762800 -2.65231400 -3.24306700	H 3.90819200 -2.33861000 -3.46267100
H 2.66759900 -1.40858900 -3.35326800	H 2.66975500 -1.07784700 -3.47467300
H 4.24948600 -1.01995600 -2.66117100	H 4.25276700 -0.76486000 -2.73856500
C 1.90727600 -3.31267100 -1.50570000	C 1.88641000 -3.13153600 -1.81443100
H 2.32828700 -4.15094000 -2.06768400	H 2.31002100 -3.90841800 -2.45867100
H 1.57717200 -3.70158400 -0.54058600	H 1.55277800 -3.61400100 -0.89394100
H 1.03769200 -2.94345700 -2.05460400	H 1.01697800 -2.70813600 -2.32393700
C 4.13453000 -2.75481500 -0.49800600	C 4.10806600 -2.68505900 -0.74522100
H 3.81675200 -3.00101000 0.51790700	H 3.77903300 -3.02673900 0.23914500
H 4.52444800 -3.67112300 -0.95114700	H 4.49608100 -3.55362900 -1.28735300
H 4.96207400 -2.04614100 -0.44825400	H 4.93474100 -1.98483900 -0.61472500
C -3.56389500 1.74210800 -2.71863500	C -3.56721500 2.02642300 -2.51443700
H -4.01235600 2.60482000 -3.21847000	H -3.96948800 2.94783800 -2.94644600
H -2.78880600 1.34394500 -3.37853300	H -2.81455600 1.63620800 -3.20555700
H -4.34393900 0.98733100 -2.60144200	H -4.38445700 1.30468100 -2.44840500
C -1.92080800 3.26487200 -1.59638800	C -1.87637500 3.40915800 -1.29327100
H -2.37301400 4.10782000 -2.12634000	H -2.32088500 4.30640100 -1.73523700
H -1.52664600 3.64968800 -0.65408400	H -1.44752800 3.69386600 -0.33061900
H -1.09027900 2.89309600 -2.20097700	H -1.06917600 3.07266900 -1.94852900
C -4.09312800 2.71889600 -0.45349100	C -4.04714900 2.83468400 -0.17723600
H -3.69344900 3.05429800 0.50568500	H -3.63279500 3.06536300 0.80719700
H -4.57240600 3.57748900 -0.93360600	H -4.48782700 3.75362900 -0.57813200
H -4.85819100 1.96791200 -0.25607700	H -4.84541400 2.10217300 -0.05219600
Fe 0.01500100 0.01199800 2.02239500	Fe -0.01629800 -0.20627700 2.04880200
C 2.00104600 -0.46642800 2.34494500	C 2.00957200 -0.59755300 2.29149100
C 0.24563000 -1.89012400 2.79252600	C 0.34157000 -2.14746500 2.64808800
C 1.17604600 -0.98742900 3.37507500	C 1.23088200 -1.24052100 3.28834500
C -0.43698500 1.91759600 1.31802400	C -0.57213400 1.77176600 1.64377700
C -1.15235900 1.07288900 3.32496800	C -1.29108500 0.55416400 3.44943100
C -0.20919600 1.94247200 2.71169200	C -0.38828800 1.57036100 3.02944300
H 2.78823600 0.25841500 2.47517200	H 2.76355800 0.15237000 2.47082500
H -0.52721300 -2.43315700 3.31429800	H -0.39452500 -2.77049800 3.13456300
H 1.24377100 -0.73390800 4.42189500	H 1.29875300 -1.05961700 4.35086800
H 0.12609400 2.46164600 0.57507300	H -0.02386800 2.46646900 1.02602100
H -1.22901200 0.86581200 4.38141000	H -1.38153000 0.15924800 4.45054200
H 0.56474800 2.50081200 3.21545000	H 0.33780400 2.07464500 3.64996700
C 0.48005900 -1.91865000 1.40043000	C 0.55661700 -2.05838200 1.25509200
C 1.60567000 -1.06150000 1.11044400	C 1.63316900 -1.12705100 1.02196800
C -1.97968300 0.51957300 2.31537600	C -2.04928100 0.13487500 2.32625100
C -1.57059600 1.05873200 1.06283500	C -1.64204400 0.91131500 1.20254700
H -0.07579800 -2.49801200 0.68018500	H 0.01892700 -2.60537300 0.49628500
H -2.80039200 -0.16313400 2.45883600	H -2.81598900 -0.62339200 2.32743700

C	3.42351500	0.62593100	-0.46233000	C	3.43791600	0.68126700	-0.43017100
N	3.17677500	1.67321700	-1.25191400	N	3.23442100	1.77593200	-1.17210200
C	4.54256200	0.54786900	0.36543500	C	4.53087000	0.53850700	0.42049000
C	4.04843000	2.67883400	-1.24644600	C	4.12436600	2.76411600	-1.08470600
C	5.44120400	1.60693900	0.36450300	C	5.44717700	1.57884700	0.50441900
H	6.32205400	1.57824600	0.99581700	H	6.30642500	1.49806800	1.16101000
C	5.19309600	2.69418700	-0.45829100	C	5.24220600	2.71511400	-0.26209400
H	5.86971100	3.53945200	-0.49303100	H	5.93089200	3.55119700	-0.22813000
H	4.71293700	-0.31474300	0.99662800	H	4.66610400	-0.36009800	1.00801700
H	3.81813500	3.51094800	-1.90464900	H	3.93116700	3.63766600	-1.70018400
C	-3.41552800	-0.64713200	-0.46671000	C	-3.40759700	-0.58849900	-0.58079800
N	-4.38031400	-0.48552800	0.44101800	N	-4.50220700	-0.46552000	0.17660600
C	-3.34100100	-1.75005600	-1.31305300	C	-3.17623600	-1.67959100	-1.41207500
C	-5.31337700	-1.43100600	0.53019000	C	-5.40550300	-1.44357700	0.12267200
C	-4.32592900	-2.72488900	-1.21484800	C	-4.12785800	-2.69062000	-1.45987400
H	-4.30489000	-3.59433800	-1.86194600	H	-3.97931100	-3.55449300	-2.09826400
C	-5.33252900	-2.56370100	-0.27635000	C	-5.26560400	-2.57118500	-0.67843000
H	-6.12078400	-3.29837000	-0.16502900	H	-6.03563600	-3.33384000	-0.68200700
H	-2.53045300	-1.83590800	-2.02660700	H	-2.26977200	-1.73113900	-2.00347800
H	-6.08314000	-1.27172500	1.27917800	H	-6.28367300	-1.31986300	0.74978800
H	0.00075500	-0.07649800	-2.54390700	H	0.00335900	0.26622100	-2.51679500
**N-2A**				**S-N-2A**			
Pd	0.05271600	0.13452200	-1.62510900	Pd	0.03773700	0.06524800	-1.64282500
P	-1.90630600	0.75146800	-0.49399900	P	-1.92215300	0.73283600	-0.57820500
P	1.96940300	-0.70882600	-0.71207900	P	1.93192400	-0.71964500	-0.63862800
C	3.19448700	-2.00780500	-1.37245900	C	3.03284900	-2.15236700	-1.24653800
C	-2.94034000	2.32312700	-0.76926500	C	-2.96632700	2.27783900	-0.98208300
C	3.87619300	-1.40488800	-2.60159800	C	3.78607000	-1.64484000	-2.47665600
H	4.48596800	-2.16870800	-3.09225800	H	4.30601700	-2.48238700	-2.95287600
H	3.14549200	-1.04535600	-3.33071900	H	3.10591700	-1.21171500	-3.21646600
H	4.54158300	-0.57722300	-2.34109800	H	4.53619300	-0.89312400	-2.21766500
C	2.29998700	-3.17723400	-1.80385100	C	2.05086500	-3.25096600	-1.66777000
H	2.91738600	-3.93563800	-2.29355900	H	2.61373100	-4.07657200	-2.11536300
H	1.80895000	-3.65185200	-0.95168300	H	1.49480800	-3.65199300	-0.81682300
H	1.53347900	-2.85834900	-2.51449300	H	1.33229000	-2.89010900	-2.40903900
C	4.22285300	-2.49920700	-0.35703600	C	4.00226300	-2.70765700	-0.20833100
H	3.75712800	-2.82518200	0.57474800	H	3.49328600	-3.00072000	0.71262900
H	4.75871200	-3.35706100	-0.77452300	H	4.48656700	-3.60155000	-0.61653300
H	4.97736000	-1.74360400	-0.13015500	H	4.79493500	-2.00064400	0.04113800
C	-3.68626100	2.14111200	-2.09309300	C	-3.74548500	1.96621300	-2.26075700
H	-4.19379100	3.07453400	-2.35359100	H	-4.24520000	2.87497900	-2.61246400
H	-3.00470900	1.89428400	-2.91230600	H	-3.08676000	1.61827000	-3.06278700
H	-4.44585000	1.35969900	-2.03069200	H	-4.51394500	1.20691300	-2.09704800
C	-1.95648000	3.48893300	-0.89763300	C	-1.97889600	3.41489200	-1.25270900
H	-2.50984800	4.40723900	-1.11620000	H	-2.53184300	4.31248300	-1.55032400
H	-1.40180000	3.65559700	0.02889300	H	-1.39377700	3.66835700	-0.36498100
H	-1.24522300	3.32195900	-1.71162700	H	-1.28482800	3.16234800	-2.05957400
C	-3.91915100	2.58808900	0.37273800	C	-3.91939800	2.67701000	0.14071500
H	-3.39733300	2.76848100	1.31464200	H	-3.37957900	2.93666300	1.05472400
H	-4.50931600	3.48091800	0.14200100	H	-4.49309500	3.55919500	-0.16644300
H	-4.60504400	1.75397100	0.52284000	H	-4.62479200	1.87900700	0.37679500
Fe	0.04548400	-0.38490200	2.14149100	Fe	-0.00490200	-0.16602600	2.17570700
C	1.97454300	-1.07713800	2.25117600	C	1.92583500	-0.84429600	2.34953900
C	0.11499100	-2.41925800	2.45226900	C	0.06377100	-2.16286400	2.66679600

C	1.15249900	-1.77440600	3.17647500	C	1.09869600	-1.45259500	3.33344500
C	-0.22767900	1.58767800	1.67477400	C	-0.24719900	1.75778300	1.50704500
C	-1.10587600	0.58614100	3.54009500	C	-1.14252500	0.95550800	3.46410800
C	-0.07159700	1.44208900	3.07743800	C	-0.09681200	1.74942500	2.91887200
H	2.83433900	-0.48044400	2.51211600	H	2.78468300	-0.22571900	2.55749900
H	-0.68396800	-3.00820700	2.87530200	H	-0.73775300	-2.71306300	3.13773400
H	1.28063600	-1.78488900	4.24812600	H	1.22332400	-1.36233400	4.40299700
H	0.40889800	2.16350900	1.02338800	H	0.40456500	2.25333600	0.80372000
H	-1.24716400	0.25396600	4.55743400	H	-1.28950200	0.72766700	4.51028000
H	0.70585200	1.88487600	3.68113300	H	0.69033100	2.23744500	3.47524800
C	0.28230400	-2.11622700	1.07823500	C	0.24232500	-1.98839700	1.26986500
C	1.45107900	-1.29483500	0.93605300	C	1.40923100	-1.18078900	1.05776600
C	-1.90830500	0.21493100	2.42914900	C	-1.94584500	0.48180900	2.39045400
C	-1.37627600	0.83629000	1.25948600	C	-1.40410000	0.98213700	1.16559200
H	-0.36642000	-2.42616600	0.27356400	H	-0.39856100	-2.38097300	0.49486500
H	-2.77248100	-0.42779200	2.44473400	H	-2.81194600	-0.15429600	2.47794000
C	3.02654100	0.79193100	-0.30188700	C	3.13526000	0.68074000	-0.33127800
N	2.49793600	1.91062200	-0.83836600	N	2.81515600	1.79975300	-1.01460300
C	4.21185200	0.92852600	0.40591400	C	4.27849400	0.72072700	0.44930600
C	3.03176200	3.13291600	-0.73693500	C	3.52358400	2.93420100	-0.99437100
C	4.80482300	2.17996900	0.53096200	C	5.04478200	1.88034800	0.49767800
H	5.73192600	2.28405900	1.08324300	H	5.93905300	1.90609600	1.10972000
C	4.21532600	3.29760300	-0.04998300	C	4.67011900	2.99885100	-0.23351100
H	4.66022000	4.28045700	0.03154200	H	5.24991900	3.91198800	-0.21413600
H	4.66744500	0.06125000	0.86085300	H	4.57343300	-0.14604600	1.02237300
H	2.49396100	3.94135800	-1.21519000	H	3.14321500	3.75249400	-1.59097200
C	-3.14939500	-0.63157000	-0.55793300	C	-3.16748000	-0.65161600	-0.49891700
N	-4.07978600	-0.73176100	0.39495800	N	-4.16558800	-0.62340000	0.39205100
C	-3.09037600	-1.50898300	-1.63974500	C	-3.02257400	-1.68872500	-1.41946000
C	-4.97974100	-1.70825000	0.29377100	C	-5.04239800	-1.62822500	0.38567900
C	-4.03696700	-2.51953000	-1.73864600	C	-3.94404500	-2.72724300	-1.42027600
H	-4.01838700	-3.21179500	-2.57285400	H	-3.85251700	-3.54408200	-2.12814600
C	-5.00444600	-2.62344800	-0.75196600	C	-4.97839900	-2.69832000	-0.49782100
H	-5.76474700	-3.39436900	-0.78536500	H	-5.72235500	-3.48549900	-0.45594600
H	-2.31475200	-1.39174200	-2.38853000	H	-2.19135000	-1.67258300	-2.11668900
H	-5.71947500	-1.75775600	1.08751400	H	-5.83773400	-1.57397300	1.12430300
H	1.57183400	1.72842500	-1.31502900	H	1.94534000	1.75158500	-1.56404100
**TS-2A_N-2A**				**S-TS-2A_N-2A**			
Pd	-0.03199200	-0.16754900	-1.34583700	Pd	-0.07788300	-0.06892600	-1.31203800
P	-2.02678500	0.75794400	-0.53033700	P	-2.05619800	0.79665400	-0.48114800
P	2.10344300	-0.69504400	-0.64023600	P	2.10301500	-0.59331200	-0.67252100
C	3.17682200	-2.04190000	-1.41701200	C	3.08945100	-1.96974500	-1.52417300
C	-2.92849400	2.35525700	-0.99090500	C	-2.87622400	2.44292800	-0.91375700
C	3.70050600	-1.47397200	-2.73899600	C	3.66647000	-1.35878900	-2.80320300
H	4.25460700	-2.25322300	-3.26956300	H	4.16225000	-2.14607600	-3.37974400
H	2.88682800	-1.14132700	-3.38812800	H	2.88554200	-0.92471500	-3.43414200
H	4.37900700	-0.63253500	-2.58410100	H	4.40741100	-0.58456100	-2.59063300
C	2.24801800	-3.22925300	-1.68965800	C	2.09080200	-3.07087800	-1.88549800
H	2.80712200	-4.00307900	-2.22305500	H	2.60374600	-3.83049600	-2.48393700
H	1.87088500	-3.67586300	-0.76733800	H	1.68774100	-3.56624100	-0.99968400
H	1.39931600	-2.94409400	-2.31752100	H	1.25730000	-2.68392200	-2.47869000
C	4.33034700	-2.48229600	-0.51729400	C	4.20229100	-2.53907300	-0.64959200
H	3.98418500	-2.80907200	0.46588700	H	3.82333900	-2.91524400	0.30375500
H	4.84034800	-3.32993400	-0.98460600	H	4.66946500	-3.37906900	-1.17502500

H 5.07473200 -1.69547700 -0.38830400<br>C -3.58040200 2.12641400 -2.35649500<br>H -4.04975300 3.05745400 -2.68594500<br>H -2.84682700 1.84000600 -3.11483000<br>H -4.35910400 1.36216600 -2.31918400<br>C -1.88589400 3.46995800 -1.09445300<br>H -2.38211900 4.38540100 -1.42904200<br>H -1.41997000 3.68491500 -0.13126500<br>H -1.10314400 3.22989800 -1.81792400<br>C -3.97583700 2.70039200 0.06665000<br>H -3.52079800 2.87360900 1.04365800<br>H -4.48997300 3.61980400 -0.22960200<br>H -4.72460200 1.91513400 0.17631900<br>Fe 0.06555100 -0.25261100 2.08139500<br>C 1.99850700 -0.90268700 2.32879300<br>C 0.14891800 -2.26017100 2.54611200<br>C 1.13943500 -1.53885800 3.26366900<br>C -0.20841300 1.68922000 1.45810000<br>C -1.00186600 0.85468600 3.44305100<br>C 0.01850900 1.65080900 2.85567800<br>H 2.82768200 -0.26044800 2.57813200<br>H -0.65623600 -2.83674800 2.97404400<br>H 1.21567200 -1.46446500 4.33760400<br>H 0.41210700 2.18188400 0.72600900<br>H -1.09378200 0.61281600 4.49100500<br>H 0.83809100 2.12146900 3.37651100<br>C 0.39400500 -2.07632500 1.16324700<br>C 1.55859600 -1.24418900 1.01247000<br>C -1.87147000 0.40974900 2.41484600<br>C -1.39949000 0.93780100 1.17482500<br>H -0.18595800 -2.51751400 0.36475200<br>H -2.74791100 -0.20562600 2.52764200<br>C 3.14357600 0.81835200 -0.40274900<br>N 2.72281600 1.88318700 -1.08983300<br>C 4.27740300 0.85288800 0.40588100<br>C 3.42131400 3.01250300 -1.00410300<br>C 4.99658100 2.03805600 0.49445300<br>H 5.88227200 2.09489400 1.11691300<br>C 4.56650100 3.13962300 -0.22762700<br>H 5.10086600 4.08107800 -0.19184100<br>H 4.59576500 -0.02076500 0.95834200<br>H 3.04674700 3.85046600 -1.58356000<br>C -3.31888000 -0.57112500 -0.49823000<br>N -4.12955000 -0.66554000 0.55730600<br>C -3.41394800 -1.41916500 -1.60121300<br>C -5.06576200 -1.61181600 0.54291900<br>C -4.39946500 -2.39805700 -1.60756500<br>H -4.50398000 -3.06798500 -2.45337200<br>C -5.24360500 -2.49837000 -0.51355600<br>H -6.02635400 -3.24598400 -0.47252300<br>H -2.73770000 -1.30392100 -2.44136300<br>H -5.70726500 -1.66020700 1.41771100<br>H 0.65074200 1.12024200 -1.74798900	H 4.98527400 -1.80556200 -0.45035000<br>C -3.52119300 2.26257800 -2.28959100<br>H -3.93039400 3.22345900 -2.61635200<br>H -2.79407000 1.93784500 -3.03948200<br>H -4.34110700 1.54110300 -2.26839400<br>C -1.78828600 3.51417400 -0.98993700<br>H -2.24850100 4.45470900 -1.30921200<br>H -1.31626400 3.68997700 -0.02137200<br>H -1.01056100 3.25649500 -1.71315500<br>C -3.92114800 2.83438600 0.12933000<br>H -3.47747400 2.95585700 1.12045500<br>H -4.36198900 3.79489500 -0.15786000<br>H -4.72524200 2.10087700 0.19974100<br>Fe 0.00888100 -0.35248200 2.10441700<br>C 1.99424200 -0.87350300 2.29238700<br>C 0.24716500 -2.36598500 2.47223700<br>C 1.19183500 -1.60302500 3.21058400<br>C -0.37770400 1.63328900 1.68685400<br>C -1.19548700 0.51202600 3.51745700<br>C -0.21080700 1.44076700 3.08121900<br>H 2.78138900 -0.18706600 2.56027700<br>H -0.51323700 -3.01347800 2.88357800<br>H 1.27345800 -1.56029800 4.28690500<br>H 0.23420300 2.26142200 1.05775400<br>H -1.31235400 0.13400400 4.52274100<br>H 0.55343300 1.89510700 3.69467800<br>C 0.46530200 -2.11248600 1.09533300<br>C 1.57014800 -1.20276700 0.96842400<br>C -1.98279700 0.13791500 2.39657100<br>C -1.50066300 0.85017600 1.25593800<br>H -0.09973000 -2.54151800 0.28007400<br>H -2.80840200 -0.55538200 2.40426400<br>C 3.28303000 0.80178300 -0.37553100<br>N 3.05210200 1.89838600 -1.10474000<br>C 4.35420000 0.70303900 0.50972500<br>C 3.88225300 2.93181300 -0.96757200<br>C 5.20880700 1.78897500 0.64686600<br>H 6.04811300 1.73957400 1.33184400<br>C 4.97063000 2.92808500 -0.10539200<br>H 5.61155600 3.79880400 -0.03171600<br>H 4.52378100 -0.19915200 1.08141800<br>H 3.66438500 3.80454800 -1.57616200<br>C -3.37109800 -0.50462100 -0.56084400<br>N -4.37898600 -0.48018900 0.31663700<br>C -3.26162500 -1.46744800 -1.56107500<br>C -5.31426700 -1.42429400 0.21440900<br>C -4.24405700 -2.44440300 -1.65752400<br>H -4.18813300 -3.20654700 -2.42700800<br>C -5.29331400 -2.42248900 -0.75288500<br>H -6.08458900 -3.16225900 -0.78746500<br>H -2.42172200 -1.44527700 -2.24780000<br>H -6.12087300 -1.37995500 0.94072300<br>H 0.37216000 1.32117100 -1.66391600
**2B**	**S-2B**

Pd	0.08860100	-1.75436000	-0.01491800	Pd	-0.03008300	-1.75111500	0.01378000
P	-1.78733600	-0.51500800	-0.61005800	P	-1.75650000	-0.36967300	-0.69063700
P	1.70701100	-0.09338700	0.78380000	P	1.79265400	-0.27279500	0.70194100
C	2.46592500	-0.46395400	2.49451100	C	2.67332400	-0.90668200	2.27258600
C	-3.03146800	-1.19534100	-1.88763200	C	-2.77851800	-0.89372700	-2.21658800
C	-2.80199400	-0.15198400	0.90187500	C	-2.98445300	-0.16020100	0.68630500
C	3.11093600	-0.22700700	-0.42098500	C	3.10553800	-0.21433300	-0.60819100
N	-3.67681900	0.85342300	0.84291100	N	-3.97952200	0.71597800	0.51792100
N	2.95391100	-1.24803300	-1.27245600	N	2.89756300	-1.09232900	-1.59804600
C	4.23201300	0.59968200	-0.44987200	C	4.23042400	0.60734100	-0.57007800
C	3.90488500	-1.46946000	-2.17823700	C	3.79918200	-1.17002400	-2.57705700
C	5.21542000	0.36082400	-1.40058200	C	5.15883400	0.52122900	-1.59887000
H	6.09794400	0.98895500	-1.44482800	H	6.04077300	1.15245200	-1.59181600
C	5.05148400	-0.69321300	-2.28542600	C	4.94238500	-0.38467200	-2.62520000
H	5.79361100	-0.91480900	-3.04267100	H	5.64109400	-0.48594600	-3.44742200
H	4.34235100	1.41193600	0.25528000	H	4.39064600	1.29865300	0.24562000
H	3.73958000	-2.30660400	-2.84955000	H	3.59512100	-1.89487000	-3.35974800
C	-2.65596600	-0.95716300	2.02849000	C	-2.85423500	-0.93771100	1.83342400
C	-4.43354200	1.08993400	1.91233400	C	-4.87429100	0.84403300	1.49700000
C	-3.45698300	-0.70768300	3.13537000	C	-3.79488400	-0.79702500	2.84601600
H	-3.37076200	-1.31995700	4.02584000	H	-3.71898600	-1.38978600	3.75099200
C	-4.36431400	0.33814100	3.07942400	C	-4.82624900	0.11240600	2.67721800
H	-5.00871200	0.57144800	3.91825300	H	-5.58297200	0.25734000	3.43937200
H	-1.92696800	-1.75841300	2.02934700	H	-2.03151400	-1.63769500	1.92380200
H	-5.13114400	1.91785000	1.82879100	H	-5.66863200	1.56582000	1.32905100
C	1.31465800	-0.47969600	3.50382400	C	1.56974500	-1.28727800	3.26292800
H	1.70084300	-0.77570200	4.48355700	H	2.02626500	-1.69142700	4.17242300
H	0.85586300	0.50383300	3.61814300	H	0.96633400	-0.42382400	3.55356500
H	0.53880500	-1.19555200	3.22089900	H	0.90209100	-2.04948100	2.85246800
C	3.49650900	0.58581500	2.90878300	C	3.58133800	0.14519800	2.90585800
H	3.80781600	0.39216000	3.93981700	H	4.01383400	-0.26970700	3.82280800
H	4.39516100	0.54269400	2.29186500	H	4.41166300	0.42578700	2.25531200
H	3.09068300	1.59861200	2.87195400	H	3.03330600	1.04930300	3.17939400
C	3.12030900	-1.84516100	2.44375400	C	3.48898200	-2.14582800	1.90592300
H	3.91839100	-1.89828400	1.70047300	H	4.32462900	-1.90510500	1.24500100
H	3.56200300	-2.06847900	3.41912700	H	3.90273300	-2.58277800	2.82046800
H	2.39449500	-2.63299900	2.23063900	H	2.88002800	-2.91344700	1.42179400
C	-3.77590400	-2.38575600	-1.28306600	C	-3.77488200	-1.98017400	-1.81038200
H	-4.46656400	-2.78627000	-2.03051200	H	-4.28302100	-2.34189500	-2.71024600
H	-3.10782500	-3.19362600	-0.98896500	H	-3.29745800	-2.83670800	-1.33558800
H	-4.36954200	-2.09361300	-0.41464700	H	-4.53953600	-1.59444200	-1.13274500
C	-2.24073000	-1.61063200	-3.12974600	C	-1.79865900	-1.42187900	-3.26608200
H	-2.93247900	-2.02106500	-3.87081100	H	-2.36010400	-1.70265400	-4.16326300
H	-1.73815700	-0.75713300	-3.58895900	H	-1.06938200	-0.66199800	-3.55802800
H	-1.49107200	-2.37214400	-2.91363800	H	-1.25398600	-2.30193600	-2.92011000
C	-4.04106100	-0.11100400	-2.27532600	C	-3.54397700	0.29687200	-2.79704100
H	-4.76734500	-0.55104500	-2.96557800	H	-4.15427200	-0.06252800	-3.63258400
H	-4.58451500	0.27669200	-1.41366100	H	-4.21042700	0.75060900	-2.06231700
H	-3.56456300	0.72607800	-2.78591000	H	-2.87474200	1.06747100	-3.18329500
Fe	-0.04063000	2.44462000	-0.51045200	Fe	0.05664800	2.49352900	-0.07045100
C	1.83688300	2.80068000	0.18617700	C	1.94253500	2.69343500	0.66975400
C	-0.07318100	3.51852300	1.25081200	C	0.04024500	3.18563400	1.87241100
C	1.01865000	3.93206600	0.44394900	C	1.12900900	3.75220900	1.15644900
C	-0.10554700	1.21850900	-2.13300200	C	-0.03376500	1.57991200	-1.89075200
C	-1.15320200	3.26255700	-2.04079800	C	-1.03352900	3.59741700	-1.42196700

C	-0.03271800	2.56604600	-2.56805600	C	0.07154100	2.98369200	-2.07209200
H	2.71797300	2.79866600	-0.43363100	H	2.82503300	2.81737700	0.06353100
H	-0.88375000	4.14331700	1.59247700	H	-0.76559100	3.72970400	2.34314300
H	1.18551800	4.92754700	0.06204200	H	1.29761700	4.80516200	0.98238600
H	0.61066800	0.43858800	-2.34342900	H	0.66740200	0.83939000	-2.24522800
H	-1.36398700	4.31335000	-2.16913200	H	-1.22351100	4.65902400	-1.35585700
H	0.75568600	2.99266100	-3.16880900	H	0.87183000	3.49448600	-2.58750500
C	0.05926600	2.12800400	1.49310000	C	0.17142900	1.77344700	1.82742300
C	1.25335600	1.66640800	0.84203400	C	1.35681800	1.45257100	1.08395200
C	-1.93031000	2.35276800	-1.27914100	C	-1.82998400	2.57708100	-0.83687000
C	-1.28486700	1.07352700	-1.32733700	C	-1.21444200	1.31404700	-1.12247000
H	-0.63909300	1.51486000	2.04012400	H	-0.51965700	1.05937700	2.24897800
H	-2.83327400	2.56596300	-0.73207500	H	-2.73520800	2.72019000	-0.26998500
H	1.37683800	-3.12720200	0.27143300	H	1.02777600	-3.28052700	0.43711300
C	-0.63169200	-3.64240200	-0.44886300	C	-1.03825300	-3.53286500	-0.26628000
C	0.72777000	-4.04822800	-0.02627000	C	0.23057900	-4.10605900	0.22879400
H	1.33390400	-4.48025600	-0.82155600	H	0.75724600	-4.72155000	-0.50113500
H	-1.44342000	-3.92507500	0.21513400	H	-1.89904500	-3.61582800	0.39148600
H	-0.87503100	-3.78961000	-1.49517700	H	-1.27469000	-3.72638100	-1.30745900
H	0.75856700	-4.65754800	0.87637400	H	0.15895800	-4.59901500	1.19806200
**2C**				**S-2C**			
Pd	0.20243200	-1.53831600	-0.69364200	Pd	0.18302900	-1.55448000	-0.61109300
P	-1.95764500	-0.25026900	-0.41835600	P	-1.94534200	-0.20159400	-0.45153200
P	1.68544300	-0.03852500	0.53711100	P	1.70764600	-0.04776700	0.54309000
C	1.55609500	-0.18139900	2.43108000	C	1.60975600	-0.22197800	2.43620700
C	-3.34983400	-0.65641000	-1.68237900	C	-3.21218200	-0.56548600	-1.85023900
C	-2.77789100	-0.72414800	1.18184600	C	-2.88752700	-0.69849200	1.07077300
C	3.51527500	-0.24226900	0.23189000	C	3.53027900	-0.25152400	0.18140200
N	-3.69761700	0.09908300	1.68292700	N	-3.89712000	0.07530100	1.47772400
N	4.04889700	-1.36276900	0.72508100	N	4.10187300	-1.34995200	0.68576900
C	4.27376700	0.71413500	-0.43816200	C	4.24918600	0.68379200	-0.55980000
C	5.35466000	-1.56783300	0.56991400	C	5.40183100	-1.54844400	0.47214800
C	5.63540300	0.48830300	-0.59505200	C	5.60343000	0.46539200	-0.77632100
H	6.25169500	1.21767800	-1.10831300	H	6.18398400	1.18204700	-1.34702100
C	6.19154500	-0.67292400	-0.08387400	C	6.19702000	-0.67172900	-0.25232600
H	7.24896900	-0.88493600	-0.18375100	H	7.25061500	-0.87991500	-0.39694100
H	3.82067300	1.61789300	-0.82069900	H	3.77310500	1.56849300	-0.95815500
H	5.74549000	-2.48969700	0.98974900	H	5.82483700	-2.45175400	0.90256900
C	-2.46969400	-1.95283100	1.76317500	C	-2.55295100	-1.89354700	1.70244300
C	-4.34056900	-0.27473700	2.78735900	C	-4.60560900	-0.32433700	2.53351100
C	-3.15259900	-2.33793500	2.90869300	C	-3.30197700	-2.30215000	2.79803300
H	-2.93966100	-3.28978100	3.38175800	H	-3.06568000	-3.22864300	3.30992000
C	-4.10758800	-1.48220700	3.43510400	C	-4.35012600	-1.50222400	3.22475200
H	-4.66310700	-1.73898600	4.32889200	H	-4.96032300	-1.77689500	4.07726600
H	-1.71149000	-2.59285900	1.32637400	H	-1.72068600	-2.48717400	1.34074200
H	-5.07958800	0.42424400	3.16724400	H	-5.41787300	0.32851500	2.84048300
C	0.24345600	0.44494300	2.89671300	C	0.31396100	0.41535700	2.93162600
H	0.13989600	0.27158500	3.97180300	H	0.20559600	0.18620000	3.99657400
H	0.20484300	1.51923200	2.72502400	H	0.31479500	1.49899200	2.82326800
H	-0.61596200	-0.01404900	2.40846600	H	-0.55962500	0.01188800	2.41853200
C	2.73847500	0.50184700	3.12364000	C	2.80620400	0.42955800	3.13146300
H	2.57540800	0.45914300	4.20461600	H	2.65599900	0.35297500	4.21343200
H	3.68046900	-0.00232400	2.91282900	H	3.74395700	-0.07195700	2.89184700
H	2.84274800	1.55186500	2.84778700	H	2.90803300	1.48821200	2.88687800

C 1.54883100 -1.67539600 2.77604400<br>H 2.46680300 -2.16786500 2.45698500<br>H 1.45778100 -1.78189200 3.86115300<br>H 0.70058700 -2.18787000 2.31594200<br>C -3.91148100 -2.04202400 -1.34947200<br>H -4.57953700 -2.35117700 -2.15810600<br>H -3.14455000 -2.81193900 -1.25515200<br>H -4.49328000 -2.03155000 -0.42753500<br>C -2.76627800 -0.66501200 -3.09730100<br>H -3.56220400 -0.90860800 -3.80681700<br>H -2.36101000 0.30325600 -3.39159000<br>H -1.97902100 -1.40980300 -3.22241900<br>C -4.48542900 0.36547300 -1.58541800<br>H -5.31193800 0.02454300 -2.21653000<br>H -4.85663400 0.46511300 -0.56464000<br>H -4.18712700 1.35307700 -1.93455500<br>Fe -0.23119100 2.76308300 -0.38725200<br>C 1.36386000 2.02565500 -1.38797600<br>C 1.41771600 3.98444600 -0.18609400<br>C 1.35396000 3.44085300 -1.49641900<br>C -1.86321600 2.42729600 -1.53722200<br>C -1.88102700 3.77365600 0.32347800<br>C -1.86320700 3.77623100 -1.09488700<br>H 1.31714500 1.32336800 -2.20688300<br>H 1.38487500 5.03277400 0.06842700<br>H 1.27216100 4.00291000 -2.41404100<br>H -1.82505000 2.11579000 -2.56786500<br>H -1.86021900 4.64413900 0.96107700<br>H -1.82237500 4.64688100 -1.73128300<br>C 1.47946800 2.91225600 0.74000200<br>C 1.44959900 1.68273300 0.00567700<br>C -1.89962000 2.42671500 0.76474500<br>C -1.90235700 1.57527600 -0.38526200<br>H 1.53019400 3.02108000 1.81050000<br>H -1.93481100 2.10098700 1.78927800<br>C 1.91208200 -2.78581700 -1.06349000<br>C -0.57484300 -3.00155900 -1.69038300<br>O -0.92445000 -3.90633600 -2.27382300<br>C 2.57312900 -2.39458300 -2.36512100<br>H 1.92771700 -2.54630800 -3.23304600<br>H 2.89974400 -1.35287500 -2.37083600<br>H 3.46712500 -3.01094700 -2.52201400<br>H 1.58974700 -3.82651000 -1.06878900<br>H 2.55215300 -2.64123800 -0.19887200	C 1.57549900 -1.72141000 2.75195600<br>H 2.48916300 -2.22469700 2.43556200<br>H 1.47224700 -1.84452600 3.83505500<br>H 0.72477800 -2.21266600 2.27201800<br>C -3.86328700 -1.92299700 -1.57080200<br>H -4.43890100 -2.21637200 -2.45434900<br>H -3.14801700 -2.72032600 -1.37173700<br>H -4.55385600 -1.87193300 -0.72708100<br>C -2.45773900 -0.61271300 -3.18114200<br>H -3.17316400 -0.80233200 -3.98794600<br>H -1.94855900 0.32644500 -3.40643600<br>H -1.71286100 -1.41153900 -3.20331700<br>C -4.32542600 0.48252700 -1.90983500<br>H -5.08686400 0.12940300 -2.61349200<br>H -4.80550100 0.62104800 -0.93934800<br>H -3.97566400 1.45120800 -2.26422800<br>Fe -0.20357100 2.79090900 -0.30254400<br>C 1.33439000 2.01546500 -1.36390200<br>C 1.47638100 3.97918700 -0.17610800<br>C 1.34674400 3.42991400 -1.48055500<br>C -1.88048300 2.55800400 -1.41545100<br>C -1.81142800 3.76484600 0.54134500<br>C -1.84425300 3.87144800 -0.87449500<br>H 1.23632500 1.30729600 -2.17375900<br>H 1.47301100 5.03023700 0.07412600<br>H 1.23401000 3.98868000 -2.39809400<br>H -1.88251800 2.32046900 -2.46686400<br>H -1.75228700 4.58549200 1.24145900<br>H -1.81191000 4.78660500 -1.44758900<br>C 1.55944400 2.90972100 0.75309500<br>C 1.47465100 1.67947300 0.02624300<br>C -1.83111300 2.38736900 0.88115800<br>C -1.88961300 1.62359900 -0.32955400<br>H 1.65686400 3.01678700 1.82146800<br>H -1.81706100 1.98756000 1.88116400<br>C 1.86378900 -2.83084500 -0.97177500<br>C -0.64860800 -3.10959500 -1.39217400<br>O -1.02244500 -4.09024100 -1.81963400<br>C 2.44274400 -2.49573400 -2.32633600<br>H 1.73726000 -2.68733400 -3.13923100<br>H 2.75759700 -1.45184300 -2.39819300<br>H 3.33033900 -3.11505800 -2.51636400<br>H 1.55049100 -3.87332500 -0.91268800<br>H 2.55567500 -2.64760800 -0.15589900
**TS-2C_2D**<br><br>Pd 0.18061900 -1.50383100 -0.54311500<br>P -1.91864300 -0.33202600 -0.40452600<br>P 1.64966000 0.12774300 0.59497100<br>C 1.59598300 0.15384200 2.49831800<br>C -3.27569200 -0.90055800 -1.63214400<br>C -2.70702800 -0.69714900 1.23894800<br>C 3.45293100 -0.17403700 0.25248100<br>N -3.66433600 0.12522400 1.66502100<br>N 3.87976700 -1.39559800 0.58629800	**S-TS-2C_2D**<br><br>Pd 0.17429800 -1.49640800 -0.55821700<br>P -1.92108400 -0.31811900 -0.41177100<br>P 1.64160100 0.11564100 0.61440500<br>C 1.58841400 0.14708600 2.51806000<br>C -3.26114000 -0.87386700 -1.66319100<br>C -2.71515100 -0.72410700 1.21732800<br>C 3.44543300 -0.18847700 0.26775000<br>N -3.69319200 0.06409400 1.66823400<br>N 3.88975200 -1.39735000 0.62888500

C	4.29757200	0.79312800	-0.28719900	C	4.27452600	0.76398200	-0.32113900
C	5.16745100	-1.68802400	0.40939000	C	5.17319700	-1.69145500	0.41994500
C	5.63621700	0.47420200	-0.47135100	C	5.60843300	0.44465100	-0.53505800
H	6.31975500	1.20774200	-0.88375000	H	6.27617600	1.16989100	-0.98743400
C	6.08532200	-0.78790300	-0.11411900	C	6.07207800	-0.80667900	-0.15826900
H	7.12248100	-1.07411800	-0.23836700	H	7.10552600	-1.09643700	-0.30769000
H	3.92215500	1.77332700	-0.54795200	H	3.89396900	1.73725400	-0.60003500
H	5.47383100	-2.68860600	0.69910100	H	5.49388300	-2.68246900	0.72866000
C	-2.34116400	-1.85058000	1.92958400	C	-2.30992400	-1.87887500	1.88249300
C	-4.28911700	-0.17388700	2.80227300	C	-4.29320800	-0.27450600	2.80959400
C	-3.00484100	-2.15862700	3.10910300	C	-2.94866800	-2.22607100	3.06494200
H	-2.74809900	-3.05210600	3.66670200	H	-2.65674900	-3.12050300	3.60429300
C	-3.99884200	-1.30353900	3.55825500	C	-3.95962600	-1.40645900	3.54222800
H	-4.54168700	-1.50226800	4.47432800	H	-4.48314800	-1.63280600	4.46379800
H	-1.55745000	-2.49380900	1.54620500	H	-1.51122500	-2.48913400	1.47549800
H	-5.06057600	0.52168800	3.11835900	H	-5.08077600	0.39062000	3.15229300
C	0.28110100	0.77753600	2.96478100	C	0.28750500	0.79777200	2.98296500
H	0.22309500	0.69204200	4.05390400	H	0.20626800	0.66764500	4.06710900
H	0.19646300	1.83226900	2.70863800	H	0.25476500	1.86619800	2.77423200
H	-0.57959900	0.25196400	2.55006400	H	-0.58473000	0.32632300	2.52852400
C	2.78724400	0.91987500	3.07708800	C	2.78909200	0.89197000	3.10181800
H	2.68267200	0.96026800	4.16548100	H	2.67796000	0.92908200	4.19072100
H	3.73409000	0.42607200	2.85701000	H	3.73135300	0.38579500	2.88480200
H	2.84784500	1.94646800	2.71365700	H	2.86051600	1.91962800	2.74188800
C	1.64512500	-1.30550000	2.96544800	C	1.60588300	-1.31090600	2.98849900
H	2.54716200	-1.81425600	2.62562000	H	2.51022600	-1.83291000	2.67417800
H	1.63011500	-1.32451200	4.05929700	H	1.56802800	-1.32215700	4.08278400
H	0.77804200	-1.86729200	2.60991900	H	0.74012000	-1.86486600	2.61745900
C	-3.74737600	-2.29209500	-1.20058000	C	-3.72941500	-2.27260000	-1.25329800
H	-4.41976300	-2.68387900	-1.96863500	H	-4.36955700	-2.66919200	-2.04741600
H	-2.92951500	-3.00562200	-1.09543700	H	-2.90534800	-2.97410400	-1.11651900
H	-4.30222300	-2.26125400	-0.26244600	H	-4.31692400	-2.25063900	-0.33370700
C	-2.69260700	-0.97521800	-3.04547200	C	-2.65776800	-0.92450900	-3.06825400
H	-3.47553400	-1.31634200	-3.72870000	H	-3.42844000	-1.26859400	-3.76552400
H	-2.34964400	-0.00759500	-3.41185400	H	-2.31942800	0.05279700	-3.41467300
H	-1.86514600	-1.68126800	-3.11943900	H	-1.81799800	-1.61755600	-3.13641900
C	-4.46464600	0.06360500	-1.60026100	C	-4.46222500	0.07308700	-1.64493400
H	-5.26529100	-0.35671800	-2.21639800	H	-5.23938400	-0.34541200	-2.29324400
H	-4.85176500	0.20245900	-0.59011600	H	-4.88390600	0.17919700	-0.64377300
H	-4.21481400	1.04415400	-2.00431300	H	-4.21583900	1.06594900	-2.02232200
Fe	-0.28768800	2.75868900	-0.58962400	Fe	-0.29457500	2.76211700	-0.55843400
C	1.38090200	2.03667100	-1.48136500	C	1.33572000	1.98769400	-1.48450400
C	1.28493400	4.08134200	-0.43743700	C	1.30595300	4.05623200	-0.48278500
C	1.29797600	3.43561700	-1.70187100	C	1.26847400	3.38283400	-1.73369400
C	-1.87483400	2.24538900	-1.74338700	C	-1.93378000	2.30890900	-1.67038800
C	-2.00163200	3.73707000	-0.00108300	C	-1.96947300	3.73835900	0.13102000
C	-1.94955300	3.62399700	-1.41437000	C	-1.97485900	3.67638500	-1.28790100
H	1.40145000	1.27294400	-2.24441000	H	1.31897200	1.20504500	-2.22898400
H	1.18937300	5.14284000	-0.26718000	H	1.23160700	5.12355300	-0.33230200
H	1.21890100	3.91938000	-2.66327900	H	1.16460500	3.84672900	-2.70373000
H	-1.79859600	1.85263600	-2.74359700	H	-1.90337200	1.94950800	-2.68619900
H	-2.03558900	4.65649800	0.56292200	H	-1.96623700	4.63716300	0.73041000
H	-1.93388300	4.44070900	-2.11958300	H	-1.97370300	4.51905400	-1.96384400
C	1.37417900	3.08870800	0.57225900	C	1.41139300	3.08325800	0.54584900
C	1.43751600	1.80741200	-0.06325700	C	1.43364700	1.78967400	-0.06458900

C -1.96600700 2.43178600 0.55032800<br>C -1.90346100 1.48938800 -0.52563100<br>H 1.37740000 3.28445600 1.63144400<br>H -2.00898700 2.19058700 1.59779000<br>C 1.75806100 -3.12350000 -1.03794000<br>C -0.24140300 -3.09020700 -1.47500400<br>O -0.69188600 -3.95826500 -2.07313400<br>C 2.43292000 -2.94505100 -2.37742800<br>H 1.85156000 -3.35594600 -3.20481000<br>H 2.64179800 -1.89664300 -2.59449600<br>H 3.39315800 -3.47203600 -2.36615100<br>H 1.55213500 -4.16183600 -0.78465600<br>H 2.32456600 -2.69124400 -0.21234100	C -1.92882000 2.41108700 0.63163500<br>C -1.92401800 1.50881900 -0.48116300<br>H 1.45361000 3.29655600 1.60174600<br>H -1.91420700 2.13100500 1.67089900<br>C 1.76458600 -3.06395500 -1.05715900<br>C -0.25274700 -3.05301600 -1.51971600<br>O -0.67981200 -3.92314200 -2.13530000<br>C 2.47280400 -2.78061700 -2.35840900<br>H 1.90575000 -3.12175500 -3.22805500<br>H 2.68369700 -1.71759000 -2.48914300<br>H 3.43290000 -3.31103700 -2.36881000<br>H 1.55583600 -4.12010500 -0.89435900<br>H 2.30339300 -2.70108400 -0.18281800
**2D**<br><br>Pd -0.20979400 1.42213300 0.10283000<br>P 1.82057400 0.31430100 -0.18556600<br>P -1.66664700 -0.48895300 0.83159600<br>C -2.45423900 -0.84119300 2.52638200<br>C 3.44305800 1.07871900 -0.82875400<br>C 2.30967200 -0.50654800 1.40446900<br>C -2.91574200 0.56026900 -0.05278100<br>N 2.80919400 -1.74159300 1.36513900<br>N -2.35826300 1.77666500 -0.24646700<br>C -4.20644600 0.28182900 -0.47568100<br>C -3.06270200 2.74100000 -0.83869600<br>C -4.94227200 1.28975300 -1.08951500<br>H -5.95532100 1.09954400 -1.42504500<br>C -4.36641700 2.53832700 -1.26668200<br>H -4.90908200 3.34622500 -1.74117100<br>H -4.62898800 -0.70361100 -0.33097700<br>H -2.56272600 3.69065600 -0.98534200<br>C 2.18583000 0.21819300 2.58888200<br>C 3.20577500 -2.29412600 2.51057400<br>C 2.62322900 -0.35850700 3.77320000<br>H 2.55287500 0.18476900 4.70878100<br>C 3.14359400 -1.64313300 3.73645000<br>H 3.49405200 -2.13570700 4.63526500<br>H 1.74797400 1.20925200 2.57818900<br>H 3.60131800 -3.30307000 2.44078300<br>C -1.41410800 -1.58793100 3.36626000<br>H -1.79420900 -1.69636700 4.38623200<br>H -1.22577100 -2.59185000 2.98354100<br>H -0.46573900 -1.04884900 3.41886300<br>C -3.72742600 -1.67593900 2.41371200<br>H -4.08242100 -1.92680100 3.41825900<br>H -4.53227200 -1.13099200 1.91799000<br>H -3.55604300 -2.61292200 1.88023800<br>C -2.74941700 0.51729500 3.16679200<br>H -3.51336400 1.07388300 2.61995000<br>H -3.12113200 0.36162400 4.18351300<br>H -1.85270800 1.13872300 3.23320200<br>C 3.80163400 2.28002800 0.04650000<br>H 4.75075700 2.69807500 -0.30024500<br>H 3.05468600 3.07182900 -0.01991200	**S-2D**<br><br>Pd -0.29136300 1.40594000 0.09528800<br>P 1.79737000 0.41108000 -0.16765900<br>P -1.61973800 -0.58224600 0.81354100<br>C -2.35040500 -0.98814200 2.51946500<br>C 3.39809900 1.25444100 -0.76944000<br>C 2.29605500 -0.39401300 1.42631000<br>C -2.93540500 0.39818700 -0.04540500<br>N 2.79820700 -1.62981900 1.40581200<br>N -2.44426100 1.64499800 -0.22641200<br>C -4.21548000 0.05677900 -0.44857600<br>C -3.21026400 2.58565500 -0.77957700<br>C -5.01367100 1.03758900 -1.02724100<br>H -6.02182300 0.80006900 -1.34777600<br>C -4.50957900 2.31930200 -1.18483000<br>H -5.10516600 3.10849800 -1.62710300<br>H -4.58337800 -0.95195500 -0.31508900<br>H -2.76950900 3.56718800 -0.90579800<br>C 2.16619100 0.34758200 2.59928400<br>C 3.18632800 -2.16689100 2.56348800<br>C 2.59786400 -0.21274000 3.79324400<br>H 2.51853100 0.34423200 4.72032700<br>C 3.11661700 -1.49854000 3.77839400<br>H 3.45782500 -1.98125000 4.68668100<br>H 1.72930100 1.33880500 2.57825500<br>H 3.57881700 -3.17863100 2.51343100<br>C -1.23141500 -1.59084000 3.37082200<br>H -1.60299900 -1.72994000 4.39115400<br>H -0.91391900 -2.56790300 3.00285800<br>H -0.35888200 -0.93600400 3.41780100<br>C -3.51938500 -1.96397900 2.42624000<br>H -3.86112300 -2.20355300 3.43914100<br>H -4.36884400 -1.54152300 1.88677300<br>H -3.23108500 -2.90079800 1.94381400<br>C -2.79785900 0.33903100 3.13463900<br>H -3.63180900 0.78689900 2.58961800<br>H -3.13088700 0.15717000 4.16120600<br>H -1.98135500 1.06590100 3.17377300<br>C 3.67938300 2.46948100 0.11305800<br>H 4.61901700 2.92672500 -0.21287200<br>H 2.89896700 3.22674100 0.03088200

H	3.93222000	2.00644300	1.09621300	H	3.79405100	2.20041800	1.16576700
C	3.22385700	1.52456800	-2.27712200	C	3.19517500	1.69390300	-2.22021300
H	4.12682200	2.03763300	-2.62105900	H	4.07672500	2.26084400	-2.53667200
H	3.05782800	0.67349800	-2.93871800	H	3.09258600	0.83981100	-2.89220800
H	2.38765400	2.21571100	-2.38497900	H	2.32253600	2.33718400	-2.34067000
C	4.57140700	0.04698800	-0.77559500	C	4.57402800	0.28107100	-0.68991300
H	5.46625400	0.49202700	-1.22132800	H	5.45602200	0.77269200	-1.11420600
H	4.81981200	-0.24641100	0.24477700	H	4.81253100	0.00460800	0.33850400
H	4.33370700	-0.85291300	-1.34432100	H	4.39708800	-0.63047700	-1.26402100
Fe	-0.10339000	-2.35532400	-1.49958400	Fe	0.04680000	-2.34864900	-1.53667700
C	-2.14966300	-2.37676000	-1.34956600	C	-1.99789700	-2.46414300	-1.38788700
C	-0.79584300	-4.13115400	-0.73283300	C	-0.56365200	-4.16372100	-0.79230300
C	-1.67734800	-3.65740700	-1.73911400	C	-1.46468900	-3.71652300	-1.79547700
C	0.55826000	-0.71151900	-2.52679000	C	0.60689800	-0.64698900	-2.52553200
C	1.43026600	-2.82079200	-2.78801900	C	1.60027700	-2.69559000	-2.83965400
C	0.57669800	-1.84782400	-3.37392700	C	0.68710100	-1.76106800	-3.40005800
H	-2.81501700	-1.75129800	-1.92310000	H	-2.69631900	-1.86335200	-1.94993200
H	-0.25520700	-5.06486600	-0.74990900	H	0.01963800	-5.07255500	-0.81903900
H	-1.92729100	-4.16768100	-2.65661500	H	-1.68878200	-4.22403700	-2.72250600
H	0.00871400	0.20277400	-2.69261700	H	-0.00344600	0.23344400	-2.66268200
H	1.62420500	-3.80928500	-3.17604700	H	1.84976800	-3.66394900	-3.24920100
H	0.01491200	-1.96189500	-4.28824900	H	0.12577500	-1.88817000	-4.31422700
C	-0.70506500	-3.14206900	0.28013500	C	-0.52485200	-3.18578800	0.23650700
C	-1.56594600	-2.05233100	-0.08420500	C	-1.43248400	-2.13254400	-0.11700400
C	1.95190200	-2.29137400	-1.57993000	C	2.09569400	-2.16132600	-1.62118200
C	1.42151800	-0.97325700	-1.40857700	C	1.48881000	-0.88108200	-1.41624900
H	-0.07587100	-3.19699000	1.15348500	H	0.09136400	-3.22751400	1.12063300
H	2.60444600	-2.78589500	-0.88048000	H	2.78610100	-2.63835700	-0.94600200
C	0.46827500	4.24428700	0.52256300	C	0.20803800	4.26393800	0.47161700
C	0.35663300	3.21827200	-0.59904800	C	0.16928200	3.21551600	-0.62162700
O	0.41344700	3.48120000	-1.76178600	O	0.22979000	3.47538300	-1.79772800
C	0.78246100	5.65474200	0.04167000	C	0.36814200	5.69069500	-0.03119200
H	1.73629600	5.68877400	-0.48633000	H	1.29935200	5.81424700	-0.58856000
H	0.01579400	6.02123800	-0.64174800	H	-0.45811200	5.97709900	-0.68556600
H	0.83764600	6.33529700	0.89316400	H	0.38533400	6.38180600	0.81491300
H	1.22019700	3.87857200	1.22772200	H	1.01402900	3.98383400	1.15634800
H	-0.47794100	4.21140700	1.07565900	H	-0.71409100	4.14606000	1.05214900

**2D-CO**				**S-2D-CO**			
Pd	0.04606200	1.54464000	0.28512600	Pd	0.03585700	1.54117300	0.32016800
P	1.90829600	0.09027100	-0.30283100	P	1.91330600	0.11767200	-0.30673800
P	-1.73677500	-0.24881700	0.71443500	P	-1.72811100	-0.26898200	0.71489100
C	-2.35992500	-0.44487600	2.51300600	C	-2.36759400	-0.47396400	2.50785200
C	3.60904500	0.64003100	-1.00851400	C	3.60693600	0.70268400	-0.99630400
C	2.40922700	-0.75802200	1.26959100	C	2.41407100	-0.76472200	1.24531900
C	-3.20816100	0.37706400	-0.24288400	C	-3.18977100	0.33103300	-0.27382800
N	2.82383000	-2.02297500	1.21136100	N	2.91864700	-1.99748500	1.16449000
H	2.76216500	4.80999500	-1.90847300	H	2.67934300	4.88467600	-1.80785800
N	-3.04615200	1.59780300	-0.76037000	N	-3.01849700	1.53435500	-0.83017300
C	0.83241900	3.93331300	-1.41593800	C	0.77340800	3.93201400	-1.36842700
C	1.72514800	5.11634000	-1.76394100	C	1.62420800	5.14681900	-1.70359500
H	1.70956200	5.86553900	-0.97205700	H	1.54085800	5.91333200	-0.93031200
C	-4.39403400	-0.33949700	-0.40297900	C	-4.37674400	-0.38537400	-0.41934900
C	-4.04902000	2.14305100	-1.44629900	C	-4.01658800	2.06444400	-1.53633000
C	-5.43555900	0.23727200	-1.11669900	C	-5.41221100	0.17509900	-1.15457500

H	-6.36832500	-0.29881100	-1.25039500
C	-5.26327000	1.50389500	-1.65311400
H	-6.04925300	1.99015900	-2.21783900
H	-4.50270800	-1.33061300	0.01453300
H	-3.87151900	3.13692200	-1.84583100
C	2.41774700	-0.01149700	2.44704200
C	3.25353700	-2.59240600	2.33720000
C	2.88195300	-0.61084600	3.60970000
H	2.91188200	-0.05457900	4.53966400
C	3.30594900	-1.93000200	3.55725200
H	3.67479600	-2.43905800	4.43939200
H	2.08018400	1.01894100	2.44623400
H	3.57998300	-3.62470100	2.25431800
C	1.19692500	3.23934700	-0.11945700
O	1.97584100	3.64533000	0.68228100
H	0.80390600	3.18136600	-2.20927600
C	-1.15935200	-0.84615600	3.37379500
H	-1.46009700	-0.84044800	4.42559600
H	-0.80534900	-1.85160100	3.14461500
H	-0.32632500	-0.14938500	3.26155900
C	-3.45791000	-1.49986000	2.64293300
H	-3.66931300	-1.65660400	3.70512700
H	-4.39101700	-1.18097600	2.17769200
H	-3.16128100	-2.46171900	2.22107300
C	-2.88200100	0.91080500	2.99078600
H	-3.68944000	1.29592700	2.36468100
H	-3.27811200	0.79959300	4.00419400
H	-2.08718500	1.65662200	3.03940800
C	4.38733400	1.42446000	0.05013800
H	5.30956100	1.79559000	-0.40643600
H	3.84206200	2.28431100	0.43628600
H	4.67520200	0.79071900	0.89030300
C	3.36806600	1.49806500	-2.24975700
H	4.32859900	1.69276400	-2.73540000
H	2.73019400	0.99572200	-2.98084300
H	2.93078500	2.46412200	-2.00404000
C	4.42143600	-0.59485500	-1.40765200
H	5.42244300	-0.26008700	-1.69612800
H	4.53285400	-1.30487100	-0.58727300
H	3.98804000	-1.11482300	-2.26141300
Fe	-0.19904800	-2.45630800	-1.35853300
C	-2.20387400	-2.56181000	-1.02716600
C	-0.69343500	-4.08493700	-0.20069100
C	-1.68261100	-3.87103100	-1.19489800
C	0.33765000	-0.84960000	-2.48568700
C	1.09508500	-2.98491300	-2.86960700
C	0.19641300	-1.98307700	-3.32516400
H	-2.94621300	-2.10073000	-1.65705700
H	-0.09131500	-4.97251500	-0.08235300
H	-1.96880100	-4.56672500	-1.96853100
H	-0.22298600	0.07062400	-2.56001500
H	1.19719400	-3.97808800	-3.27999400
H	-0.49931300	-2.07881800	-4.14451600
C	-0.59173900	-2.90843700	0.58432800
C	-1.54180800	-1.95199400	0.09053400

H	-6.34555400	-0.36318300	-1.27781100
C	-5.23319700	1.42544200	-1.72615600
H	-6.01492600	1.89903000	-2.30830000
H	-4.49549900	-1.36451100	0.02294100
H	-3.83181900	3.04373500	-1.96833300
C	2.31139100	-0.06665400	2.44603100
C	3.32405300	-2.58027100	2.29339300
C	2.75231100	-0.67802400	3.61167600
H	2.68959400	-0.15822600	4.56128000
C	3.26537900	-1.96364300	3.53671000
H	3.61573800	-2.48462400	4.42007000
H	1.89570500	0.93444100	2.46098300
H	3.72025200	-3.58712200	2.19664200
C	1.12995300	3.25638300	-0.06704900
O	1.88135300	3.71240000	0.74966900
H	0.79096700	3.17768900	-2.15938900
C	-1.15218100	-0.74614800	3.39544800
H	-1.48190400	-0.78936300	4.43868800
H	-0.67160500	-1.69824100	3.16497900
H	-0.40982400	0.04990700	3.31314200
C	-3.37085200	-1.61696000	2.64509600
H	-3.60667900	-1.74287800	3.70720900
H	-4.30925400	-1.40910400	2.12924300
H	-2.97232300	-2.56565200	2.28027700
C	-3.01289700	0.83965800	2.94929200
H	-3.83491700	1.14751700	2.29917100
H	-3.42025900	0.70338600	3.95620800
H	-2.28441300	1.65030900	3.00599800
C	4.36084400	1.47855200	0.08421500
H	5.27967100	1.87830300	-0.35700800
H	3.78944200	2.31860900	0.47801800
H	4.65034600	0.83518800	0.91765200
C	3.36537300	1.58226200	-2.22101800
H	4.32775500	1.78264900	-2.70294600
H	2.72360600	1.09493200	-2.95940800
H	2.92840100	2.54392200	-1.95894400
C	4.44643200	-0.50589600	-1.41412700
H	5.44014000	-0.14179500	-1.69566600
H	4.57221400	-1.22765200	-0.60583200
H	4.02603900	-1.01910600	-2.27939500
Fe	-0.15392800	-2.44206700	-1.37085300
C	-2.14934800	-2.61504400	-1.00172300
C	-0.56800000	-4.07191000	-0.18130600
C	-1.58063900	-3.90660500	-1.16360600
C	0.31594600	-0.79586600	-2.47760100
C	1.09497500	-2.90992900	-2.93765100
C	0.16304500	-1.91310900	-3.33767600
H	-2.92264000	-2.19215000	-1.62239300
H	0.06923200	-4.93580400	-0.06096500
H	-1.85323400	-4.62214900	-1.92550200
H	-0.25974300	0.11775900	-2.51019700
H	1.20164800	-3.89225800	-3.37500000
H	-0.56007200	-2.00142100	-4.13535800
C	-0.50095400	-2.88207100	0.58893300
C	-1.49041900	-1.96879500	0.09607600

C 1.80157700 -2.47917100 -1.74902800	C 1.83118500 -2.41516700 -1.82962500
C 1.33968300 -1.14571400 -1.49843800	C 1.35451500 -1.09602200 -1.53229000
H 0.11206600 -2.75658600 1.38514300	H 0.19612800 -2.69382500 1.38896500
H 2.52386500 -3.00408200 -1.14743100	H 2.59007800 -2.94492000 -1.27891200
H -0.20985700 4.25146400 -1.29585700	H -0.28291900 4.21089000 -1.27055000
H 1.37808800 5.58262300 -2.68749000	H 1.28866400 5.57983000 -2.64869600
C -1.23727200 2.93930000 0.85215900	C -1.28482200 2.88627700 0.88652000
O -1.89715600 3.78246100 1.21529100	O -1.97527500 3.71163200 1.23574900
**2D-CH_3OH**	**S-2D-CH_3OH**
Pd 0.10876100 1.48897200 -0.00921900	Pd 0.06112100 1.50285900 0.09205300
P 1.98191000 0.09222200 -0.15842000	P 1.99211700 0.20882500 -0.09699900
P -1.73849300 -0.26913000 0.58112600	P -1.70637200 -0.38436100 0.59648200
C -2.22592100 -0.54544000 2.41522700	C -2.24719900 -0.86577000 2.37540200
C 3.74096000 0.61118200 -0.73761800	C 3.74567000 0.84791100 -0.54671300
C 2.32585700 -0.53457800 1.55520400	C 2.29009400 -0.54706600 1.57232700
C -3.38952200 0.25196800 -0.13354500	C -3.34748300 0.13522500 -0.13173200
N 2.54646700 -1.83391500 1.74363200	N 2.58270100 -1.84427600 1.66655200
H 2.60637500 4.62199200 -2.51483900	H 1.97976700 4.98280500 -2.50523800
N -3.55230400 1.57082900 -0.31158500	N -3.53975400 1.45913400 -0.21238400
C 0.79867300 3.50828800 -2.03714000	C 0.54344200 3.41223500 -2.06306800
C 1.52386000 4.76017100 -2.51413700	C 0.90300800 4.80638100 -2.55409600
H 1.30053100 5.61308400 -1.87274500	H 0.40613200 5.57679500 -1.96032000
C -4.42026100 -0.64112500 -0.41960700	C -4.34753100 -0.75853000 -0.50703000
C -4.71348000 2.03540600 -0.77564300	C -4.70358500 1.93251400 -0.66148800
C -5.62855200 -0.15476200 -0.89867100	C -5.55818300 -0.26430900 -0.97243000
H -6.44030900 -0.83777800 -1.12206600	H -6.34752600 -0.94796300 -1.26490400
C -5.78217800 1.21041600 -1.08567800	C -5.74362900 1.10725800 -1.05551900
H -6.70657700 1.63108000 -1.46105500	H -6.67200400 1.53379700 -1.41544400
H -4.27738400 -1.70256500 -0.27335800	H -4.18783200 -1.82553700 -0.43659400
H -4.78212200 3.11082100 -0.90335600	H -4.79634800 3.01289600 -0.70463700
C 2.40429600 0.40243900 2.58679800	C 2.25651600 0.30042200 2.67944500
C 2.85658200 -2.24515000 2.97367400	C 2.85044600 -2.34002200 2.87633500
C 2.74840000 -0.03488700 3.85705000	C 2.56305300 -0.22120200 3.92733000
H 2.83097200 0.67054500 4.67608900	H 2.55531600 0.41651200 4.80446600
C 2.97857000 -1.38806300 4.05879000	C 2.86435500 -1.57112300 4.03174600
H 3.24779600 -1.77539800 5.03390100	H 3.10218000 -2.02542900 4.98648900
H 2.20369500 1.44993400 2.39329100	H 1.99099200 1.34473900 2.56367400
H 3.02557000 -3.31154700 3.09022600	H 3.07298200 -3.40250500 2.91883200
C 1.13992900 3.10673200 -0.61495700	C 0.93722900 3.14490800 -0.62648200
O 1.75981700 3.76876000 0.15873800	O 1.52958900 3.92428900 0.07071900
O -1.44918400 3.09770500 0.13655500	O -1.53755000 3.05432300 0.34977500
H -2.32078500 2.60693100 0.01277500	H -2.37786000 2.50270600 0.20851500
C -1.52708800 4.10033000 1.14697200	C -1.60450500 3.82866100 1.54444500
H -1.90121700 3.69731300 2.09181100	H -1.68228700 3.20203200 2.43687200
H -0.52761300 4.50117200 1.30598700	H -0.69586800 4.42690800 1.61317400
H -2.18355200 4.91051900 0.81822500	H -2.46636900 4.50147100 1.50268000
H 0.98591000 2.65016800 -2.68716200	H 1.01479900 2.63613800 -2.67293000
C -0.95814800 -0.87685400 3.20251900	C -0.98193900 -1.04967600 3.21163600
H -1.21016600 -0.94823500 4.26472200	H -1.26854200 -1.30984800 4.23597200
H -0.51428200 -1.82821900 2.91139900	H -0.33815500 -1.84710100 2.83722000
H -0.20377700 -0.09807000 3.08933900	H -0.40046000 -0.12762100 3.25119200
C -3.26709300 -1.64610800 2.60097900	C -3.10418900 -2.12692100 2.42002600
H -3.45665200 -1.77512000 3.67115500	H -3.36902200 -2.32859000 3.46368800
H -4.22061100 -1.39214300 2.13558900	H -4.03721800 -2.01070500 1.86542600

H -2.93206500 -2.60670000 2.20730800	H -2.57981400 -3.00334300 2.03693400
C -2.77755600 0.78602900 2.93152200	C -3.04102700 0.30981000 2.94830300
H -3.70710100 1.07361000 2.43822300	H -4.00315600 0.44569400 2.45096000
H -2.98748200 0.69173200 4.00079500	H -3.24076500 0.11103200 4.00623500
H -2.05324400 1.59444300 2.80887900	H -2.48522300 1.24852000 2.89095800
C 4.35421900 1.61630500 0.23948700	C 4.22504300 1.85383900 0.50099700
H 5.30607800 1.95962000 -0.17636500	H 5.17973200 2.26993400 0.16286800
H 3.72856700 2.49386600 0.39766200	H 3.53099400 2.68376400 0.63568200
H 4.57124300 1.16148900 1.20671500	H 4.40065300 1.38314500 1.46976900
C 3.63986500 1.23094500 -2.13119700	C 3.70364400 1.51183100 -1.92220100
H 4.65080800 1.40800700 -2.50931000	H 4.72933500 1.74794600 -2.22332700
H 3.13674100 0.57312600 -2.84273400	H 3.27898500 0.85901800 -2.68795000
H 3.13040800 2.19357000 -2.11682700	H 3.14849100 2.44919800 -1.91072800
C 4.63377900 -0.63088600 -0.80326700	C 4.71966900 -0.33079200 -0.58994400
H 5.64992100 -0.30750600 -1.04863500	H 5.72327900 0.06534000 -0.77719100
H 4.68018600 -1.16283100 0.14841900	H 4.75454400 -0.88042000 0.35258000
H 4.31319400 -1.33006000 -1.57518100	H 4.48603900 -1.03017500 -1.39380200
Fe -0.00508900 -2.55479500 -1.36322700	Fe 0.16932500 -2.42126500 -1.53056000
C -2.01902800 -2.30853100 -1.46504000	C -1.86004400 -2.27510200 -1.61297200
C -1.00109100 -4.14912200 -0.54092800	C -0.73684400 -4.11637800 -0.81569300
C -1.68853900 -3.67196700 -1.68666800	C -1.45373300 -3.60013600 -1.92787600
C 0.91511900 -1.13439300 -2.49191200	C 1.00135700 -0.85637200 -2.53307100
C 1.61235000 -3.32087600 -2.37418600	C 1.80993900 -3.00667500 -2.62303800
C 0.92856500 -2.37295600 -3.18266300	C 1.06822600 -2.02431100 -3.33515200
H -2.52542200 -1.66515500 -2.16675900	H -2.40495200 -1.61407700 -2.26876100
H -0.58799500 -5.13769900 -0.41200300	H -0.27085100 -5.08896000 -0.75250500
H -1.89723500 -4.23312100 -2.58453000	H -1.63202100 -4.10808800 -2.86451800
H 0.45698300 -0.21891800 -2.83446400	H 0.49471600 0.06326800 -2.78465300
H 1.75509300 -4.36520300 -2.60720300	H 2.00311300 -4.01777900 -2.95163300
H 0.46448400 -2.56820700 -4.13723900	H 0.59936900 -2.15528000 -4.29955900
C -0.89970100 -3.08449500 0.39283300	C -0.69463400 -3.11220300 0.18893100
C -1.55670100 -1.93557200 -0.15960400	C -1.41613100 -1.96844600 -0.28560200
C 2.03539800 -2.67535300 -1.18525000	C 2.21529600 -2.44976200 -1.38244700
C 1.60942700 -1.30994000 -1.24594100	C 1.71817100 -1.10855700 -1.31391200
H -0.39673600 -3.14318300 1.34315500	H -0.20547500 -3.20580500 1.14490500
H 2.55051500 -3.11968900 -0.35111900	H 2.77097800 -2.94821900 -0.60644400
H -0.28672600 3.65100300 -2.04115700	H -0.53199100 3.22580200 -2.13845300
H 1.21189400 5.00385700 -3.53127200	H 0.58722700 4.92365200 -3.59345400
**TS-2D_P**	**S-TS-2D_P**
Pd -0.30314700 1.27923900 0.34457300	Pd -0.27105500 1.35096700 0.31068600
P 1.90382700 0.75410300 -0.20441000	P 1.90263900 0.69325500 -0.18534000
P -1.41731100 -0.88459900 0.75663300	P -1.45651700 -0.85090300 0.73320100
C -1.97351300 -1.59074700 2.45877100	C -1.97953300 -1.53391400 2.45256700
C 3.27109100 1.96935500 -0.75395500	C 3.32379000 1.86795900 -0.69504100
C 2.65735600 -0.00759800 1.31868900	C 2.61029800 -0.11975800 1.33108000
C -3.09523600 -0.63537700 -0.04544000	C -3.13004500 -0.59061600 -0.05970400
N 3.43795400 -1.08359400 1.21207700	N 3.41668700 -1.17724300 1.21025100
H -1.19110200 5.41365100 -1.98315000	H -0.88594200 5.54202300 -1.91673300
N -3.50802900 0.64540900 -0.16567100	N -3.53066100 0.69156400 -0.20062000
C -1.01050000 3.26315200 -1.77584100	C -0.86430900 3.37379700 -1.75583000
C -1.78063300 4.51846500 -2.18377000	C -1.55020800 4.69360700 -2.09596000
H -2.71961000 4.60158800 -1.63385200	H -2.45412800 4.83530000 -1.49885500
C -3.96347300 -1.63151300 -0.47791200	C -4.00729400 -1.58728300 -0.45908500
C -4.70076000 0.99392500 -0.66698400	C -4.72268100 1.04311900 -0.69675300

C	-5.20623100	-1.30118300	-1.00172200	C	-5.25153200	-1.25362900	-0.98009600
H	-5.87961000	-2.08484000	-1.33003500	H	-5.93317000	-2.03882800	-1.28619200
C	-5.58639100	0.03181400	-1.09933400	C	-5.61831100	0.07818900	-1.10245900
H	-6.54736900	0.32217800	-1.50274900	H	-6.57972400	0.37084800	-1.50328900
H	-3.65959900	-2.66523000	-0.39837000	H	-3.72340700	-2.62482100	-0.36069100
H	-4.90350400	2.05642900	-0.70834200	H	-4.91928700	2.10571300	-0.75420700
C	2.40997400	0.61723200	2.54258500	C	2.30850600	0.45253100	2.56645400
C	3.99971800	-1.56698200	2.32026300	C	3.94248200	-1.69731700	2.32051000
C	3.01728500	0.11896500	3.68569700	C	2.88090700	-0.08174100	3.71189000
H	2.85624700	0.59575400	4.64614500	H	2.67113100	0.35198400	4.68358500
C	3.83019200	-0.99972200	3.57608700	C	3.71447800	-1.18323800	3.58970600
H	4.32448800	-1.42601200	4.44063400	H	4.17900000	-1.64062800	4.45549500
H	1.75654600	1.48118000	2.58588600	H	1.63587600	1.30078100	2.62141600
H	4.62511200	-2.44581700	2.19216900	H	4.58482800	-2.56320500	2.18526700
C	-0.61741200	3.29832900	-0.31331900	C	-0.41232800	3.30705800	-0.30985900
O	-0.04380600	4.18365400	0.25654000	O	0.13911700	4.21852100	0.28254900
O	-2.04517600	2.75224300	0.46776500	O	-1.95475200	2.78474700	0.47202500
H	-2.86549700	1.45718100	0.12909000	H	-2.89046300	1.48579400	0.08475200
C	-2.29058700	3.40671700	1.70614000	C	-2.18402000	3.37509600	1.73573000
H	-2.55718400	2.67868900	2.47622000	H	-2.45815800	2.61992200	2.47836100
H	-1.39805900	3.95327600	2.01968000	H	-1.29003800	3.89960800	2.09022300
H	-3.10949500	4.12124300	1.58070300	H	-2.99852000	4.10452200	1.65363500
H	-0.08368700	3.18913300	-2.34677200	H	0.03153100	3.24853200	-2.36921800
C	-0.69840400	-1.76372500	3.29042600	C	-0.74097600	-1.44569200	3.34663900
H	-0.98011200	-1.99797800	4.32125200	H	-1.00748300	-1.78914200	4.35155600
H	-0.08307300	-2.58800500	2.93032900	H	0.07824500	-2.07338700	2.99195700
H	-0.09031600	-0.85717300	3.30640900	H	-0.37950900	-0.41869000	3.42605300
C	-2.71772900	-2.91896900	2.37364600	C	-2.49658900	-2.96706200	2.40772500
H	-2.87982300	-3.30455300	3.38511100	H	-2.74048100	-3.28218500	3.42825700
H	-3.70312800	-2.81375300	1.91596100	H	-3.40772700	-3.06044100	1.81407000
H	-2.15066900	-3.67153700	1.82248700	H	-1.75274500	-3.66284900	2.01565000
C	-2.84807000	-0.52044900	3.11334200	C	-3.05654700	-0.60244000	3.00990800
H	-3.80093700	-0.38357800	2.59626200	H	-4.00575900	-0.69741600	2.47838900
H	-3.07931100	-0.82186000	4.13886500	H	-3.23763100	-0.86480800	4.05708400
H	-2.33392600	0.44279700	3.16238300	H	-2.74652600	0.44540600	2.98457500
C	3.41200100	3.03866700	0.33205200	C	3.54595200	2.87170700	0.43732800
H	4.10948600	3.80652900	-0.01565800	H	4.27976100	3.61562600	0.10994400
H	2.45933700	3.52699700	0.54856500	H	2.62827500	3.40212500	0.70059700
H	3.81774100	2.62784400	1.25830000	H	3.94094200	2.39423800	1.33657800
C	2.82779800	2.62757900	-2.06084000	C	2.90906000	2.61003100	-1.96448900
H	3.62985500	3.28048300	-2.41753900	H	3.74864700	3.22708200	-2.30135600
H	2.62531700	1.89738000	-2.84723800	H	2.65407800	1.92833600	-2.77945600
H	1.94526000	3.25055400	-1.91344300	H	2.06485100	3.27607500	-1.78604400
C	4.60072800	1.24751100	-0.96791200	C	4.61480100	1.09506800	-0.95988200
H	5.35850000	1.98465400	-1.25233400	H	5.40428200	1.81296500	-1.20856500
H	4.95096300	0.74466100	-0.06565300	H	4.94610300	0.52758400	-0.08873200
H	4.54204000	0.50927600	-1.76958900	H	4.51797900	0.40827500	-1.80308100
Fe	0.75368100	-2.23478400	-1.58241300	Fe	0.70502600	-2.24055600	-1.60514000
C	-1.18854200	-2.84819300	-1.44718700	C	-1.25158600	-2.81368800	-1.46677800
C	0.60456800	-4.09511000	-0.72821600	C	0.51833600	-4.09779300	-0.75000000
C	-0.35020300	-3.95035400	-1.76732500	C	-0.43438500	-3.93144700	-1.78997100
C	0.95289100	-0.46794000	-2.58657800	C	0.90338400	-0.45818800	-2.58240100
C	2.30747000	-2.28847600	-2.92787700	C	2.22933900	-2.29059200	-2.98535800
C	1.21929600	-1.54883200	-3.46427400	C	1.13990600	-1.52613200	-3.48538300
H	-1.98254700	-2.47136300	-2.07163600	H	-2.04595600	-2.42281500	-2.08289200

H 1.40209600 -4.82097000 -0.69303600<br>H -0.41199600 -4.54773800 -2.66397000<br>H 0.17704400 0.27312300 -2.70257000<br>H 2.72594400 -3.19184500 -3.34548000<br>H 0.66989200 -1.78668700 -4.36233000<br>C 0.36919500 -3.08068800 0.23549500<br>C -0.76949100 -2.31340500 -0.18379500<br>C 2.72691000 -1.66112800 -1.72647000<br>C 1.89236600 -0.52324100 -1.50189000<br>H 0.97251000 -2.90579700 1.11076000<br>H 3.50102200 -1.99101400 -1.05468700<br>H -1.58686800 2.35960100 -1.98297800<br>H -2.00825000 4.49002600 -3.25134300	H 1.29941200 -4.84312500 -0.71280100<br>H -0.50833200 -4.52537800 -2.68933000<br>H 0.13241800 0.29296100 -2.67218600<br>H 2.62875300 -3.19169900 -3.42842700<br>H 0.56659800 -1.74010900 -4.37574200<br>C 0.29917000 -3.08121400 0.21706500<br>C -0.81956700 -2.29006700 -0.20550600<br>C 2.67752000 -1.69316000 -1.77808500<br>C 1.85949600 -0.54882300 -1.51412900<br>H 0.88020500 -2.93605100 1.11395200<br>H 3.46912600 -2.05104800 -1.14157000<br>H -1.51790600 2.52718400 -1.96973500<br>H -1.83530700 4.70579200 -3.15112300
**N2A-C_2H_4**<br><br>Pd 0.24987800 -1.72180500 -0.33902500<br>P -1.86134900 -0.56498800 -0.57084600<br>P 1.59364300 -0.07583000 0.68281700<br>C 2.14258800 -0.42655400 2.48951400<br>C -3.24595100 -1.24516500 -1.69892000<br>C -2.73481900 -0.34257800 1.05463600<br>C 3.26425000 -0.09692300 -0.17276000<br>N -3.41932300 0.77398400 1.31215900<br>N 3.43440900 -1.19733600 -0.92765500<br>C 4.32081700 0.80182600 -0.10322000<br>C 4.55437200 -1.49098600 -1.59966300<br>C 5.49885000 0.53697600 -0.78983900<br>H 6.32645300 1.23481200 -0.73083100<br>C 5.62258200 -0.62330700 -1.54872400<br>H 6.52968800 -0.85082600 -2.09270400<br>H 4.21592000 1.69953500 0.48933600<br>H 4.55564900 -2.41423500 -2.16431000<br>C -2.66975000 -1.40276600 1.96008000<br>C -4.05800700 0.86555000 2.47807400<br>C -3.35706800 -1.30593700 3.16109200<br>H -3.33454400 -2.12085100 3.87600100<br>C -4.06644600 -0.14519400 3.43058000<br>H -4.61567400 -0.02037100 4.35590100<br>H -2.08747300 -2.28383000 1.71569400<br>H -4.59794400 1.79168000 2.65301400<br>C 0.85587600 -0.62018900 3.29786300<br>H 1.11907900 -0.94434100 4.30905800<br>H 0.28305200 0.30255900 3.39382600<br>H 0.21269100 -1.38217300 2.85366200<br>C 2.97684000 0.69713200 3.09648600<br>H 3.17559700 0.46646100 4.14767600<br>H 3.94792600 0.80206900 2.60817800<br>H 2.46129000 1.65795500 3.06043000<br>C 2.92787300 -1.73934400 2.49128000<br>H 3.89639600 -1.64700200 1.99330200<br>H 3.12743200 -2.03440000 3.52524100<br>H 2.36477000 -2.54585700 2.01657900<br>C -3.67266300 -2.61420900 -1.16789500<br>H -4.41720000 -3.04443400 -1.84422600<br>H -2.83306800 -3.30891100 -1.11264400	**S-N2A-C_2H_4**<br><br>Pd 0.11569200 -1.78872200 0.02850500<br>P -1.79366500 -0.48700000 -0.66550500<br>P 1.71491000 -0.19678100 0.79583900<br>C 2.66842400 -0.56297800 2.41345400<br>C -2.95065600 -1.15754900 -2.03017300<br>C -2.93488000 -0.21364800 0.78032600<br>C 3.06028900 -0.11306000 -0.50114100<br>N -3.89723800 0.71390300 0.72556200<br>N 2.89310300 -1.00989600 -1.49510000<br>C 4.18505100 0.69286900 -0.55767300<br>C 3.73203900 -1.17099000 -2.52431400<br>C 5.08288000 0.55801600 -1.61087500<br>H 5.96265800 1.19006400 -1.64838400<br>C 4.85886100 -0.38292300 -2.60593400<br>H 5.54226300 -0.50848400 -3.43515100<br>H 4.36927600 1.42185800 0.21724700<br>H 3.46834700 -1.93080600 -3.24751100<br>C -2.75511800 -1.03294900 1.89429500<br>C -4.70048300 0.85011100 1.78143300<br>C -3.60318100 -0.88877000 2.98391000<br>H -3.48448600 -1.51663000 3.86042700<br>C -4.59828000 0.07496700 2.92927800<br>H -5.28363800 0.22919400 3.75464400<br>H -1.95471800 -1.76561200 1.89540100<br>H -5.46670000 1.61689000 1.70371600<br>C 1.58935300 -0.94090100 3.43318200<br>H 2.07112900 -1.20897600 4.37912200<br>H 0.90682300 -0.11135500 3.63436800<br>H 0.99972600 -1.79819400 3.09837700<br>C 3.47000700 0.62344600 2.93922300<br>H 3.91630900 0.34743600 3.90085400<br>H 4.28818600 0.90369700 2.27362500<br>H 2.84253600 1.50118600 3.10656600<br>C 3.58546700 -1.76067800 2.17062600<br>H 4.40781700 -1.51811100 1.49324900<br>H 4.02388100 -2.07254000 3.12418600<br>H 3.03993800 -2.61369300 1.76115400<br>C -3.64509900 -2.40358300 -1.48043600<br>H -4.21505800 -2.88340900 -2.28319600<br>H -2.92758700 -3.13398300 -1.09865000

H -4.13170200 -2.54551700 -0.17969000	H -4.34592500 -2.15905500 -0.67826400
C -2.65165700 -1.39578500 -3.10117700	C -2.06026600 -1.54230800 -3.21347600
H -3.39285300 -1.86073100 -3.75802600	H -2.67811200 -1.98688800 -4.00121100
H -2.38626200 -0.43043400 -3.53605300	H -1.55467800 -0.67351400 -3.64288000
H -1.76192000 -2.02909600 -3.09869000	H -1.30083300 -2.27437500 -2.92739600
C -4.45005100 -0.30624100 -1.74380500	C -3.99164900 -0.13788900 -2.48393800
H -5.20872500 -0.73140600 -2.40866900	H -4.62815300 -0.59340300 -3.25122900
H -4.90602500 -0.17368100 -0.76158200	H -4.63260800 0.18312000 -1.66137800
H -4.18564600 0.67865800 -2.13108100	H -3.52812900 0.74857900 -2.92249000
Fe -0.22483500 2.48967300 -0.60928100	Fe -0.10928300 2.46493700 -0.30205300
C 1.74785800 2.74369400 -0.18649600	C 1.76630300 2.76876200 0.44899800
C 0.04826200 3.67291900 1.05256500	C -0.16789900 3.30802800 1.57727300
C 1.04458900 3.94912600 0.08177400	C 0.91679600 3.84252800 0.83117300
C -0.54259700 1.31522400 -2.24076800	C -0.18651900 1.41534100 -2.04605800
C -1.60427400 3.31971100 -1.88963500	C -1.21149900 3.44759800 -1.73373800
C -0.57198800 2.67983000 -2.62712000	C -0.09441900 2.80292900 -2.33210200
H 2.53416900 2.63530100 -0.91603500	H 2.65890900 2.86783200 -0.14741800
H -0.67201300 4.37501800 1.44304400	H -0.99507900 3.86684700 1.99007800
H 1.22039200 4.89935400 -0.39850800	H 1.06095600 4.88143200 0.57150800
H 0.14087400 0.56105300 -2.60087800	H 0.52669800 0.65945700 -2.33921500
H -1.85555700 4.36877700 -1.93139500	H -1.41480700 4.50894500 -1.74591900
H 0.09368400 3.15420200 -3.33199100	H 0.70060900 3.28522000 -2.88213900
C 0.12257500 2.29598700 1.38661800	C 0.00154100 1.90089700 1.65336000
C 1.19607200 1.70594700 0.63739100	C 1.20905400 1.55164300 0.96054100
C -2.22561700 2.35309200 -1.05720100	C -2.00087100 2.45920400 -1.08506200
C -1.57191300 1.09815500 -1.26322200	C -1.37151800 1.18648900 -1.27008500
H -0.54530800 1.78431700 2.05969000	H -0.67644600 1.20649700 2.12545100
H -3.01886000 2.51794400 -0.34798900	H -2.90487800 2.63098000 -0.52382300
H 2.59920300 -1.84067400 -0.93249500	H 2.04875400 -1.59897100 -1.42976600
C -0.06462500 -3.70012700 -1.10743700	C -0.27734300 -3.89853500 -0.35103100
C 1.30326300 -3.60354900 -0.82195200	C 0.98669300 -3.77740300 0.23137700
H 1.99384000 -3.56822900 -1.66404200	H 1.88150000 -3.86869400 -0.37844800
H -0.72214300 -4.21925600 -0.41749500	H -1.12560300 -4.21915900 0.24715800
H -0.40640400 -3.67433900 -2.13560200	H -0.37432300 -4.07898600 -1.41774000
H 1.70712400 -4.06970600 0.07096600	H 1.12678800 -4.00483300 1.28448900
**TS-N2A-C_2H_4_2B**	**S-TS-N2A-C_2H_4_2B**
Pd 0.28336300 -1.66625600 -0.37381200	Pd 0.27234900 -1.65976100 -0.39202300
P -1.83796400 -0.60495400 -0.53091900	P -1.83706400 -0.59983300 -0.53404200
P 1.57703800 0.01765700 0.71633700	P 1.58041900 0.00667900 0.71932100
C 2.05009100 -0.28912800 2.54290100	C 2.01592600 -0.31235600 2.55058600
C -3.22533500 -1.32882800 -1.62345800	C -3.23550700 -1.31727200 -1.61927300
C -2.66521600 -0.40395800 1.11868100	C -2.65330600 -0.40065900 1.12103000
C 3.22762300 -0.16836900 -0.13437100	C 3.23077200 -0.17494100 -0.12503700
N -3.31963600 0.72291200 1.40198600	N -3.29805400 0.72866500 1.42463300
N 3.34415500 -1.33302000 -0.78177900	N 3.33543600 -1.32241400 -0.80294800
C 4.29329000 0.72391200 -0.10365400	C 4.30042200 0.70973400 -0.06487100
C 4.47526800 -1.67136700 -1.39829200	C 4.46325600 -1.65057800 -1.42970500
C 5.47548400 0.38904800 -0.75230300	C 5.47979100 0.38318200 -0.72304800
H 6.31513900 1.07458300 -0.73970400	H 6.32449800 1.06223200 -0.68984400
C 5.57423200 -0.82888600 -1.41035500	C 5.56774400 -0.81473800 -1.41728500
H 6.48024900 -1.12234200 -1.92512500	H 6.47097300 -1.09982000 -1.94253100
H 4.19995200 1.66681000 0.41786300	H 4.21898400 1.63732500 0.48541300
H 4.48800500 -2.63551500 -1.89457600	H 4.46772500 -2.59962700 -1.95457400
C -2.60930400 -1.48113500 2.00334200	C -2.58703900 -1.48392500 1.99683100

C	-3.94049100	0.80953000	2.57785000	C	-3.89149800	0.81097800	2.61667300
C	-3.27802300	-1.38834900	3.21535500	C	-3.22786100	-1.39469200	3.22398100
H	-3.26308400	-2.21481400	3.91693000	H	-3.19879500	-2.22597900	3.92003100
C	-3.95804100	-0.21716200	3.51310900	C	-3.89237100	-0.22065200	3.54542500
H	-4.49168300	-0.09617800	4.44795600	H	-4.40006700	-0.09935900	4.49520500
H	-2.05021600	-2.37137500	1.73949700	H	-2.03825900	-2.37617300	1.71924900
H	-4.45703800	1.74425300	2.77526400	H	-4.39719300	1.74757000	2.83503900
C	0.75134900	-0.39398000	3.34581400	C	0.70529400	-0.54145300	3.30503600
H	0.99322100	-0.68174700	4.37304900	H	0.93657000	-0.77514200	4.34947600
H	0.21410800	0.55332800	3.39550800	H	0.05554000	0.33527000	3.30216600
H	0.08205600	-1.15017900	2.93219900	H	0.14973700	-1.38326900	2.88693000
C	2.93238700	0.81897900	3.11150100	C	2.79581300	0.83954400	3.17662300
H	3.12490900	0.61451400	4.16920900	H	3.01512700	0.59062500	4.22059900
H	3.90322600	0.86821100	2.61487000	H	3.75043300	1.01287100	2.67540200
H	2.45666300	1.79872500	3.04474300	H	2.22780400	1.77186700	3.17073600
C	2.77720500	-1.63528900	2.60538600	C	2.84133300	-1.59984700	2.60266300
H	3.74640100	-1.60912700	2.10359400	H	3.82846600	-1.47929500	2.15152400
H	2.95966700	-1.89225900	3.65266900	H	2.98872000	-1.87957700	3.65062000
H	2.18103600	-2.43960700	2.16665400	H	2.33205800	-2.43122800	2.10710700
C	-3.58205300	-2.72250300	-1.10530300	C	-3.57346100	-2.72173300	-1.11978800
H	-4.33341600	-3.16609200	-1.76492100	H	-4.32940200	-3.15931100	-1.78004800
H	-2.71783800	-3.38799200	-1.09244600	H	-2.70383500	-3.38107700	-1.12581900
H	-4.01040300	-2.69045200	-0.10153700	H	-3.98985100	-2.70923200	-0.10985800
C	-2.68605300	-1.42377300	-3.05240800	C	-2.71699200	-1.38610800	-3.05651600
H	-3.43295800	-1.91373300	-3.68370100	H	-3.46404100	-1.88873600	-3.67944700
H	-2.48871900	-0.43896200	-3.47855600	H	-2.55017500	-0.39226000	-3.47684800
H	-1.76772100	-2.01177600	-3.10568600	H	-1.78534600	-1.95145300	-3.13140200
C	-4.46358600	-0.43302200	-1.59842400	C	-4.48465200	-0.43991700	-1.56578300
H	-5.22745000	-0.86853200	-2.25003400	H	-5.24613000	-0.87019800	-2.22543800
H	-4.89150900	-0.34647200	-0.59884800	H	-4.90971700	-0.38863900	-0.56153700
H	-4.24859400	0.57105200	-1.96624800	H	-4.28736100	0.57735200	-1.90980100
Fe	-0.24804700	2.49960600	-0.69112100	Fe	-0.24919400	2.50651300	-0.68711200
C	1.74682500	2.73907600	-0.35691600	C	1.75130500	2.72855000	-0.34910600
C	0.11239200	3.81631100	0.84932700	C	0.12032900	3.81310600	0.85899300
C	1.08084700	3.98011600	-0.17395300	C	1.09016300	3.97350300	-0.16606500
C	-0.59831200	1.26969400	-2.27348200	C	-0.58687200	1.26666600	-2.26757700
C	-1.71023600	3.24939600	-1.93095000	C	-1.69002500	3.25726000	-1.94956800
C	-0.69021600	2.62089700	-2.69475000	C	-0.66344600	2.61711800	-2.69685900
H	2.51154400	2.54410400	-1.09136700	H	2.52114800	2.52980600	-1.07825400
H	-0.57620800	4.56938000	1.20026700	H	-0.56349100	4.57013800	1.21464100
H	1.26343100	4.88065400	-0.73990100	H	1.27809600	4.87445500	-0.73194400
H	0.09271100	0.52874500	-2.64630200	H	0.10685900	0.51988400	-2.62560400
H	-1.99636600	4.28857400	-1.99015500	H	-1.96974300	4.29887900	-2.01703400
H	-0.06906600	3.09504800	-3.43916500	H	-0.02690000	3.08308200	-3.43501000
C	0.16589400	2.47256700	1.30115300	C	0.17158400	2.46749800	1.31003200
C	1.20008600	1.79369400	0.57200100	C	1.19963600	1.78655300	0.57742900
C	-2.26334500	2.29000700	-1.04483400	C	-2.25979600	2.30364300	-1.06512100
C	-1.57920600	1.04994400	-1.24683900	C	-1.58111600	1.05709300	-1.25251500
H	-0.48640200	2.04306100	2.04320400	H	-0.46340500	2.03891700	2.06875700
H	-3.03215700	2.45031400	-0.30820600	H	-3.04224800	2.48150600	-0.34672400
H	2.15567100	-2.12525600	-0.73367600	H	2.12288100	-2.15053000	-0.76265300
C	0.02902000	-3.55635300	-1.23887100	C	0.03653100	-3.53987000	-1.27998000
C	1.44159000	-3.44940500	-1.02406700	C	1.44852600	-3.43721400	-1.04103400
H	2.05070400	-3.44958000	-1.92749900	H	2.07601400	-3.44369600	-1.93300400
H	-0.55537900	-4.17291700	-0.56294000	H	-0.55932800	-4.16377200	-0.61913300

H -0.34627600 -3.52617400 -2.25489200<br>H 1.87555600 -4.03205300 -0.21535300	H -0.31916400 -3.50109100 -2.30424900<br>H 1.86492100 -4.01992200 -0.22217100

Table S5. Computed Energetic Data for All Optimized Structures

**CO**<br><br>HF= -113.304332<br>ZPE= 0.005076<br>NImag= 0<br>Htot = -113.295951<br>Gtot = -113.318375	**S-CO**<br><br>HF= -113.29534<br>ZPE= 0.005061<br>NImag= 0<br>Htot = -113.286973<br>Gtot = -113.309397<br><br>HF(empiricaldispersion=gd3bj)= -113.2960501
**CH_3OH**<br><br>HF= -115.724973<br>ZPE= 0.05124<br>NImag= 0<br>Htot = -115.669466<br>Gtot = -115.696472	**S-CH_3OH**<br><br>HF= -115.733956<br>ZPE= 0.051124<br>NImag= 0<br>Htot = -115.678545<br>Gtot = -115.7056<br><br>HF(empiricaldispersion=gd3bj)= -115.7370073
**$(CH_3OH)_3$**<br><br>HF= -347.201075<br>ZPE= 0.159369<br>NImag= 0<br>Htot = -347.02903<br>Gtot = -347.081073	**S-$(CH_3OH)_3$**<br><br>HF= -347.211996<br>ZPE= 0.157196<br>NImag= 0<br>Htot = -347.041392<br>Gtot =-347.096782
**C_2H_4**<br><br>HF= -78.586425<br>ZPE= 0.050974<br>NImag= 0<br>Htot = -78.531465<br>Gtot = -78.55697	**S-C_2H_4**<br><br>HF= -78.587419<br>ZPE= 0.050828<br>NImag= 0<br>Htot = -78.532607<br>Gtot = -78.558114<br><br>HF(empiricaldispersion=gd3bj)= -78.5913029
**$CH_3OCOCH_2CH_3$**<br><br>HF= -307.696533<br>ZPE= 0.11834<br>NImag= 0<br>Htot = -307.569849<br>Gtot = -307.61028	**S-$CH_3OCOCH_2CH_3$**<br><br>HF= -307.705488<br>ZPE= 0.117962<br>NImag= 0<br>Htot = -307.579256<br>Gtot = -307.619487<br><br>HF(empiricaldispersion=gd3bj)= -307.7213122
**1A**<br><br>HF= -1750.913535<br>ZPE= 0.638122<br>NImag= 0<br>Htot = -1750.239247	**S-1A**<br><br>HF= -1750.995839<br>ZPE= 0.637165<br>NImag= 0<br>Htot = -1750.322857

Gtot = -1750.338511	Gtot = -1750.420551<br><br>HF(empiricaldispersion=gd3bj)= -1751.20763
**1B**<br><br>HF= -1829.537997<br>ZPE= 0.696643<br>NImag= 0<br>Htot = -1828.802673<br>Gtot = -1828.905819	**S-1B**<br><br>HF= -1829.615231<br>ZPE= 0.694619<br>NImag= 0<br>Htot = -1828.881876<br>Gtot = -1828.985922<br><br>HF(empiricaldispersion=gd3bj)= -1829.844582
**1C**<br><br>HF= -1942.85383<br>ZPE= 0.706346<br>NImag= 0<br>Htot = -1942.105995<br>Gtot = -1942.215727	**S-1C**<br><br>HF= -1942.928593<br>ZPE= 0.704867<br>NImag= 0<br>Htot = -1942.182513<br>Gtot = -1942.291065<br><br>HF(empiricaldispersion=gd3bj)= -1943.167947
**TS-1C_1D**<br><br>HF= -1942.846745<br>ZPE= 0.705066<br>NImag= -257.9799<br>Htot = -1942.100634<br>Gtot = -1942.209744	**S-TS-1C_1D**<br><br>HF= -1942.923448<br>ZPE= 0.704438<br>NImag= -276.339<br>Htot = -1942.178517<br>Gtot =-1942.285885<br><br>HF(empiricaldispersion=gd3bj)= -1943.162462
**1D**<br><br>HF= -1942.873842<br>ZPE= 0.707165<br>NImag= 0<br>Htot = -1942.125504<br>Gtot = -1942.23523	**S-1D**<br><br>HF= -1942.95389<br>ZPE= 0.706024<br>NImag= 0<br>Htot = -1942.207284<br>Gtot =-1942.314547<br><br>HF(empiricaldispersion=gd3bj)= -1943.187352
**1D-CO**<br><br>HF= -2056.188629<br>ZPE= 0.71547<br>NImag= 0<br>Htot = -2055.429357<br>Gtot = -2055.544354	**S-1D-CO**<br><br>HF= -2056.265852<br>ZPE= 0.714658<br>NImag= 0<br>Htot = -2055.50795<br>Gtot =-2055.620963<br><br>HF(empiricaldispersion=gd3bj)= -2056.51052
**1D-CH_3OH**<br><br>HF= -2058.600113<br>ZPE= 0.760869<br>NImag= 0<br>Htot = -2057.793678<br>Gtot = -2057.912468	**S-1D-CH_3OH**<br><br>HF= -2058.684212<br>ZPE= 0.760004<br>NImag= 0<br>Htot = -2057.879131<br>Gtot =-2057.996415

	HF(empiricaldispersion=gd3bj)= -2058.936367
**TS-1D_P**<br><br>HF= -2058.554972<br>ZPE= 0.757328<br>NImag= -200.9275<br>Htot = -2057.752368<br>Gtot = -2057.872637	**S-TS-1D_P**<br><br>HF= -2058.637796<br>ZPE=0.75711<br>NImag= -118.594<br>Htot = -2057.836319<br>Gtot = -2057.952647<br><br>HF(empiricaldispersion=gd3bj)= -2058.884264
**2A**<br><br>HF= -2130.260054<br>ZPE= 0.563231<br>NImag= 0<br>Htot = -2129.659883<br>Gtot = -2129.76543	**S-2A**<br><br>HF= -2130.339641<br>ZPE= 0.561649<br>NImag= 0<br>Htot = -2129.74097<br>Gtot = -2129.847214<br><br>HF(empiricaldispersion=gd3bj)= -2130.553763
**N-2A**<br><br>HF= -2130.262915<br>ZPE= 0.567239<br>NImag= 0<br>Htot = -2129.659207<br>Gtot = -2129.762776	**S-N-2A**<br><br>HF= -2130.348594<br>ZPE= 0.566356<br>NImag= 0<br>Htot = -2129.745515<br>Gtot = -2129.851519<br><br>HF(empiricaldispersion=gd3bj)= -2130.565485
**2B**<br><br>HF= -2208.885043<br>ZPE= 0.621005<br>NImag= 0<br>Htot = -2208.224669<br>Gtot = -2208.333327	**S-2B**<br><br>HF= -2208.961541<br>ZPE= 0.619138<br>NImag= 0<br>Htot = -2208.302981<br>Gtot = -2208.411201<br><br>HF(empiricaldispersion=gd3bj)= -2209.200394
**2C**<br><br>HF= -2322.197185<br>ZPE= 0.630552<br>NImag= 0<br>Htot = -2321.5245<br>Gtot = -2321.638464	**S-2C**<br><br>HF= -2322.270886<br>ZPE= 0.629535<br>NImag= 0<br>Htot = -2321.599457<br>Gtot = -2321.712412<br><br>HF(empiricaldispersion=gd3bj)= -2322.521399
**TS-2C_2D**<br><br>HF= -2322.189872<br>ZPE= 0.629662<br>NImag= -245.4711<br>Htot = -2321.518556<br>Gtot = -2321.631978	**S-TS-2C_2D**<br><br>HF= -2322.264303<br>ZPE= 0.628406<br>NImag= -302.9848<br>Htot = -2321.594472<br>Gtot =-2321.706433

	HF(empiricaldispersion=gd3bj)= -2322.514233
**2D**<br><br>HF= -2322.228336<br>ZPE= 0.631796<br>NImag= 0<br>Htot = -2321.554724<br>Gtot = -2321.670231	**S-2D**<br><br>HF= -2322.308537<br>ZPE= 0.630137<br>NImag= 0<br>Htot = -2321.636699<br>Gtot = -2321.750884<br><br>HF(empiricaldispersion=gd3bj)= -2322.553633
**2D-CO**<br><br>HF= -2435.539526<br>ZPE= 0.640219<br>NImag= 0<br>Htot = -2434.855047<br>Gtot = -2434.975384	**S-2D-CO**<br><br>HF= -2435.615277<br>ZPE= 0.638533<br>NImag= 0<br>Htot = -2434.932607<br>Gtot =-2435.051504<br><br>HF(empiricaldispersion=gd3bj)= -2435.872701
**2D-CH_3OH**<br><br>HF= -2437.961477<br>ZPE= 0.686778<br>NImag= 0<br>Htot = -2437.229365<br>Gtot = -2437.350969	**S-2D-CH_3OH**<br><br>HF= -2438.038432<br>ZPE= 0.683966<br>NImag= 0<br>Htot = -2437.309068<br>Gtot = -2437.430327<br><br>HF(empiricaldispersion=gd3bj)= -2438.301335
**TS-2D_P**<br><br>HF= -2437.928708<br>ZPE= 0.685337<br>NImag= -286.6979<br>Htot = -2437.198713<br>Gtot = -2437.319175	**S-TS-2D_P**<br><br>HF= -2438.01858<br>ZPE= 0.684537<br>NImag= -272.5647<br>Htot = -2437.289712<br>Gtot = -2437.408366<br><br>HF(empiricaldispersion=gd3bj)= -2438.280008
**N2A-C_2H_4**<br><br>HF= -2208.879327<br>ZPE= 0.622266<br>NImag= 0<br>Htot = -2208.217861<br>Gtot = -2208.325585	**S-N2A-C_2H_4**<br><br>HF= -2208.966698<br>ZPE= 0.620384<br>NImag= 0<br>Htot = -2208.306744<br>Gtot = -2208.415826<br><br>HF(empiricaldispersion=gd3bj)= -2209.204806
**TS-N2A-C_2H_4_2B**<br><br>HF= -2208.865755<br>ZPE= 0.616606<br>NImag= -1229.1429<br>Htot = -2208.210368<br>Gtot = -2208.317015	**S-TS-N2A-C_2H_4_2B**<br><br>HF= -2208.940619<br>ZPE= 0.615249<br>NImag= -1292.5934<br>Htot = -2208.286738<br>Gtot = -2208.392276<br><br>HF(empiricaldispersion=gd3bj)= -2209.1808308

Table S6. Computed geometry and energetic data for the three-methanol cluster at B3LYP/LANL2DZ(Pd)/6-31g(d) level of theory

Mecking's work[7]	This work
Cartesian coordinates	Cartesian coordinates
O 1.554293 0.238264 0.000000<br>H 1.082673 -0.619779 0.000000<br>O -0.573437 -1.465259 0.000000<br>H -1.078382 -0.625989 0.000000<br>O -0.977030 1.224410 0.000000<br>H 0.000000 1.238611 0.000000<br>C 2.954052 0.035606 0.000000<br>C -1.452182 -2.573314 0.000000<br>C -1.508964 2.538171 0.000000<br>H -0.838855 -3.479215 0.000000<br>H -2.095161 -2.594909 0.892928<br>H -2.095161 -2.594909 -0.892928<br>H 3.428725 1.021206 0.000000<br>H 3.295658 -0.509274 0.893004<br>H 3.295658 -0.509274 -0.893004<br>H -0.717337 3.297430 0.000000<br>H -2.132929 2.697003 -0.889910<br>H -2.132929 2.697003 0.889910	O 0.642949 -1.338737 -0.600143<br>C 1.419624 -2.135314 0.283759<br>H 2.408689 -2.257182 -0.167002<br>H 1.541452 -1.670122 1.273352<br>H 0.979494 -3.132912 0.422038<br>H -0.232648 -1.169840 -0.178158<br>O -1.492087 -0.076750 0.563637<br>C -2.733967 0.078273 -0.113090<br>H -3.277716 0.965274 0.240852<br>H -2.609717 0.155497 -1.202709<br>H -3.338987 -0.805773 0.106591<br>H -0.912700 0.684158 0.323039<br>O 0.554592 1.410447 -0.491960<br>H 0.852919 0.491491 -0.688359<br>C 1.560544 2.060179 0.273123<br>H 2.495861 2.165049 -0.294444<br>H 1.192755 3.061154 0.515066<br>H 1.779756 1.534693 1.214707
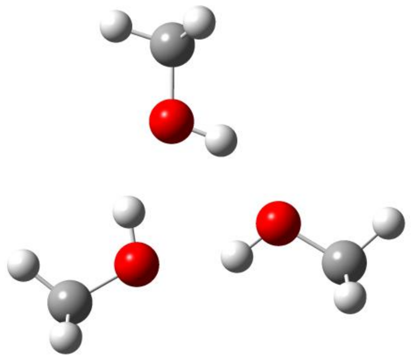	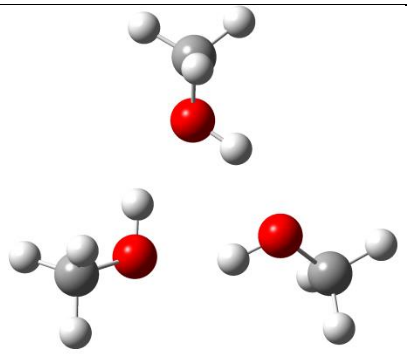
-347.1736537 a.u.<br>ΔE = 23.1 kJ/mol	-347.1817391 a.u.<br>ΔE = 0.0 kJ/mol
-347.049626 a.u.<br>ΔG = 25.1 kJ/mol	-347.059231 a.u.<br>ΔG = 0.0 kJ/mol

5. Alkoxycarbonylation of various alkenes with ferrocenyl phosphine ligands

General procedure: Under argon atmosphere, a vial (4 mL) was charged with [Pd(acac)$_2$] (1.22 mg, 0.2 mol%), **L2** (8.2 mg, 0.8 mol%), PTSA (12.2 mg, 3.2 mol%) and a stirring bar. Then MeOH (2.0 mL) and the alkene **1** (2.0 mmol) were added. The vial was placed in an alloyed plate, which was then transferred into an autoclave (300 mL) under argon atmosphere. The autoclave was flushed with CO three times at room temperature and then pressurized with CO to 40 bar. The reaction was performed at 120 ^{o}C for 20 h. After the reaction finished, the autoclave was cooled to room temperature and the pressure was carefully released. The regioselectivity of the product was measured by GC analysis. The yield of the desired ester **2**[8] was determined by isolated products through column chromatography on silica gel or detected by GC analysis using isooctane as internal standard. Methoxycarbonylation of **1b** (40 mmol) and **1c** (80 mmol) on large scale was carried out in a 100 mL autoclave following modified procudure.

methyl 3,5,5-trimethylhexanoate **2b**

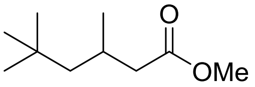

^{1}H-NMR ($CDCl_3$, 300 MHz): δ = 0.65 (s, 9 H, -CH_3), 0.72 (d, ^{2}J (^{1}H, ^{1}H) = 6.523 Hz, 3 H, - CH_3), 0.92 (m, 2 H, -CH_2), 1.88 (m, 2 H, -CH_2), 2.05 (m, 1 H, - C*H*), 3.40 (s, 3 H, -CH_3). **^{13}C-NMR** ($CDCl_3$, 300 MHz): δ = 22.66 (s, -*C*H$_3$), 26.94 (s, -*C*H), 29.86 (s, -CH$_3$), 31.00 (s, -$C_{quartär}$), 43.77 (s, -*C*H), 50.48 (s, -*C*H$_2$), 51.21 (s, -*C*H$_3$), 173.48 (s, -*C*=O).

methyl cyclooctanecarboxylate **2d**

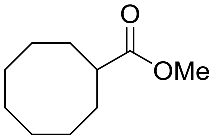

^{1}H-NMR ($CDCl_3$, 300 MHz): δ = 1.62 (s, 14 H, - CH_2), 2.50 (m, 1 H, - C*H*), 3.63 (s, 3 H, -CH_3). **^{13}C-NMR** ($CDCl_3$, 300 MHz): δ = 25.19 (s, -CH$_2$), 26.08 (s, -*C*H$_2$), 26.73 (s, -*C*H$_2$), 28.69 (s, -*C*H$_2$), 43.43 (s, -*C*H), 51.45 (s, -*C*H$_3$), 177.71 (s, -*C*=O).

methyl 2,3-dihydro-1H-indene-1-carboxylate **2e**

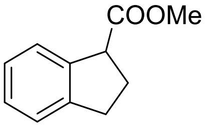

major product

^{1}H-NMR ($CDCl_3$, 300 MHz): δ = 2.40-3.42 (m, 4 H, aliph. –CH_2), 3.84 (s, 3 H, -CH_3), 4.18 (t, ^{3}J (^{1}H, ^{1}H) = 7.292 Hz), 1 H, -C*H*), 7.29-7.53 (s, 4H, -C*H*). **^{13}C-NMR** ($CDCl_3$, 300 MHz): major: δ = 28.87 (s, -CH$_2$), 31,87 (s, -CH$_2$), 50.20 (s, -*C*H), 52.01 (s, *C*H$_3$), 124.42(s, -$CH_{,arom.}$) , 124.77 (s, -$CH_{,arom.}$), 126.54 (s, -$CH_{,arom.}$), 127.65 (s, -$CH_{,arom.}$), 140.82 (s, -$CH_{arom.}$), 144.16 (s, -$C_{arom.}$), 174,34 (s, -C=O). minor: δ= 36.31(s, -CH$_2$), 43.58 (s, -*C*H), 51.91(s, *C*H$_3$), 124.88 (s, -$CH_{,arom.}$), 126.72 (s, -$CH_{,arom.}$), 141.63(s, -$C_{arom.}$) ,175.71 (s, -*C*=O).

methyl 4-phenylbutanoate **2i**

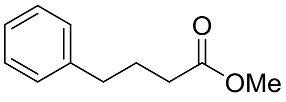

major product

^{1}H-NMR ($CDCl_3$, 300 MHz): δ = 1.86 (m, 2H, -CH_2), 2.22 (t, ^{3}J (^{1}H, ^{1}H) = 7.63 Hz, 2 H, -CH_2-$C_{arom.}$), 2.54 (t, ^{3}J (^{1}H, ^{1}H) = 7.32 Hz , 2 H, -CH_2-C=O), 3.54 (s, 3 H, -CH_3), 7.05-7.21 (s, 5 H, -$CH_{arom.}$). **^{13}C-NMR** ($CDCl_3$, 300 MHz): δ = 26.53 (s, -*C*H$_2$), 33.37 (s, -*C*H$_2$-C=O), 35.15 (s, -$CH_{2.}$), 51.47 (s, -*C*H$_3$), 126.0-141.38 (s, -$C_{arom.}$), 173.87 (s, -*C*=O).

methyl 3-methyldecanoate **2j**

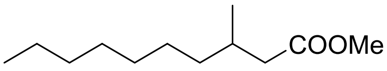

^{1}H-NMR ($CDCl_3$, 300 MHz): δ = 0.82 (m, 6 H, -CH_3), 1.19 (s, 12 H, -CH_2), 2.01 (m, 2 H, -CH_2), 2.23 (m, 1 H, -CH), 3.59 (s, 3 H, -CH_3). **^{13}C-NMR** ($CDCl_3$, 300 MHz): δ = 14.02 (s, -CH_3), 19.68 (s, -CH_3), 22.63 (s, -CH_2), 26.89 (s, -CH_2), 29.26 (s, -CH), 29.68 (s, -CH_2), 30.31(s, -CH_2), 31.84 (s, -CH_2), 36.71 (s, -CH_2), 41.61 (s, -CH_2), 51.50 (s, -CH_3), 173.68 (s, -C=O).

methyl 3-phenylbutanoate **2k**

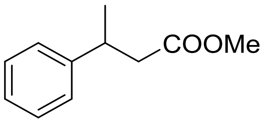

^{1}H-NMR ($CDCl_3$, 300 MHz): δ = 1.20 (d, ^{2}J (^{1}H, ^{1}H) = 6.954 Hz, 3 H, - CH_3), 2.47 (m, 2 H, -CH_2), 3.18 (m, 1 H, -CH), 3.50 (s, 3 H, - CH_3), 7.06-7.21 (m/s, 5 H, -CH_{arom}). **^{13}C-NMR** ($CDCl_3$, 300 MHz): δ = 21,84 (s, - CH_3), 36.50 (s, -CH_2), 42.77 (s, -CH), 51.49 (s, CH_3), 126.47 (s, -$CH_{,arom.}$), 126.76(s, -$CH_{,arom.}$), 128.57(s, -$CH_{,arom.}$), 145.76 (s, -$C_{arom.}$), 172.82 (s, -C=O).

methyl 3,3-diphenylpropanoate **2l**

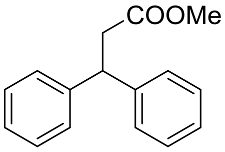

^{1}H-NMR ($CDCl_3$, 300 MHz): δ = 3.16 (d, ^{2}J (^{1}H, ^{1}H) = 7.986 Hz, 2 H, - CH_2), 3.65 (s, 3 H, - CH_3), 4.66 (t, ^{3}J (^{1}H, ^{1}H) = 4.659 Hz, 1 H, -CH), 7.24-7.39 (m, 10 H, $CH_{arom.}$). **^{13}C-NMR** ($CDCl_3$, 300 MHz): δ = 40.65 (s, -CH_2), 47.05 (s, -CH), 51.72 (s, CH_3), 126.62 (s, -$CH_{arom.}$), 127.72 (s, -$CH_{arom.}$), 128.64 (s, -$CH_{arom.}$), 143.55 (s, -$C_{arom.}$), 172.30 (s, -C=O).

dimethyl 3,3'-(1,3-phenylene)dibutyrate **2m**

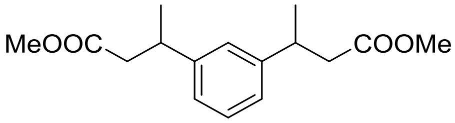

^{1}H-NMR ($CDCl_3$, 300 MHz): δ = 1.32 (d, ^{2}J (^{1}H, ^{1}H) = 6.960 Hz, 6 H, - CH_3), 2.60 (m, 4 H, -CH_2), 3.31 (m, 2 H, -CH), 3.63 (s, 6 H, - CH_3), 7.07-7.27 (s, 4H, -CH). **^{13}C-NMR** ($CDCl_3$, 300 MHz): δ = 21.72 (s, CH_3), 36.47 (s, -CH_2), 42.75 (s, -CH), 51.37 (s, CH_3), 124.68 (s, -$CH_{arom.}$), 125.36 (s, -$CH_{arom.}$), 128.67 (s, -$CH_{arom.}$), 145.92 (s, -$C_{arom.}$), 172.66 (s, -C=O).

methyl 5-cyanopentanoate **2o**

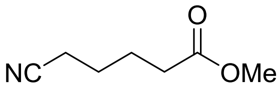

^{1}H-NMR ($CDCl_3$, 300 MHz): δ = 1.69 (m, 4 H, -CH_2), 2.32 (m, 4 H, -CH_2, 3.61 (s, 3 H, -CH_3). **^{13}C-NMR** ($CDCl_3$, 300 MHz): δ = 16.84 (s, -CH_2), 23.80 (s, -CH_2), 24.74 (s, -CH_2), 32.94 (s, -CH_2), 51.55 (s, -CH_3), 119.39 (s, -CN), 173.12 (s, -C=O).

dimethyl acetylaspartate **2p**

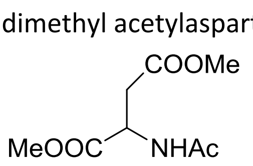

^{1}H-NMR ($CDCl_3$, 300 MHz): δ = 1.97 (s, 3 H, -CH_3), 2.86 (m, 2 H, -CH_2), 3.62 (s, 3 H, -CH_3), 3.69 (s, 3 H, -CH_3), 4.80 (m, 1 H, -CH), 6.78 (d, ^{2}J (^{1}H, ^{1}H) = 7.923 Hz, 1 H, -NH). **^{13}C-NMR** ($CDCl_3$, 300 MHz): δ = 22.92 (s, -CH_3), 36.06 (s, -CH_2), 48.48 (s, -CH_3), 51.95 (s, -CH_3), 52.68 (s, -CH), 169.99 (s, -C=O), 171.24 (s, -C=O), 171.45 (s, -C=O).

6. References

(1) Frisch, M. J.; Trucks, G. W.; Schlegel, H. B.; Scuseria, G. E.; Robb, M. A.; Cheeseman, J. R.; Scalmani, G.; Barone, V.; Petersson, G. A.; Nakatsuji, H.; Li, X.; Caricato, M.; Marenich, A.; Bloino, J.; Janesko, B. G.; Gomperts, R.; Mennucci, B.; Hratchian, H. P.; Ortiz, J. V.; Izmaylov, A. F.; Sonnenberg, J. L.; Williams-Young, D.; F. Ding, F. L.; F. Egidi, J. G.; B. Peng, A. P.; Henderson, T.; Ranasinghe, D.; Zakrzewski, V. G.; J. Gao, N. R.; G. Zheng, W. L.; M. Hada, M. E.; Toyota, K.; Fukuda, R.; Hasegawa, J.; Ishida, M.; Nakajima, T.; Honda, Y.; Kitao, O.; Nakai, H.; Vreven, T.; Throssell, K.; Jr., J. A. M.; Peralta, J. E.; Ogliaro, F.; Bearpark, M.; Heyd, J. J.; Brothers, E.; Kudin, K. N.; Staroverov, V. N.; Keith, T.; Kobayashi, R.; Normand, J.; Raghavachari, K.; Rendell, A.; Burant, J. C.; Iyengar, S. S.; Tomasi, J.; Cossi, M.; Millam, J. M.; Klene, M.; Adamo, C.; Cammi, R.; Ochterski, J. W.; R. L. Martin; Morokuma, K.; Farkas, O.; Foresman, J. B.; Fox, D. J. *Gaussian Inc.: Wallingford, CT, USA* **2009**.

(2) Becke, A. D. *J. Chem. Phys.* **1993**, *98*, 5648-5652.

(3) Schäfer, A.; Huber, C.; Ahlrichs, R. *J. Chem. Phys.* **1994**, *100*, 5829-5835.

(4) Hay, P. J.; Wadt, W. R. *J. Chem. Phys.* **1985**, *82*, 299-310.

(5) Marenich, A. V.; Cramer, C. J.; Truhlar, D. G. *The Journal of Physical Chemistry B* **2009**, *113*, 6378-6396.

(6) (a) Walther, G.; Knöpke, L. R.; Rabeah, J.; Chęciński, M. P.; Jiao, H.; Bentrup, U.; Brückner, A.; Martin, A.; Köckritz, A. *J. Catal.* **2013**, *297*, 44-55. (b) Crawford, L.; Cole-Hamilton, D. J.; Drent, E.; Bühl, M. *Chem. Eur. J.* **2014**, *20*, 13923-13926.

(7) (a) Roesle, P.; Dürr, C. J.; Möller, H. M.; Cavallo, L.; Caporaso, L.; Mecking, S. *J. Am. Chem. Soc.* **2012**, *134*, 17696-17703. (b) Roesle, P.; Caporaso, L.; Schnitte, M.; Goldbach, V.; Cavallo, L.; Mecking, S. *J. Am. Chem. Soc.* **2014**, *136*, 16871-16881.

(8) Dong, K.; Fang, X.; Gülak, S.; Franke, R.; Spannenberg, A.; Neumann, H.; Jackstell, R.; Beller, M. *Nat. Commun.* **2017**, *8*, 14117-14123.

7. Spectra of the carbonylation products (Figure S12)

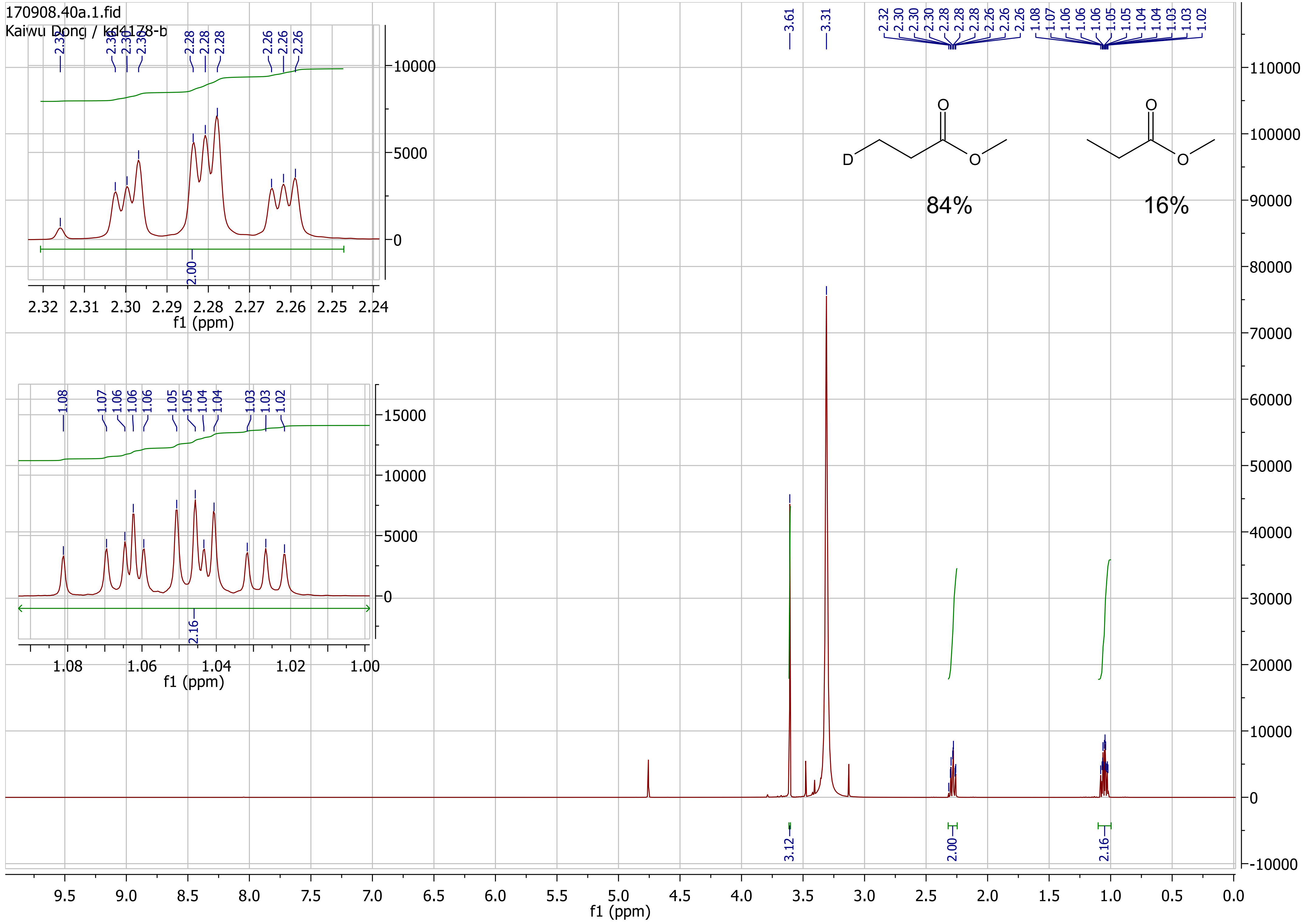
170908.40a.1.fid
Kaiwu Dong / kd4178-b
84%
16%
D
O
3.61
3.31
3.12
2.00
2.16
f1 (ppm)

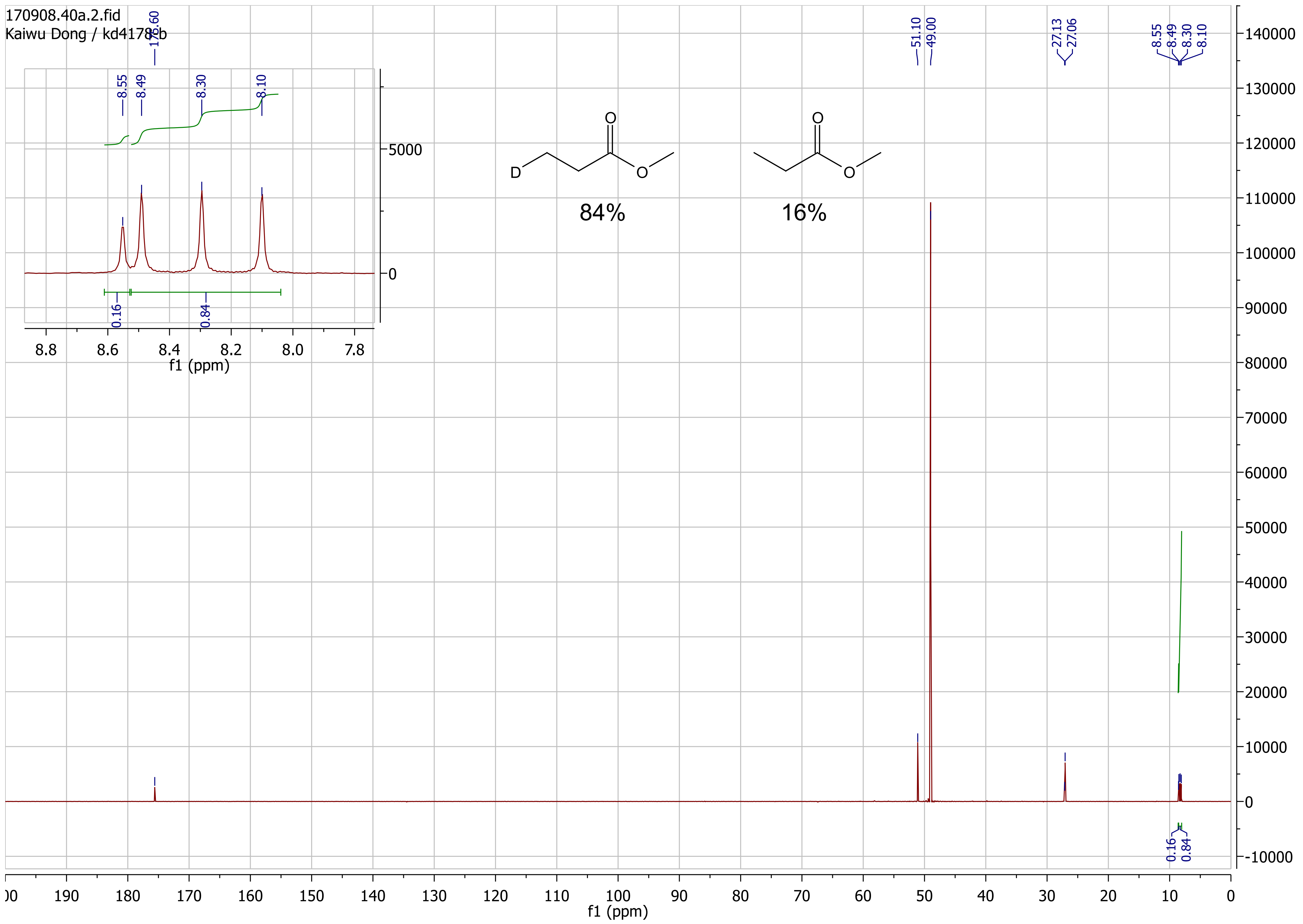

170908.40a.2.fid
Kaiwu Dong / kd4178-b
175.60
51.10
49.00
27.13
27.06
8.55
8.49
8.30
8.10
0.16
0.84
84%
16%
f1 (ppm)

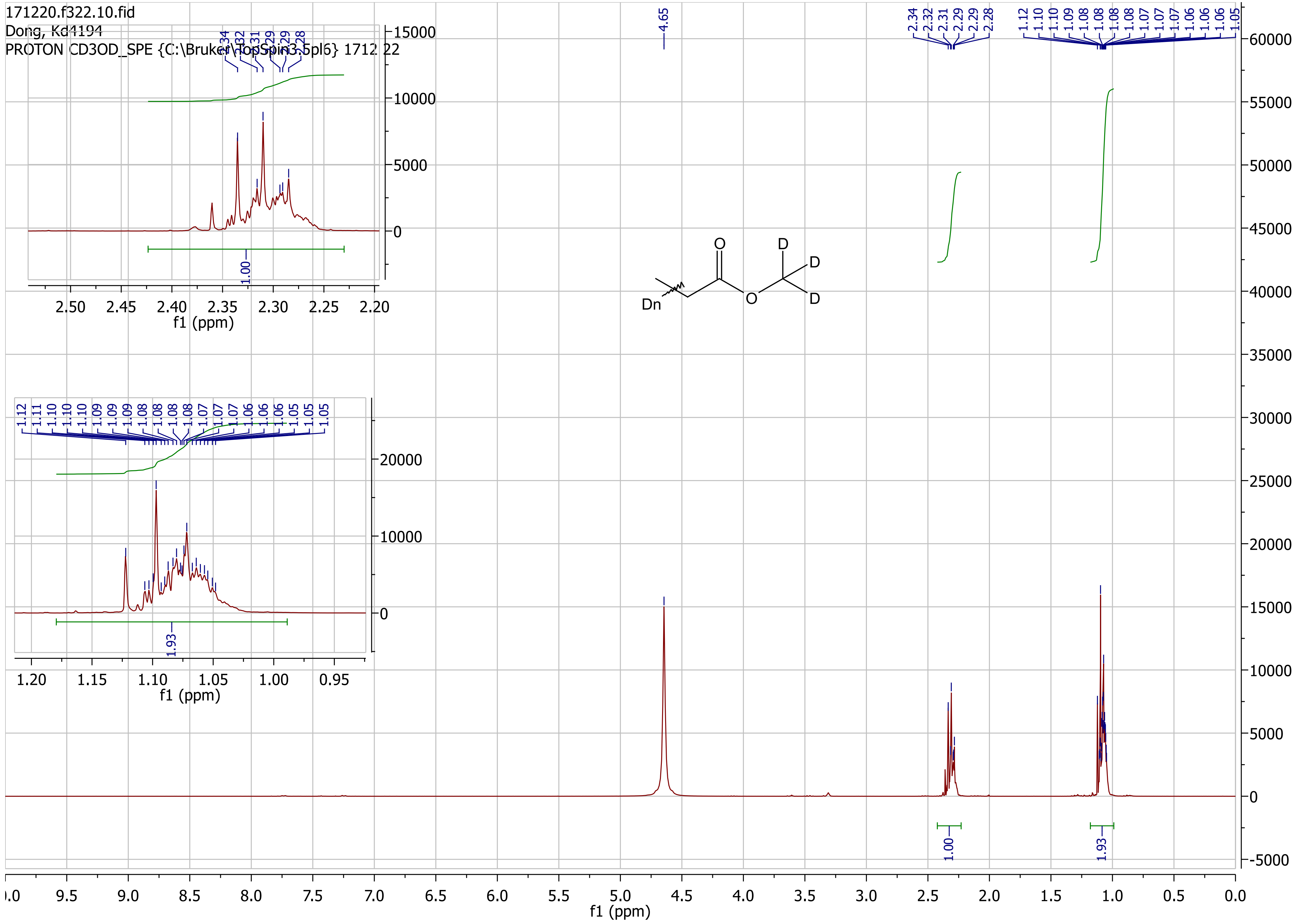
171220.f322.10.fid
Dong, Kd4194
PROTON CD3OD_SPE {C:\Bruker\TopSpin3.5pl6} 1712 22
4.65
2.34 2.32 2.31 2.29 2.29 2.28
1.12 1.10 1.10 1.09 1.08 1.08 1.08 1.08 1.07 1.07 1.06 1.06 1.06 1.05
1.00
1.93
Dn
O
O
D
D
D
f1 (ppm)

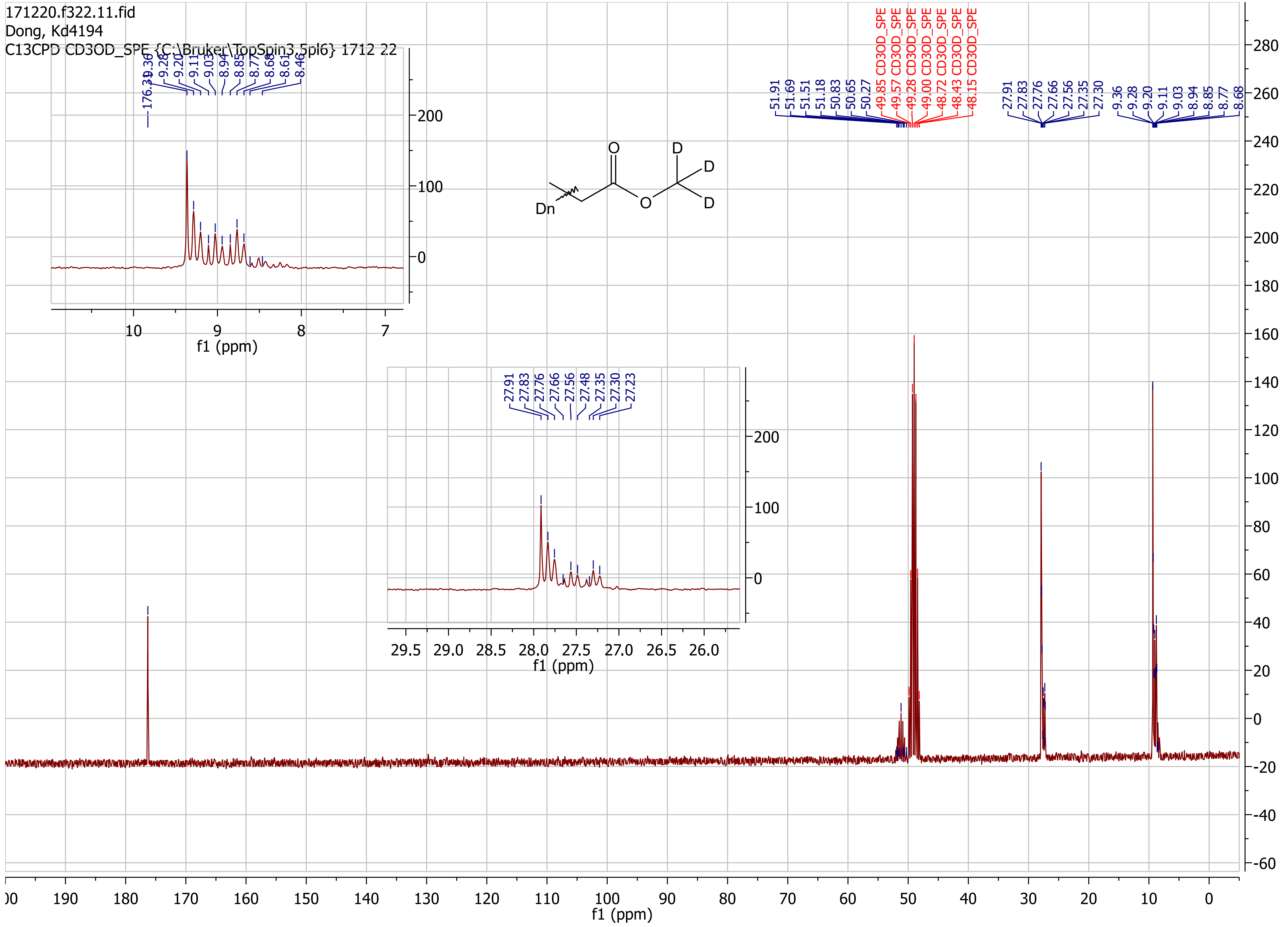
171220.f322.11.fid
Dong, Kd4194
C13CPD CD3OD_SPE {C:\Bruker\TopSpin3.5pl6} 1712 22
176.31
9.36
9.28
9.20
9.11
9.03
8.94
8.85
8.77
8.68
8.61
8.46
51.91
51.69
51.51
51.18
50.83
50.65
50.27
49.85 CD3OD_SPE
49.57 CD3OD_SPE
49.28 CD3OD_SPE
49.00 CD3OD_SPE
48.72 CD3OD_SPE
48.43 CD3OD_SPE
48.15 CD3OD_SPE
27.91
27.83
27.76
27.66
27.56
27.48
27.35
27.30
27.23
O
D
D
D
O
Dn
f1 (ppm)

[Type text]

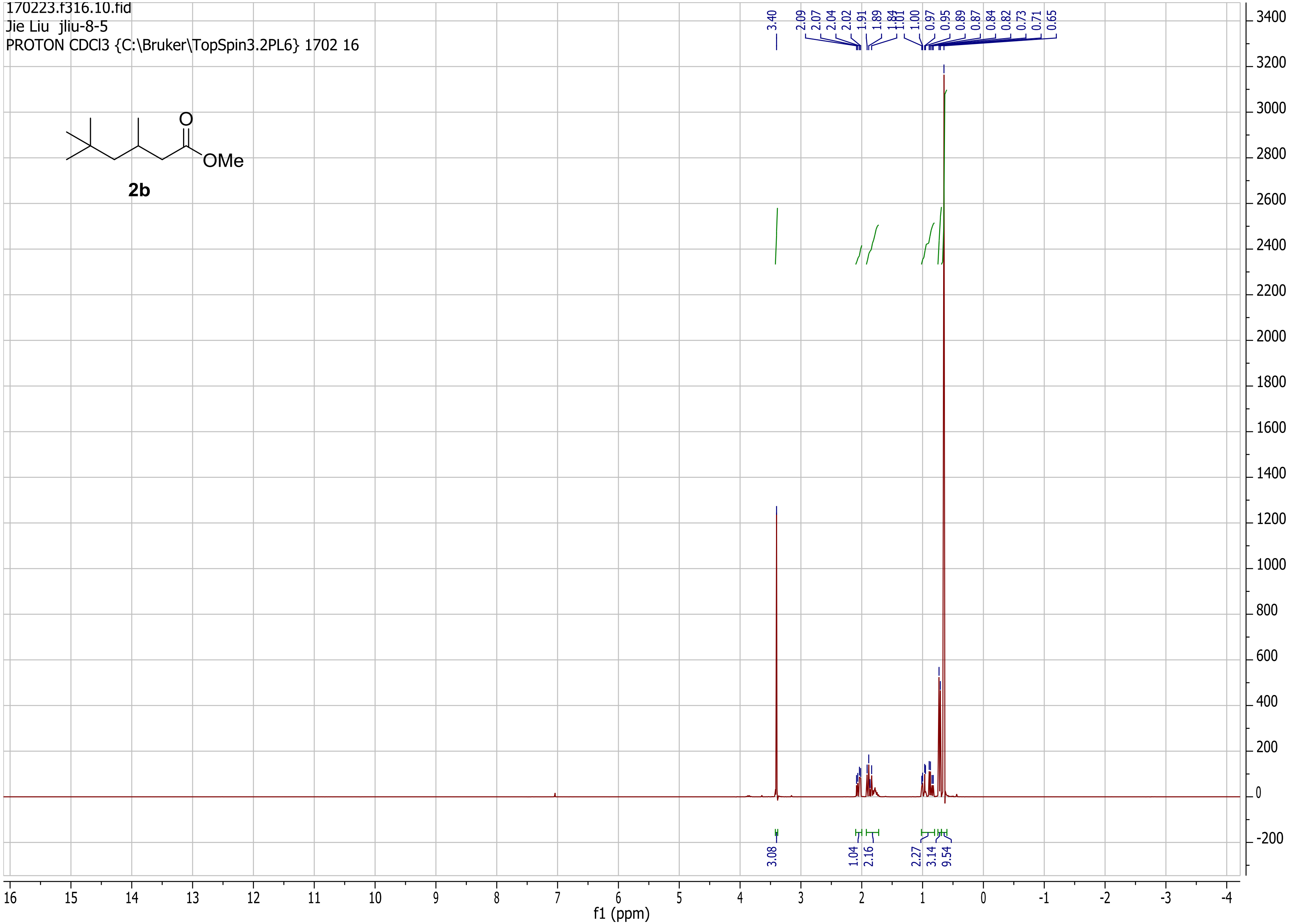

170223.f316.10.fid
Jie Liu jliu-8-5
PROTON CDCl3 {C:\Bruker\TopSpin3.2PL6} 1702 16
OMe
O
2b
3.40
2.09
2.07
2.04
2.02
1.91
1.89
1.84
1.81
1.00
0.97
0.95
0.89
0.87
0.84
0.82
0.73
0.71
0.65
3.08
1.04
2.16
2.27
3.14
9.54
f1 (ppm)

[Type text]

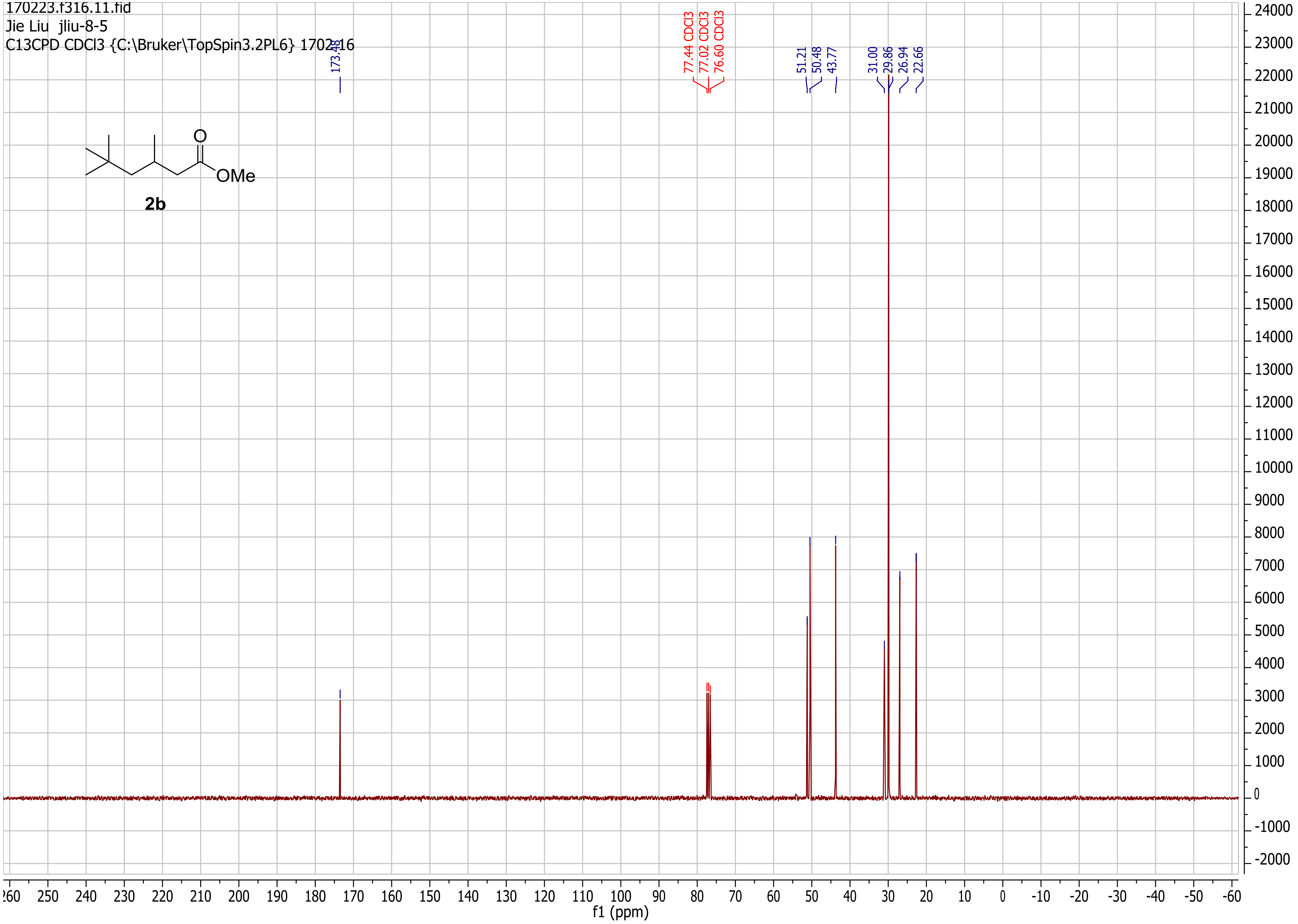

170223.f316.11.fid
Jie Liu jliu-8-5
C13CPD CDCl3 {C:\Bruker\TopSpin3.2PL6} 1702?16
173.48
77.44 CDCl3
77.02 CDCl3
76.60 CDCl3
51.21
50.48
43.77
31.00
29.86
26.94
22.66
O
OMe
2b
24000
23000
22000
21000
20000
19000
18000
17000
16000
15000
14000
13000
12000
11000
10000
9000
8000
7000
6000
5000
4000
3000
2000
1000
0
-1000
-2000
260 250 240 230 220 210 200 190 180 170 160 150 140 130 120 110 100 90 80 70 60 50 40 30 20 10 0 -10 -20 -30 -40 -50 -60
f1 (ppm)

[Type text]

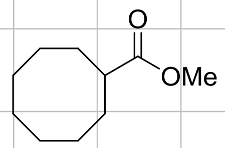

2d

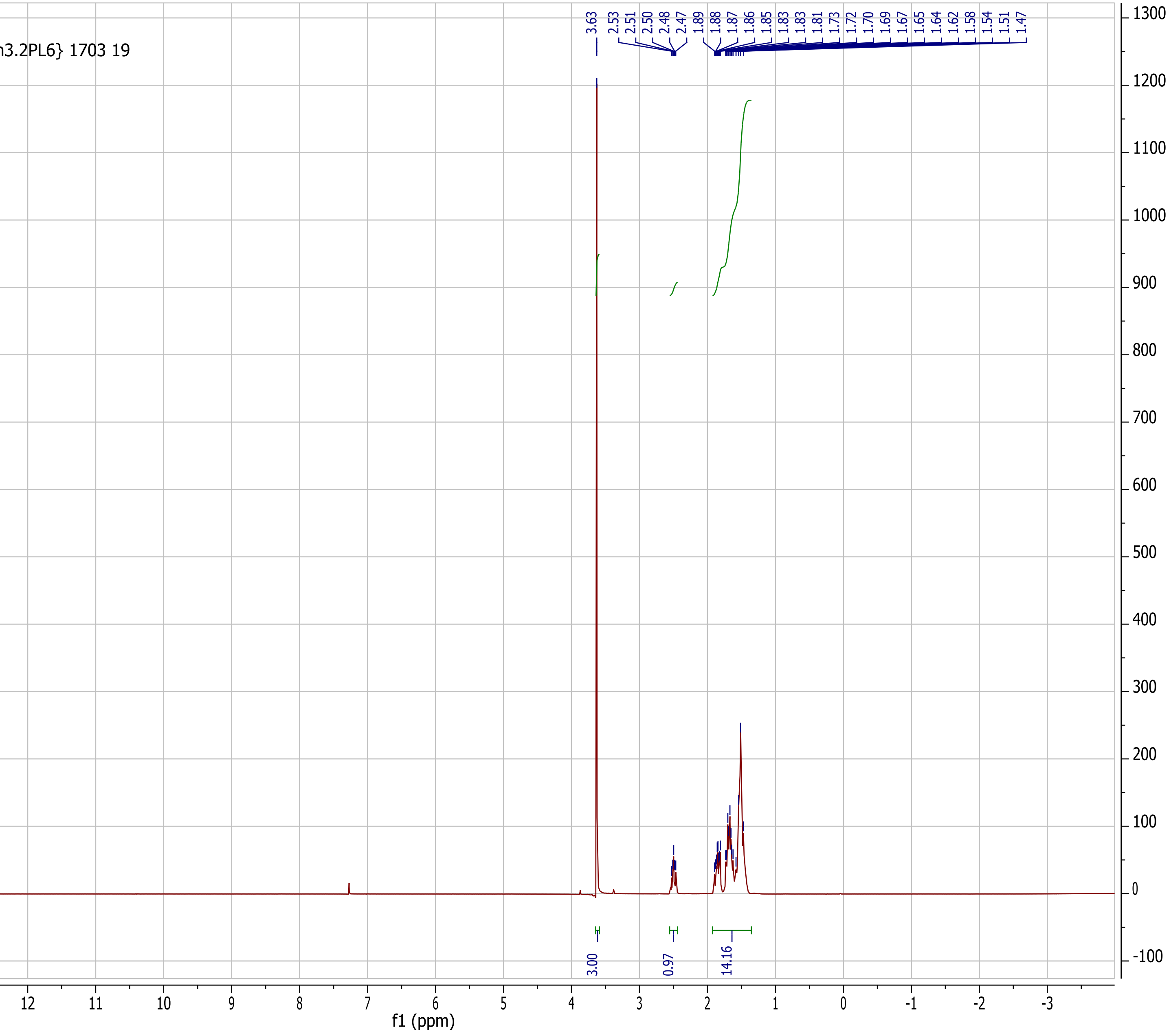

170302.f319.10.fid
Jie Liu jliu-8-15-2
PROTON CDCl3 {C:\Bruker\TopSpin3.2PL6} 1703 19
3.63
2.53
2.51
2.50
2.48
2.47
1.89
1.88
1.87
1.86
1.85
1.83
1.83
1.81
1.73
1.72
1.70
1.69
1.67
1.65
1.64
1.62
1.58
1.54
1.51
1.47
3.00
0.97
14.16
f1 (ppm)

[Type text]

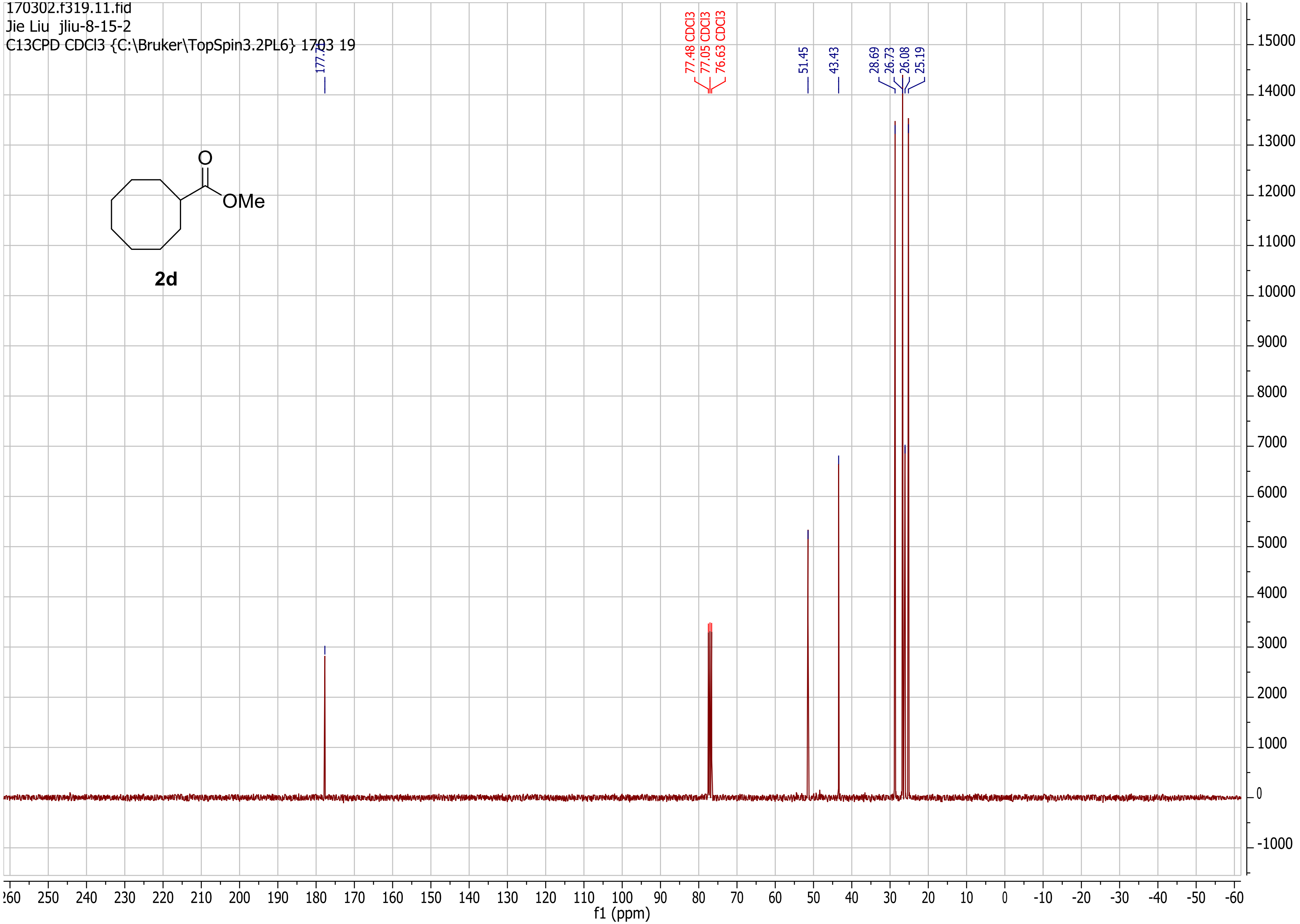
170302.f319.11.fid
Jie Liu jliu-8-15-2
C13CPD CDCl3 {C:\Bruker\TopSpin3.2PL6} 1703 19
177.75
77.48 CDCl3
77.05 CDCl3
76.63 CDCl3
51.45
43.43
28.69
26.73
26.08
25.19
OMe
2d
f1 (ppm)

[Type text]

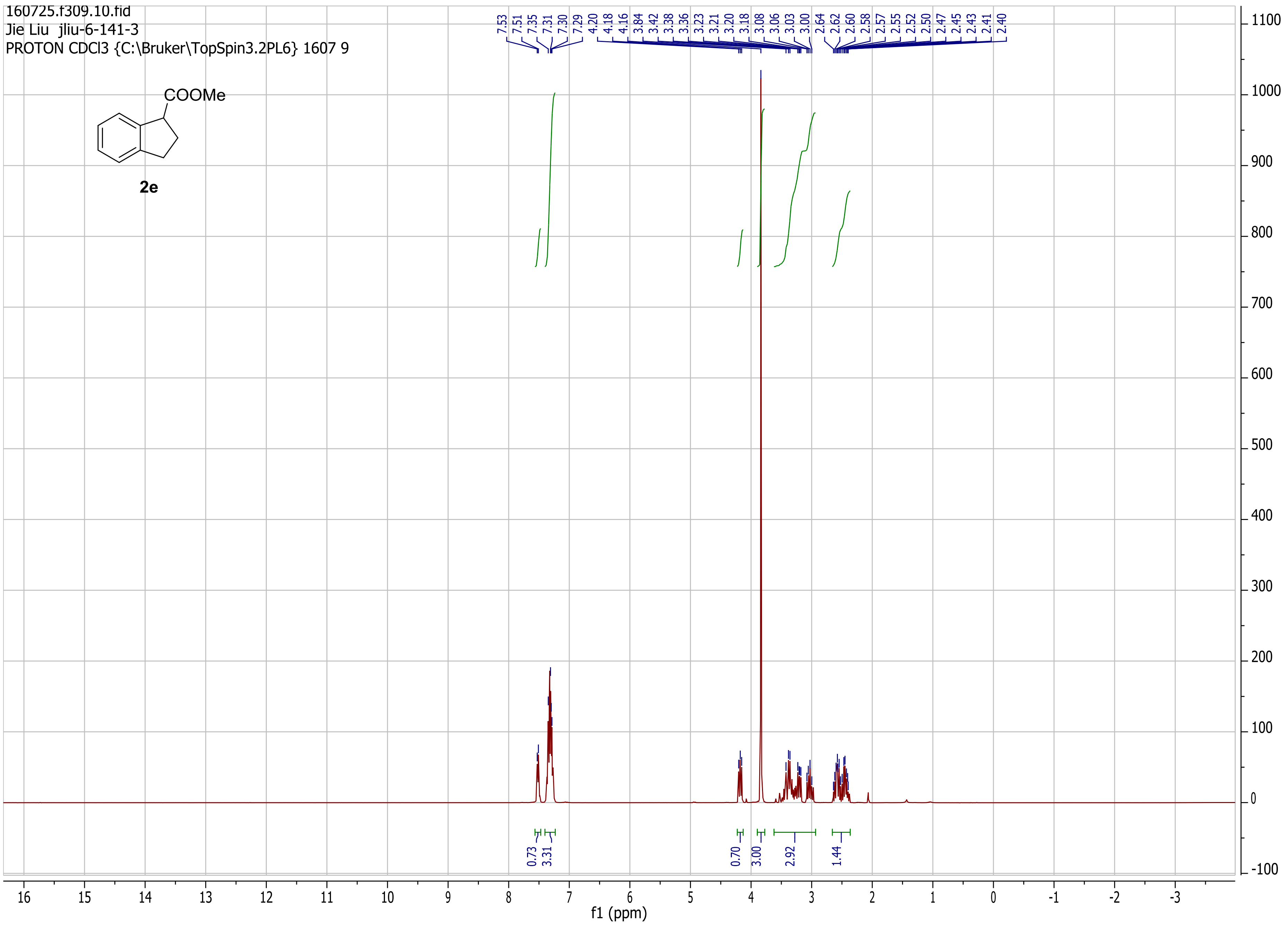
160725.f309.10.fid
Jie Liu jliu-6-141-3
PROTON CDCl3 {C:\Bruker\TopSpin3.2PL6} 1607 9
COOMe
2e
7.53 7.51 7.35 7.31 7.30 7.29 4.20 4.18 4.16 3.84 3.42 3.38 3.36 3.23 3.21 3.20 3.18 3.08 3.06 3.03 3.00 2.64 2.62 2.60 2.58 2.57 2.55 2.52 2.50 2.47 2.45 2.43 2.41 2.40
0.73 3.31 0.70 3.00 2.92 1.44
f1 (ppm)

[Type text]

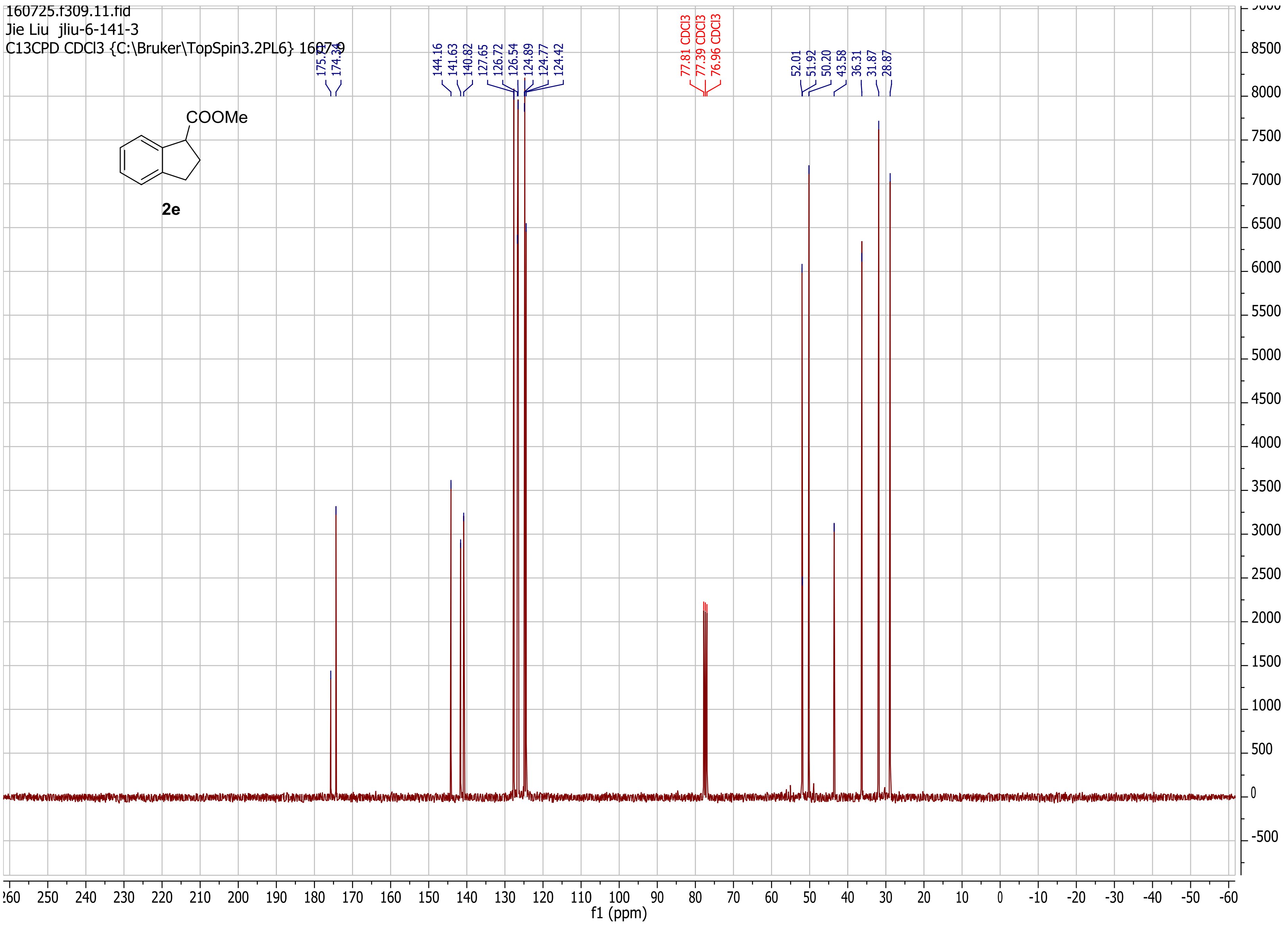

160725.f309.11.fid
Jie Liu jliu-6-141-3
C13CPD CDCl3 {C:\Bruker\TopSpin3.2PL6} 160729
175.74
174.36
144.16
141.63
140.82
127.65
126.72
126.54
124.89
124.77
124.42
77.81 CDCl3
77.39 CDCl3
76.96 CDCl3
52.01
51.92
50.20
43.58
36.31
31.87
28.87
COOMe
2e
f1 (ppm)

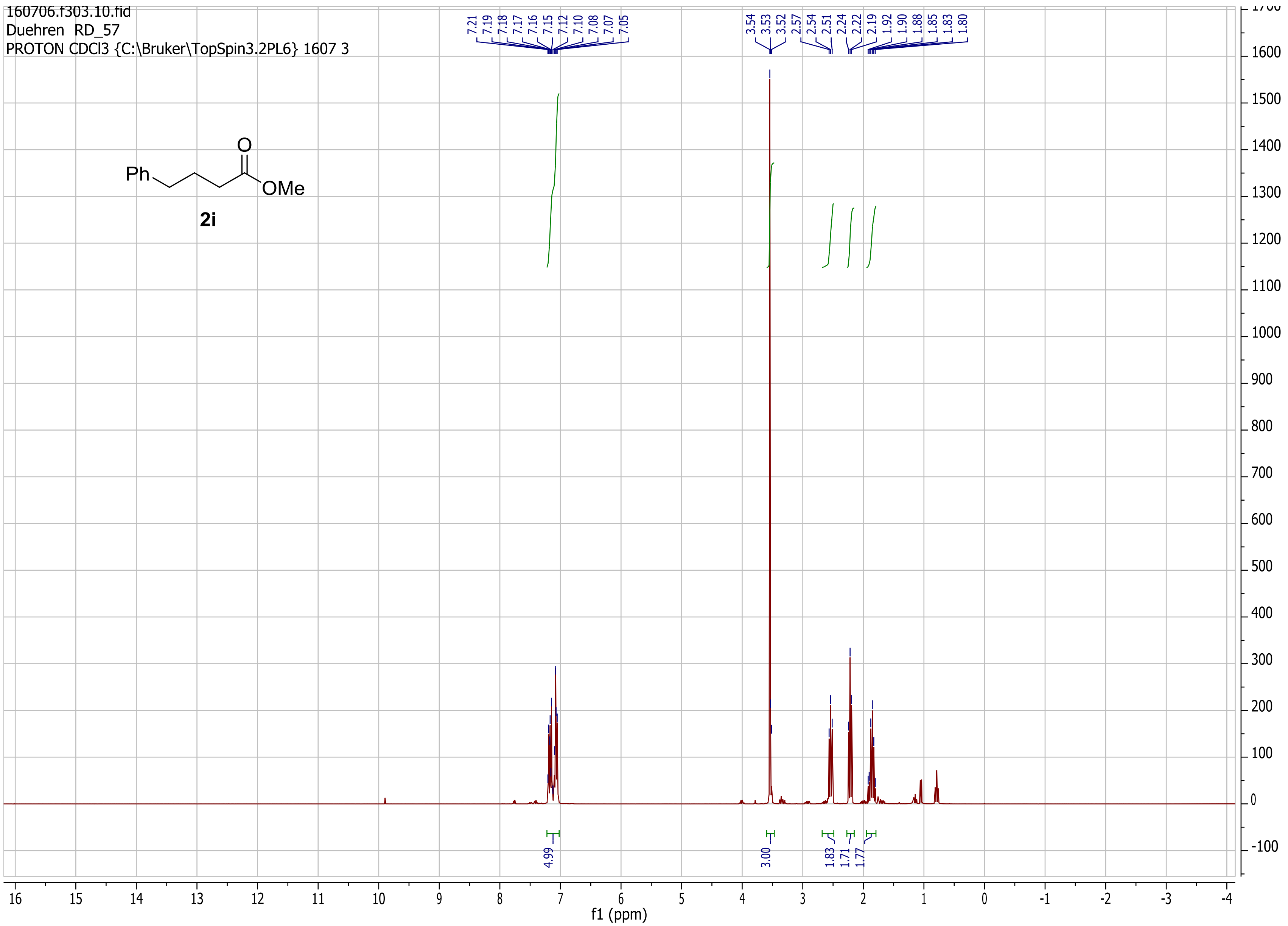
160706.f303.10.fid
Duehren RD_57
PROTON CDCl3 {C:\Bruker\TopSpin3.2PL6} 1607 3
Ph
O
OMe
2i
7.21
7.19
7.18
7.17
7.16
7.15
7.12
7.10
7.08
7.07
7.05
3.54
3.53
3.52
2.57
2.54
2.51
2.24
2.22
2.19
1.92
1.90
1.88
1.85
1.83
1.80
4.99
3.00
1.83
1.71
1.77
f1 (ppm)

[Type text]

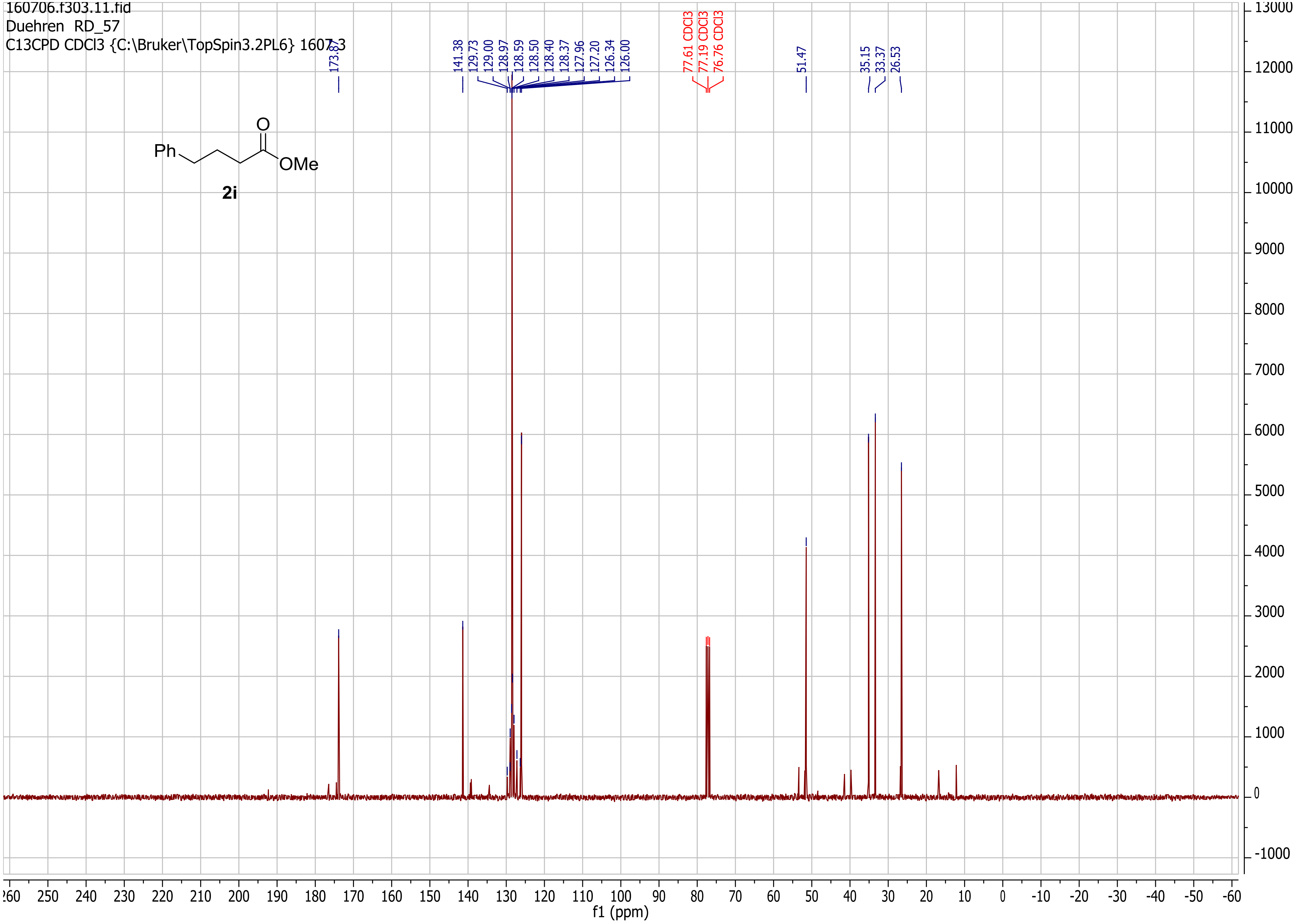

160706.f303.11.fid
Duehren RD_57
C13CPD CDCl3 {C:\Bruker\TopSpin3.2PL6} 1607_3
173.83
141.38
129.73
129.00
128.97
128.59
128.50
128.40
128.37
127.96
127.20
126.34
126.00
77.61 CDCl3
77.19 CDCl3
76.76 CDCl3
51.47
35.15
33.37
26.53
Ph
O
OMe
2i
f1 (ppm)

[Type text]

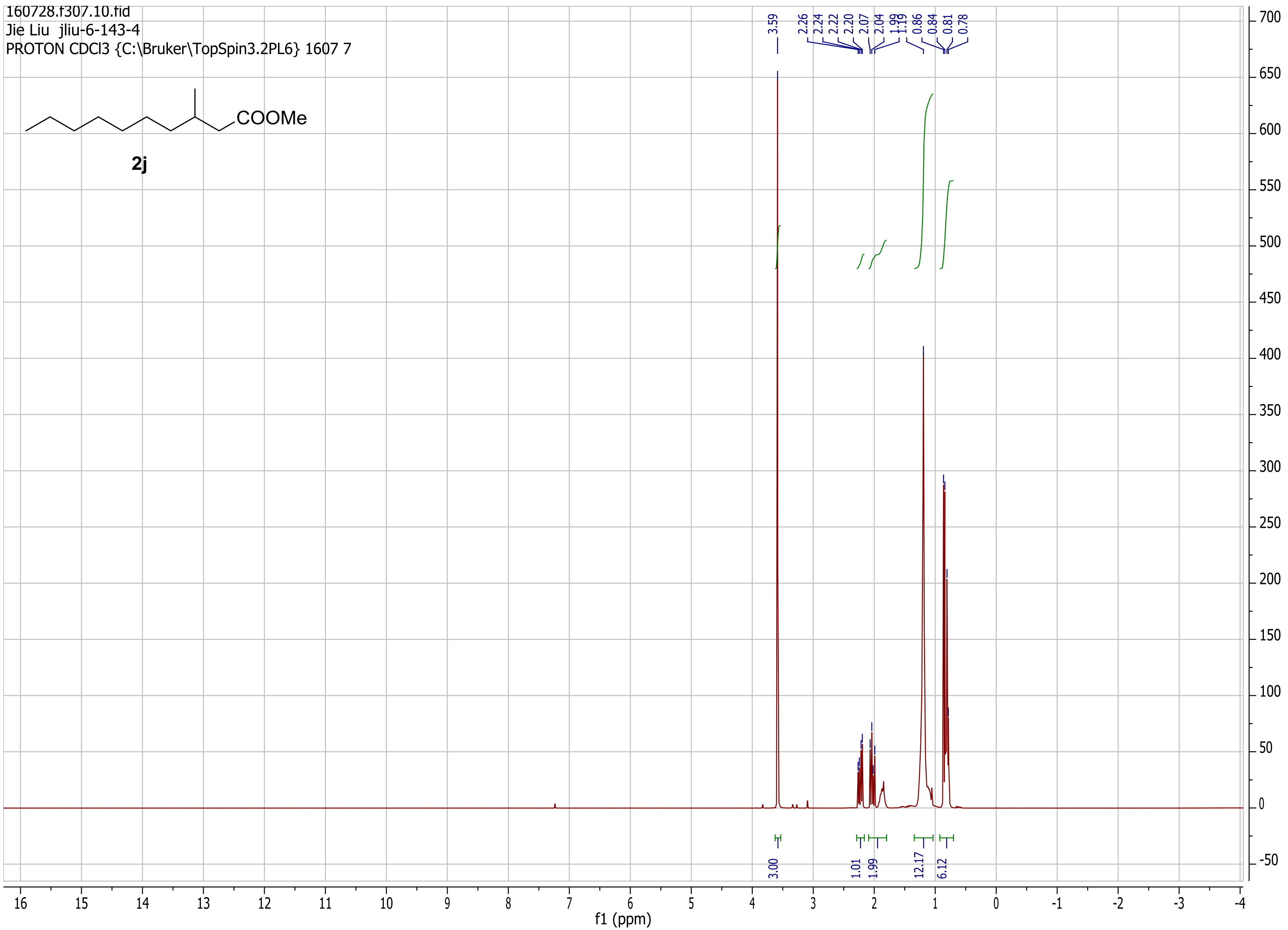

160728.f307.10.fid
Jie Liu jliu-6-143-4
PROTON CDCl3 {C:\Bruker\TopSpin3.2PL6} 1607 7
COOMe
2j
3.59
2.26
2.24
2.22
2.20
2.07
2.04
1.99
1.19
0.86
0.84
0.81
0.78
3.00
1.01
1.99
12.17
6.12
f1 (ppm)

[Type text]

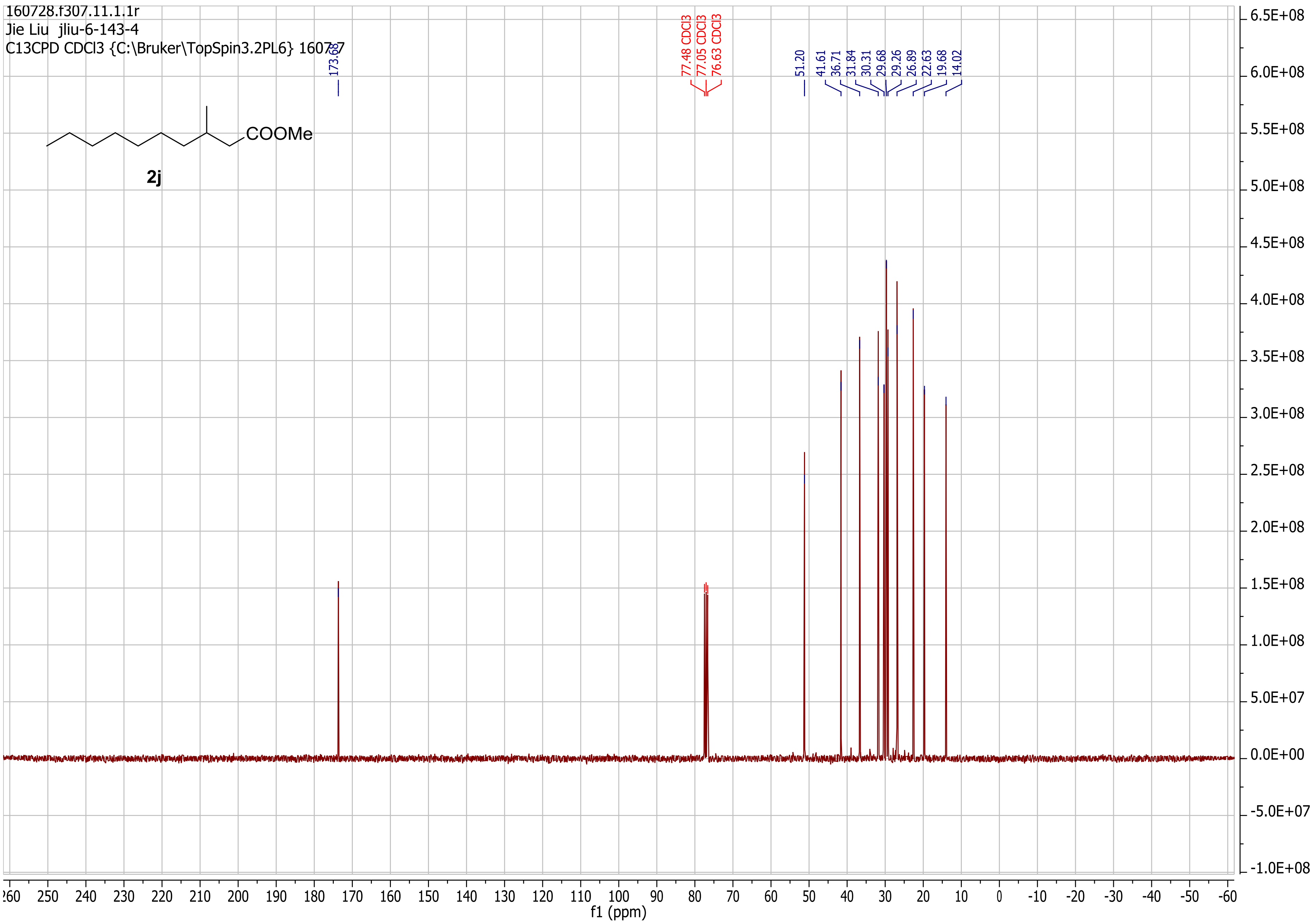
160728.f307.11.1.1r
Jie Liu jliu-6-143-4
C13CPD CDCl3 {C:\Bruker\TopSpin3.2PL6} 160727
173.68
77.48 CDCl3
77.05 CDCl3
76.63 CDCl3
51.20
41.61
36.71
31.84
30.31
29.68
29.26
26.89
22.63
19.68
14.02
COOMe
2j
f1 (ppm)

[Type text]

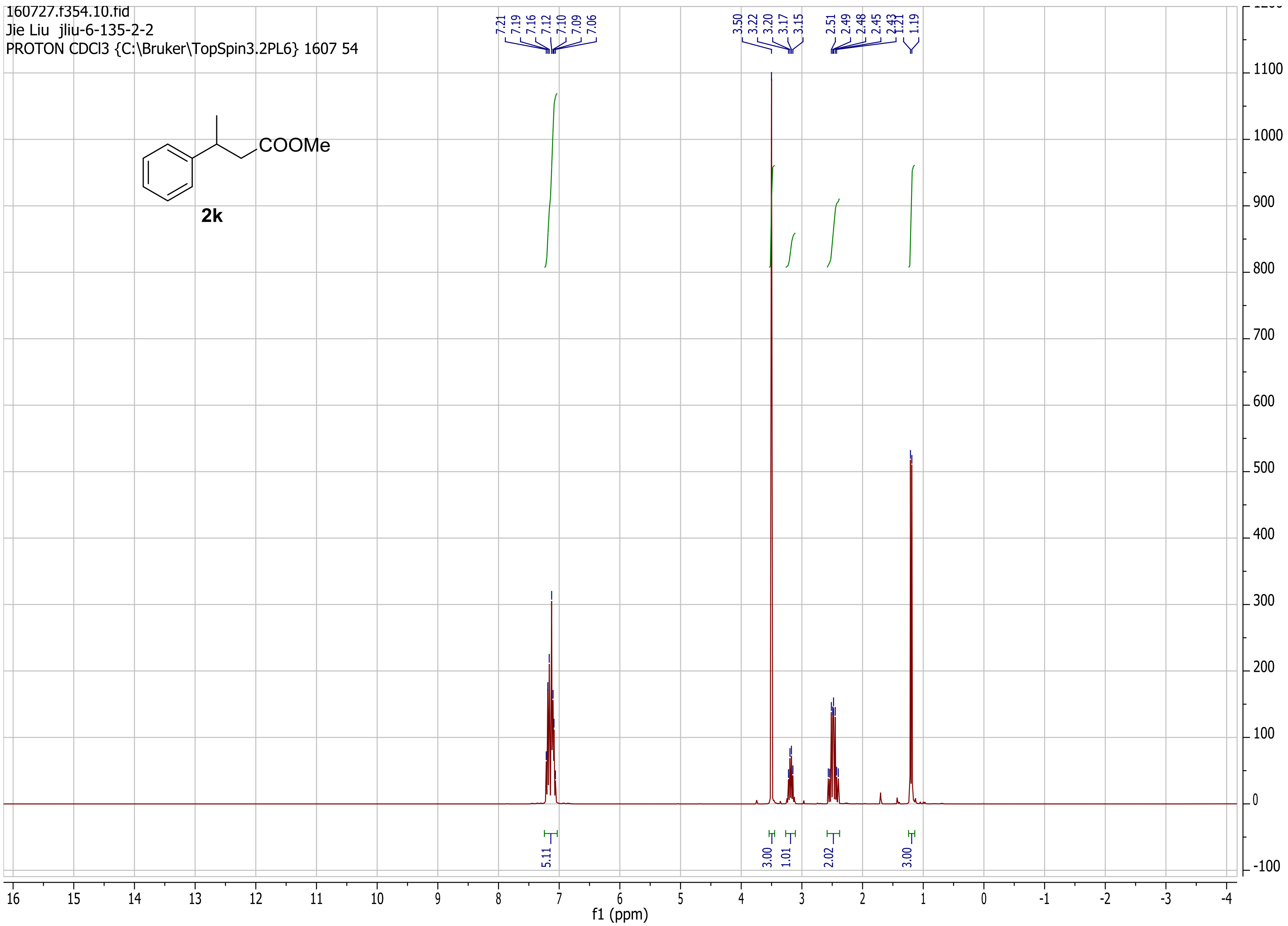
160727.f354.10.fid
Jie Liu jliu-6-135-2-2
PROTON CDCl3 {C:\Bruker\TopSpin3.2PL6} 1607 54
7.21
7.19
7.16
7.12
7.10
7.09
7.06
3.50
3.22
3.20
3.17
3.15
2.51
2.49
2.48
2.45
2.43
1.21
1.19
COOMe
2k
5.11
3.00
1.01
2.02
3.00
f1 (ppm)

[Type text]

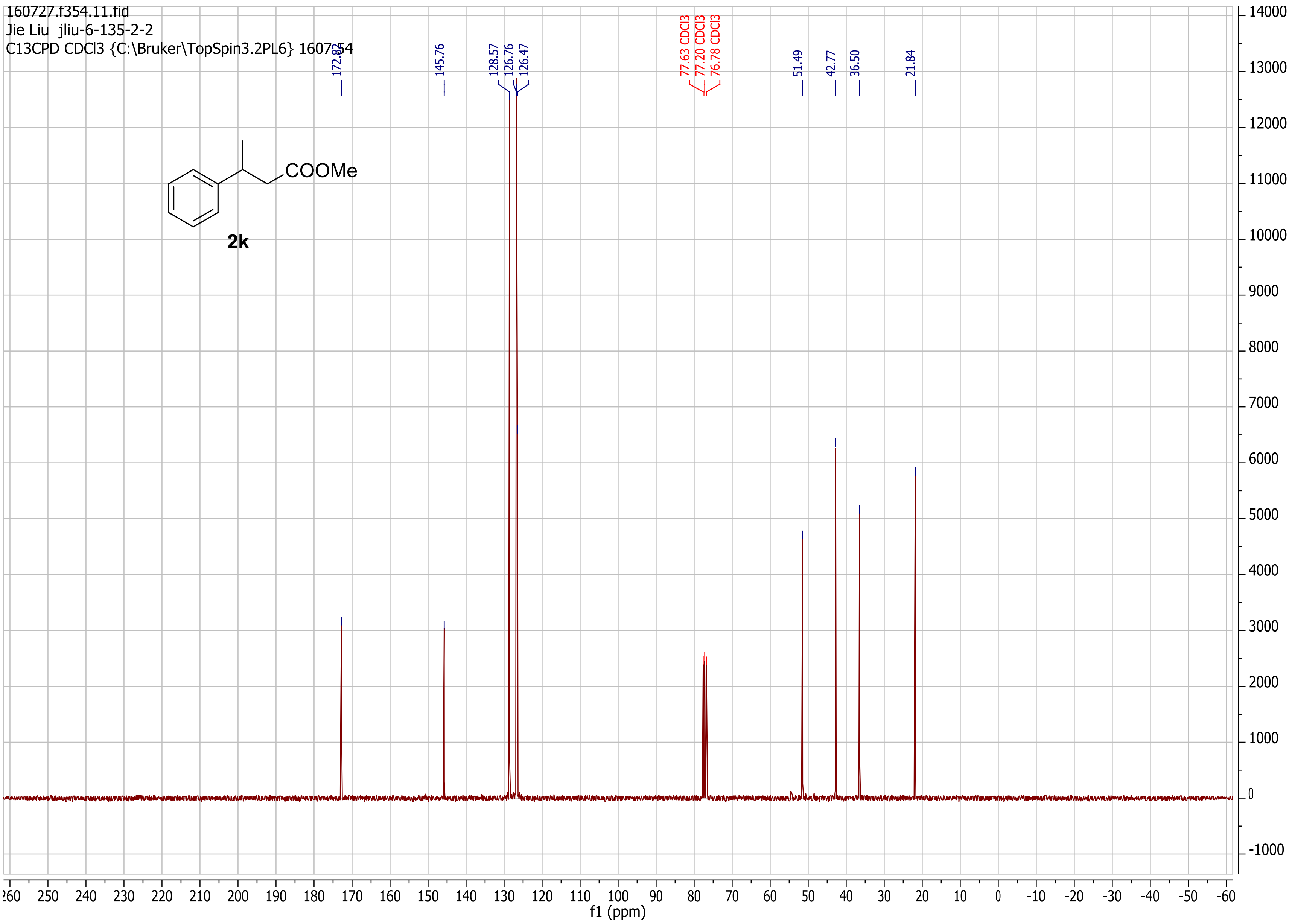

160727.f354.11.fid
Jie Liu jliu-6-135-2-2
C13CPD CDCl3 {C:\Bruker\TopSpin3.2PL6} 160754
172.87
145.76
128.57
126.76
126.47
77.63 CDCl3
77.20 CDCl3
76.78 CDCl3
51.49
42.77
36.50
21.84
COOMe
2k
f1 (ppm)

[Type text]

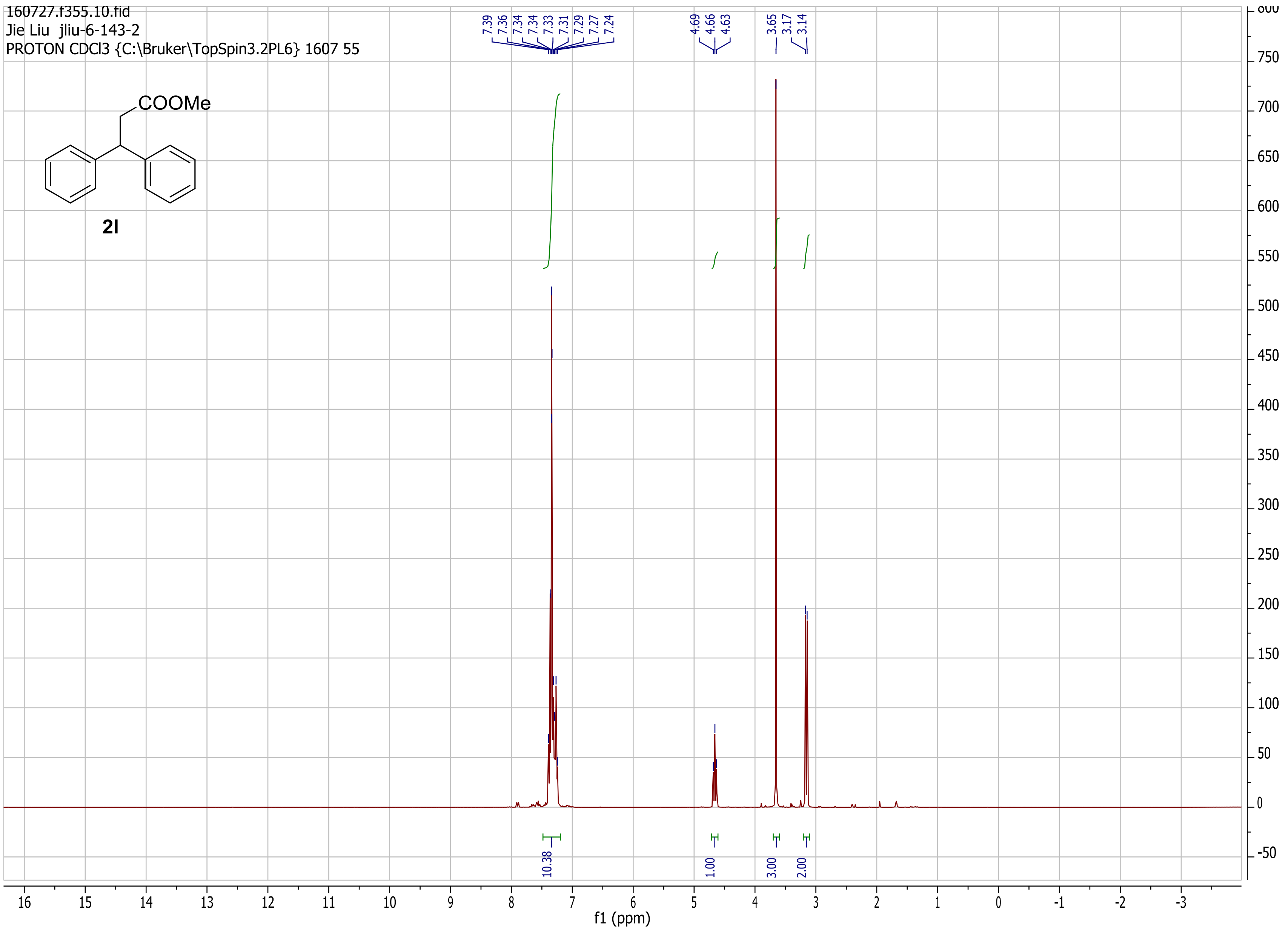
160727.f355.10.fid
Jie Liu jliu-6-143-2
PROTON CDCl3 {C:\Bruker\TopSpin3.2PL6} 1607 55
COOMe
2l
7.39
7.36
7.34
7.34
7.33
7.31
7.29
7.27
7.24
4.69
4.66
4.63
3.65
3.17
3.14
10.38
1.00
3.00
2.00
f1 (ppm)

[Type text]

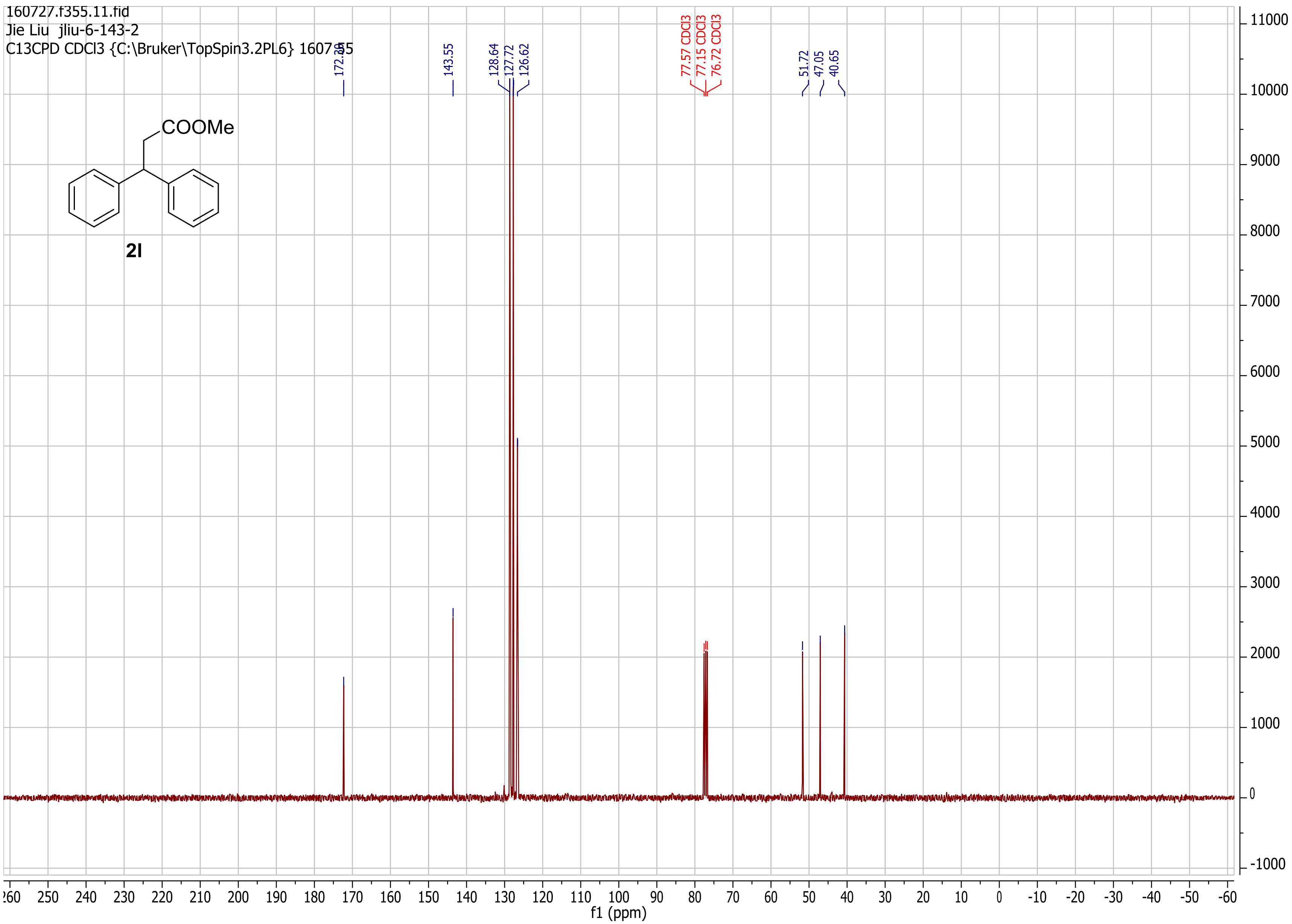

160727.f355.11.fid
Jie Liu jliu-6-143-2
C13CPD CDCl3 {C:\Bruker\TopSpin3.2PL6} 1607.55
172.30
143.55
128.64
127.72
126.62
77.57 CDCl3
77.15 CDCl3
76.72 CDCl3
51.72
47.05
40.65
COOMe
2l
f1 (ppm)

[Type text]

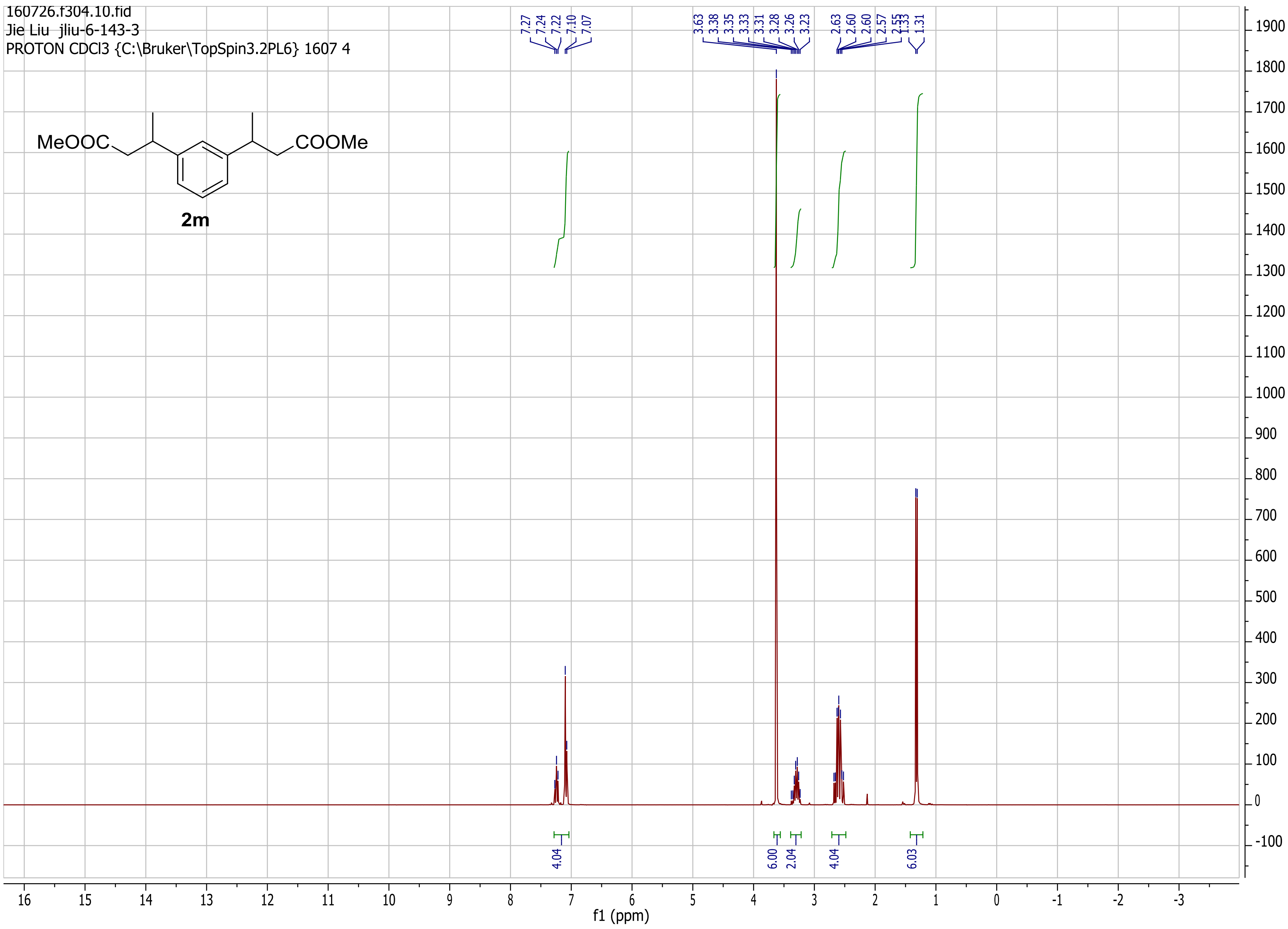
160726.f304.10.fid
Jie Liu jliu-6-143-3
PROTON CDCl3 {C:\Bruker\TopSpin3.2PL6} 1607 4
MeOOC
COOMe
2m
7.27
7.24
7.22
7.10
7.07
3.63
3.38
3.35
3.33
3.31
3.28
3.26
3.23
2.63
2.60
2.60
2.57
2.55
1.33
1.31
4.04
6.00
2.04
4.04
6.03
f1 (ppm)

[Type text]

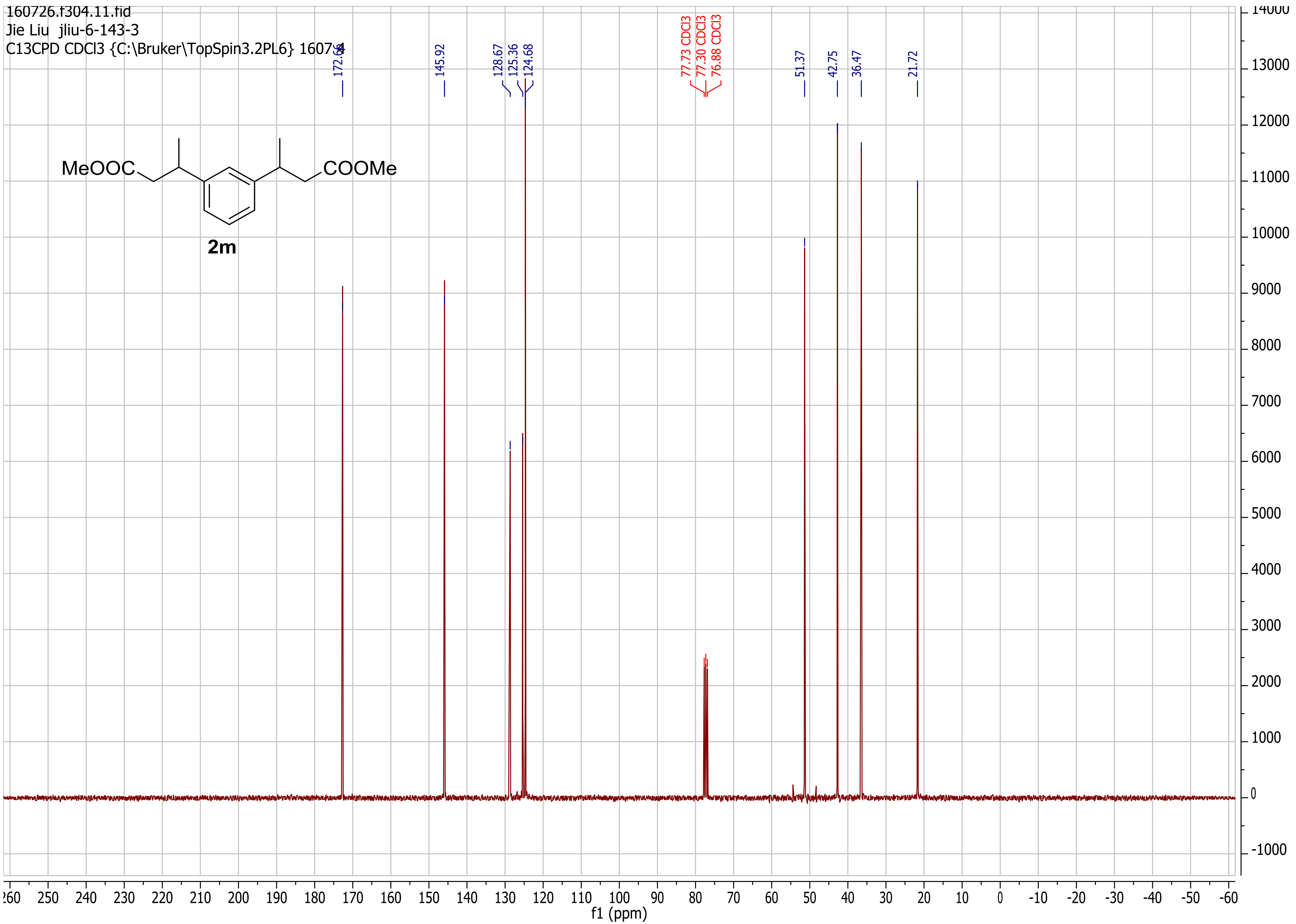
160726.f304.11.fid
Jie Liu jliu-6-143-3
C13CPD CDCl3 {C:\Bruker\TopSpin3.2PL6} 160724
172.69
145.92
128.67
125.36
124.68
77.73 CDCl3
77.30 CDCl3
76.88 CDCl3
51.37
42.75
36.47
21.72
MeOOC
COOMe
2m
f1 (ppm)

[Type text]

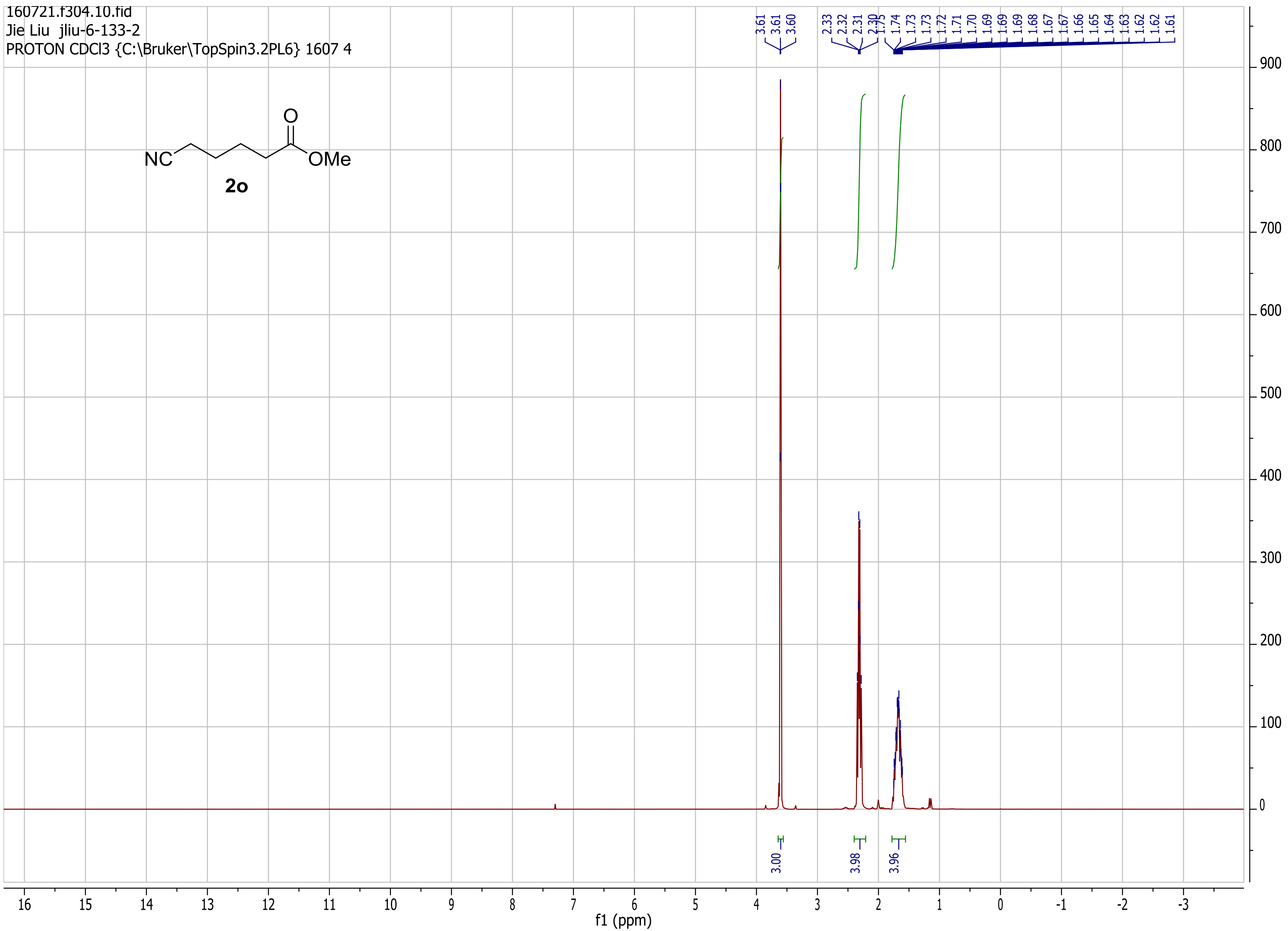

160721.f304.10.fid
Jie Liu jliu-6-133-2
PROTON CDCl3 {C:\Bruker\TopSpin3.2PL6} 1607 4
NC
O
OMe
2o
3.61
3.61
3.60
2.33
2.32
2.31
2.30
1.75
1.74
1.73
1.73
1.72
1.71
1.70
1.69
1.69
1.69
1.68
1.67
1.67
1.66
1.65
1.64
1.63
1.62
1.62
1.61
3.00
3.98
3.96
f1 (ppm)

[Type text]

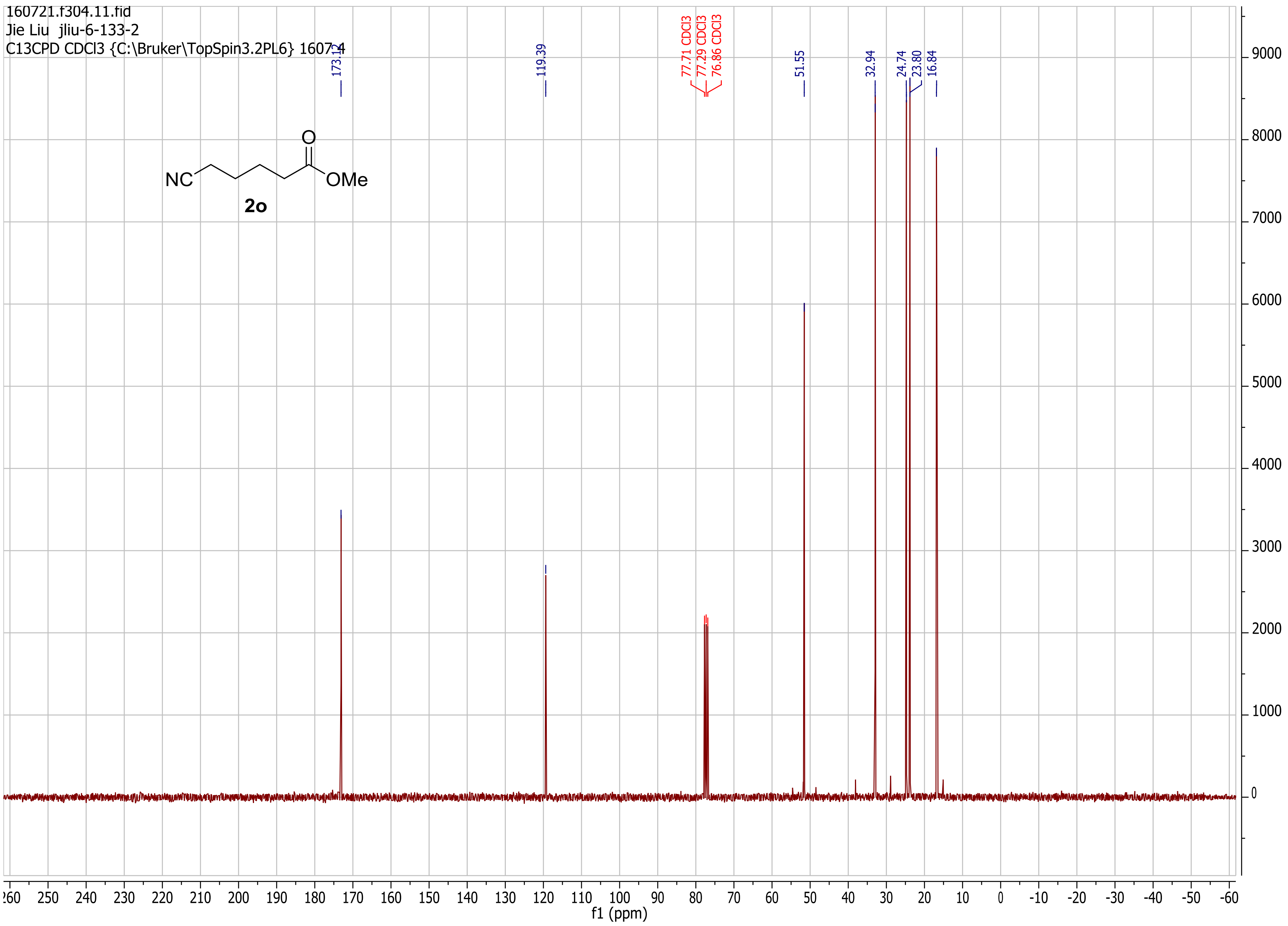
160721.f304.11.fid
Jie Liu jliu-6-133-2
C13CPD CDCl3 {C:\Bruker\TopSpin3.2PL6} 1607 4
173.12
119.39
77.71 CDCl3
77.29 CDCl3
76.86 CDCl3
51.55
32.94
24.74
23.80
16.84
NC
O
OMe
2o
f1 (ppm)

[Type text]

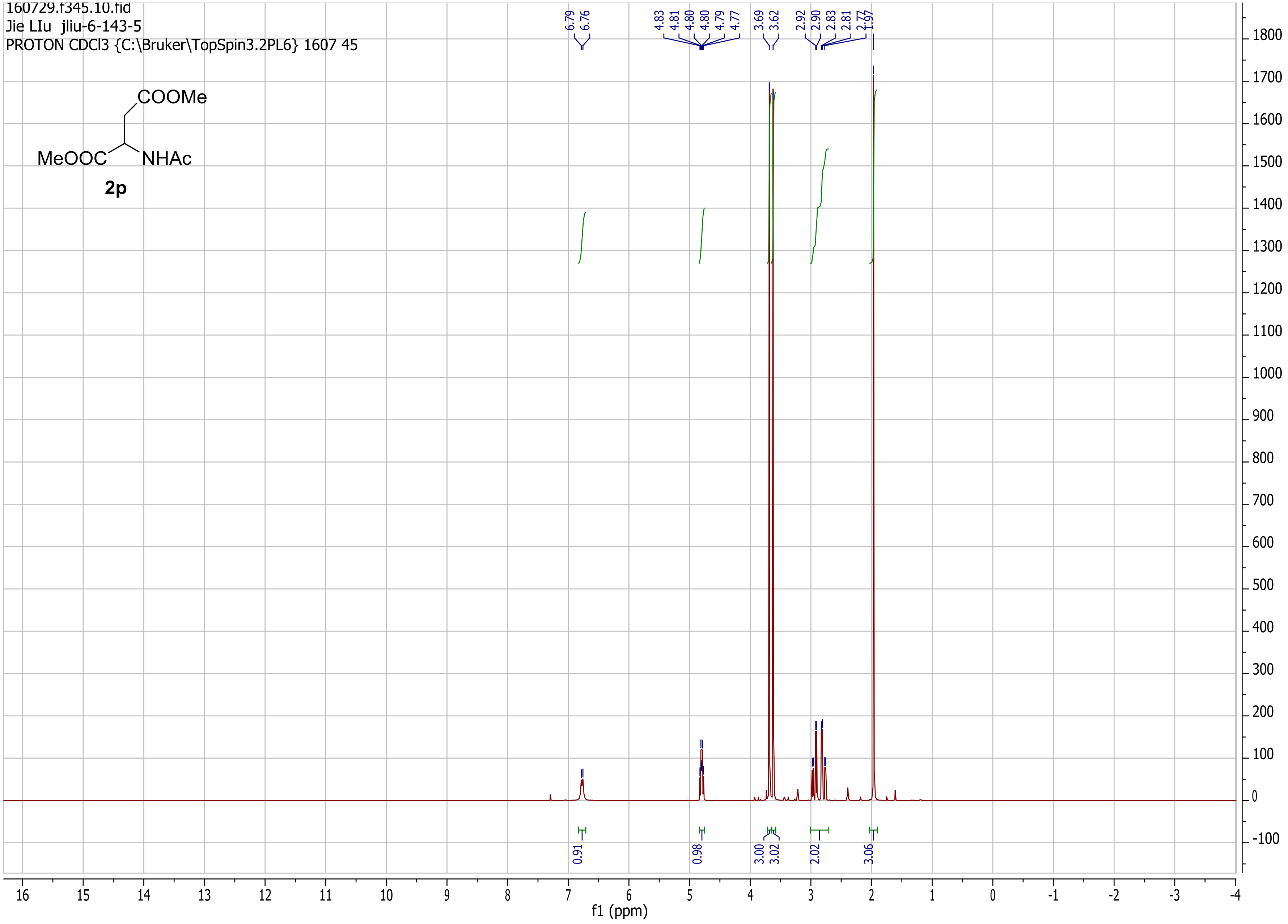
160729.f345.10.fid
Jie LIu jliu-6-143-5
PROTON CDCl3 {C:\Bruker\TopSpin3.2PL6} 1607 45
COOMe
MeOOC
NHAc
2p
6.79
6.76
4.83
4.81
4.80
4.80
4.79
4.77
3.69
3.62
2.92
2.90
2.83
2.81
2.77
1.97
0.91
0.98
3.00
3.02
2.02
3.06
f1 (ppm)

[Type text]

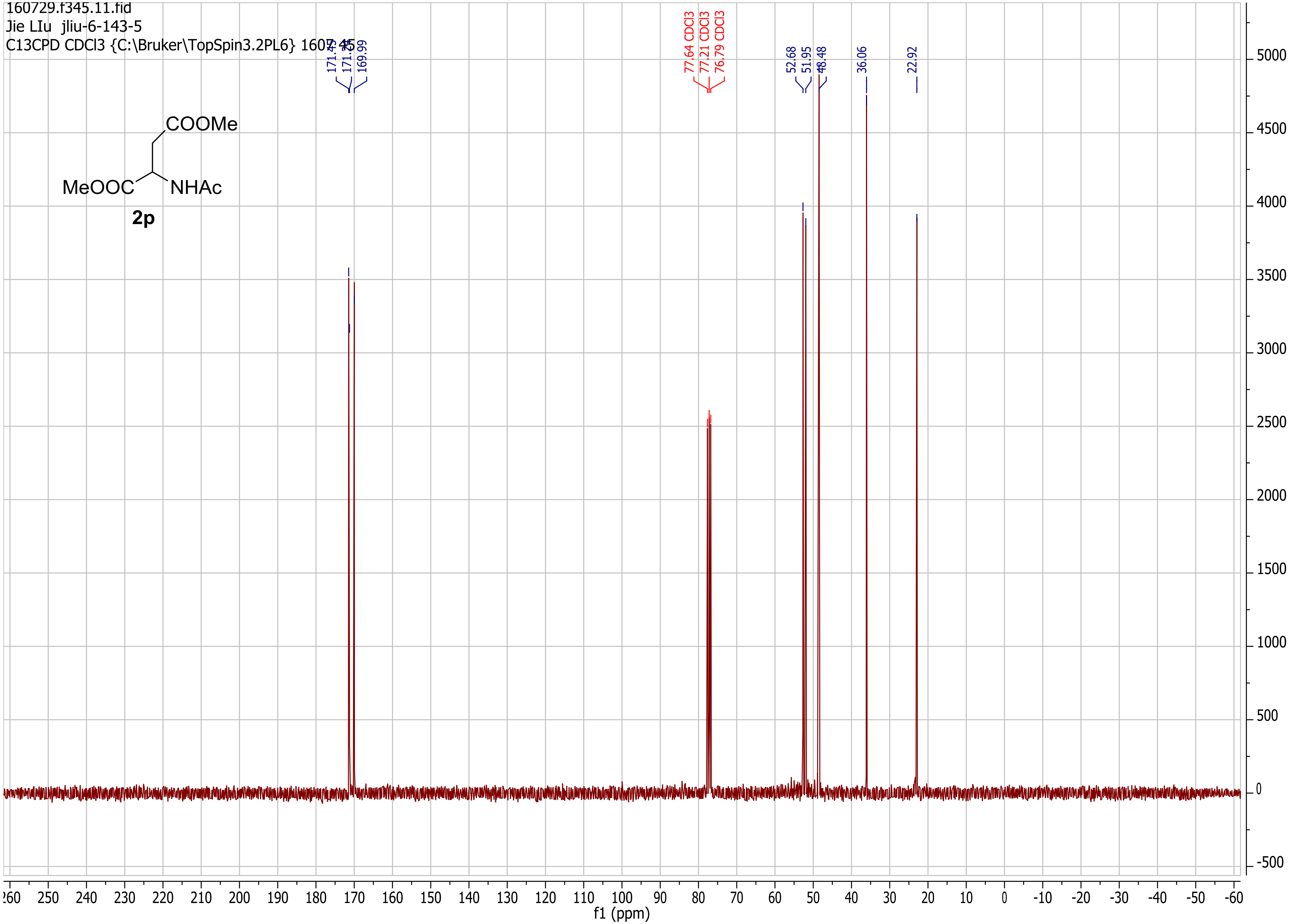
160729.f345.11.fid
Jie LIu jliu-6-143-5
C13CPD CDCl3 {C:\Bruker\TopSpin3.2PL6} 1607 45
171.49
171.45
169.99
77.64 CDCl3
77.21 CDCl3
76.79 CDCl3
52.68
51.95
48.48
36.06
22.92
COOMe
MeOOC
NHAc
2p
f1 (ppm)
